# QSAR analysis of substituent effects on tambjamine anion transporters† ‡
†Electronic supplementary information (ESI) available: Synthesis of new compounds, anion transport studies and details of the QSAR analysis. See DOI: 10.1039/c5sc03932k
‡The underlying research data for this paper are available in accordance with EPSRC open data policy from http://dx.doi.org/10.5258/SOTON/384138


**DOI:** 10.1039/c5sc03932k

**Published:** 2015-12-08

**Authors:** Nicola J. Knight, Elsa Hernando, Cally J. E. Haynes, Nathalie Busschaert, Harriet J. Clarke, Koji Takimoto, María García-Valverde, Jeremy G. Frey, Roberto Quesada, Philip A. Gale

**Affiliations:** a Chemistry , University of Southampton , Southampton , SO17 1BJ , UK . Email: philip.gale@soton.ac.uk ; Email: J.G.Frey@soton.ac.uk ; Tel: +44 (0)23 8059 3332; b Departamento de Química , Facultad de Ciencias , Universidad de Burgos , 09001 Burgos , Spain . Email: rquesada@ubu.es; c Organic and Polymeric Materials , Tokyo Institute of Technology , 2-12-1 O-okayama , Tokyo 152-8552 , Japan

## Abstract

The transmembrane anion transport activity of 43 synthetic molecules based on the structure of marine alkaloid tambjamine were assessed in model phospholipid (POPC) liposomes. The anionophoric activity of these molecules showed a parabolic dependence with lipophilicity, with an optimum range for transport efficiency. Using a quantitative structure–transport activity (QSAR) approach it was possible to rationalize these results and to quantify the contribution of lipophilicity to the transport activity of these derivatives. While the optimal value of log *P* and the curvature of the parabolic dependence is a property of the membrane (and so similar for the different series of substituents) we found that for relatively simple substituents in certain locations on the tambjamine core, hydrophobic interactions clearly dominate, but for others, more specific interactions are present that change the position of the membrane hydrophobicity parabolic envelope.

## Abstract 2

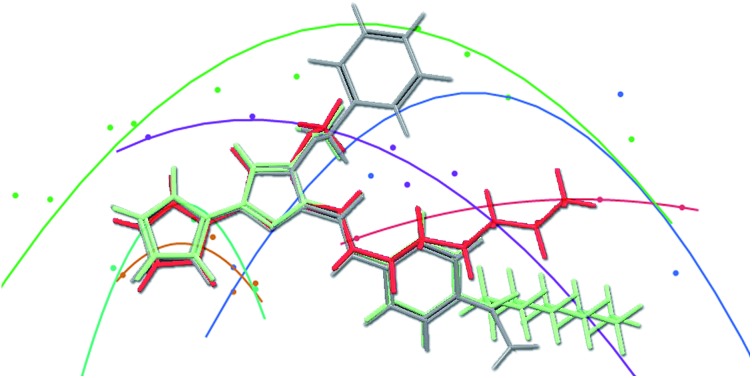
A QSAR analysis of the transmembrane anion transport activity of 43 synthetic tambjamine analogs allowed rationalization of this activity according to their lipophilicity and structural parameters.

## Introduction

The control of the transmembrane transport of ions is an essential function of living organisms. This control is essentially exerted by transmembrane proteins, although there are small lipophilic molecules (ionophores) capable of facilitating the transmembrane transport of ions.^1^,^2^ The vast majority of identified natural ionophores are cation selective. Nevertheless, anion transport is no less important and the characterization of the facilitated transmembrane anion transport by both natural and synthetic systems is receiving increasing attention.^3^–^8^ These molecules could have potential in the treatment of conditions derived from the defective regulation of chloride and bicarbonate transport such as cystic fibrosis or Bartter's syndrome.^9^,^10^ Moreover naturally occurring cationophores find applications as antimicrobials and biomembrane research tools, thus, anion selective ionophores could find similar applications.

Among the identified naturally occurring anionophores, the structurally related prodiginines and tambjamine alkaloids are the most studied examples.^11^ These compounds show interesting pharmacological properties including antitumor activity.^12^,^13^ The synthetic prodiginine analogue obatoclax has been shown to display promising anticancer activity in the clinic.^14^ We have demonstrated that the ionophoric activity of these compounds is related to their cytotoxicity.^15^ Active ionophores are able to disrupt intracellular pH gradients and to trigger apoptosis in cancer cells.^16^–^19^

An increasing number of synthetic molecules capable of facilitating anion transport by forming lipophilic supramolecular complexes or membrane spanning channels have been reported in the literature.^20^–^23^ Despite this progress, the knowledge of the requirements for designing effective anion transporters remains poor, and identification of active derivatives is mostly based on trial/error methods. Qualitative structure–transport activity studies underscored lipophilicity as one of the most important factors influencing the ionophoric transport activity of these compounds.^24^ Moreover, Gale, Davis and co-workers have also introduced the concept of lipophilic balance in the design of these compounds.^25^ Quantitative structure–activity relationship (QSAR) approaches are widely employed in medicinal chemistry. QSAR constitutes a powerful tool to assist rational molecular design and to predict different physicochemical properties.^26^ Recently, we have reported a quantitative structure–transport activity (QSAR) study of the anion binding and transport of a series of 1-hexyl-3-phenylthioureas bearing various substituents at the *para*- positions of the aromatic ring.^27^ This study allowed us to determine a statistically relevant model correlating anion transport activity with parameters such as lipophilicity, the Hammett coefficient of the varied substituent and SPAN, a descriptor for molecular size. Prompted by this success we decided to perform a more ambitious study introducing several structural changes on the studied molecules. We aimed to investigate a series of effective anion transporters having a range of lipophilicity values as well as transport activities. In this regard, the tambjamine alkaloids represent ideal candidates because of their synthetic accessibility and tolerance to different substituents while remaining as potent transmembrane anion transporters. In this work we present a QSAR study of the transmembrane anion transport activity of 43 tambjamine inspired transporters, aimed to shed light on the structural design requirements to successful anion carriers and the quantification of the relationships between lipophilicity and transmembrane anion transport activity of small molecules.

## Results and discussion

**Fig. 1:**
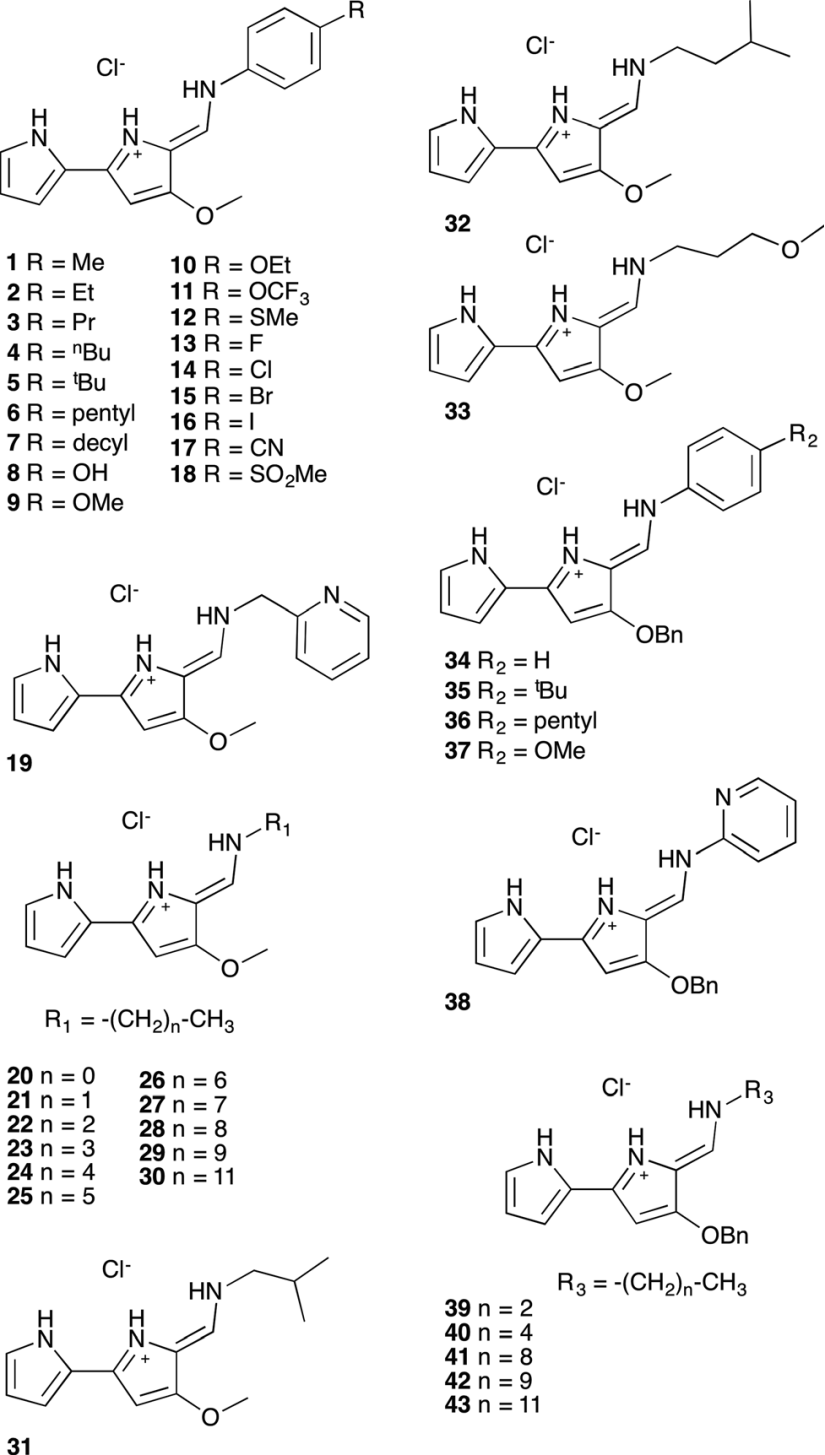
Compounds included in this study.

A series of tambjamine derivatives **1–43** were selected for this study (Fig. 1). Tambjamines are marine alkaloids containing a 4-methoxy-2,2′-bipyrrole core. Some of the studied compounds are natural products such as tambjamine B (**20**), tambjamine C (**31**), tambjamine K (**32**) or BE-18591 (**30**), whereas others are synthetic tambjamine analogues. With this selection we aimed to create a library of compounds including systematic variations on the enamine substituent and also to explore the possibility of replacing the –OMe group characteristic of naturally occurring derivatives by a benzyloxy group. The synthesis of these compounds is straightforward from the appropriate bipyrrolealdehyde.^28^ Compounds **5**, **9**, **20–32**, **34**, **35**, **37–40** and **42** have been previously reported and all of them were characterized by standard methods.^29^

### Anion transport assays

In order to measure the transmembrane transport activity of compounds **1–43**, the chloride efflux from 1-palmitoyl-2-oleoyl-*sn*-glycero-3-phosphocholine (POPC) chloride containing vesicles was monitored over time using a chloride selective electrode, according to reported methods.^30^ Briefly, 200 nm POPC liposomes containing chloride (489 mM NaCl, 5 mM phosphate buffer pH 7.2) were prepared. The vesicles were then suspended in an isotonic nitrate solution (489 mM NaNO_3_, 5 mM phosphate buffer pH 7.2) and the studied compound added as a DMSO solution (typically 10 μL or less to avoid any influence in the outcome of the experiment). Chloride release is then monitored over 300 s using a chloride selective electrode. A final reading, considered to be 100% chloride release, was obtained after addition of detergent to lyse the vesicles. The transport assays were repeated at different carrier concentrations. The data was subjected to Hill analyses in order to obtain a quantitative measure of the transporter efficiency.^31^ Thus the effective concentrations required to induce 50% of chloride efflux in the time scale of the experiments (300 s) were calculated (EC_50_, Table 1). Hill analyses also provided the Hill parameter *n* values. The Hill parameters were all consistent with a mobile carrier mechanism.^32^ All the studied compounds were found to be highly active anion carriers, with EC_50_ values of 0.003–0.346 mol% carrier/lipid. The initial rate of chloride release (*k*_ini_) was also calculated for carrier loadings of 0.05 mol% compound to POPC. An overview of all these data is provided in Table 1.

**Table 1:** Overview of transmembrane anion transport data: EC_50_, *n*, initial rate of chloride release (*k*_ini_), log *P* and retention times^*a*^

Compound		EC_50_^*b*^	Hill parameter *n*	*k*_ini_ ^*c*^	log *P*^*d*^	Retention time (min)
**1**		0.00719	1.19	0.952	3.08	10.4
**2**		0.00613	1.23	1.41	3.74	11
**3**		0.00699	1.25	1.24	4.17	11.6
**4**		0.00779	1.32	1.17	4.63	12.2
**5**		0.0104	1.29	1.02	4.72	11.9
**6**		0.00951	1.25	1.13	5.02	12.7
**7**		0.288	0.965	0.0231	7.11	14.5
**8**		0.0688	1.42	0.229	2.58	8.8
**9**		0.0197	1.29	0.638	2.86	9.8
**10**		0.0134	1.28	0.786	3.37	10.5
**11**		0.0231	1.31	0.470	3.76	11
**12**		0.0260	1.29	0.494	3.2	10.4
**13**		0.0208	1.18	0.474	2.92	9.6
**14**		0.0155	1.27	0.661	3.49	10.3
**15**		0.0236	1.37	0.444	3.62	10.5
**16**		0.0221	1.29	0.510	3.76	10.8
**17**		0.0167	1.48	0.830	2.68	9
**18**		0.0494	1.59	0.314	2.11	8.2
**19**		0.197	0.853	0.0919	1.88	n.d.
**20**		0.346	1.30	0.0368	1.03	7
**21**		0.0921	1.08	0.215	1.55	7.7
**22**		0.0274	1.03	0.517	2.03	8.5
**23**		0.0116	0.860	0.743	2.46	9.3
**24**		0.00648	1.18	1.46	2.99	10.2
**25**		0.005	1.19	1.50	3.52	10.9
**26**		0.00451	1.51	1.52	4.02	11.5
**27**		0.00312	1.07	2.63	4.79	12.1
**28**		0.0038	1.10	1.63	5.1	12.6
**29**		0.0053	1.33	1.54	5.36	13.1
**30**		0.00731	1.15	1.09	6.14	13.8
**31**		0.0113	1.20	0.941	2.24	9.2
**32**		0.00668	1.05	1.01	2.84	10
**33**		0.0977	0.963	0.224	1.5	n.d.
**34**		0.0157	1.32	0.744	4.4	11.5
**35**		0.0116	1.20	0.708	5.94	13.1
**36**		0.0123	0.857	0.321	6.46	n.d.
**37**		0.0133	1.45	0.600	4.38	11.6
**38**		0.00878	1.43	1.69	3.3	11.3
**39**		0.0196	1.14	0.605	3.62	10.7
**40**		0.00968	1.74	1.16	4.49	11.9
**41**		0.00517	1.15	1.04	6.07	n.d.
**42**		0.0204	0.929	0.420	6.42	13.9
**43**		0.0616^*e*^	—^*e*^	0.186	7.14	10.4

^*a*^n.d. not determined.

^*b*^molar percentage with respect to POPC, mol%.

^*c*^Values calculated by fitting the plot of relative chloride release (*y*) *versus* time (*x*) for 0.05 mol% compound to lipid to an asymptotic function *y* = *a* – *b* × *c*^*x*^. The initial rate of chloride release (*k*_ini_ in % s^–1^) is given by –*b* ln(*c*).

^*d*^Log *P* values calculated using ALOGPs 2.1 software.

^*e*^Determined *via* correlation between *k*_ini_ and EC_50_ (see ESI).

### Quantitative analysis of transmembrane anion transport

Quantitative structure–transport activity (QSAR) studies represent a commonly employed approach to modelling physical and biological properties of compounds.^26^,^33^ This approach is a powerful tool for structure optimization and targeted design of new compounds. The objective of a QSAR study is the construction of a statistically relevant model. Using a combination of software sources: ALOGPS 2.1 and e-dragon 1.0,^34^,^35^ (which gave constitutional descriptors, topological descriptors, topological charge indices, geometrical descriptors, WHIM descriptors, charge descriptors and molecular properties), Chemicalize,^36^ ACDiLabs 2.0,^37^ TorchV10lite^38^ and ChemBioDraw 12.0 ultra software^39^ a total of 506 descriptors were calculated. Based on our previous observations, we identified lipophilicity as an important parameter determining the transmembrane transport efficiency of a given transporter.^27^ In order to obtain an experimental measure of this property, the retention times (RT) of all compounds were measured using reverse phase HPLC. In this assay, lipophilic compounds show higher retention times whereas hydrophilic compounds are eluted more quickly.^40^ These experiments are used as an indirect measure of the lipophilicity. On the other hand, log *P*, the octanol–water partition coefficient, is the more employed quantitative measure of lipophilicity. The importance of this parameter^41^ in medicinal chemistry and drug discovery has led to the development of several software packages to predict the log *P* values without the need of experimentally time consuming measures. Moreover, these predictions allow the calculation of log *P* values of virtual compounds. Simple correlations of the measured RT and the different calculated log *P* values showed an excellent agreement (see ESI,† log *P*_RT_correlations.pdf).^40^ This correlation supported the validity of computationally obtained log *P* values for these compounds. The best correlation was found for the calculated ALOGPs values using the ALOGPs 2.1 software, therefore ALOGPs descriptor was selected as the best log *P* descriptor. Those values are shown in Table 1 along with RT data.

**Fig. 2:**
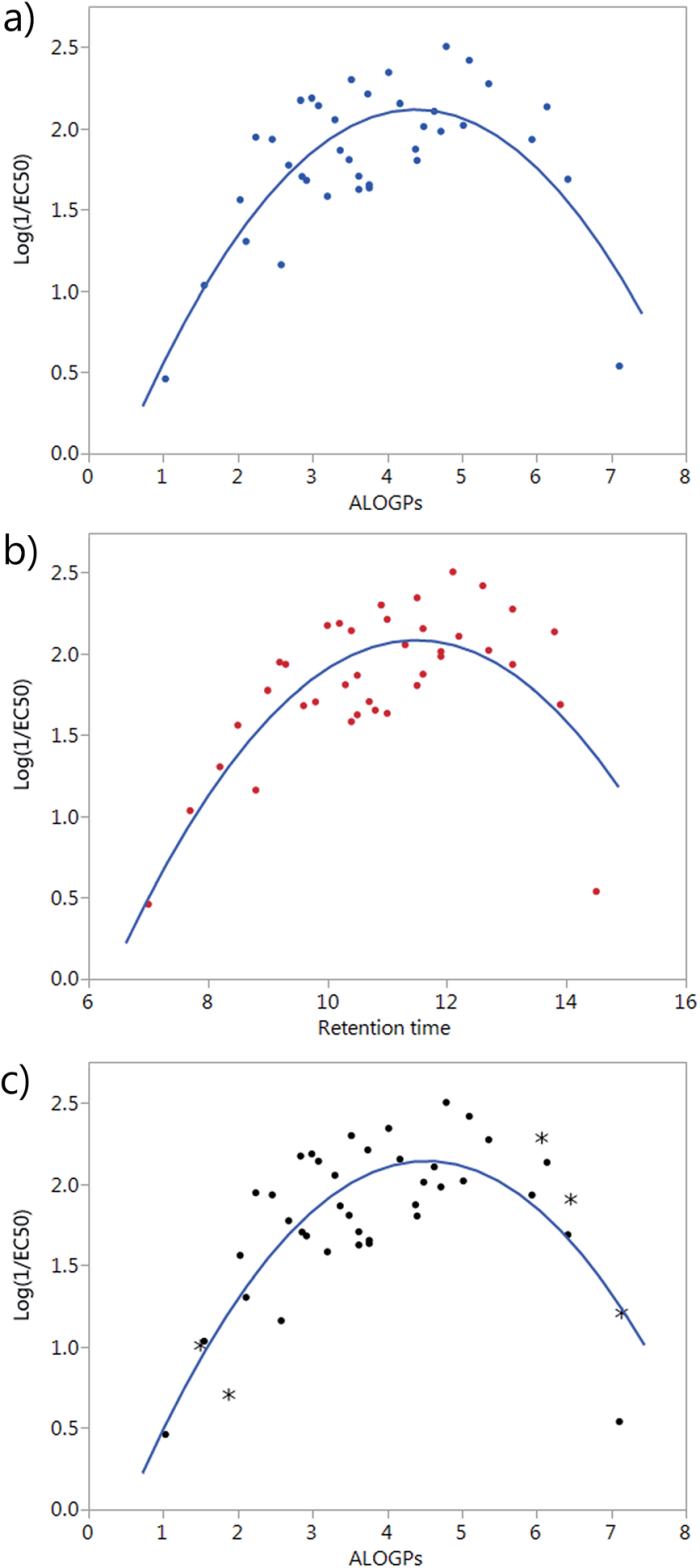
(a) Plot of log(1/EC_50_) *vs.* ALOGPs of the first set of 38 compounds; (b) plot of log(1/EC_50_) *vs.* RT of the first 38 compounds; (c) plot of log(1/EC_50_) *vs.* ALOGPs showing all 43 compounds, the additional molecules are highlighted by *.

A simple plot of the transport activity, expressed as log(1/EC_50_), *vs.* ALOGPs or retention time (RT) suggested a parabolic dependence of these variables (Fig. 2a and b). The rationale behind this observation is that there is an optimum compromise in the hydrophilicity/hydrophobicity balance, which maximizes the transmembrane transport activity of a given compound.^24^ A too hydrophilic transporter would not partition into the phospholipid membrane whereas a too hydrophobic derivative would not be able to move away from the membrane core and thus act as a carrier. At the beginning of the modelling part of this study, a set of 38 compounds had been synthesized. However, the majority of these compounds were present in the middle of the explored ALOGPs range (values from 2–6) with only a few compounds above or below this range. Therefore, the need of including further compounds, having low and high log *P* values, to confirm this parabolic dependence and to avoid an excessive leverage of data corresponding to compounds displaying low activity and extreme log *P* values was evident. Compounds of a similar structure to the existing tambjamines were hypothesised and their ALOGPs values calculated. Those that fell in the ranges of 1–2.5 and 5–7.5 were considered suitable and suggested for synthesis. Thus, 5 additional tambjamine derivatives (numbers **19**, **33**, **36**, **41**, **43**) were synthesised and measured (the additional molecules are highlighted by * in Fig. 2c). Attempts to find simple correlations between the anion transport activity and the lipophilicity of tambjamine derivatives were not satisfactory. Therefore, it was evident that a more sophisticated analysis should be made.

### Data cleaning

Prior to running any QSAR analyses the descriptor dataset was cleaned. Descriptors were removed if they were incomplete with values unavailable for some of the molecules, if the values were classed as non-numeric or if the descriptors had little or no variation across the dataset. Following the cleaning of the dataset, a total of 330 descriptors remained (see ESI,† Tambjamines_dataset_cleaned.csv). The descriptor dataset still contained different calculated values of log *P*. Some descriptors are the square of another descriptor, *e.g.* ALOGPs-sq.

### QSAR – stratified sampling and bootstrap

In the first stages of the investigation, the initial dataset (38 compounds) was split into a training set and a test set using conventional QSAR methods, and attempts were made to validate a number of model fits using cross-validation techniques. The cross validation methods were not successful with this dataset. Although the dataset is of a reasonable size, splitting the dataset into a training and test set resulted in a test set only containing 6 compounds. Due to the parabolic relationship between log(1/EC_50_) and log *P* and high leverage of the few molecules with high or low log *P*, the selection of the test set had an extremely large influence on the validation statistics obtained. It is apparent that if the training set were to miss out even a few of the high and low log *P* molecules then the most reasonable fit would simply be a line almost independent of log *P*.

To cope with the leverage of the high and low log *P* molecules, a stratified test set selection method was employed, ensuring that compounds were selected for the low, mid and high log *P* ranges. However, the size of the dataset and the relatively few molecules in the strata does not allow for much flexibility in the selection. To minimise test set selection bias and maximize the information from all the molecules in the dataset, a bootstrap method was selected as a suitable method for validation of the model fits. Using the bootstrap package, boot, in *R*,^42^,^43^ the data were sampled from the full dataset and the statistics calculated, using a resampling of the dataset 999 times. Comparing the confidence intervals for the bootstrap fit and the linear least squares prediction highlights the reasonable robustness of the fits.

### QSAR models

The first avenue that was explored was fitting the whole dataset to one model. The full descriptor set was examined in JMP,^44^ and using the stepwise fit a ‘fit all models’ was run, modelling the log(1/EC_50_) against the set of descriptors with a maximum of three parameters for the model (four parameters generated too many models for the available computing power, four parameter models were generated with a subset of descriptors). The modelling considered ALOGPs and ALOGP-sq as lipophilicity descriptors. As described earlier, the ALOGPs descriptor was identified as the best log *P* descriptor through correlation with retention times (RT) (for full correlations see ESI† (log *P*_RT_correlations.pdf)).

The simple parabolic two parameter model (ALOGPs, ALOGP-sq) generates the following eqn (1) with an *R*^2^ value of 0.629:

1 Log(1/EC_50_) = –0.579 + 1.203ALOGPs – 0.133ALOGPs-sq

Increasing the number of parameters to three increased the *R*^2^ value to approximately 0.79 for the top models. All the top 20 models have an *R*^2^ value above 0.74. Summary information about the 10 best three-parameter models to the whole dataset is shown in Table 2, ranked by *R*^2^ values (additional models can be seen in ESI†).

**Table 2:** Best fitted 3 and 4 parameter models, ranked by *R*^2^ values. 4 parameter models are fitted with a small subset

No. des.	Descriptors				*R* ^2^
3	ALOGPs	ALOGPs-sq	Mv	—	0.7901
3	ALOGPs	ALOGPs-sq	J3D	—	0.7892
3	ALOGPs	ALOGPs-sq	Mp	—	0.7836
3	ALOGPs	ALOGPs-sq	nH	—	0.7822
3	ALOGPs	ALOGPs-sq	AMW	—	0.7768
3	ALOGPs	ALOGPs-sq	J	—	0.7680
3	ALOGPs	ALOGPs-sq	E3u	—	0.7672
3	ALOGPs	ALOGPs-sq	ARR	—	0.7654
3	ALOGPs	ALOGPs-sq	Density (g cm^–3^)	—	0.7615
3	ALOGPs	ALOGPs-sq	Surface tension (dyne cm^–1^)	—	0.7571
4	ALOGPs	ALOGPs-sq	nCIC	J3D	0.8160
4	ALOGPs	ALOGPs-sq	nH	J	0.8152
4	ALOGPs	ALOGPs-sq	AMW	J	0.8151
4	ALOGPs	ALOGPs-sq	AMW	J3D	0.8141
4	ALOGPs	ALOGPs-sq	J3D	Ui	0.8140
4	ALOGPs	ALOGPs-sq	Density (g cm^–3^)	J3D	0.8138
4	ALOGPs	ALOGPs-sq	Density (g cm^–3^)	J	0.8121
4	ALOGPs	ALOGPs-sq	Parachor (cm^3^)	nH	0.8099
4	ALOGPs	ALOGPs-sq	Molar refractivity (cm^3^)	nH	0.8085
4	ALOGPs	ALOGPs-sq	Polarizability (cm^3^)	nH	0.8084

Following the ‘fit all models’ fit, confidence intervals were obtained for a selected number of models from the least-squares analysis. These models were then also run through a bootstrap method in *R* to obtain confidence intervals using a sampling method. Due to the distribution of the data still being heavily biased towards the middle of the ALOGPs range, we utilised a stratified selection within the bootstrap function to ensure that a selection of points from the lower and upper regions were always included.

Confidence intervals obtained from the bootstrap function were well aligned with the confidence intervals obtained from the linear fit (Table 3) (see ESI† for additional details). This suggests that the fits are quite robust. The most variation comes in the coefficient for the intercept with a much narrower range in the ALOGPs and ALOGPs-sq coefficients. However, plotting actual *vs.* predicted for the models gives a fairly similar appearance for all of the selection of ten models (see ESI† for details).

**Table 3:** Coefficients and confidence intervals for the best two, three and four parameter models

Coefficients	Model parameters	ALOGPs ALOGPs-sq	ALOGPs ALOGPs-sq Mv	ALOGPs ALOGPs-sq nCIC J3D
	*R* ^2^	0.6292	0.7901	0.816
	Intercept	–0.579	3.362	–5.105
Linear fit	2.5% C.I.	–1.165	1.838	–7.579
	97.5% C.I.	0.008	4.887	–2.632
Bootstrap	2.5% C.I.	–1.108	2.159	–7.681
	97.5% C.I.	–0.086	4.419	–2.694
	ALOGPs	1.203	1.372	1.284
Linear fit	2.5% C.I.	0.903	1.135	1.056
	97.5% C.I.	1.504	1.610	1.511
Bootstrap	2.5% C.I.	0.904	1.126	1.087
	97.5% C.I.	1.470	1.579	1.493
	ALOGPs-sq	–0.133	–0.158	–0.146
Linear fit	2.5% C.I.	–0.168	–0.186	–0.172
	97.5% C.I.	–0.098	–0.129	–0.120
Bootstrap	2.5% C.I.	–0.166	–0.190	–0.173
	97.5% C.I.	–0.093	–0.123	–0.116
	3rd parameter		–6.616	0.411
Linear fit	2.5% C.I.		–9.063	0.057
	97.5% C.I.		–4.168	0.764
Bootstrap	2.5% C.I.		–8.432	0.064
	97.5% C.I.		–4.473	0.796
	4th parameter			1.587
Linear fit	2.5% C.I.			0.808
	97.5% C.I.			2.367
Bootstrap	2.5% C.I.			0.796
	97.5% C.I.			2.330

As shown by the models described in Table 2, there were a large number of calculated descriptors that seemed to offer potentially useful additional descriptive power to the fits, but without any clear advantage of one over the others (apart from the clear importance of log *P*). This suggested that principle component analysis and partial least squares analysis might be useful. However, this led to insignificant improvements in the models, and made the contributions of the terms in the models less clear. Therefore, we sought an alternative classification approach along the lines of partial decisions trees by modelling subsets of the compounds based on the structural features of the molecules.

### Structural classification

**Fig. 3:**
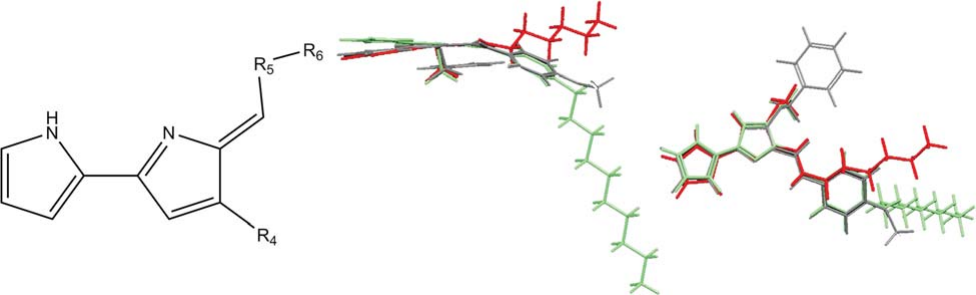
Backbone structure of tambjamines (left). Overlay of compounds side on and face on (green – compound **7**, red – compound **25**, grey – compound **37**) highlighting the similarities and differences of the structural subgroups (right).

The compounds in this series share a bipyrrole core structure, and the rest of the structure can be categorised by three variations on backbone structure (see Fig. 3). The R_4_ position on the heterocycle (ring-substituent) is either occupied by an OMe group or by an OBn group, the R_5_ position (enamine-substituent) is either an NH group or a NH–Ph moiety (with two exceptions: compound **19** is NH–CH_2_–Ph and compound **38** is NH–py), the R_6_ substituent (R-group) is quite varied but can be grouped into the type of substituent *e.g.* alkyl, halogen, *etc.* The presence or absence of a structural feature is a key aspect which could have an effect on the activity of a molecule. Due to this we looked into separating the set of molecules into groups by the structural substituents.

**Fig. 4:**
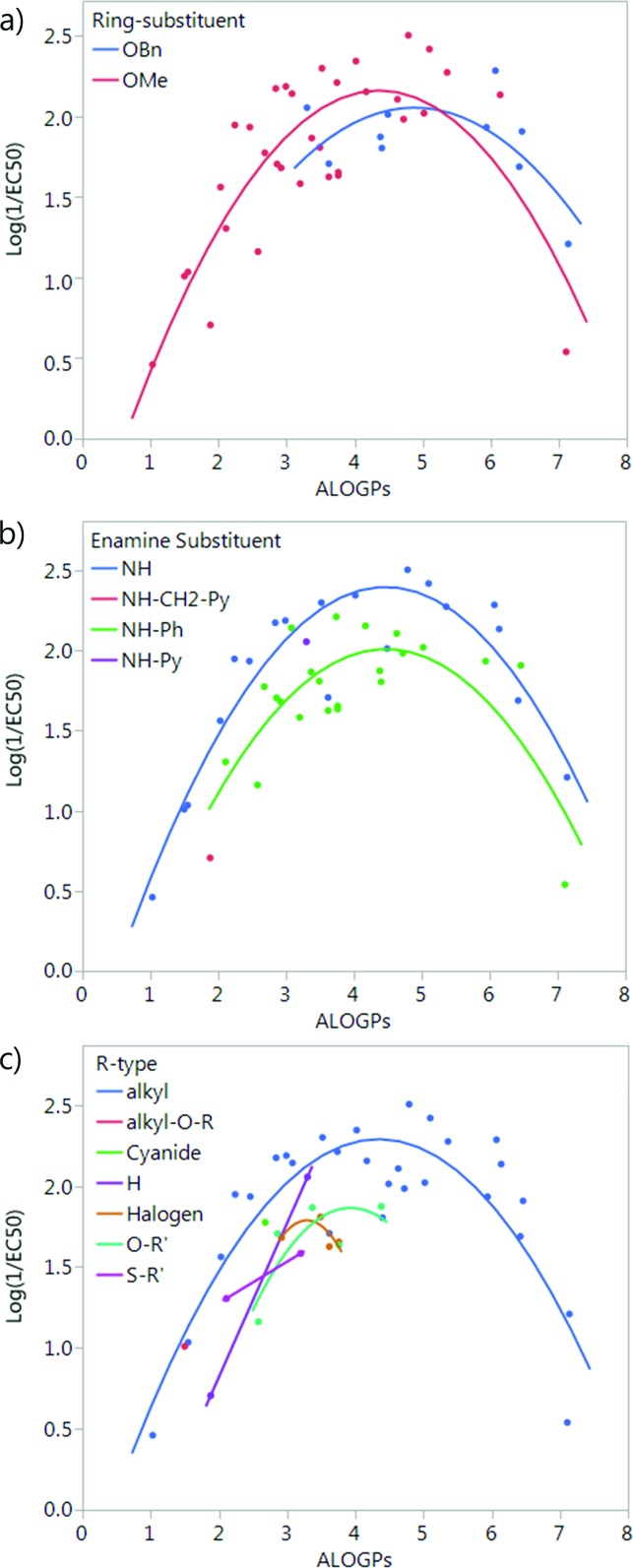
Plot of log(1/EC_50_) *vs.* ALOGPs splitting the dataset by different substituents: (a) ring-substituent (R_4_), (b) enamine-substituent (R_5_), (c) R-type (R_6_), see Fig. 3.

Splitting by ring-substituent R_4_ gives two groups: thirty-three compounds with a methoxy group and ten compounds with a –OBn substituent (Fig. 4a). Splitting considering R_5_ group gives two main groups and two points that do not fit into either the NH or NH–Ph classification. The NH group has nineteen compounds and the NH–Ph group has twenty-two compounds (Fig. 4b). Splitting by the R_6_ group is fairly difficult as there are a variety of different substituents. The most populated group is that in which R_6_ is an alkyl group, with twenty-eight compounds. The remaining fifteen compounds fit into six other groups (Fig. 4c).

**Fig. 5:**
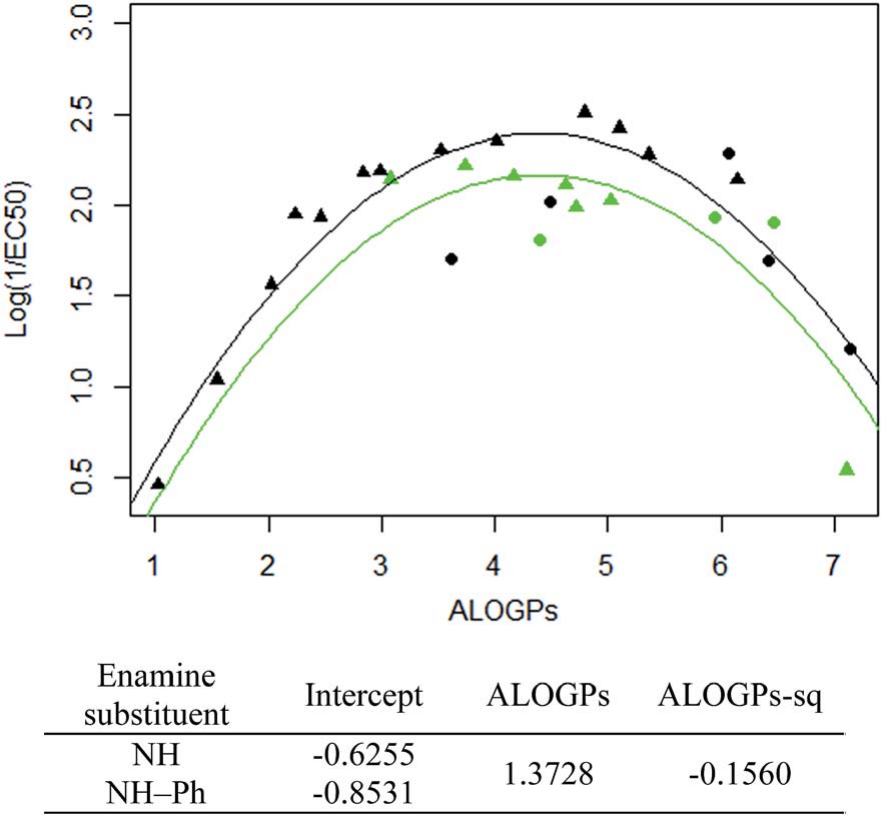
lmer fit and model for the alkyl R type for both OMe and OBn ring substituents (coefficients for ALOGPs and ALOGPs-sq are fitted using all datapoints). Points coloured by enamine-substituent: black – NH, green – NH–Ph. Shape by ring-substituent: circle – OBn, triangle – OMe.

The subset with the most interesting grouping involves the split by enamine-substituent R_5_ (Fig. 4b). From plotting log(1/EC_50_) against ALOGPs (assuming a parabolic relationship) we have two sets of data where the peak log(1/EC_50_) values appears to change between the two sets. However the optimum log *P* value appears to be similar for the two sets. The R-type plot shows a nice parabolic relationship for the R_6_ alkyl R-type, however the other groups are not populated well enough to show a proper correlation. The reason for this is that in the NH group set the main substituent that is possible is an alkyl chain. On the other hand, with the phenyl ring in NH–Ph there is the opportunity to substitute a wider variety of R-types. Since there is only a substitution at the *para* position it limits the number of compounds that will have the same R-type substituent. Due to this we choose to take only those compounds with an alkyl substituent and carry out modelling of the subset using the lme4 package,^45^,^46^ in *R*. This package allows us to use an entire dataset to fit the curve of the parabola, whilst allowing the subset of data to adjust the positioning of the curve by changing the intercept. A linear mixed effect model (lmer) was run for the subset of the compounds containing an alkyl R-type, modelling the dataset to the form log(1/EC_50_) = *a* + *b* × ALOGPs + *c* × ALOGP^2^, and further splitting by the substituent R_4_. See Fig. 5.

**Fig. 6:**
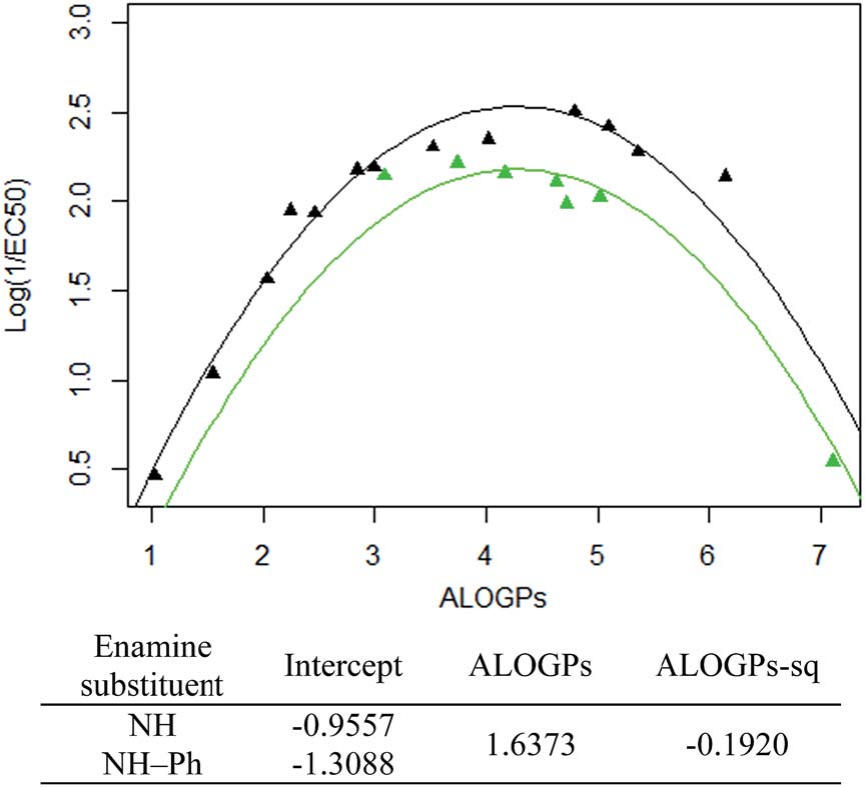
lmer fit and model for the alkyl R type, OMe ring substituent (coefficients for ALOGPs and ALOGPs-sq are fitted using all datapoints). Points coloured by enamine-substituent: black – NH, green – NH–Ph.

Taking only the OMe ring substituted compounds (20 of the 28 alkyl compounds) results in the following lmer model and plot (Fig. 6).

These models show that extending a hydrocarbon tail certainly has the classic parabolic behaviour on log *P* with the optimum value of log *P* (and the curvature) being a property of the membrane (so similar for many of the subsets). The effect of the other substituent (OMe) and (OBn) in changing the maximum value of log(1/EC_50_) is demonstrated but we are less clear what is driving this effect and this will be a subject for further investigation.

**Fig. 7:**
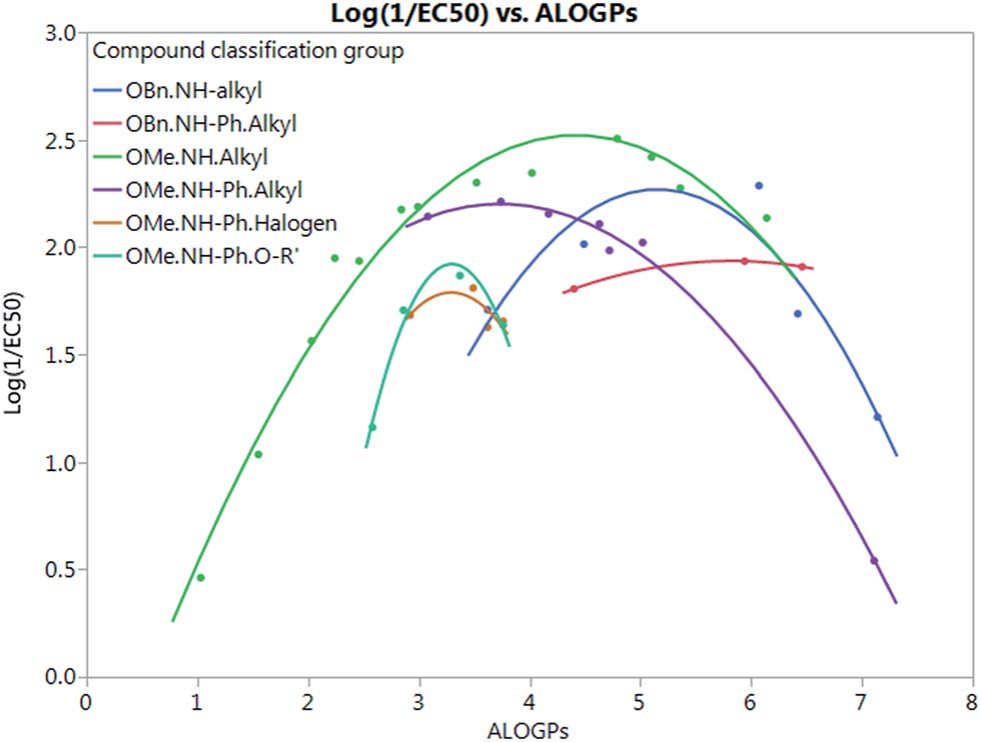
Quadratic fits for all types of compound grouping, excludes groups with less than 3 points, showing behaviour consistent with a parabolic dependence on log *P* but with differing optimum values of log *P* suggesting that other aspects of the mechanism may be more significant in these cases. Groups are classified by the following substituents; R_4_.R_5_.R_6_(R-type).

Fig. 7 shows that by defining several sub-groups of substituents in terms of substituent location and chemical type we are able to demonstrate the parabolic dependence on log *P* and begin to highlight the aspects that are a property of the membrane and those that depend on more specific interactions between the membrane and the tambjamine molecules. The parabolic dependence observed is a property of the membrane. However, each substituent series is shifted in optimal log *P* for transport. This evidence leads us to suggest that whilst for relatively simple substituents in certain locations on the tambjamine core, hydrophobic interactions dominate, for others more specific interactions are present that change the position of the membrane hydrophobicity parabolic envelope. The functions illustrated in Fig. 7 are presented in Table 4.

**Table 4:** Model equations and *R*^2^ values for quadratic fits of compound grouping shown in Fig. 7 modelling for log(1/EC_50_)

Sub group	Equation fit	*R* ^2^
OBn.NH.alkyl	*Y* = –4.783 + 2.737 × ALOGPs – 0.2656 × ALOGPs^2^	0.84
OBn.NH–Ph.alkyl	*Y* = –0.2663 + 0.7575 × ALOGPs – 0.06513 × ALOGPs^2^	0.999
OMe.NH.alkyl	*Y* = –0.8097 + 1.509 × ALOGPs – 0.1707 × ALOGPs^2^	0.97
OMe.NH–Ph.alkyl	*Y* = 0.1699 + 1.088 × ALOGPs – 0.1456 × ALOGPs^2^	0.999
OMe.NH–Ph.halogen	*Y* = –6.332 + 4.936 × ALOGPs – 0.7501 × ALOGPs^2^	0.48
OMe.NH–Ph.O–R′	*Y* = –13.52 + 9.364 × ALOGPs – 1.42 × ALOGPs^2^	0.98

## Conclusions

This study demonstrates the generality of lipophilicity as a crucial parameter governing the transmembrane transport activity of synthetic anionophores. Series of structurally similar compounds containing a common hydrogen bonding motif and a variety of substitution patterns can be grouped in subsets according to structural parameters. In general there is a parabolic dependence between log(1/EC_50_) and log *P* which is a property of the membrane. By defining subgroups of substituents and splitting the data, optimum log *P* values for each sub-group were obtained. This suggests that for different sub-groups of compounds specific interactions are taking place that change the optimum log *P* value. We have thus gained significant insight into how substitution affects the anion transport properties of this important class receptor.

## Supplementary Material

Supplementary information

Supplementary information

Supplementary information

Supplementary information
