# Supplementary material for: QSAR analysis of substituent effects on tambjamine anion transporters
Source: Chem Sci. 2015 Dec 8;7(2):1600–8. doi: 10.1039/c5sc03932k (PMC5964965; doi:10.1039/c5sc03932k)
Supplement: Supplementary file 1 [file SC-007-C5SC03932K-s001.pdf]

Electronic Supplementary Material (ESI) for Chemical Science.
This journal is © The Royal Society of Chemistry 2015

Supporting Information

QSAR analysis of substituent effects on tambjamine anion transporters

Nicola J. Knight,[a] Elsa Hernando,[b] Cally J. E. Haynes,[a] Nathalie Busschaert,[a] Harriet J. Clarke,[a] Koji Takimoto,[c] María García-Valverde,[b] Jeremy G. Frey,[a] Roberto Quesada[b] and Philip A. Gale[a]

[a] *Chemistry, University of Southampton, Southampton, SO17 1BJ, UK. E-mail: philip.gale@soton.ac.uk*

[b] *Departamento de Química, Facultad de Ciencias, Universidad de Burgos,09001 Burgos, Spain. E-mail: rquesada@ubu.es*

[c] *Organic and Polymeric Materials Tokyo Institute of Technology 2-12-1 O-okayama, Tokyo 152-8552, JAPAN*

1. SYNTHESIS AND CHARACTERIZATION S1
1.1. Overview of compounds S1
1.2. Experimental procedures S1
1.3. Characterization data S7
2. ANION TRANSPORT STUDIES S46
2.1. Preparation of vesicles S46
2.2. Chloride/nitrate transport assays S46
2.3. Initial rate of chloride efflux (k_{ini}) determination S47
2.4. Hill plot analyses. EC_{50} determination S68
2.5. K_{ini} EC_{50} correlation for EC_{50} Prediction S91
2.6. Overview of anion transport results S92
3. QSAR ANALYSIS S93
3.1. Fit all Models – 3 and 4 parameters S93
3.2. Descriptor definitions S94
3.3. Model fits S95
3.4. Model fit plots S96
3.5. R Code from linear fits S97
3.6. R Code from mixed effect fits S115

1. SYNTHESIS AND CHARACTERIZATION

1.1. Overview of compounds

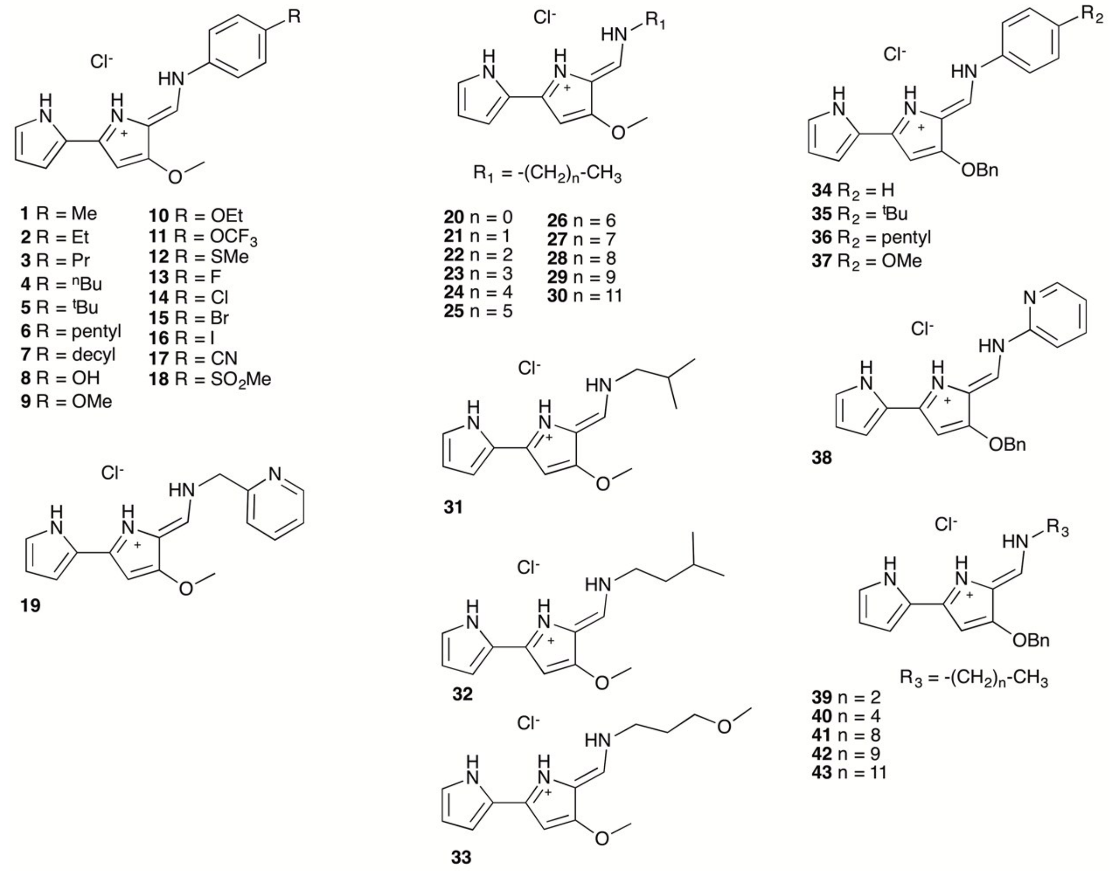

Chart S1. Overview of tambjamine derivatives included in this study.

1.2. Experimental procedures

General. NMR spectra were recorded on Varian Mercury-300 MHz and Varian Unity-400 MHz spectrometers. Chemical shifts (δ) are reported in parts per million (ppm) and calibrated to the residual solvent peak in $CDCl_3$ and DMSO-d_6. High resolution mass spectra (HRMS) were recorded on a MicromassAutospec S-2 spectrometer using EI at 70 eV. Commercial reagents were used as provided by the supplier. 4-methoxy-1H,1'H-[2,2'-bipyrrole]-5-carbaldehyde and 4-(benzyloxy)-1H,1'H-[2,2'-bipyrrole]-5-carbaldehyde[1] were prepared as described in the bibliography. Compounds **5**, **9**, **34**, **35**, **37**, **38**[2] and **20**, **21**, **22**, **23**, **24**, **25**, **26**, **27**, **28**, **29**, **30**, **31**, **32**, **39**, **40, 42**[3] were previously reported by us.

[1] a) K. Dairi, S. Tripathy, G. Attardo and J.–F. Lavallee, *Tetrahedron Lett*., 2006, **47**, 2605–2606; b) P. Iglesias Hernández, D. Moreno, A. Araujo Javier, T. Torroba, R. Pérez-Tomás, R. Quesada, *Chem. Commun.*, 2012, **48**, 1556-1558

[2] E. Hernando, V. Soto-Cerrato, S. Cortés-Arroyo, R. Pérez-Tomás and R. Quesada, *Org. Biomol. Chem.*, 2014, **12**, 1771–1778

[3] V. Saggiomo, S. Otto, I. Marques, V. Félix, T. Torroba and R. Quesada, *Chem. Commun.*, 2012, **48**, 5274–5276

Compounds **1–43** were synthesized using modifications of the previously reported method.[4] 4-methoxy-1H,1'H-[2,2'-bipyrrole]-5-carbaldehyde (190 mg, 1 mmol) or 4-(benzyloxy)-1H,1'H-[2,2'-bipyrrole]-5-carbaldehyde (266 mg, 1 mmol) and the corresponding amine (1.6–8 mmol, 1.6–8 mmol equivalents) were dissolved in 10 ml chloroform (or 1,2-dichloroethane; compounds **11**, **13**, **14**, **15**, **16**, **17**, **18**, **30**, **31** and **32**), 40 µL of acetic acid were added. The mixture was stirred at 60 ºC until TLC showed disappearance of the staring material. The reaction mixture was diluted with 40 mL of dichloromethane and washed with HCl 1M (3 × 25 mL). The organic fraction was dried over Na_2SO_4 and the solvent evaporated to yield **1**–**43** as yellow–orange solids in good to excellent yields.

4'-methoxy-5'-((p-tolylamino)methylene)-1H,5'H-[2,2'-bipyrrol]-1'-ium chloride (1). Yield: 100%; ^{1}H NMR (300 MHz, $CDCl_3$): δ = 13.86 (s, 1H), 11.17 (d, *J* = 14.5 Hz, 1H), 10.67 (s, 1H), 7.75 (d, *J* = 14.7 Hz, 1H), 7.31–7.10 (m, 5H), 6.80 (s, 1H), 6.30 (d, *J* = 3.7 Hz, 1H), 5.98 (d, *J* = 2.1 Hz, 1H), 3.96 (s, 3H, OCH_3), 2.33 (s, 3H); ^{13}C NMR (75 MHz, $CDCl_3$): δ = 165.09 (C), 144.12 (C), 136.18 (C), 136.04 (C), 130.53 (2CH), 130.31 (CH), 125.34 (CH), 122.64 (C), 117.19 (2CH), 114.77 (CH), 113.29 (C), 111.39 (CH), 92.01 (CH), 58.85 (OCH_3), 21.09 (CH_3); HRMS (EI) m/z $[M]^+$ calcd for $[C_{17}H_{17}N_3O]$ 279.1372; found: 279.1376.

5'-(((4-ethylphenyl)amino)methylene)-4'-methoxy-1H,5'H-[2,2'-bipyrrol]-1'-ium chloride (2). Yield: 95%; ^{1}H NMR (300 MHz, $CDCl_3$): δ = 13.90 (s, 1H), 11.20 (d, *J* = 14.3 Hz, 1H), 10.69 (s, 1H), 7.76 (d, *J* = 14.7 Hz, 1H), 7.41–7.10 (m, 5H), 6.81 (s, 1H), 6.32 (s, 1H), 5.99 (s, 1H), 3.97 (s, 3H, OCH_3), 2.64 (app q, 2H), 1.22 (t, *J* = 7.6 Hz, 3H); ^{13}C NMR (75 MHz, $CDCl_3$): δ = 165.07 (C), 144.05 (C), 142.38 (C), 136.32 (C), 130.29 (CH), 129.31 (2CH), 125.21 (CH), 122.63 (C), 117.26 (2CH), 114.73 (CH), 113.26 (C), 111.35 (CH), 92.04 (CH), 58.84 (OCH_3), 28.42 (CH_2), 15.62 (CH_3); HRMS (EI) m/z $[M]^+$ calcd for $[C_{18}H_{19}N_3O]$ 293.1528; found: 293.1521.

4'-methoxy-5'-(((4-propylphenyl)amino)methylene)-1H,5'H-[2,2'-bipyrrol]-1'-ium chloride (3). Yield: 92%; ^{1}H NMR (300 MHz, $CDCl_3$): δ = 13.55 (s, 1H), 11.08 (d, *J* = 14.6 Hz, 1H), 10.61 (s, 1H), 7.62 (d, *J* = 14.7 Hz, 1H), 7.26–7.06 (m, 4H), 7.01 (d, *J* = 1.2 Hz, 1H), 6.75 (s, 1H), 6.21 (d, *J* = 3.7 Hz, 1H), 5.95 (d, *J* = 2.1 Hz, 1H), 3.86 (s, 3H, OCH_3), 2.50 (t, *J* = 7.1 Hz, 2H), 1.65–1.49 (m, 2H), 0.89 (t, *J* = 7.3 Hz, 3H); ^{13}C NMR (75 MHz, $CDCl_3$): δ = 164.97 (C), 143.87 (C), 140.65 (C), 136.20 (C), 130.05 (CH), 129.75 (2CH), 124.93 (CH), 122.51 (C), 117.00 (2CH), 114.62 (CH), 113.08 (C), 111.24 (CH), 92.06 (CH), 58.78 (OCH_3), 37.41 (CH_2), 24.49 (CH_2), 13.75 (CH_3); HRMS (EI) m/z $[M]^+$ calcd for $[C_{19}H_{21}N_3O]$ 307.1685; found: 307.1690.

5'-(((4-butylphenyl)amino)methylene)-4'-methoxy-1H,5'H-[2,2'-bipyrrol]-1'-ium chloride (4). Yield: 97%; ^{1}H NMR (300 MHz, $CDCl_3$): δ = 13.59 (s, 1H), 11.08 (d, *J* = 14.6 Hz, 1H), 10.62 (s, 1H), 7.64 (d, *J* = 14.7 Hz, 1H), 7.26–7.09 (m, 4H), 7.06–7.01 (m, 1H), 6.83–6.71 (m, 1H), 6.23 (d, *J* = 3.8 Hz, 1H), 5.95 (d, *J* = 2.2 Hz, 1H), 3.88 (s, 3H, OCH_3), 2.54 (t, *J* = 7.8 Hz, 2H), 1.61–1.46 (m, 2H), 1.37–1.24 (m, 2H), 0.89 (t, *J* = 7.3 Hz, 3H); ^{13}C NMR (75 MHz, $CDCl_3$): δ = 164.99 (C), 143.90 (C), 140.93 (C), 136.18 (C), 130.10 (CH),

[4] D. M. Pinkerton, M. G. Banwell and A. C. Willis, *Org. Lett.*, 2007, **9**, 5127–5130

129.73 (2CH), 125.01 (CH), 122.53 (C), 117.06 (2CH), 114.65 (CH), 113.10 (C), 111.26 (CH), 92.06 (CH), 58.79 (OCH_3), 35.09 (CH_2), 33.57 (CH_2), 22.33 (CH_2), 13.98 (CH_3); HRMS (EI) m/z $[M]^+$ calcd for [$C_{20}H_{23}N_3O$] 321.1841; found: 321.1841.

4'-methoxy-5'-(((4-pentylphenyl)amino)methylene)-1H,5'H-[2,2'-bipyrrol]-1'-ium chloride (6).
Yield: 100%; ^{1}H NMR (300 MHz, $CDCl_3$): δ = 13.58 (s, 1H), 11.08 (d, *J* = 14.6 Hz, 1H), 10.62 (s, 1H), 7.62 (d, *J* = 14.6 Hz, 1H), 7.26–7.05 (m, 4H), 7.01 (s, 1H), 6.75 (s, 1H), 6.21 (d, *J* = 1.6 Hz, 1H), 5.94 (s, 1H), 3.86 (s, 3H, OCH_3), 2.51 (t, *J* = 7.7 Hz, 2H), 1.62–1.47 (m, 2H), 1.30–1.20 (m, 4H), 0.86 (t, *J* = 6.5 Hz, 3H); ^{13}C NMR (75 MHz, $CDCl_3$): δ = 164.96 (C), 143.86 (C), 140.91 (C), 136.17 (C), 130.06 (CH), 129.68 (2CH), 124.90 (CH), 122.53 (C), 117.01 (2CH), 114.59 (CH), 113.08 (C), 111.24 (CH), 92.05 (CH), 58.77 (OCH_3), 35.33 (CH_2), 31.43 (CH_2), 31.07 (CH_2), 22.52 (CH_2), 14.06 (CH_3); HRMS (EI) m/z $[M]^+$ calcd for [$C_{21}H_{25}N_3O$] 335.1998; found: 335.1981.

5'-(((4-decylphenyl)amino)methylene)-4'-methoxy-1H,5'H-[2,2'-bipyrrol]-1'-ium chloride (7). Yield: 95%; ^{1}H NMR (400 MHz, $CDCl_3$): δ = 13.90 (s, 1H), 11.20 (d, *J* = 14.5 Hz, 1H), 10.68 (s, 1H), 7.75 (d, *J* = 14.6 Hz, 1H), 7.32–7.16 (m, 4H), 7.12 (s, 1H), 6.81 (s, 1H), 6.31 (d, *J* = 3.6 Hz, 1H), 5.99 (d, *J* = 2.1 Hz, 1H), 3.97 (s, 3H, OCH_3), 2.58 (t, *J* = 5.7 Hz, 2H), 1.64–1.52 (m, 4H), 1.30–1.24 (m, 12H), 0.87 (t, *J* = 6.7 Hz, 3H); ^{13}C NMR (75 MHz, $CDCl_3$): δ = 165.03 (C), 143.98 (C), 141.09 (C), 136.25 (C), 130.23 (CH), 129.81 (2CH), 125.14 (CH), 122.61 (C), 117.15 (2CH), 114.69 (CH), 113.21 (C), 111.32 (CH), 92.03 (CH), 58.82 (OCH_3), 35.47 (CH_2), 31.99 (CH_2), 31.51 (CH_2), 29.70 ($2CH_2$), 29.57 (CH_2), 29.42 (CH_2), 29.33 (CH_2), 22.78 (CH_2), 14.23 (CH_3); HRMS (EI) m/z $[M]^+$ calcd for [$C_{26}H_{35}N_3O$] 405.2780; found: 405.2776.

5'-(((4-hydroxyphenyl)amino)methylene)-4'-methoxy-1H,5'H-[2,2'-bipyrrol]-1'-ium chloride (8). Yield: 38%; ^{1}H NMR (300 MHz, DMSO-d_6): δ = 13.09 (s, 1H), 12.52 (d, *J* = 14.1 Hz, 1H), 11.96 (s, 1H), 9.74 (s, 1H), 8.17 (d, *J* = 13.9 Hz, 1H), 7.46 (d, *J* = 8.4 Hz, 2H), 7.19 (s, 2H, OH), 6.83 (d, *J* = 8.4 Hz, 2H), 6.56 (s, 1H), 6.31 (s, 1H), 3.98 (s, 3H, OCH_3); ^{13}C NMR (75 MHz, DMSO-d_6): δ = 164.36 (C), 155.85 (C), 142.16 (C), 132.53 (CH), 130.45 (C), 124.39 (CH), 122.24 (C), 119.09 (2CH), 116.20 (2CH), 111.95 (CH), 111.89 (C), 110.81 (CH), 92.19 (CH), 58.75 (OCH_3); HRMS (EI) m/z $[M]^+$ calcd for [$C_{16}H_{15}N_3O_2$] 281.1164; found: 281.1166.

5'-(((4-ethoxyphenyl)amino)methylene)-4'-methoxy-1H,5'H-[2,2'-bipyrrol]-1'-ium chloride (10). Yield: 92%; ^{1}H NMR (300 MHz, $CDCl_3$): δ = 13.75 (s, 1H), 11.23 (d, *J* = 14.7 Hz, 1H), 10.61 (s, 1H), 7.69 (d, *J* = 14.8 Hz, 1H), 7.31 (d, *J* = 9.0 Hz, 2H), 7.13–7.07 (m, 1H), 6.88 (d, *J* = 9.0 Hz, 2H), 6.82–6.75 (m, 1H), 6.38–6.22 (m, 1H), 5.97 (d, *J* = 2.2 Hz, 1H), 4.01 (q, *J* = 7.0 Hz, 2H), 3.95 (s, 3H, OCH_3), 1.40 (t, *J* = 7.0 Hz, 3H); ^{13}C NMR (75 MHz, $CDCl_3$): δ = 164.75 (C), 157.40 (C), 143.54 (C), 131.83 (C), 130.59 (CH), 125.04 (CH), 122.71 (C), 118.73 (2CH), 115.71 (2CH), 114.40 (CH), 112.99 (C), 111.29 (CH), 91.91 (CH), 63.98 (CH_2), 58.79 (OCH_3), 14.88 (CH_3); HRMS (EI) m/z $[M]^+$ calcd for [$C_{18}H_{19}N_3O_2$] 309.1477; found: 309.1483.

4'-methoxy-5'-(((4-(trifluoromethoxy)phenyl)amino)methylene)-1H,5'H-[2,2'-bipyrrol]-1'-ium chloride (11). Yield: 95%; ^{1}H NMR (300 MHz, $CDCl_3$): δ = 13.68 (s, 1H), 11.18 (d, *J* = 14.1 Hz, 1H), 10.64 (s, 1H), 7.66 (d, *J* = 14.3 Hz, 1H), 7.36 (d, *J* = 8.8 Hz, 2H), 7.17 (d, *J* = 8.5 Hz, 2H), 7.10 (s, 1H), 6.82 (s, 1H), 6.29 (s, 1H), 5.94 (s, 1H), 3.89 (s, 3H, OCH_3); ^{13}C NMR (75 MHz, $CDCl_3$): δ = 165.71 (C), 146.51 (q, *J* = 1.7 Hz, C), 145.27 (C), 137.32(C), 129.65 (CH), 125.97 (CH), 122.70 (2CH), 122.43 (C), 120.5 (q, *J* = 256.0 Hz, CF_3) 118.30 (2CH), 115.69 (CH), 114.06 (C), 111.72 (CH), 92.34 (CH), 58.94 (OCH_3); HRMS (EI) m/z $[M]^+$ calcd for $[C_{17}H_{14}F_3N_3O_2]$ 349.1038; found: 349.1039.

4'-methoxy-5'-(((4-(methylthio)phenyl)amino)methylene)-1H,5'H-[2,2'-bipyrrol]-1'-ium chloride (12). Yield: 87%; ^{1}H NMR (300 MHz, DMSO-d_6): δ = 13.16 (s, 1H), 12.52 (d, *J* = 14.1 Hz, 1H), 12.06 (s, 1H), 8.27 (d, *J* = 14.2 Hz, 1H), 7.58 (d, *J* = 8.4 Hz, 2H), 7.34 (d, *J* = 8.4 Hz, 2H), 7.25 (s, 2H), 6.60 (s, 1H), 6.35 (s, 1H), 4.01 (s, 3H, OCH_3), 2.50 (s, 3H); ^{13}C NMR (75 MHz, DMSO-d_6): δ = 165.21 (C), 143.58 (C), 135.94 (C), 135.40 (C), 131.90 (CH), 127.29 (2CH), 125.10 (CH), 122.15 (2C), 118.02 (2CH), 112.79 (CH), 111.14 (CH), 92.54 (CH), 58.92 (OCH_3), 15.06 (CH_3); HRMS (EI) m/z $[M]^+$ calcd for $[C_{17}H_{17}N_3OS]$ 311.1092; found: 311.1098.

5'-(((4-fluorophenyl)amino)methylene)-4'-methoxy-1H,5'H-[2,2'-bipyrrol]-1'-ium chloride (13). Yield: 82%; ^{1}H NMR (300 MHz, DMSO-d_6): δ = 13.19 (s, 1H), 12.58 (d, *J* = 14.1 Hz, 1H), 12.05 (s, 1H), 8.25 (d, *J* = 14.0 Hz, 1H), 7.72–7.59 (m, 2H), 7.38–7.16 (m, 4H), 6.59 (s, 1H), 6.34 (s, 1H), 4.00 (s, 3H, OCH_3); ^{13}C NMR (75 MHz, DMSO-d_6): δ = 165.53 (C), 159.78 (C, d, *J* = 241.5 Hz), 143.87 (C), 135.30 (C, d, *J* = 2.3 Hz), 132.58 (CH), 125.21 (CH), 122.12 (C), 119.35 (2CH, d, *J* = 8.3 Hz), 116.60 (2CH, d, *J* = 23.1 Hz), 112.92 (CH), 111.83 (C), 111.20 (CH), 92.61 (CH), 58.99 (OCH_3); HRMS (EI) m/z [M]+ calcd for $[C_{16}H_{14}FN_3O]$ 283.1121; found: 283.1130.

5'-(((4-chlorophenyl)amino)methylene)-4'-methoxy-1H,5'H-[2,2'-bipyrrol]-1'-ium chloride (14). Yield: 74%; ^{1}H NMR (300 MHz, DMSO-d_6): δ = 13.21 (s, 1H), 12.55 (d, *J* = 13.8 Hz, 1H), 12.08 (s, 1H), 8.25 (d, *J* = 13.5 Hz, 1H), 7.63 (d, *J* = 8.8 Hz, 2H), 7.48 (d, *J* = 8.8 Hz, 2H), 7.26 (s, 2H), 6.59 (s, 1H), 6.35 (s, 1H), 4.00 (s, 3H, OCH_3); ^{13}C NMR (75 MHz, DMSO-d_6): δ = 165.66 (C), 144.33 (C), 137.72 (C), 131.85 (CH), 129.58 (2CH), 129.32 (C), 125.41 (CH), 122.03 (C), 118.95 (2CH), 113.18 (CH, C), 111.22 (CH), 92.66 (CH), 58.94 (OCH_3); HRMS (EI) m/z [M]+ calcd for $[C_{16}H_{14}ClN_3O]$ 299.0825; found: 299.0828.

5'-(((4-bromophenyl)amino)methylene)-4'-methoxy-1H,5'H-[2,2'-bipyrrol]-1'-ium chloride (15). Yield: 95%; ^{1}H NMR (300 MHz, DMSO-d_6): δ = 13.06 (s, 1H), 12.37 (d, *J* = 12.1 Hz, 1H), 12.07 (s, 1H), 8.18 (d, *J* = 8.9 Hz, 1H), 7.62–7.48 (m, 4H), 7.23 (s, 2H), 6.56 (s, 1H), 6.34 (s, 1H), 3.97 (s, 3H, OCH_3); ^{13}C NMR (75 MHz, DMSO-d_6): δ = 165.84 (C), 144.55 (C), 138.19 (C), 132.58 (2CH), 131.65 (CH), 125.58 (CH), 122.11 (C), 119.33 (2CH), 117.59 (C), 113.34 (CH, C), 111.39 (CH), 92.80 (CH), 59.09 (OCH_3); HRMS (EI) m/z [M]+ calcd for $[C_{16}H_{14}BrN_3O]$ 343.0320; found: 343.0328.

5'-(((4-iodophenyl)amino)methylene)-4'-methoxy-1H,5'H-[2,2'-bipyrrol]-1'-ium chloride (16). Yield: 72%; ^{1}H NMR (300 MHz, DMSO-d_6): δ = 13.19 (s, 1H), 12.47 (d, *J* = 14.1 Hz, 1H), 12.08 (s, 1H), 8.24 (d, *J* = 13.8 Hz, 1H), 7.76 (d, *J* = 8.7 Hz, 2H), 7.43 (d, *J* = 8.7 Hz, 2H), 7.26 (s, 2H), 6.59 (s, 1H), 6.35-6.33 (m, 1H), 4.00 (s, 3H, OCH_3); ^{13}C NMR (75 MHz, DMSO-d_6): δ = 165.70 (C), 144.42 (C), 138.56 (C), 138.28 (2CH), 131.52 (CH), 125.49 (CH), 122.04 (C), 119.43 (2CH), 113.25 (CH, C), 111.27 (CH), 92.69 (CH), 89.79 (C), 58.97 (OCH_3); HRMS (EI) m/z [M]+ calcd for [$C_{16}H_{14}IN_3O$] 391.0182; found: 391.0179.

5'-(((4-cyanophenyl)amino)methylene)-4'-methoxy-1H,5'H-[2,2'-bipyrrol]-1'-ium chloride (17). Yield: 100%; ^{1}H NMR (300 MHz, $CDCl_3$): δ = 13.99 (s, 1H), 11.34 (d, *J* = 13.7 Hz, 1H), 10.72 (s, 1H), 7.71 (d, *J* = 13.9 Hz, 1H), 7.65 (d, *J* = 8.7 Hz, 2H), 7.46 (d, *J* = 8.8 Hz, 2H), 7.24–7.18 (m, 1H), 6.96–6.89 (m, 1H), 6.43–6.32 (m, 1H), 6.03 (d, *J* = 2.0 Hz, 1H), 4.02 (s, 3H; OCH_3); ^{1}H NMR (300 MHz, DMSO-d_6): δ = 13.28 (s, 1H), 12.56 (d, *J* = 13.7 Hz, 1H), 12.19 (s, 1H), 8.32 (d, *J* = 0.6 Hz, 1H), 7.89 (d, *J* = 8.5 Hz, 2H), 7.77 (d, *J* = 8.4 Hz, 2H), 7.34 (s, 2H), 6.64 (s, 1H), 6.39 (s, 1H), 4.03 (s, 3H; OCH_3); ^{13}C NMR (75 MHz, DMSO-d_6): δ = 166.52 (C), 145.92 (C), 142.69 (C), 133.97 (2CH), 130.70 (CH), 126.39 (CH), 121.86 (C), 118.77 (C), 117.58 (2CH), 114.47 (C), 114.20 (CH), 111.65 (CH), 106.61 (C), 93.09 (CH), 59.13 (OCH_3); HRMS (EI) m/z $[M]^+$ calcd for [$C_{17}H_{14}N_4O$] 290.1168; found: 290.1165.

4'-methoxy-5'-(((4-(methylsulfonyl)phenyl)amino)methylene)-1H,5'H-[2,2'-bipyrrol]-1'-ium chloride (18). Yield: 66%; ^{1}H NMR (300 MHz, DMSO-d_6): δ = 13.32 (s, 1H), 12.60 (d, *J* = 13.7 Hz, 1H), 12.20 (s, 1H), 8.34 (d, *J* = 13.6 Hz, 1H), 7.95 (d, *J* = 8.6 Hz, 2H), 7.82 (d, *J* = 8.6 Hz, 2H), 7.33 (s, 2H), 6.65 (s, 1H), 6.39 (s, 1H), 4.04 (s, 3H, OCH_3), 3.23 (s, 3H); ^{13}C NMR (75 MHz, DMSO-d_6): δ = 166.49 (C), 145.80 (C), 143.04 (C), 136.37 (C), 130.89 (CH), 128.95 (2CH), 126.31 (CH), 121.89 (C), 117.35 (2CH), 114.34 (CH), 114.13 (C), 111.64 (CH), 93.08 (CH), 59.14 (OCH_3), 43.70 (CH_3); HRMS (EI) m/z $[M]^+$ calcd for [$C_{17}H_{17}N_3O_3S$] 343.0991; found: 343.0986.

1-(4'-methoxy-1H,5'H-[2,2'-bipyrrol]-5'-ylidene)-N-(pyridin-2-ylmethyl)methanamine (19). Yield: 19%; ^{1}H NMR (300 MHz, $CDCl_3$): δ = 13.70 (s, 1H), 10.67 (s, 1H), 9.83 (s, 1H), 8.57 (d, *J* = 4.8 Hz, 1H), 7.73 (td, *J* = 7.9, 1.5 Hz, 1H), 7.58–7.45 (m, 2H), 7.30–7.20 (m, 1H), 7.06 (s, 1H), 6.80–6.71 (m, 1H), 6.31–6.24 (m, 1H), 5.95 (s, 1H), 4.73 (s, 2H), 3.92 (s, 3H, OCH_3). ^{13}C NMR (75 MHz, $CDCl_3$): δ = 164.63 (C), 155.37 (C), 149.91 (CH), 143.34 (C), 140.75 (CH), 137.61 (CH), 124.63 (CH), 123.41 (CH), 122.71 (C), 122.51 (CH), 113.86 (CH), 111.79 (C), 111.04 (CH), 91.46 (CH), 58.66 (OCH_3), 55.19 (CH_2). HRMS (EI) m/z $[M]^+$ calcd for [$C_{16}H_{16}N_4O_4$] 280.1324; found: 280.1325.

4'-methoxy-5'-(((3-methoxypropyl)amino)methylene)-1H,5'H-[2,2'-bipyrrol]-1'-ium chloride (33). Yield: 88%; ^{1}H NMR (300 MHz, $CDCl_3$): δ = 13.63 (s, 1H), 10.61 (s, 1H), 9.40 (s, 1H), 7.35 (d, *J* = 14.4 Hz, 1H), 7.05 (td, *J* = 2.7, 1.4 Hz, 1H), 6.76–6.71 (m, 1H), 6.31–6.25 (m, 1H), 5.94 (d, *J* = 1.9 Hz, 1H), 3.92 (s, 3H, OCH_3), 3.61 (app q, 2H), 3.50 (t, *J* = 5.7 Hz, 2H), 3.35 (s, 3H, OCH_3), 1.99 (app quin, 2H). ^{13}C NMR (75 MHz, $CDCl_3$): δ = 164.00 (C), 142.51 (C), 140.79 (CH), 124.23 (CH), 122.77 (C), 113.32 (CH), 110.97 (C), 110.87 (CH), 91.24 (CH), 68.50 (CH_2), 58.83 (OCH_3), 58.60 (OCH_3), 47.77 (CH_2), 30.13 (CH_2). HRMS (EI) m/z $[M]^+$ calcd for [$C_{14}H_{19}N_3O_2$] 261.1417; found: 261.1475.

4'-(benzyloxy)-5'-(((4-pentylphenyl)amino)methylene)-1H,5'H-[2,2'-bipyrrol]-1'-ium chloride (36). Yield: 95%; ^{1}H NMR (300 MHz, $CDCl_3$): δ = 13.90 (s, 1H), 11.26 (d, *J* = 14.7 Hz, 1H), 10.70 (s, 1H), 7.76 (d, *J* = 14.4 Hz, 1H), 7.53–7.35 (m, 5H), 7.33–7.12 (m, 4H), 7.09 (s, 1H), 6.79 (s, 1H), 6.29 (s, 1H), 6.06 (s, 1H), 5.19 (s, 2H), 2.57 (t, *J* = 7.6 Hz, 2H), 1.58 (app quin, 2H), 1.36–1.24 (m, 4H), 0.89 (t, *J* = 6.6 Hz, 3H). ^{13}C NMR (75 MHz, $CDCl_3$): δ = 163.90 (C), 144.04 (C), 141.26 (C), 136.34 (C), 134.91 (C), 130.64 (CH), 129.87 (2CH), 129.08 (CH), 129.00 (2CH), 128.22 (2CH), 125.29 (CH), 122.67 (C), 117.40 (2CH), 114.75 (CH), 113.57 (C), 111.35 (CH), 92.98 (CH), 73.84 (CH_2), 35.47 (CH_2), 31.50 (CH_2), 31.17 (CH_2), 22.62 (CH_2), 14.14 (CH_3). HRMS (EI) m/z $[M]^+$ calcd for $[C_{27}H_{29}N_3O]$ 411.2311; found: 411.2310.

4'-(benzyloxy)-5'-((nonylamino)methylene)-1H,5'H-[2,2'-bipyrrol]-1'-ium chloride (41). Yield: 74%; ^{1}H NMR (300 MHz, $CDCl_3$): δ = 13.44 (s, 1H), 10.48 (s, 1H), 9.37 (d, *J* = 14.9 Hz, 1H), 7.37–7.08 (m, 6H), 6.85 (s, 1H), 6.60 (s, 1H), 6.14–6.07 (m, 1H), 5.89 (d, *J* = 2.0 Hz, 1H), 4.96 (s, 2H), 3.28 (app q, 2H), 1.58 (app quin, 2H), 1.28–1.03 (m, 12H), 0.72 (t, *J* = 6.6 Hz, 3H). ^{13}C NMR (75 MHz, $CDCl_3$): δ = 162.55 (C), 142.11 (C), 140.55 (CH), 135.05 (C), 128.85 (CH), 128.82 (2CH), 128.02 (2CH), 123.96 (CH), 122.72 (C), 113.11 (CH), 111.01 (C), 110.74 (CH), 92.10 (CH), 73.43 (CH_2), 51.04 (CH_2), 31.82 (CH_2), 30.31 (CH_2), 29.40 (CH_2), 29.23 (CH_2), 29.12 (CH_2), 26.53 (CH_2), 22.66 (CH_2), 14.13 (CH_3). HRMS (EI) m/z $[M]^+$ calcd for $[C_{25}H_{33}N_3O]$ 391.2624; found: 391.2530.

4'-(benzyloxy)-5'-((undecylamino)methylene)-1H,5'H-[2,2'-bipyrrol]-1'-ium chloride (43). Yield: 59%; ^{1}H NMR (300 MHz, $CDCl_3$): δ = 13.61 (s, 1H), 10.62 (s, 1H), 9.51 (d, *J* = 14.7 Hz, 1H), 7.43–7.31 (m, 6H), 7.01 (s, 1H), 6.72 (s, 1H), 6.29–6.21 (m, 1H), 6.01 (d, *J* = 1.9 Hz, 1H), 5.11 (s, 2H), 3.43 (app q, 2H), 1.72 (app quin, 2H), 1.41–1.17 (m, 18H), 0.86 (t, *J* = 6.6 Hz, 3H). ^{13}C NMR (75 MHz, $CDCl_3$): δ = 162.60 (C), 142.21 (C), 140.59 (CH), 135.09 (C), 128.93 (CH), 128.89 (2CH), 128.07 (2CH), 124.10 (CH), 122.76 (C), 113.18 (CH), 111.07 (C), 110.79 (CH), 92.11 (CH), 73.49 (CH_2), 51.12 (CH_2), 31.98 (CH_2), 30.38 (CH_2), 29.68 ($2CH_2$), 29.64 (CH_2), 29.51 (CH_2), 29.41 (CH_2), 29.19 (CH_2), 26.60 (CH_2), 22.76 (CH_2), 14.20 (CH_3). HRMS (EI) m/z $[M]^+$ calcd for $[C_{28}H_{39}N_3O]$ 433.3093; found: 433.3112.

1.3. Characterization data

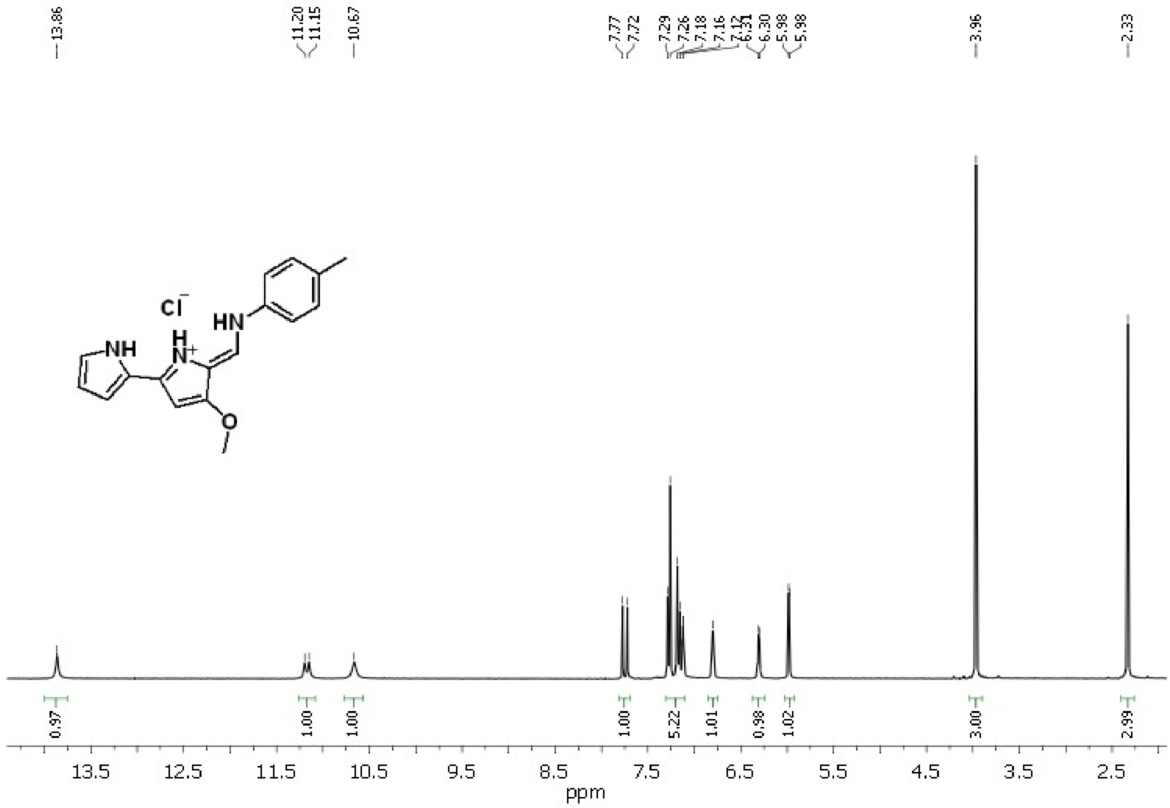

Figure S1. ^{1}H NMR ($CDCl_3$) of compound **1**. HCl.

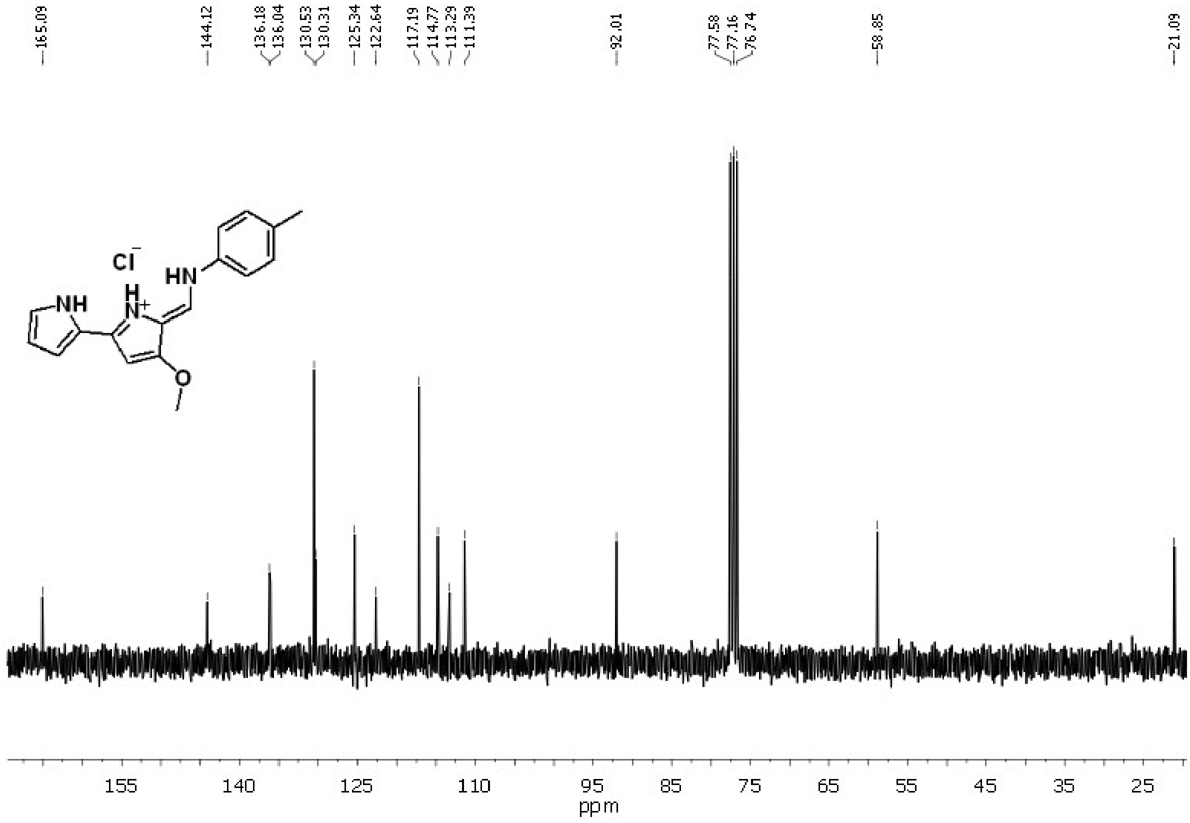

Figure S2. ^{13}C NMR ($CDCl_3$) of compound **1**. HCl.

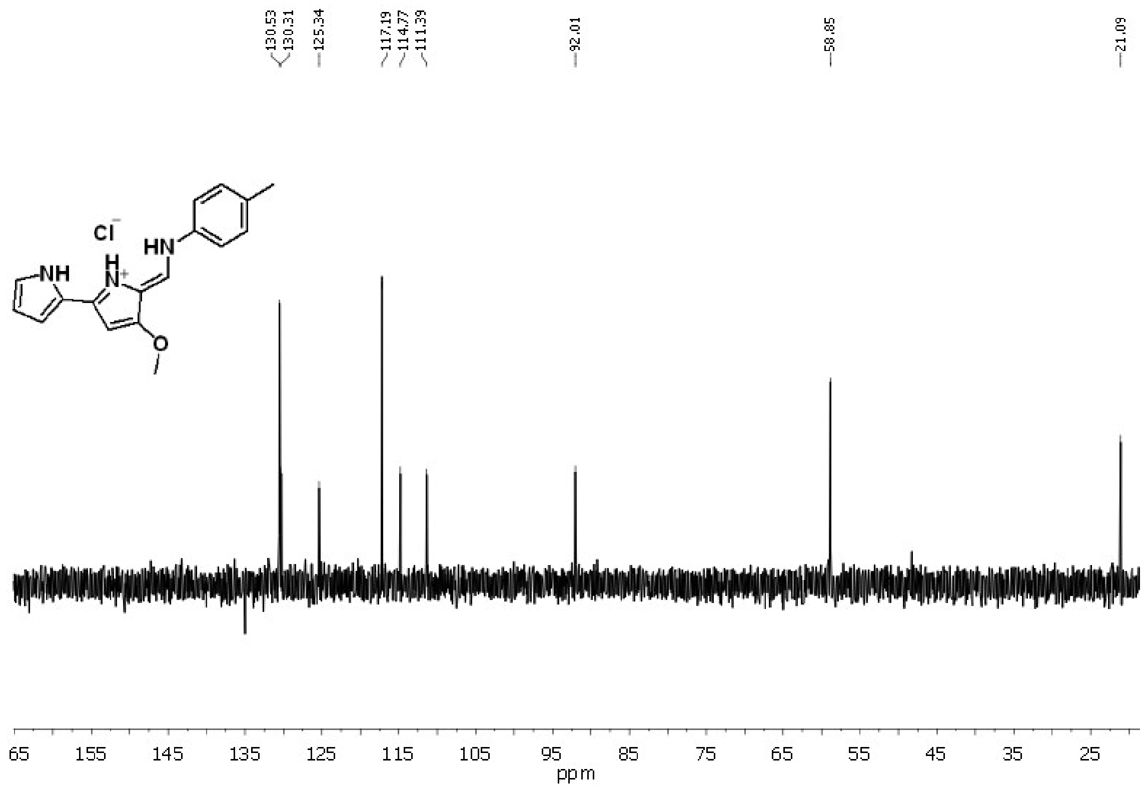

Figure S3. DEPT ^{13}C NMR ($CDCl_3$) of compound **1**. HCl.

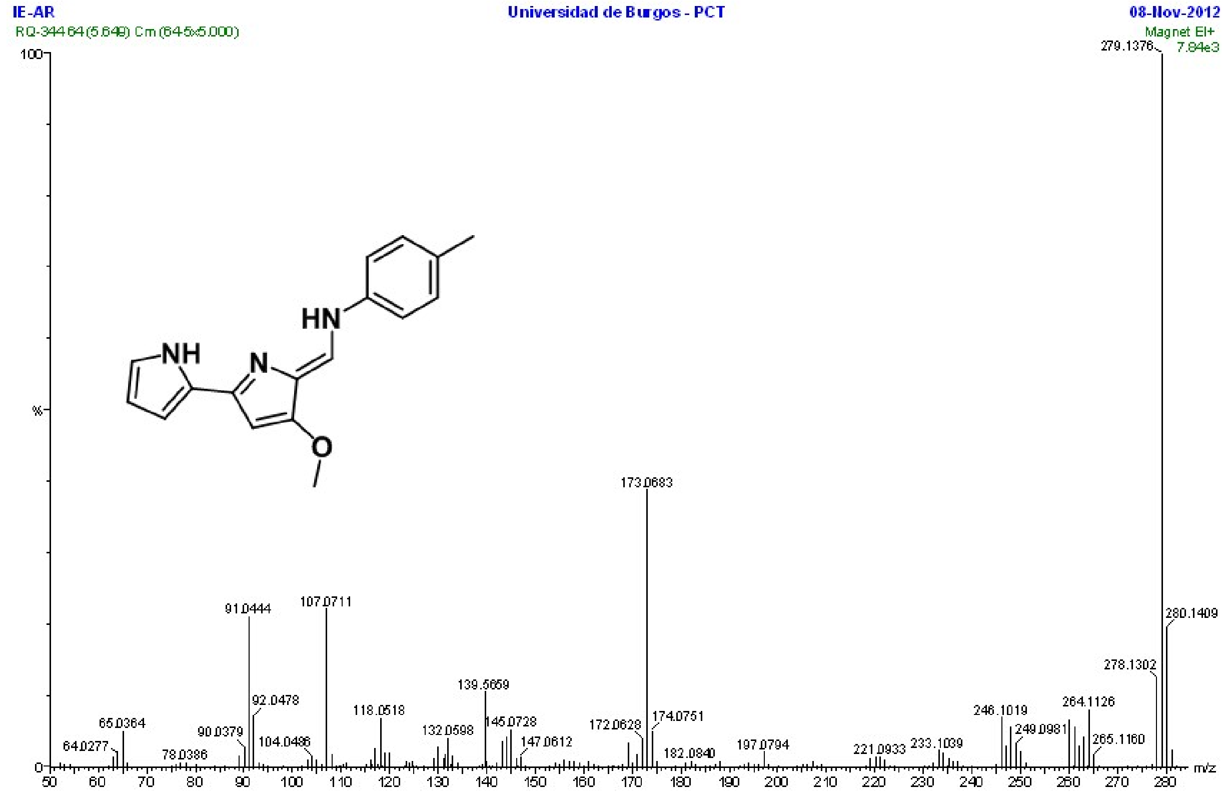

Figure S4. HRMS (EI) of compound **1**.

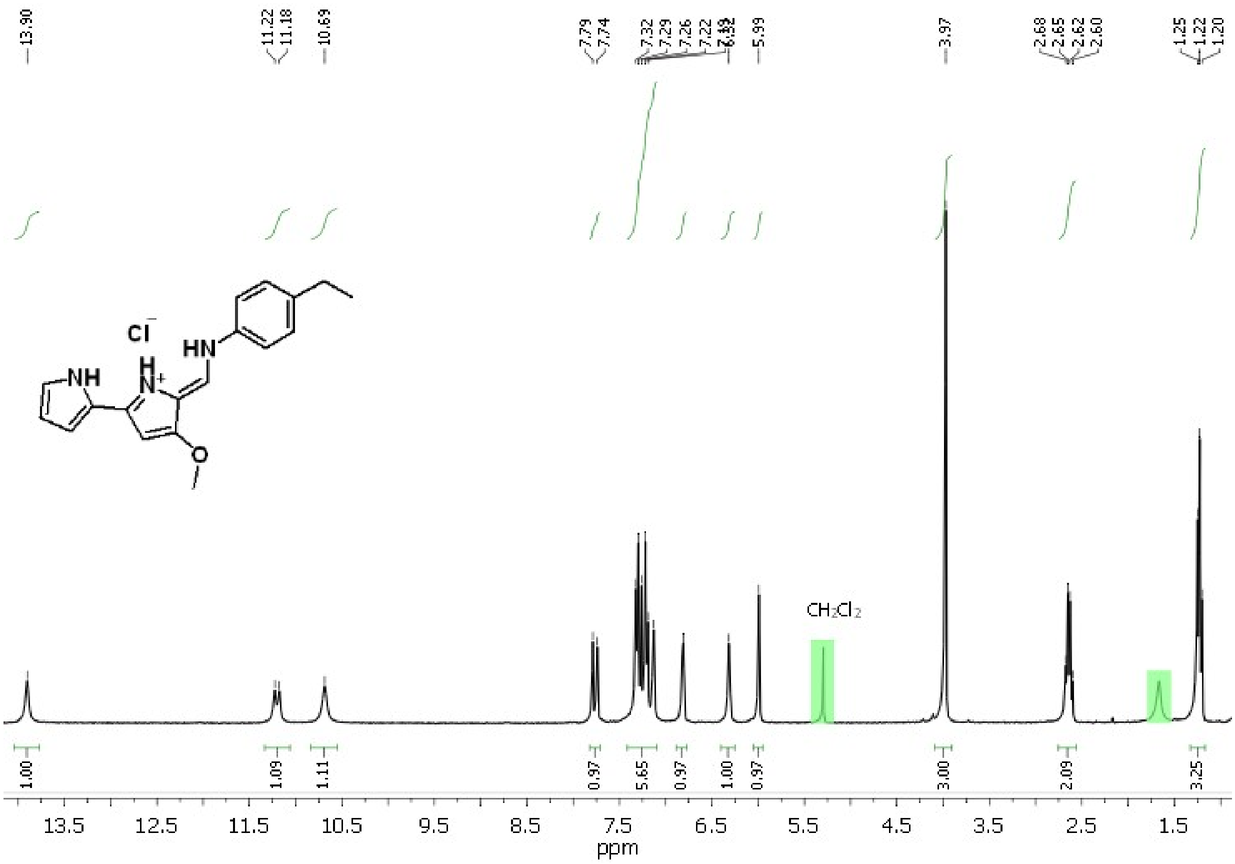

Figure S5. ^{1}H NMR ($CDCl_3$) of compound **2**. HCl.

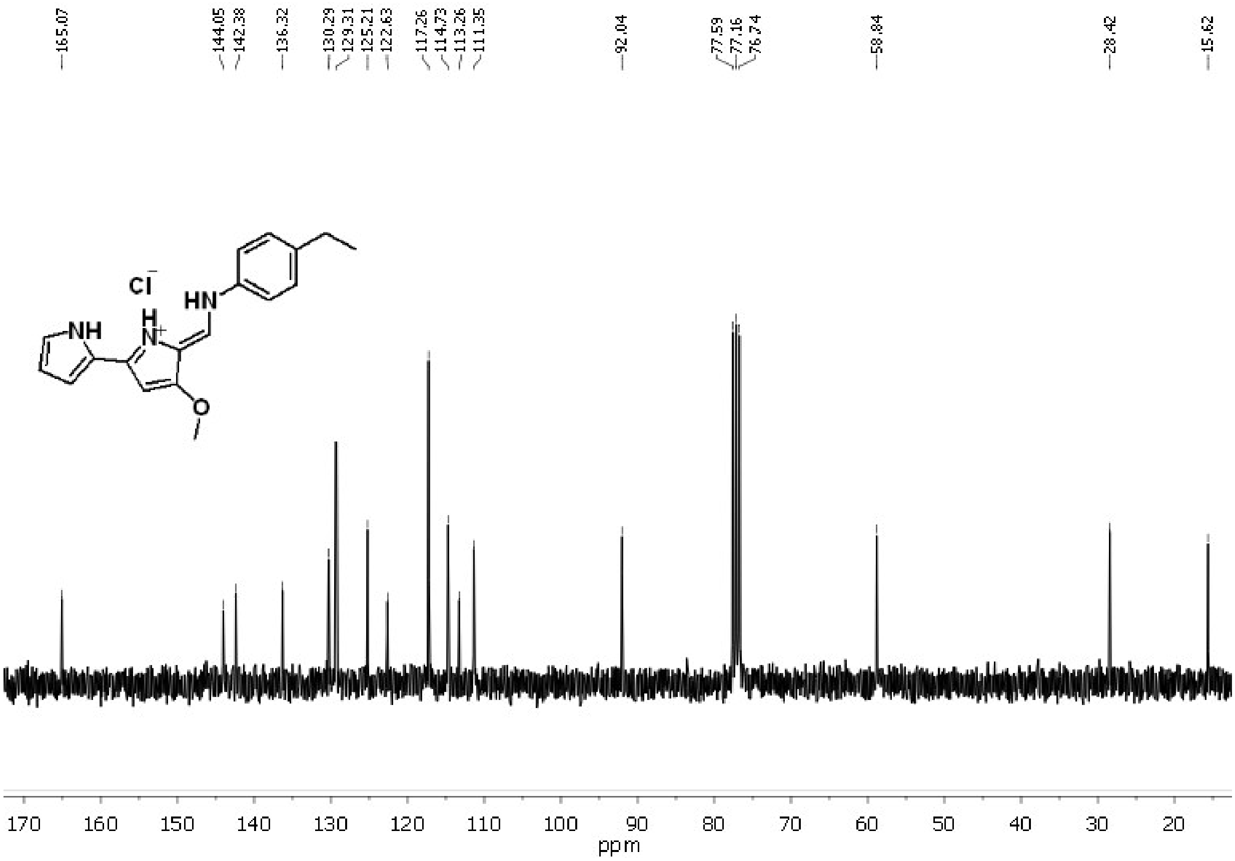

Figure S6. ^{13}C NMR ($CDCl_3$) of compound **2**. HCl.

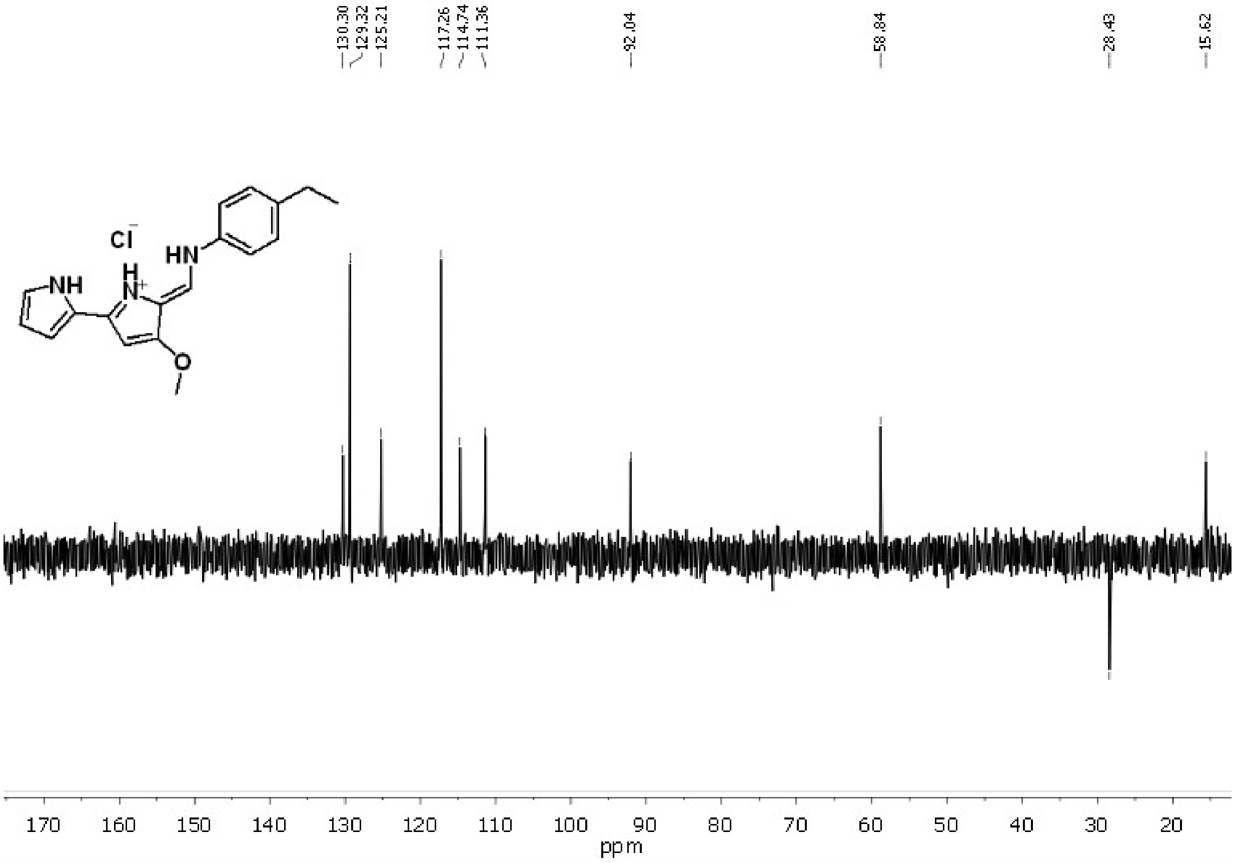

Figure S7. DEPT ^{13}C NMR ($CDCl_3$) of compound **2**. HCl.

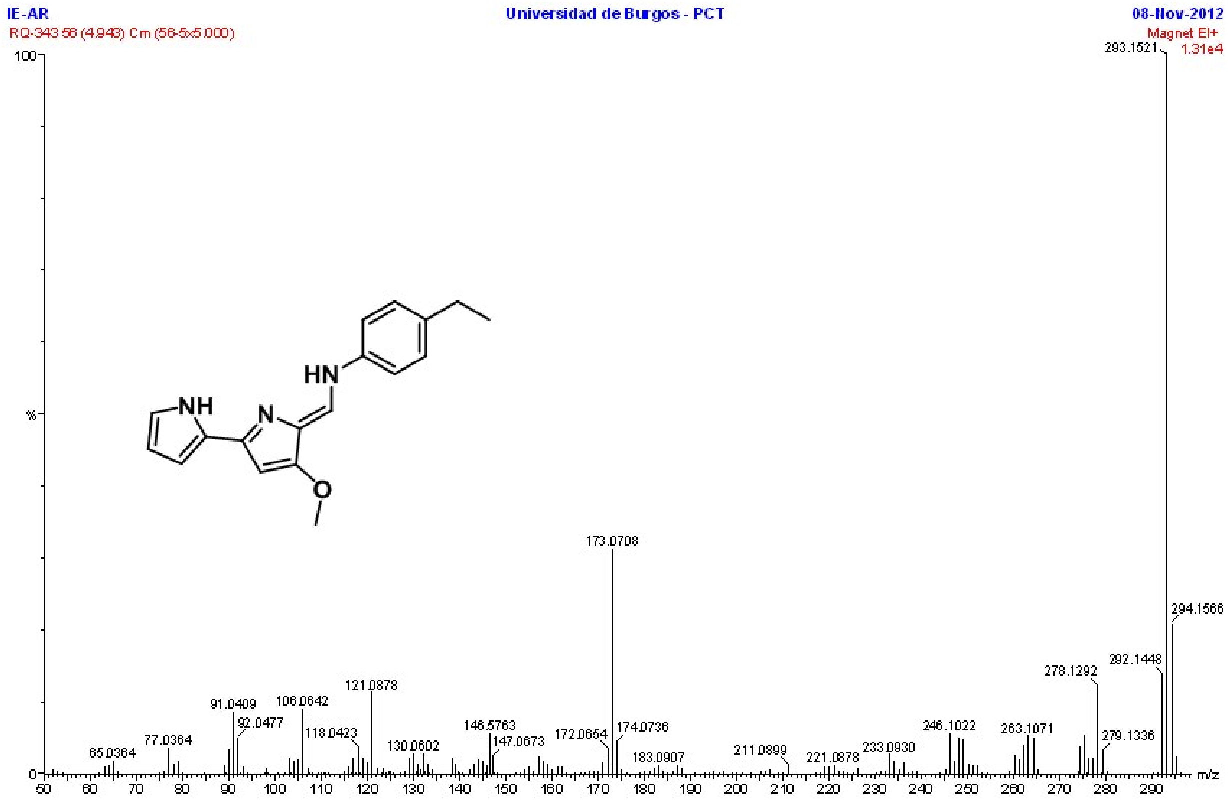

Figure S8. HRMS (EI) of compound **2**.

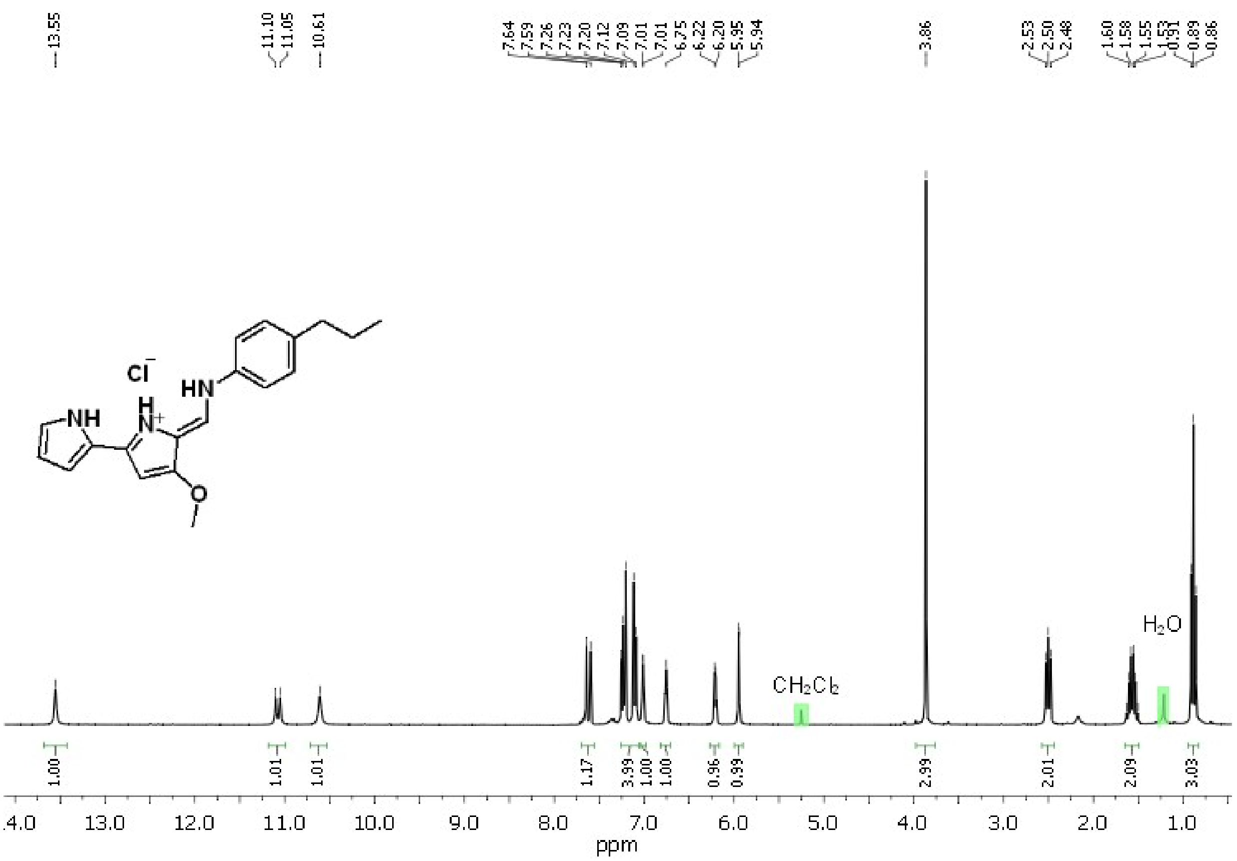

Figure S9. ^{1}H NMR ($CDCl_3$) of compound **3**. HCl.

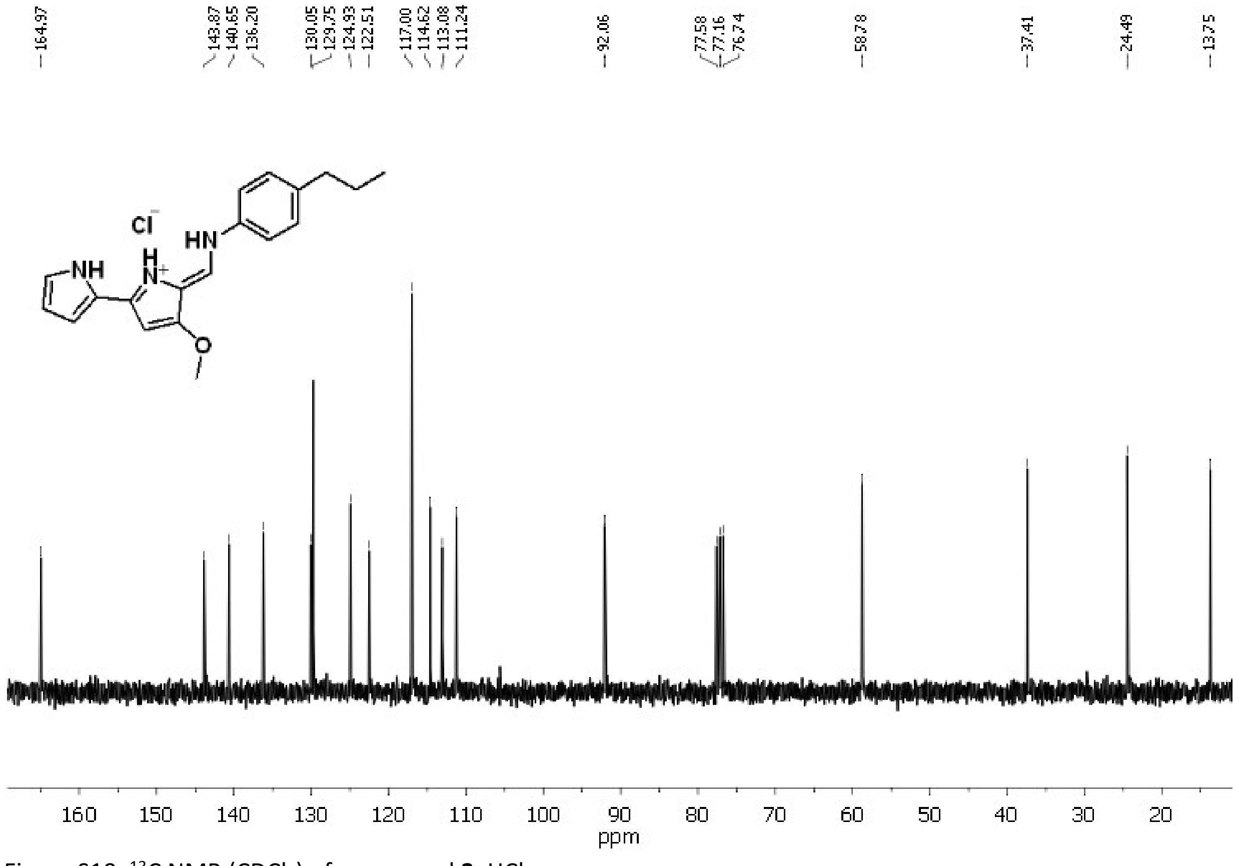

Figure S10. ^{13}C NMR ($CDCl_3$) of compound **3**. HCl.

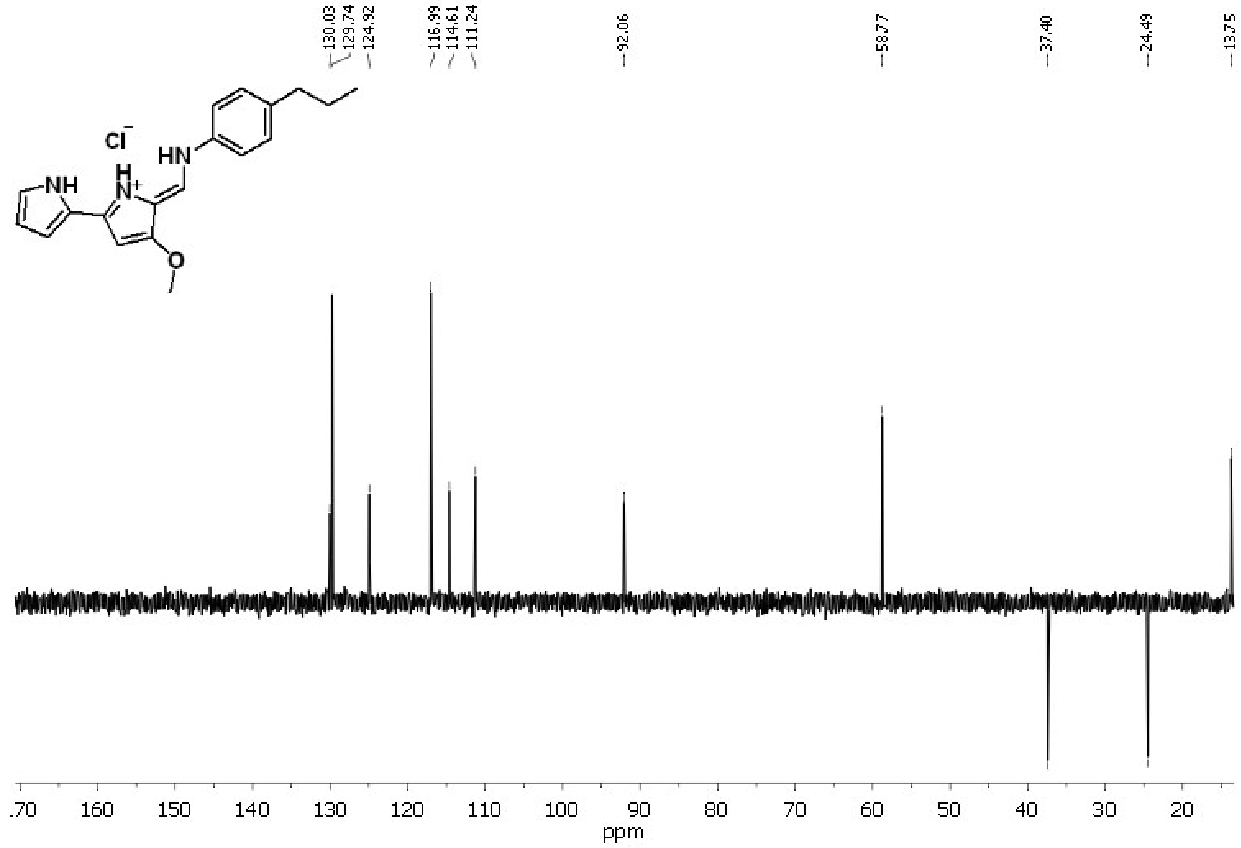

Figure S11. DEPT ^{13}C NMR ($CDCl_3$) of compound **3**. HCl.

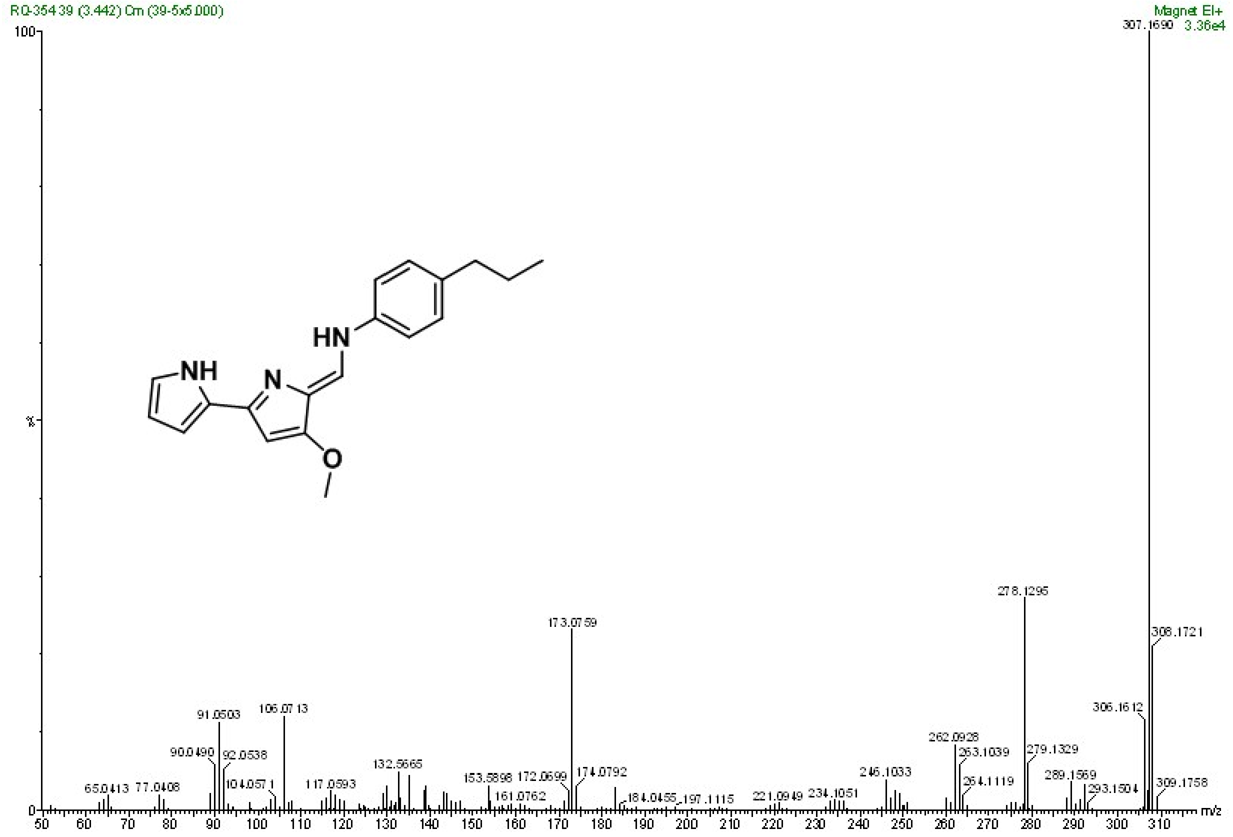

Figure S12. HRMS (EI) of compound **3**.

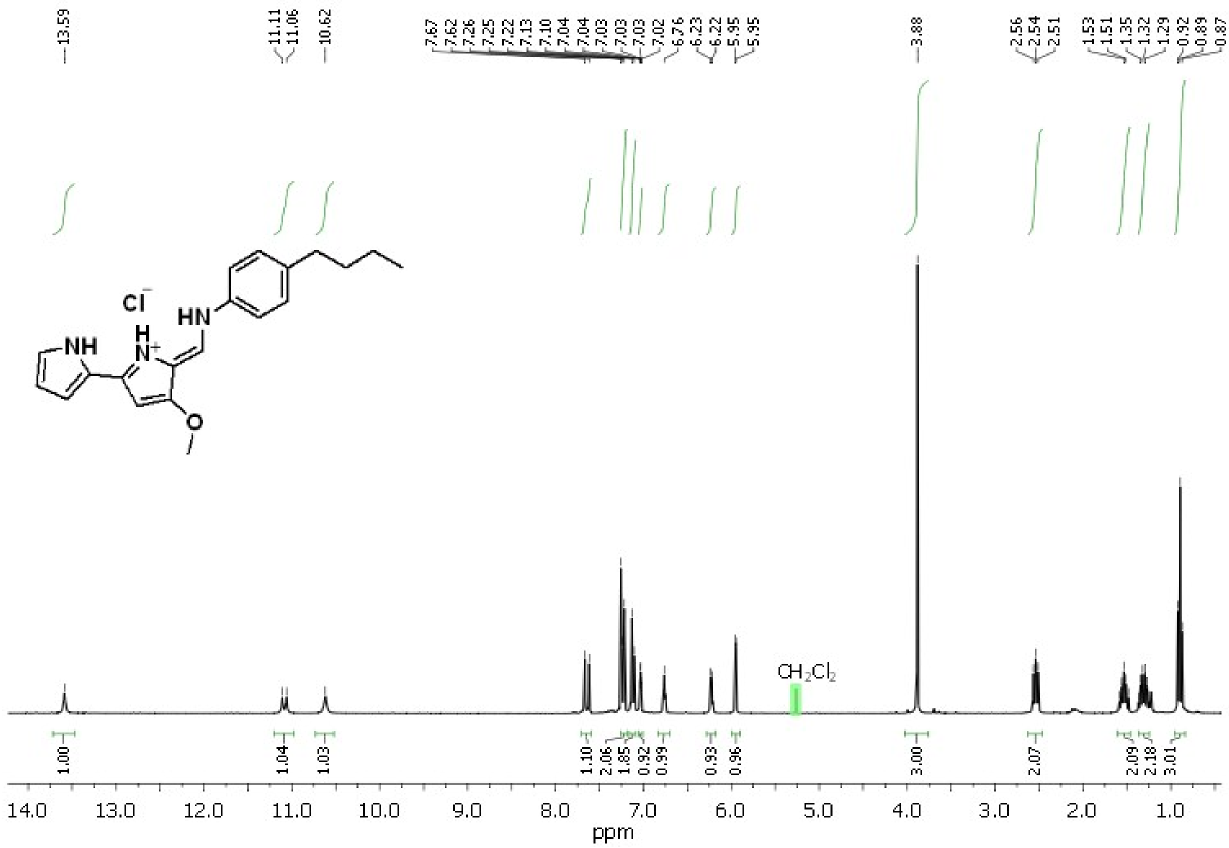

Figure S13. ^{1}H NMR ($CDCl_3$) of compound **4**. HCl.

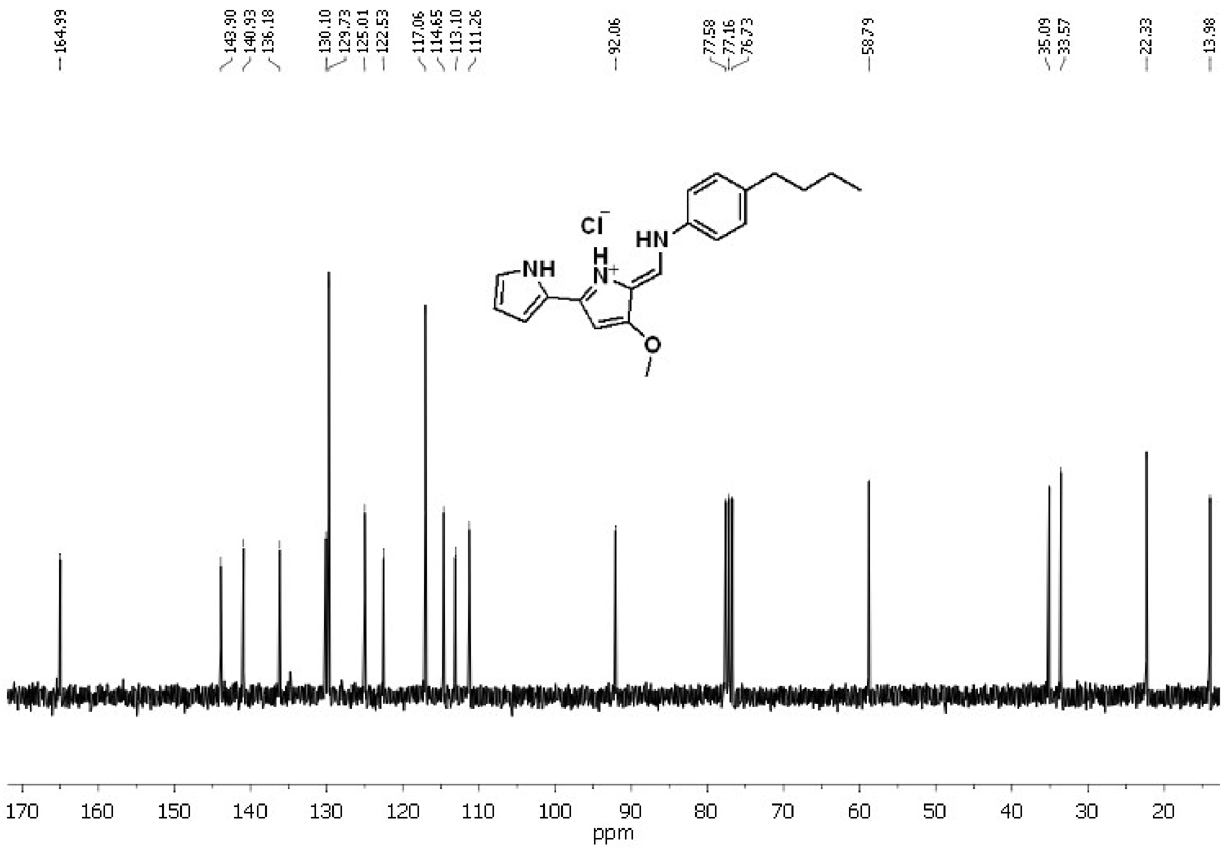

Figure S14. ^{13}C NMR ($CDCl_3$) of compound **4**. HCl.

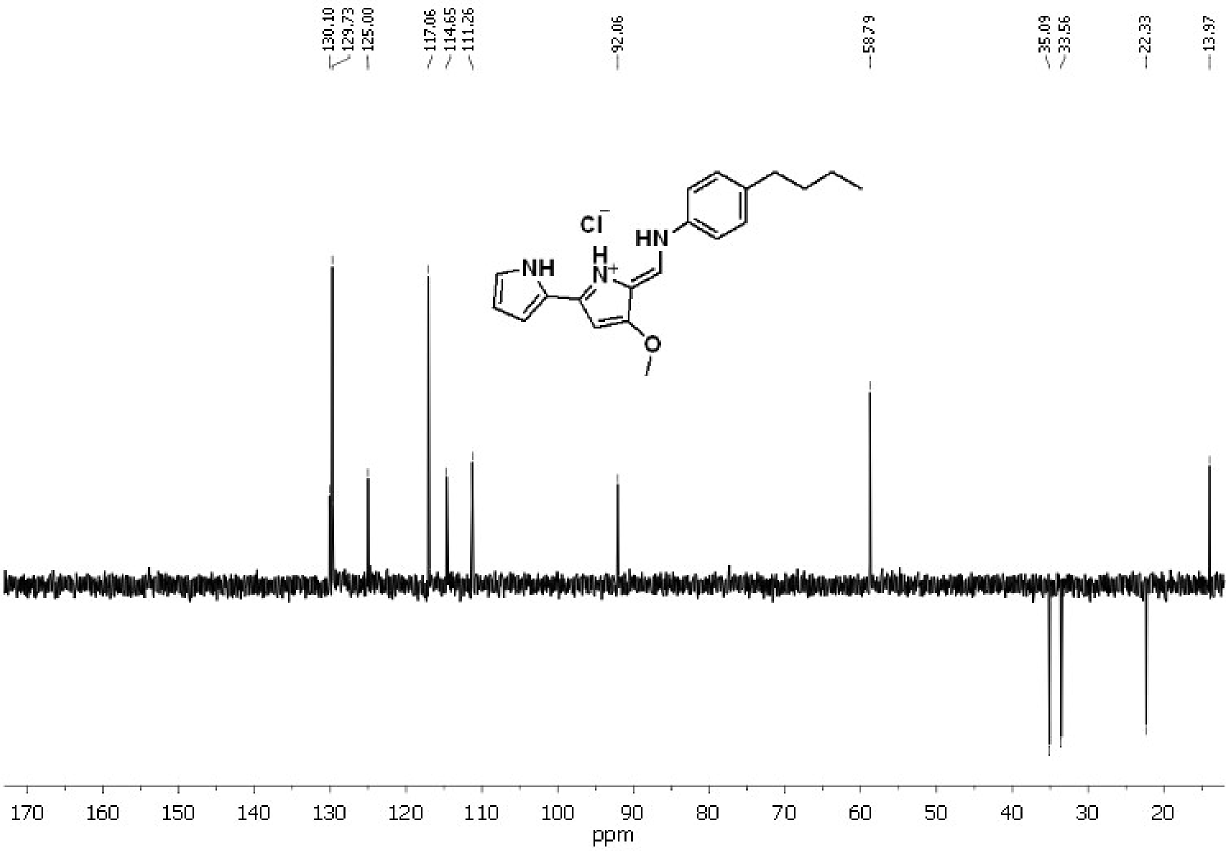

Figure S15. DEPT ^{13}C NMR ($CDCl_3$) of compound **4**. HCl.

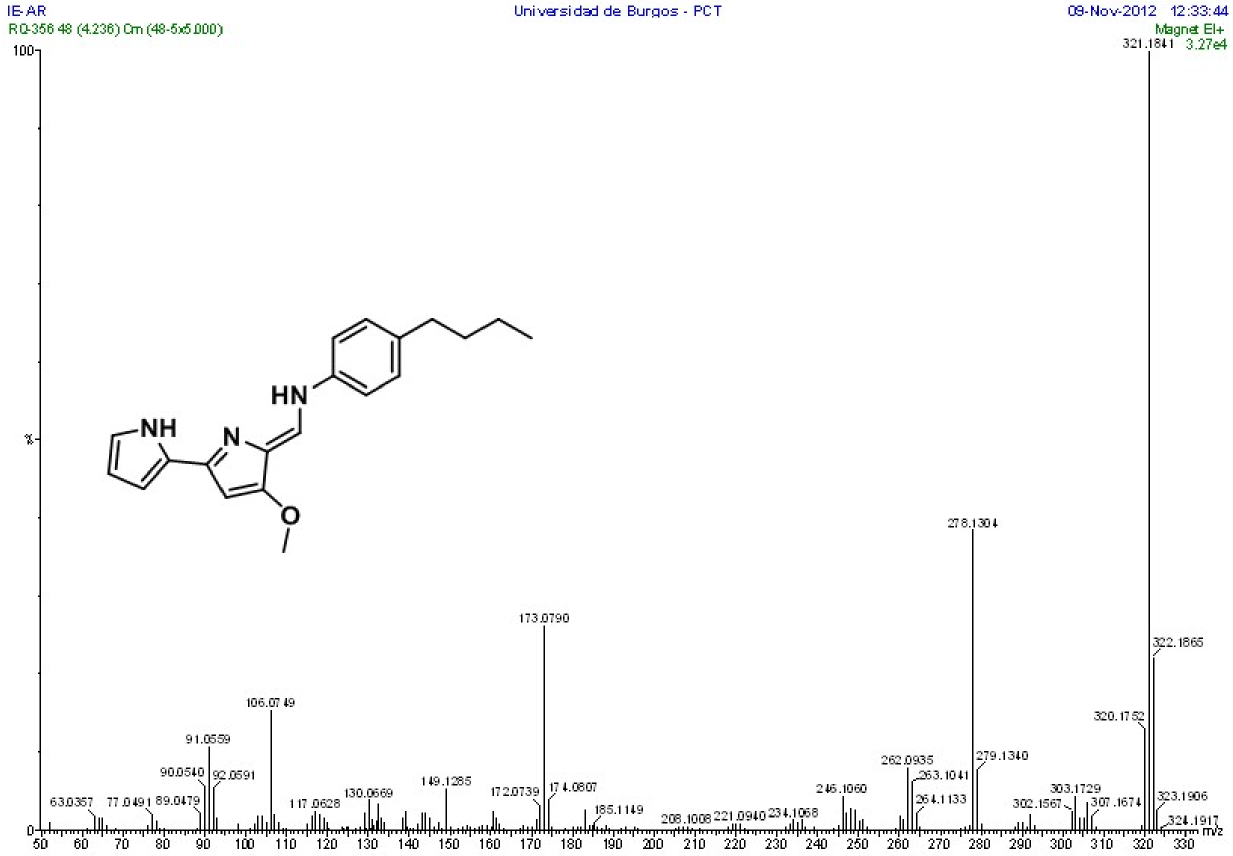

Figure S16. HRMS (EI) of compound **4**.

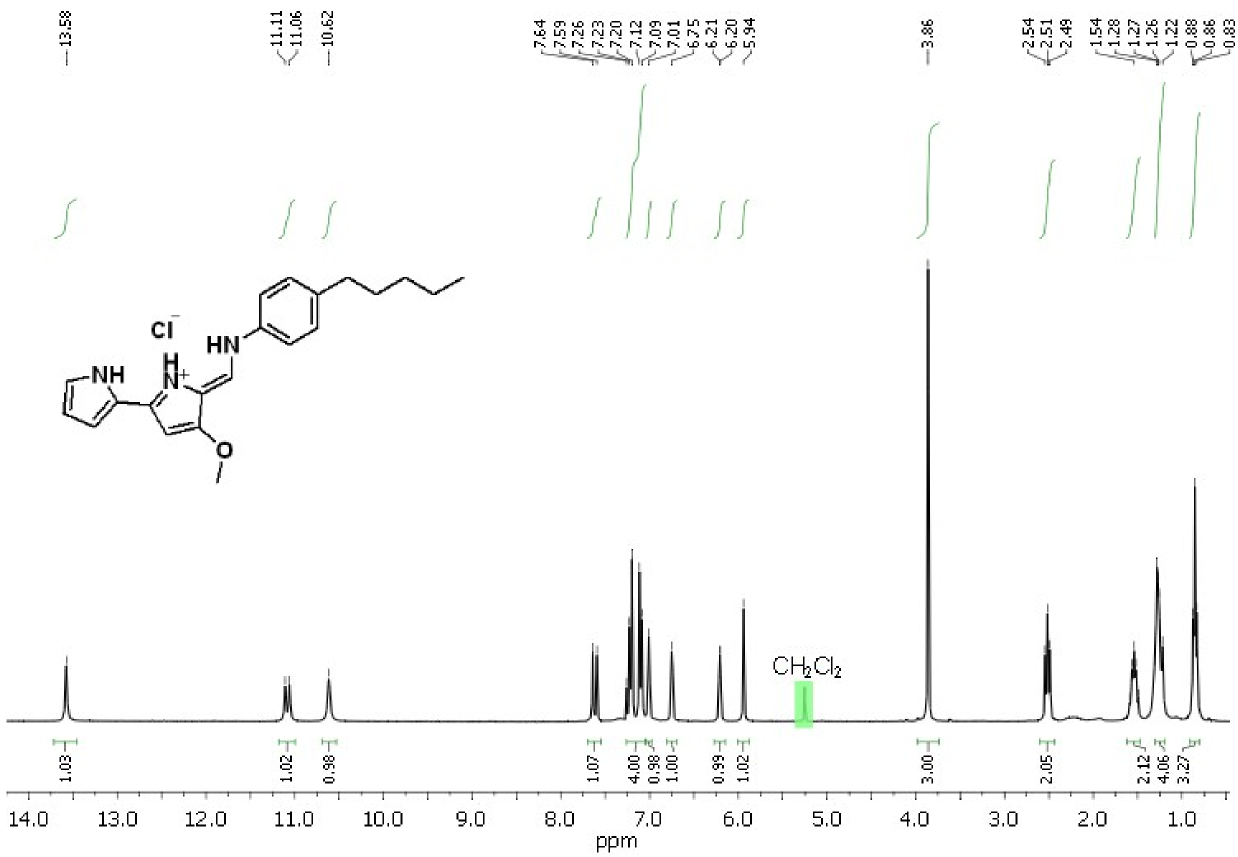

Figure S17. ^{1}H NMR ($CDCl_3$) of compound **6**. HCl.

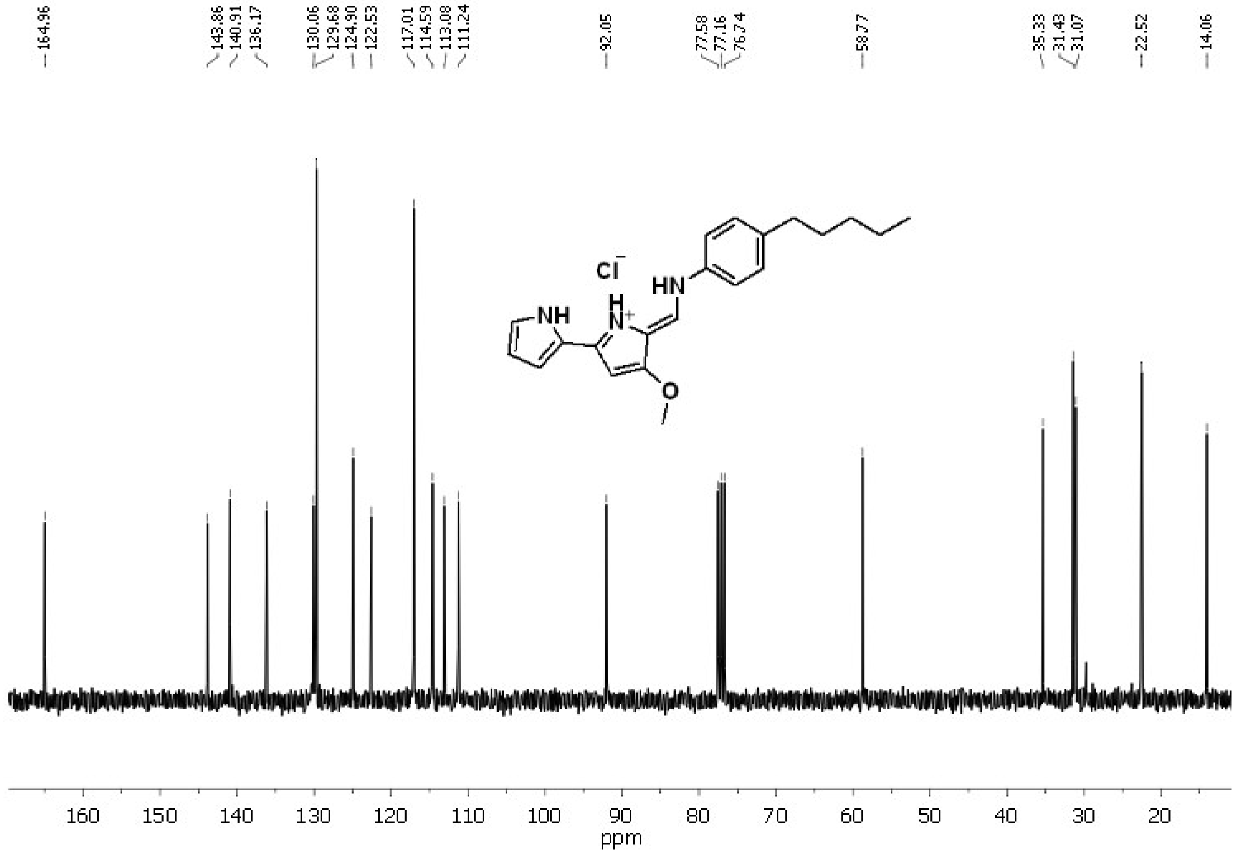

Figure S18. ^{13}C NMR ($CDCl_3$) of compound **6**. HCl.

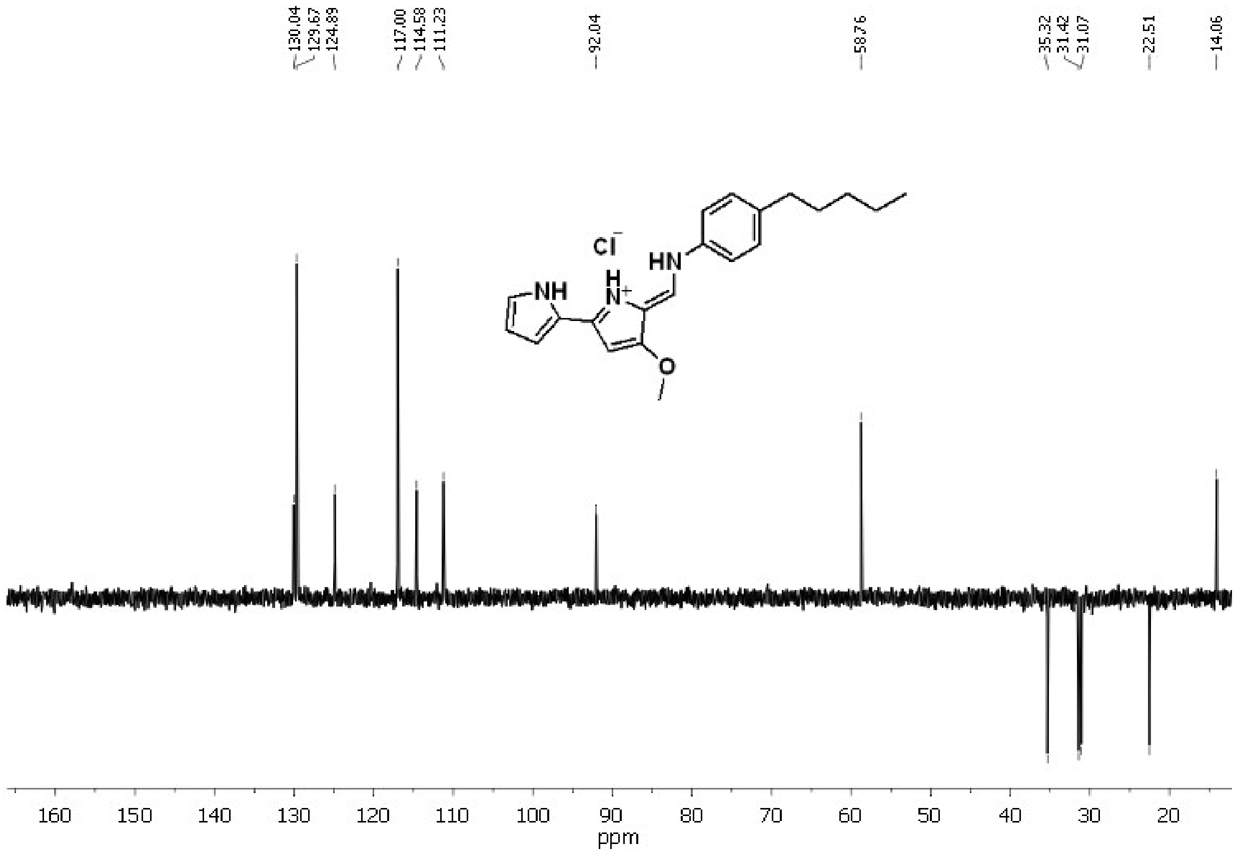

Figure S19. DEPT ^{13}C NMR ($CDCl_3$) of compound **6**. HCl.

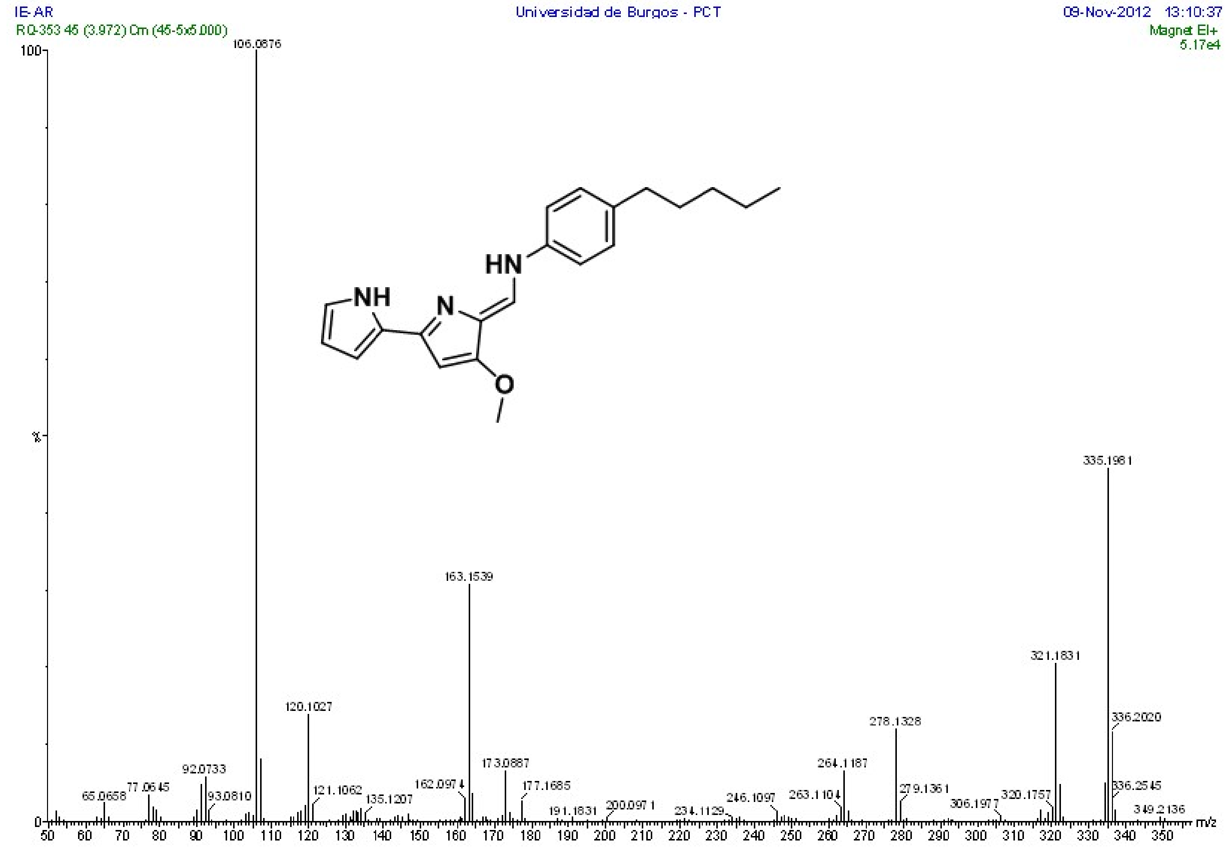

Figure S20. HRMS (EI) of compound **6**.

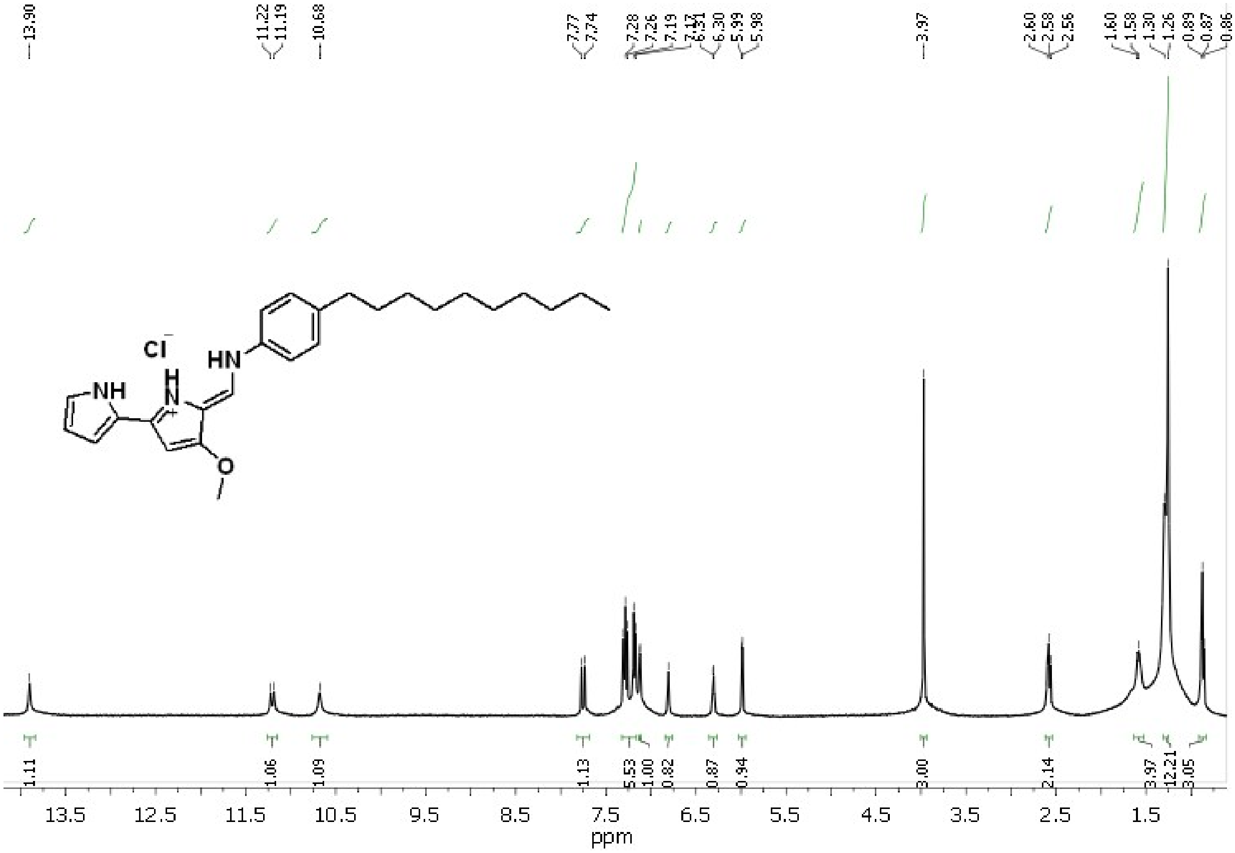

Figure S21. ^{1}H NMR ($CDCl_3$) of compound **7**. HCl.

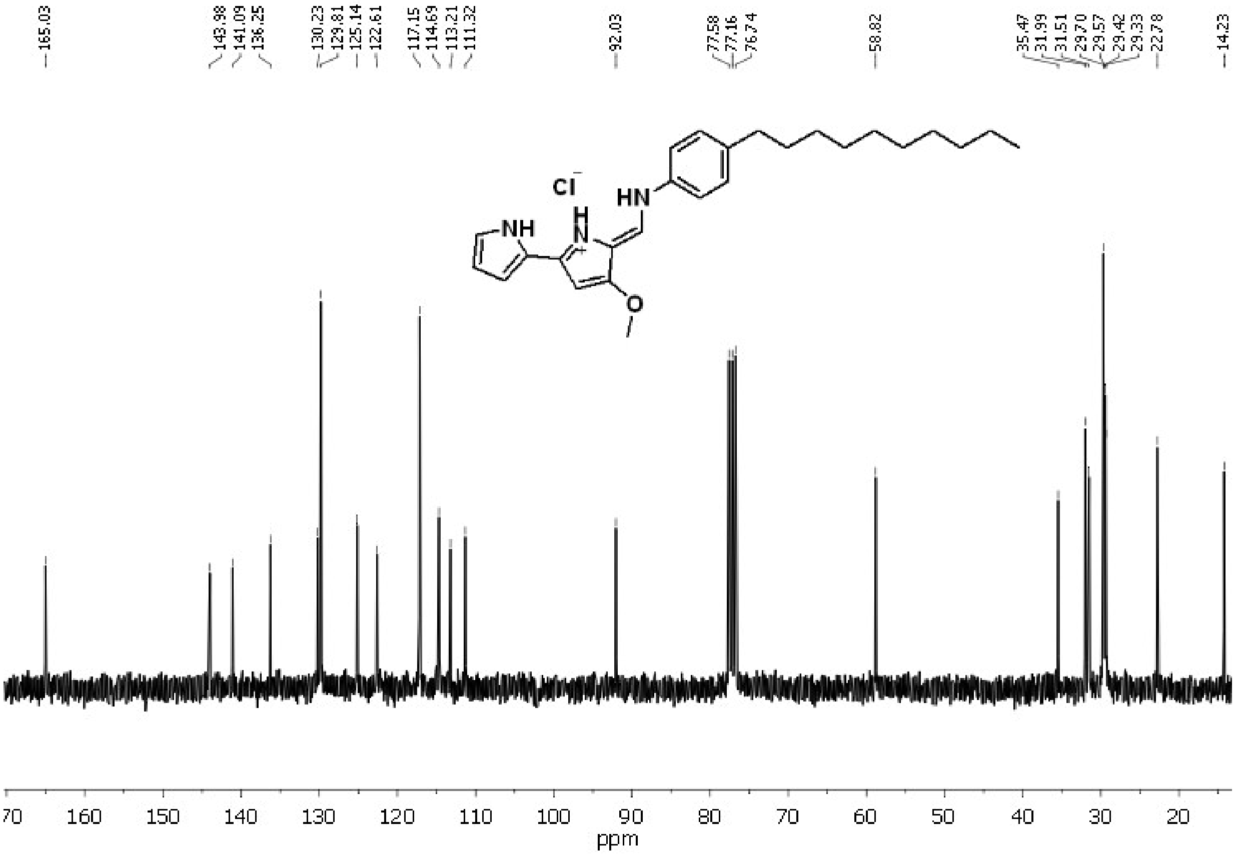

Figure S22. ^{13}C NMR ($CDCl_3$) of compound **7**. HCl.

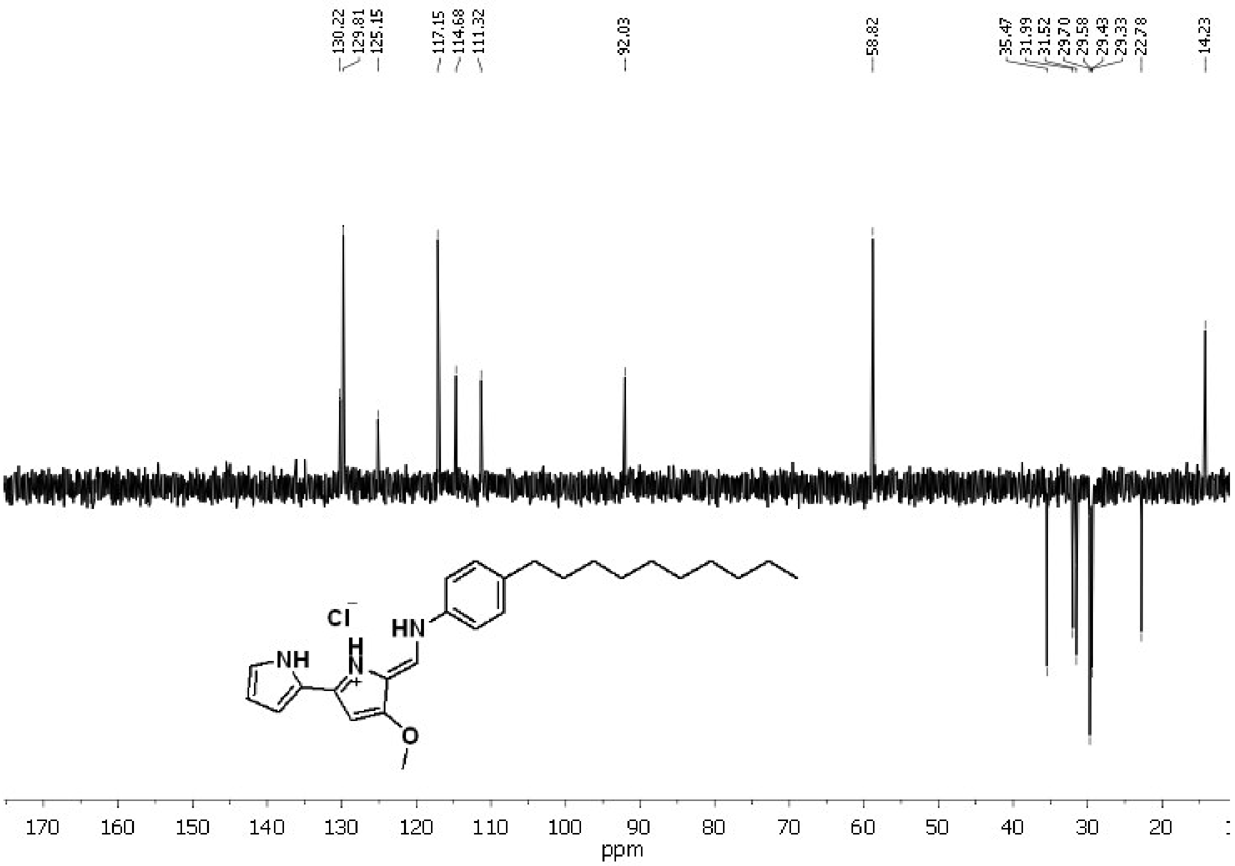

Figure S23. DEPT ^{13}C NMR ($CDCl_3$) of compound **7**. HCl.

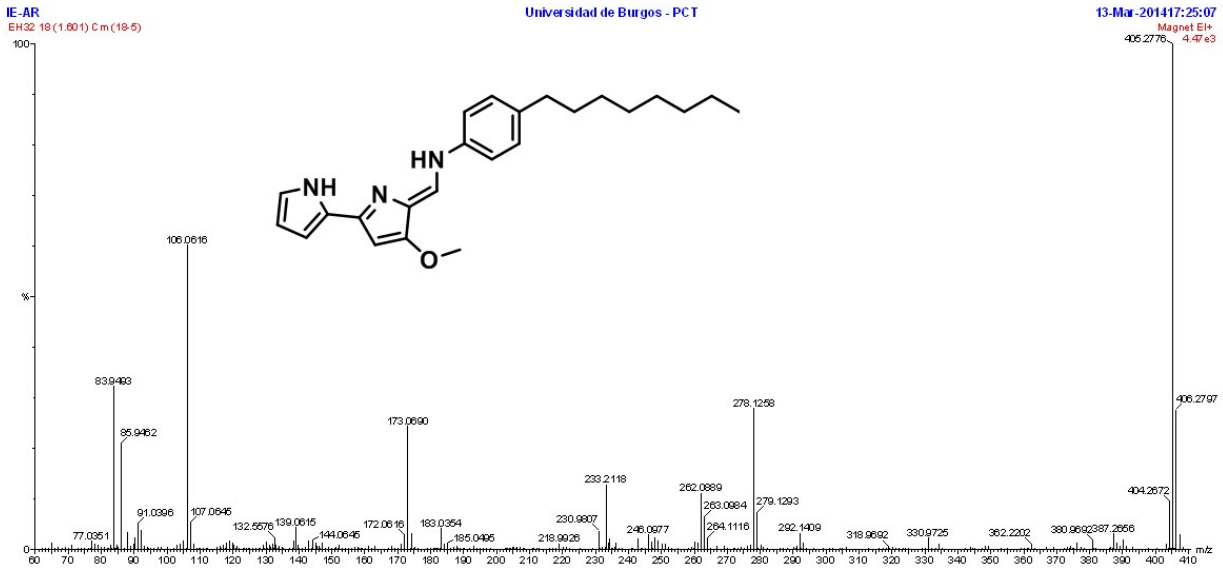

Figure S24. HRMS (EI) of compound **7**.

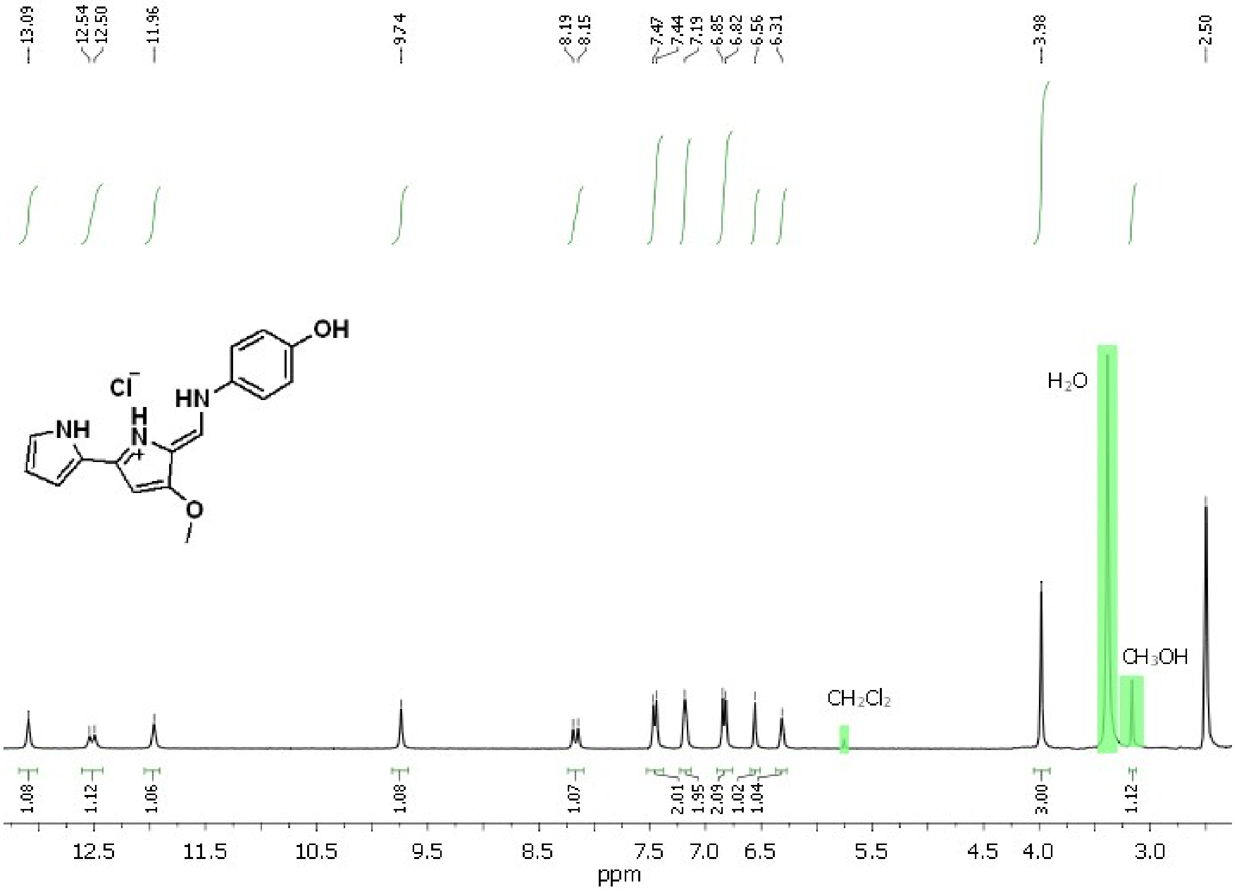

Figure S25. ^{1}H NMR (DMSO-d_6) of compound **8**. HCl.

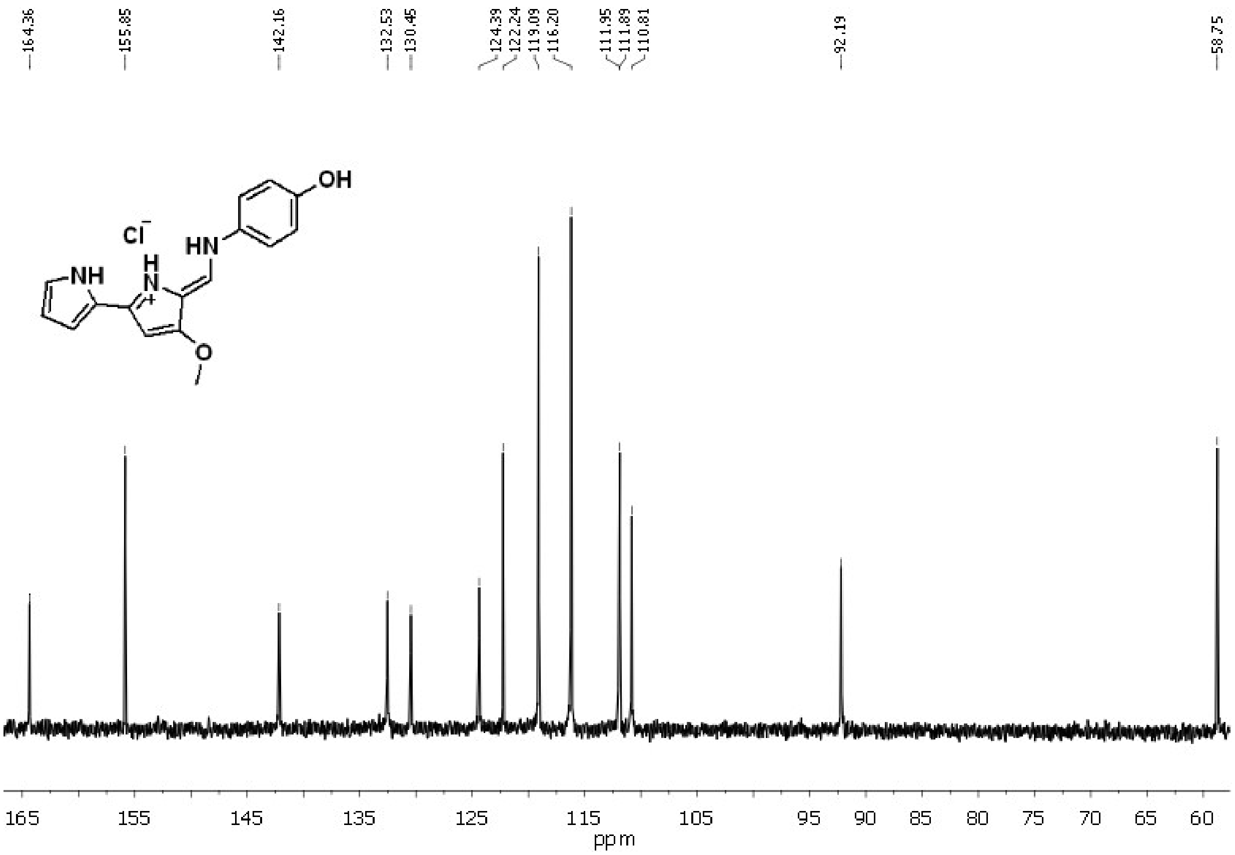

Figure S26. ^{13}C NMR (DMSO-d_6) of compound **8**. HCl.

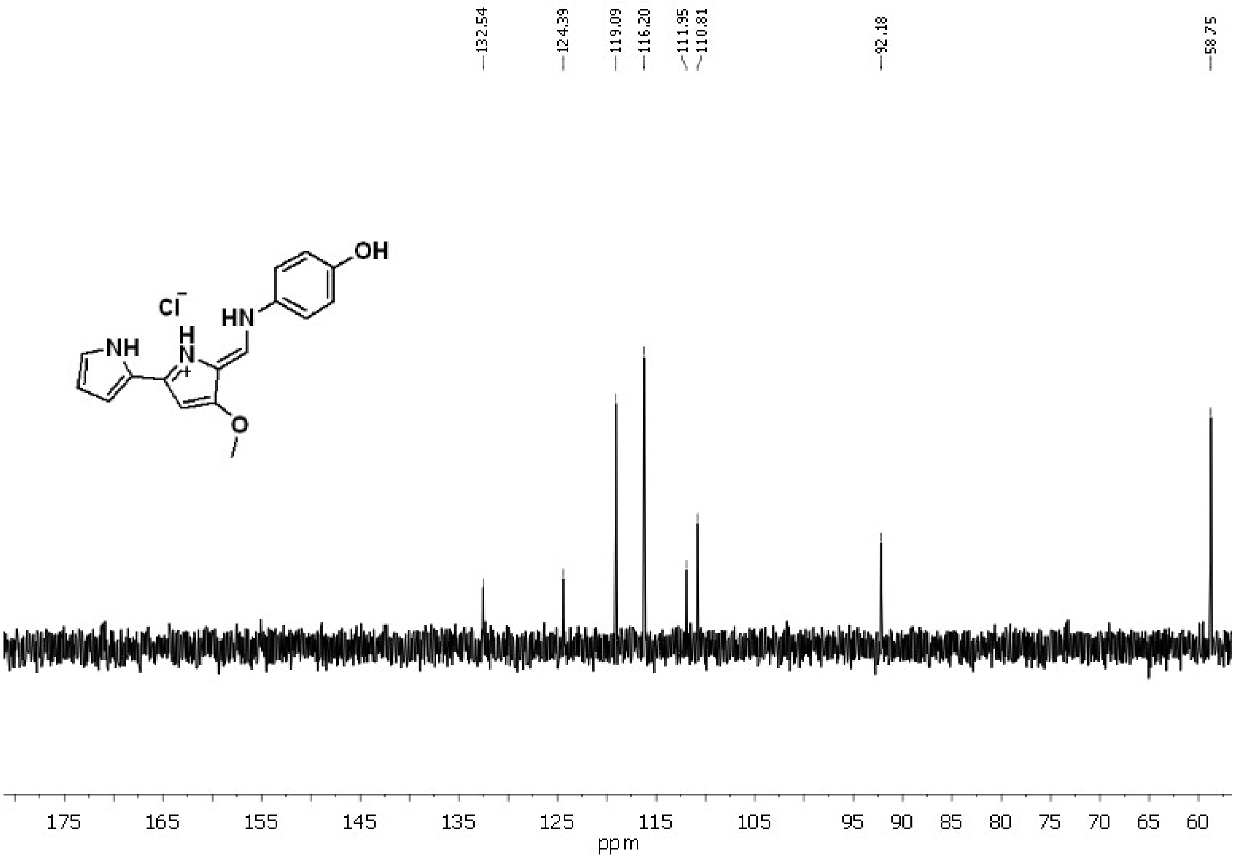

Figure S27. DEPT ^{13}C NMR (DMSO-d_6) of compound **8**. HCl.

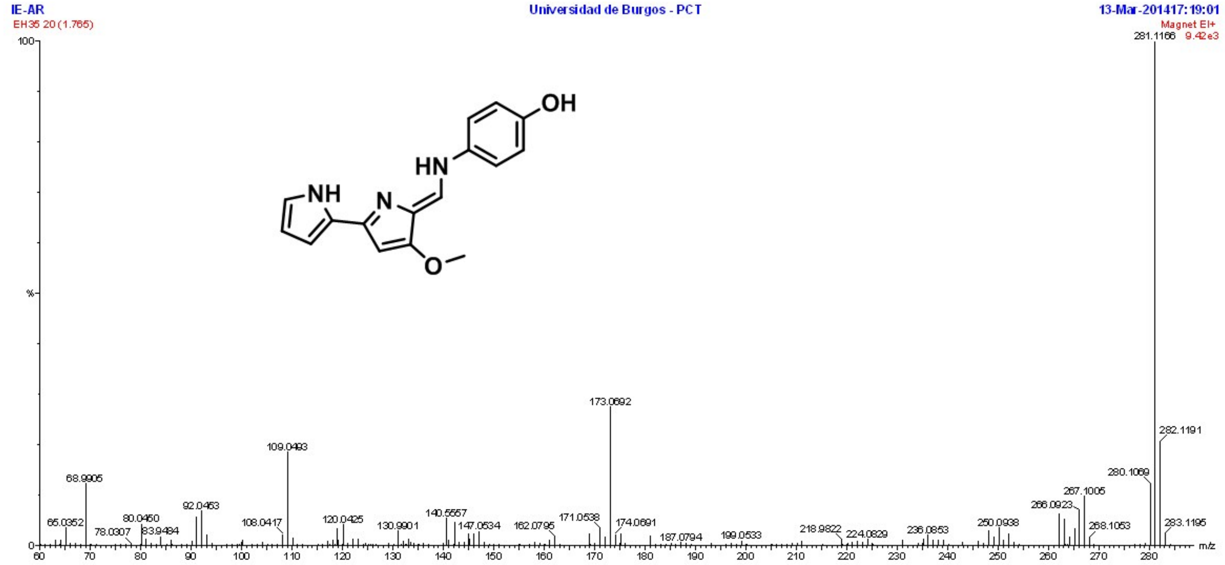

Figure S28. HRMS (EI) of compound **8**.

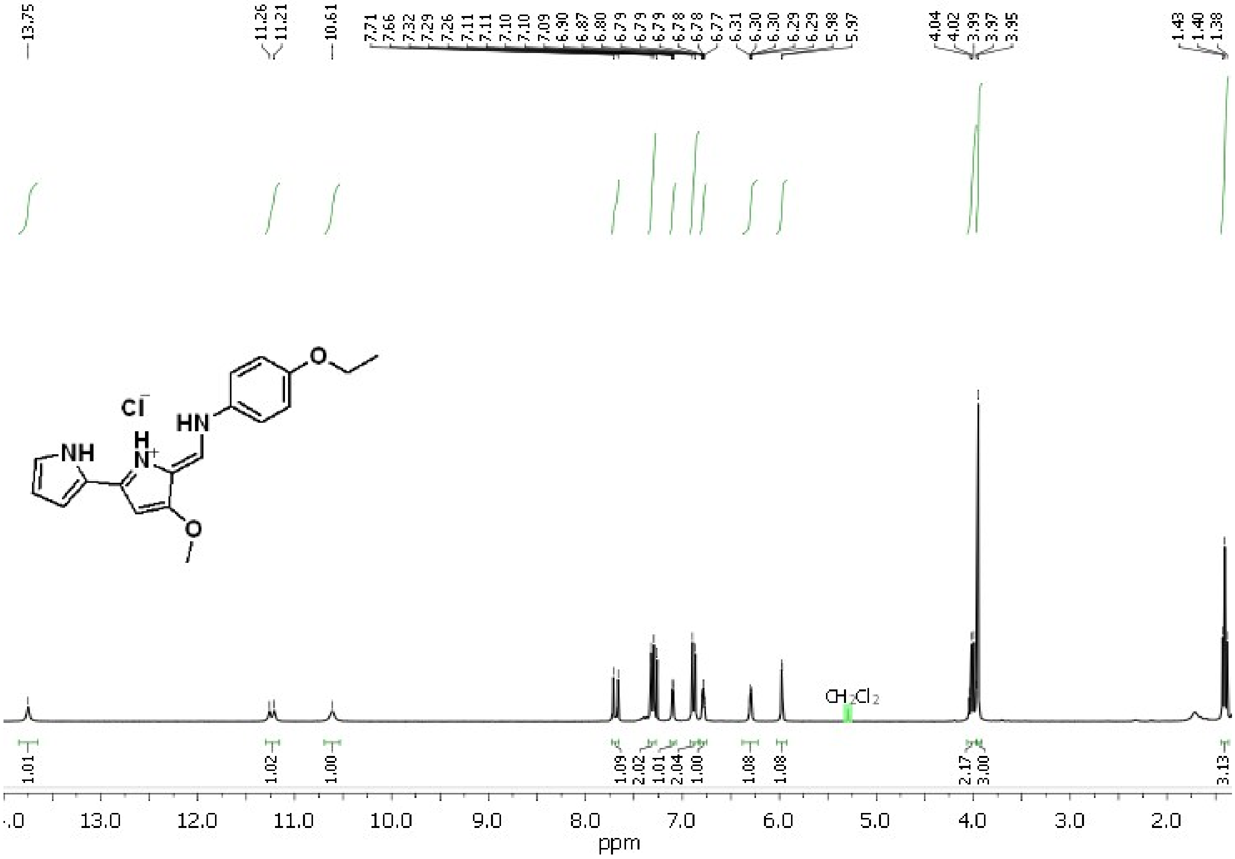

Figure S29. ^{1}H NMR ($CDCl_3$) of compound **10**. HCl.

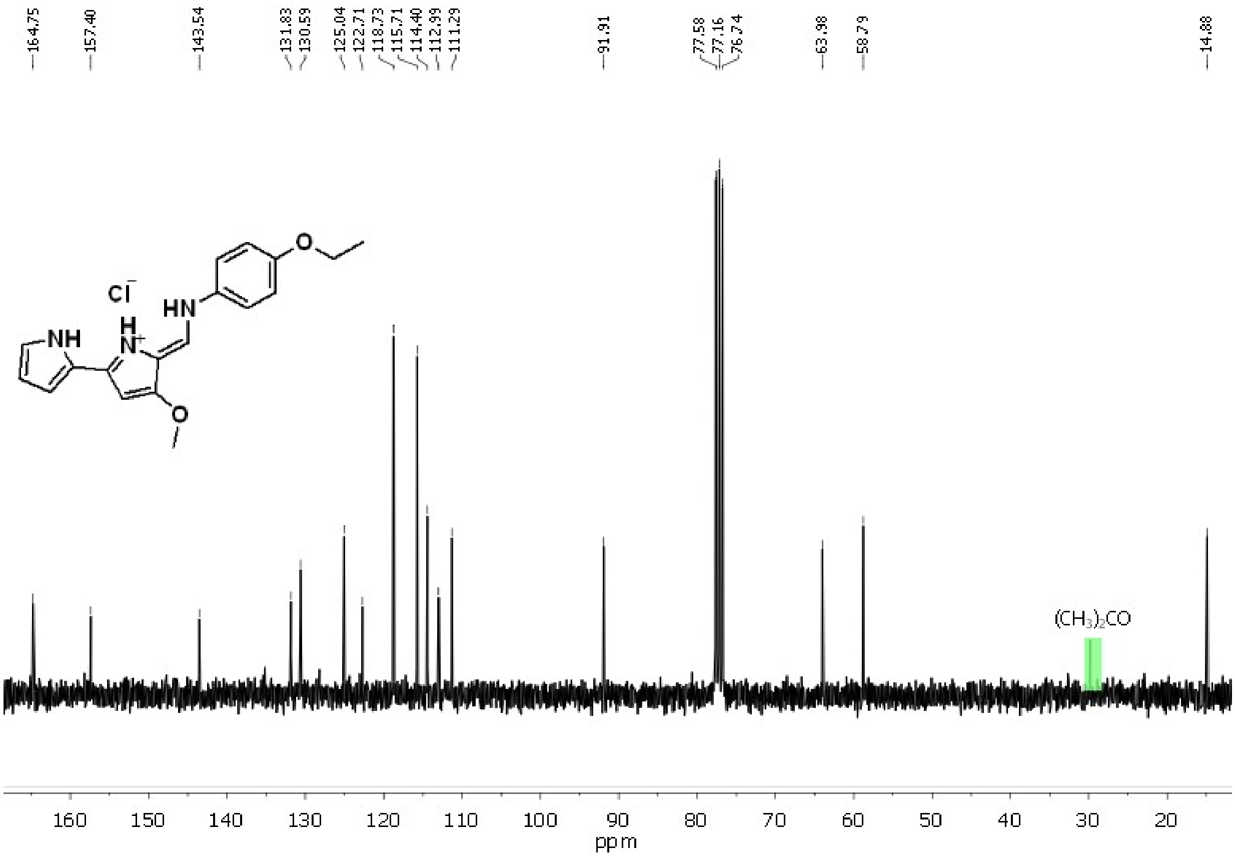

Figure S30. ^{13}C NMR ($CDCl_3$) of compound **10**. HCl.

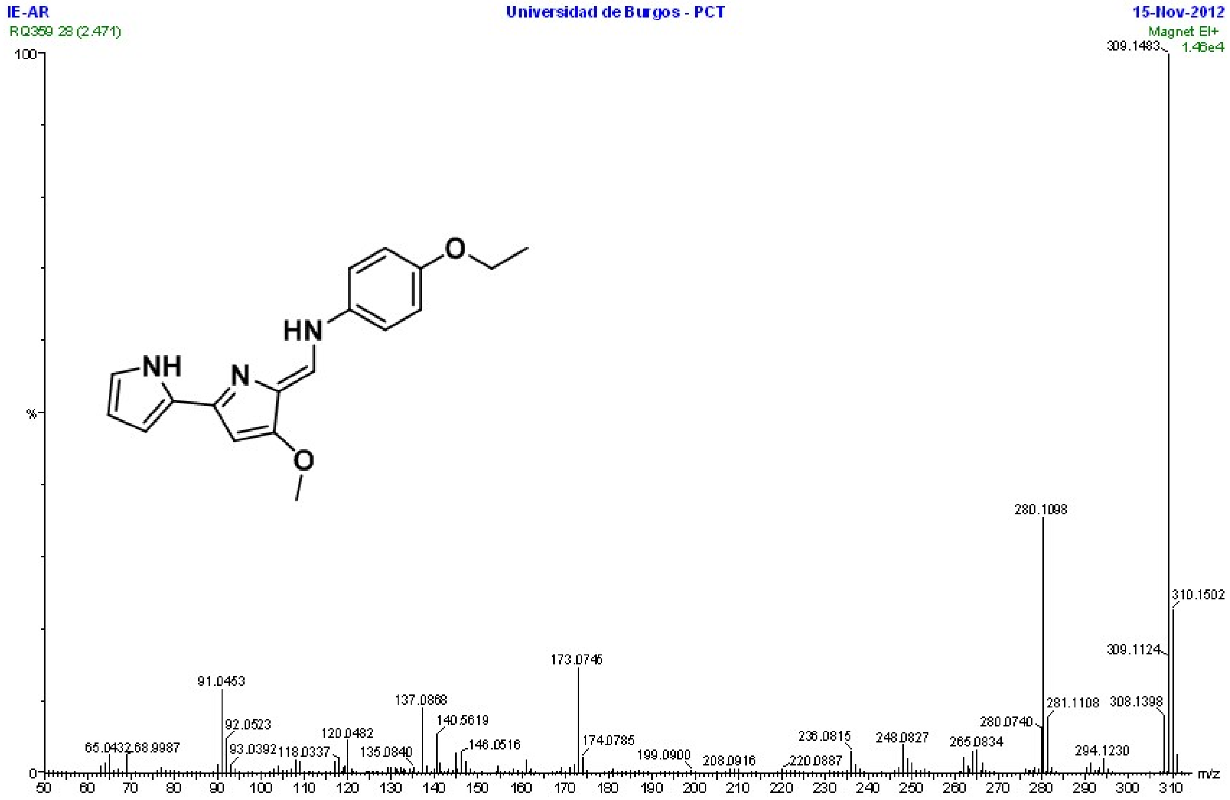

Figure S31. HRMS (EI) of compound **10**.

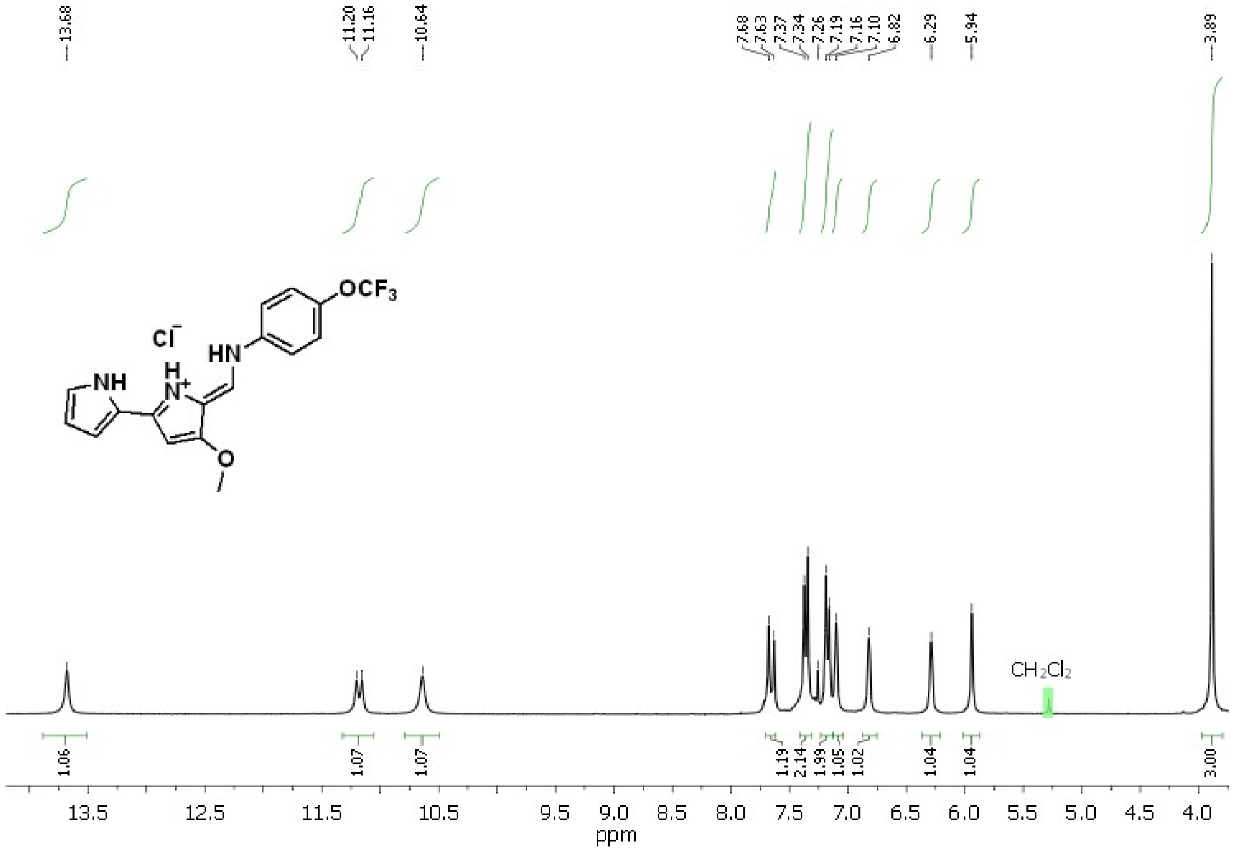

Figure S32. ^{1}H NMR ($CDCl_3$) of compound **11**. HCl.

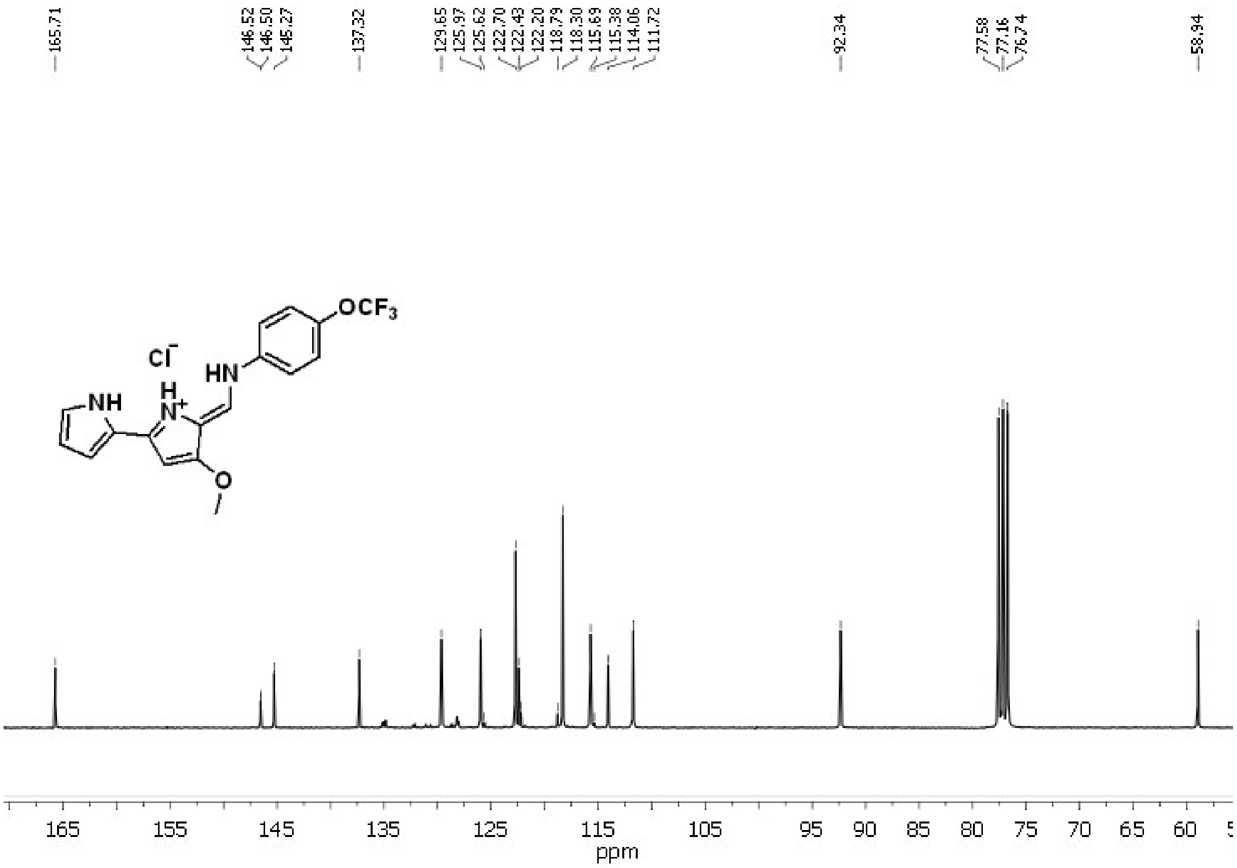

Figure S33. ^{13}C NMR ($CDCl_3$) of compound **11**. HCl.

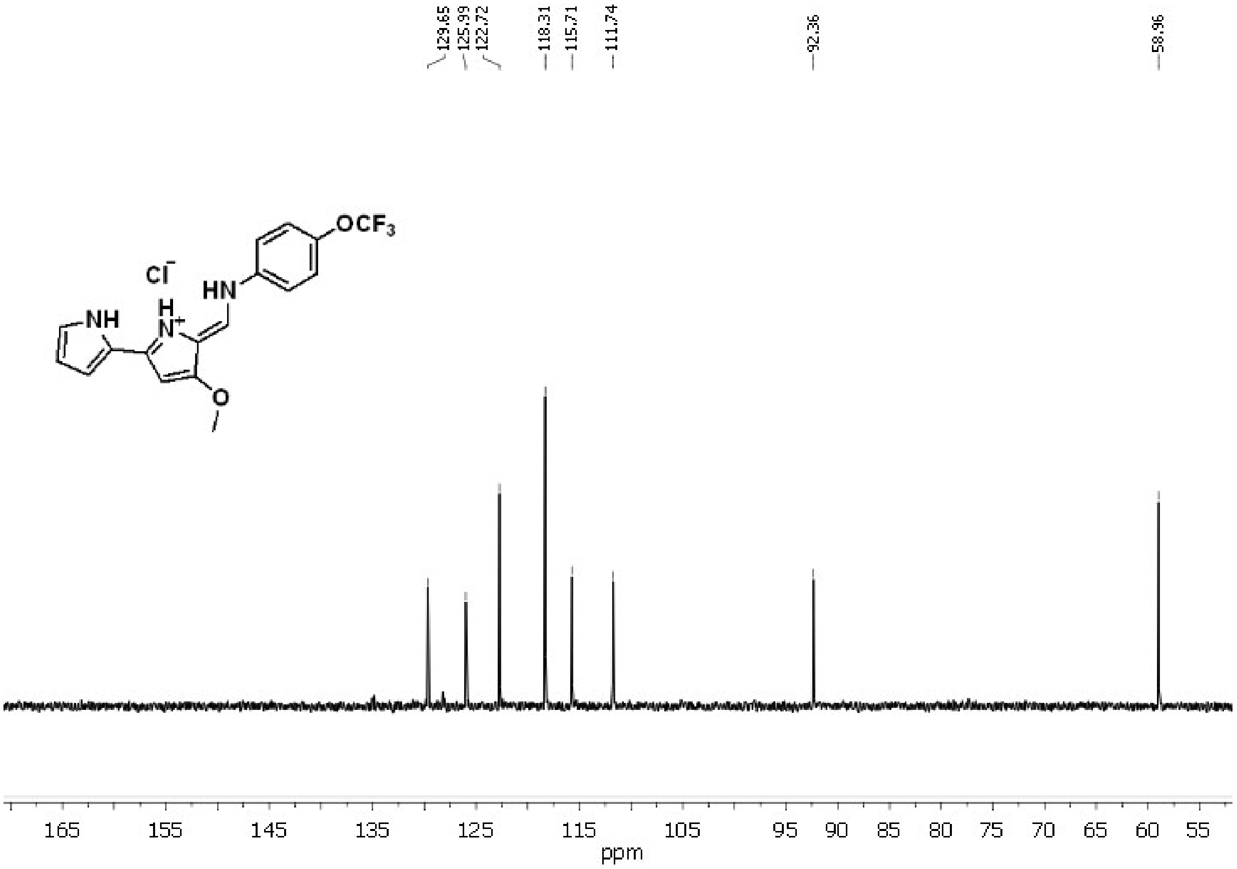

Figure S34. DEPT ^{13}C NMR ($CDCl_3$) of compound **11**. HCl.

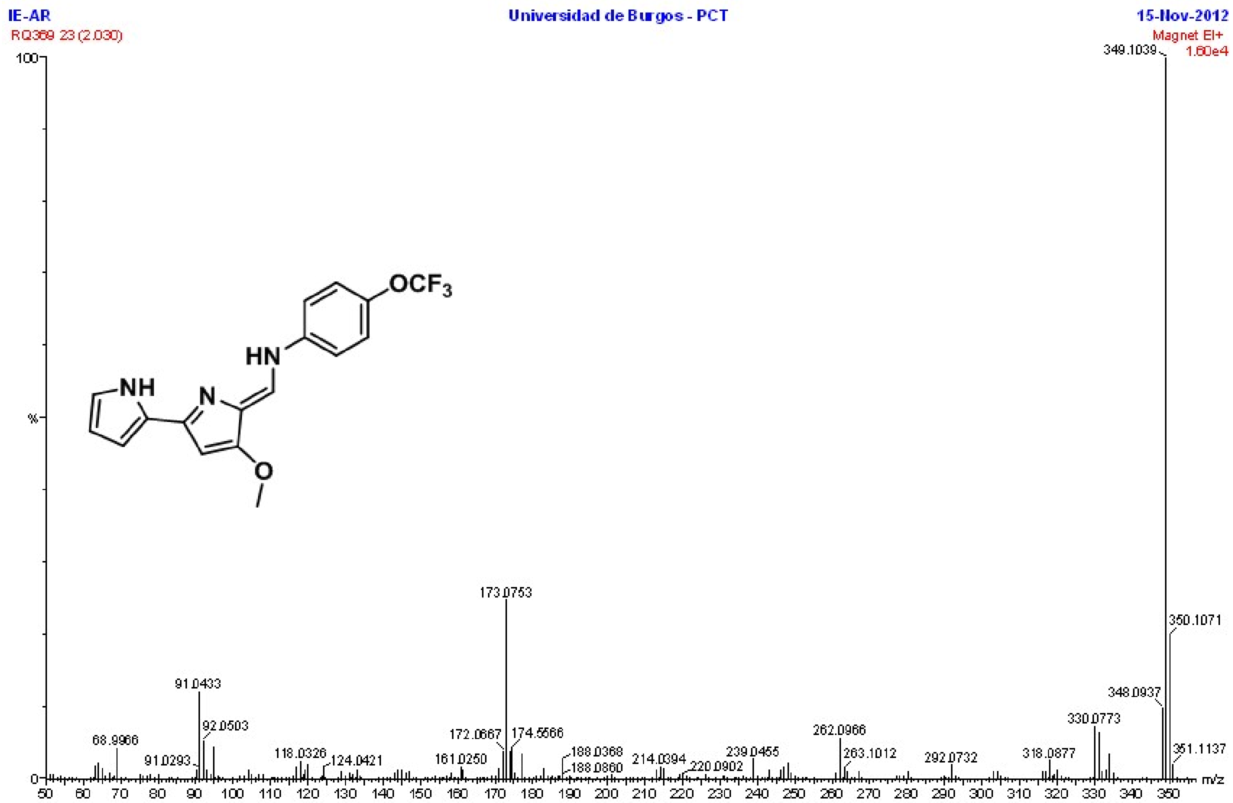

Figure S35. HRMS (EI) of compound **11**.

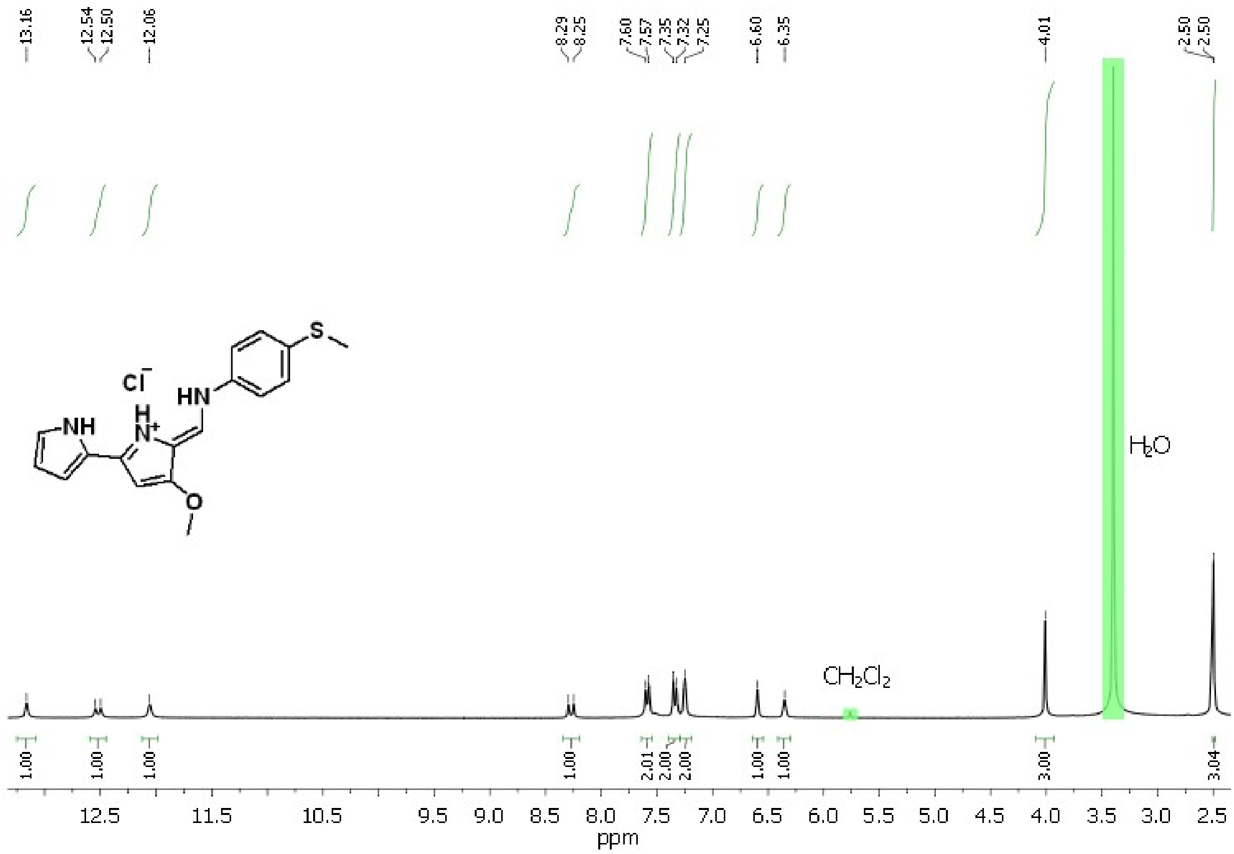

Figure S36. ^{1}H NMR (DMSO-d_6) of compound **12**. HCl.

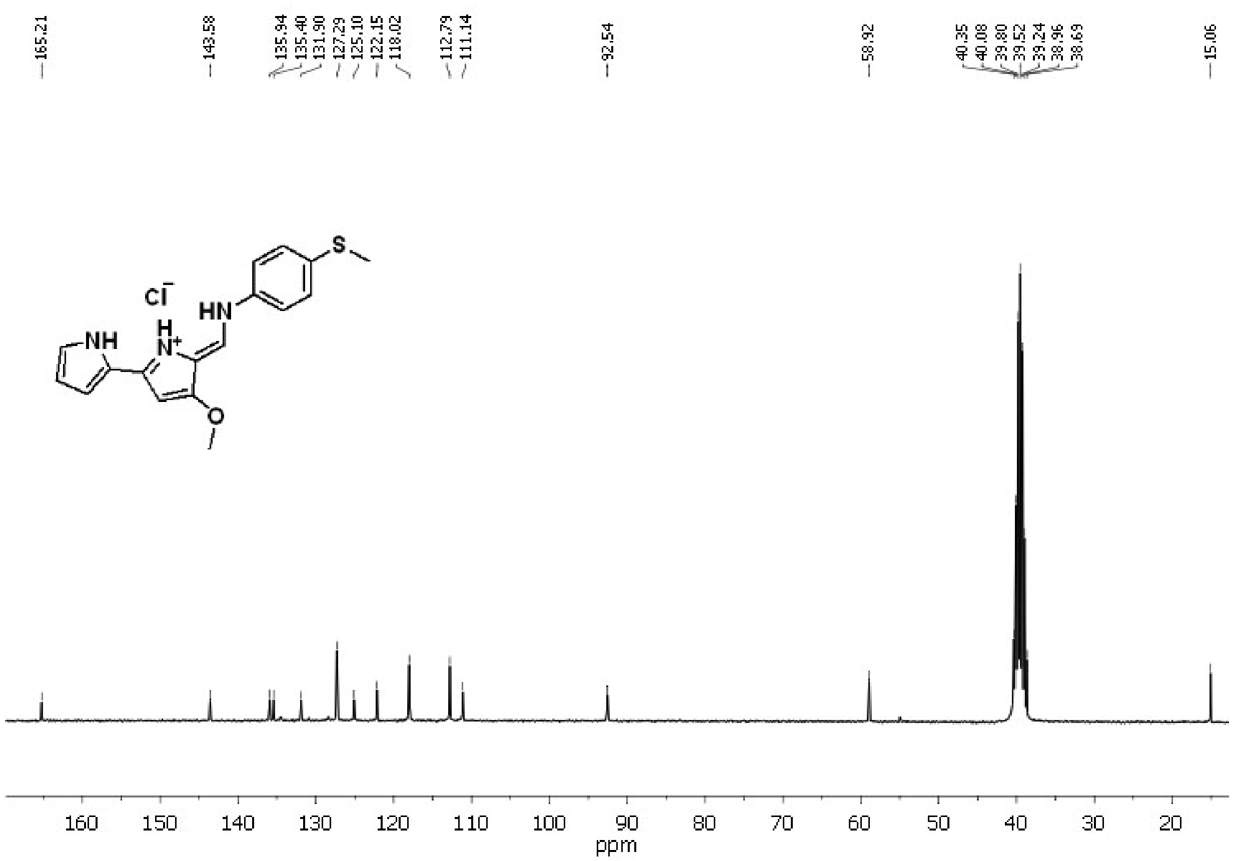

Figure S37. ^{13}C NMR (DMSO-d_6) of compound **12**. HCl.

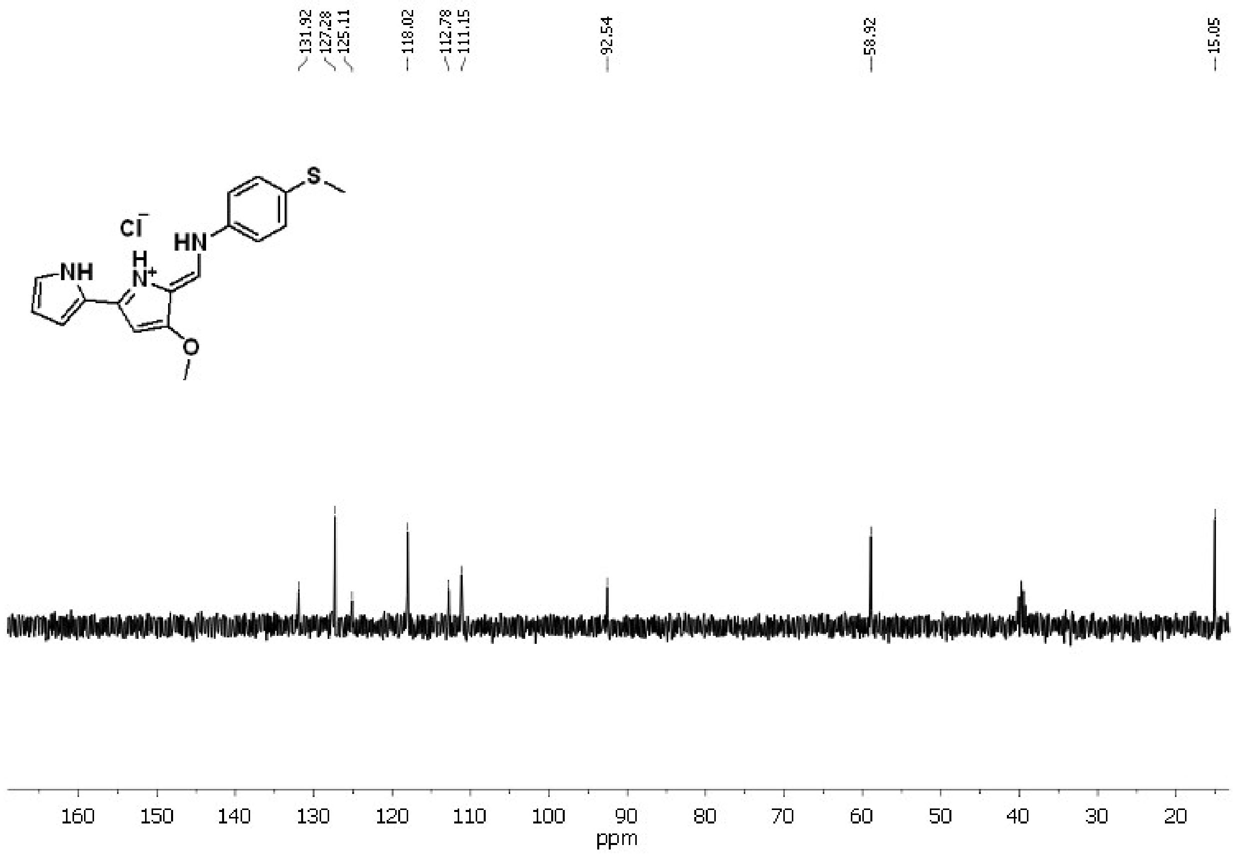

Figure S38. DEPT ^{13}C NMR (DMSO-d_6) of compound **12**. HCl.

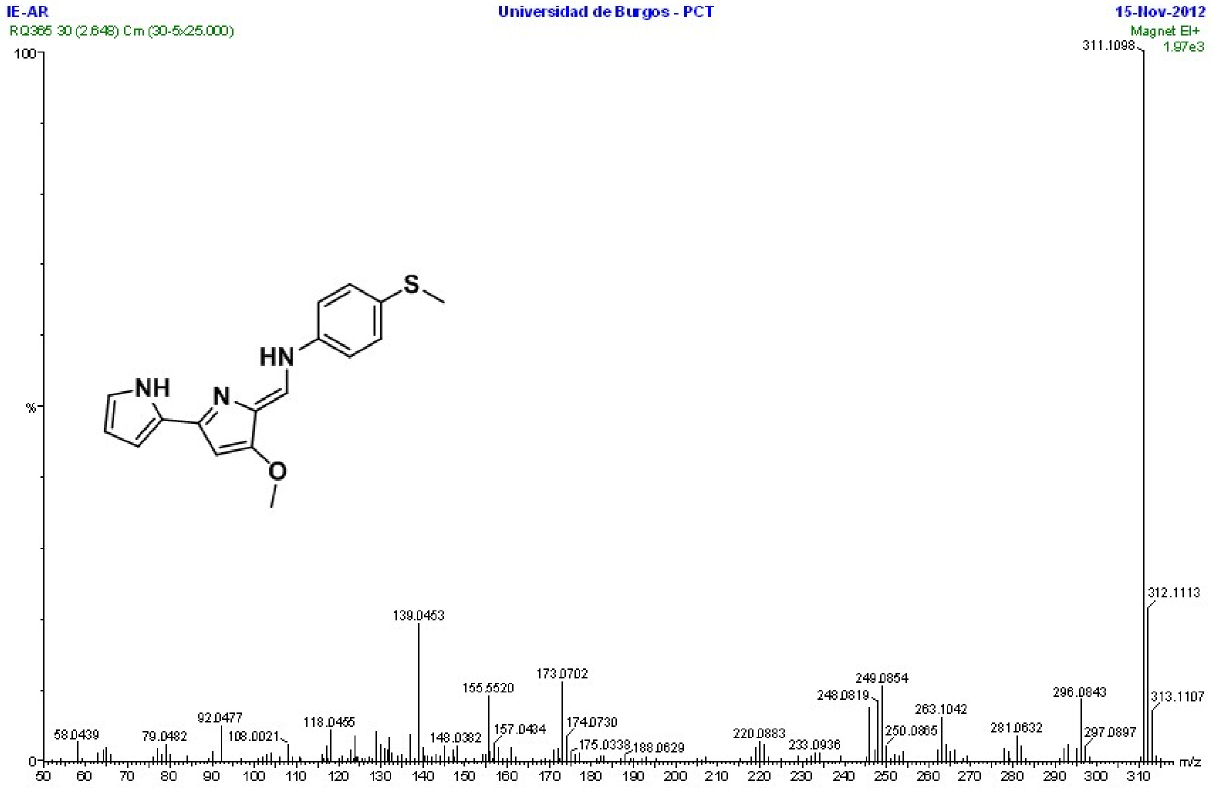

Figure S39. HRMS (EI) of compound **12**.

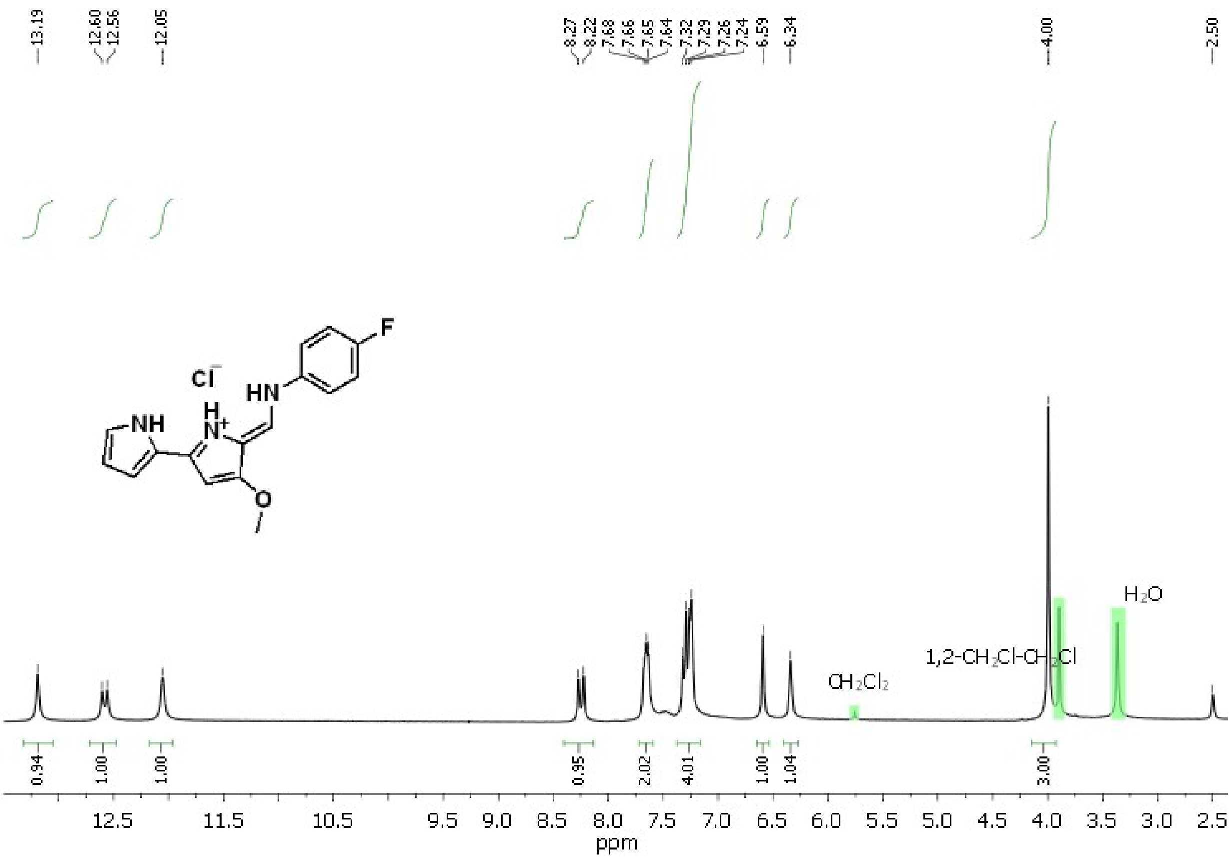

Figure S40. ^{1}H NMR (DMSO-d_6) of compound **13**. HCl.

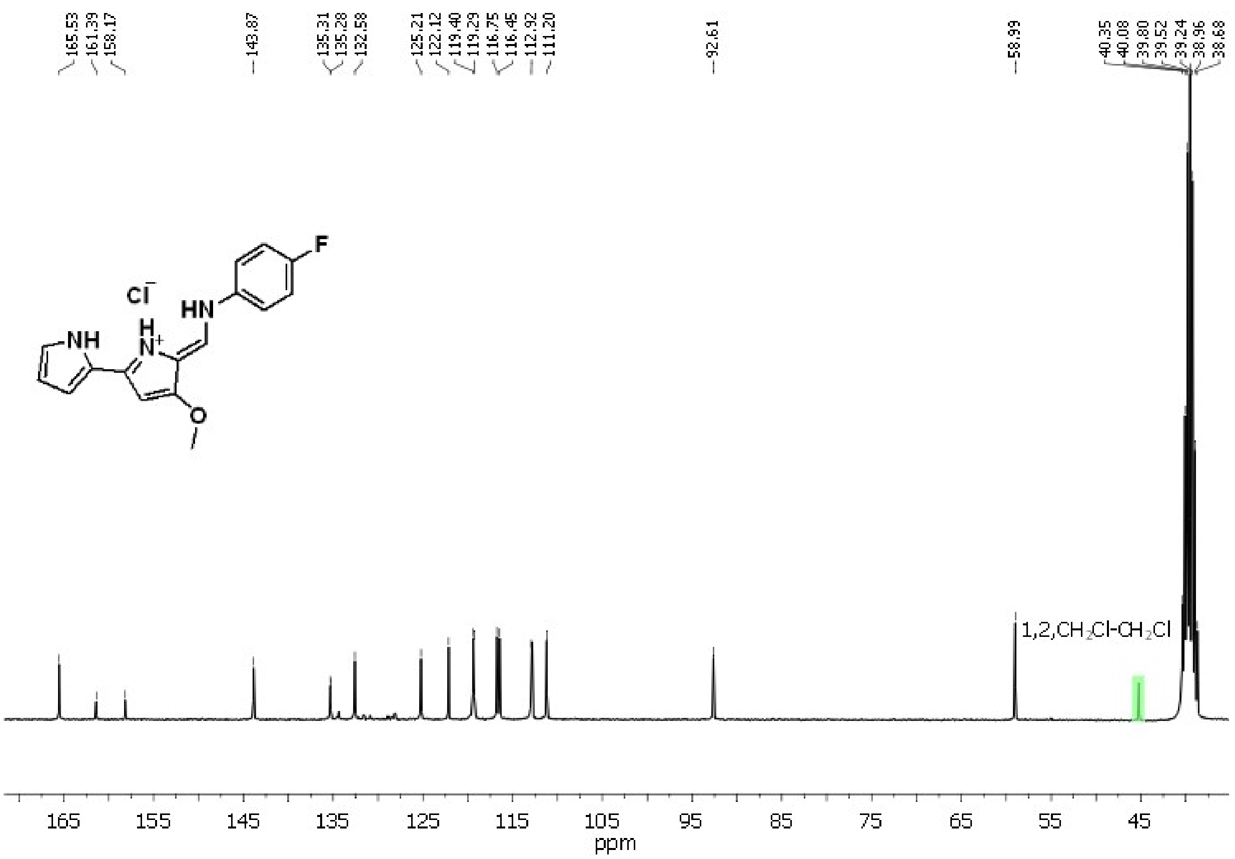

Figure S41. ^{13}C NMR (DMSO-d_6) of compound **13**. HCl.

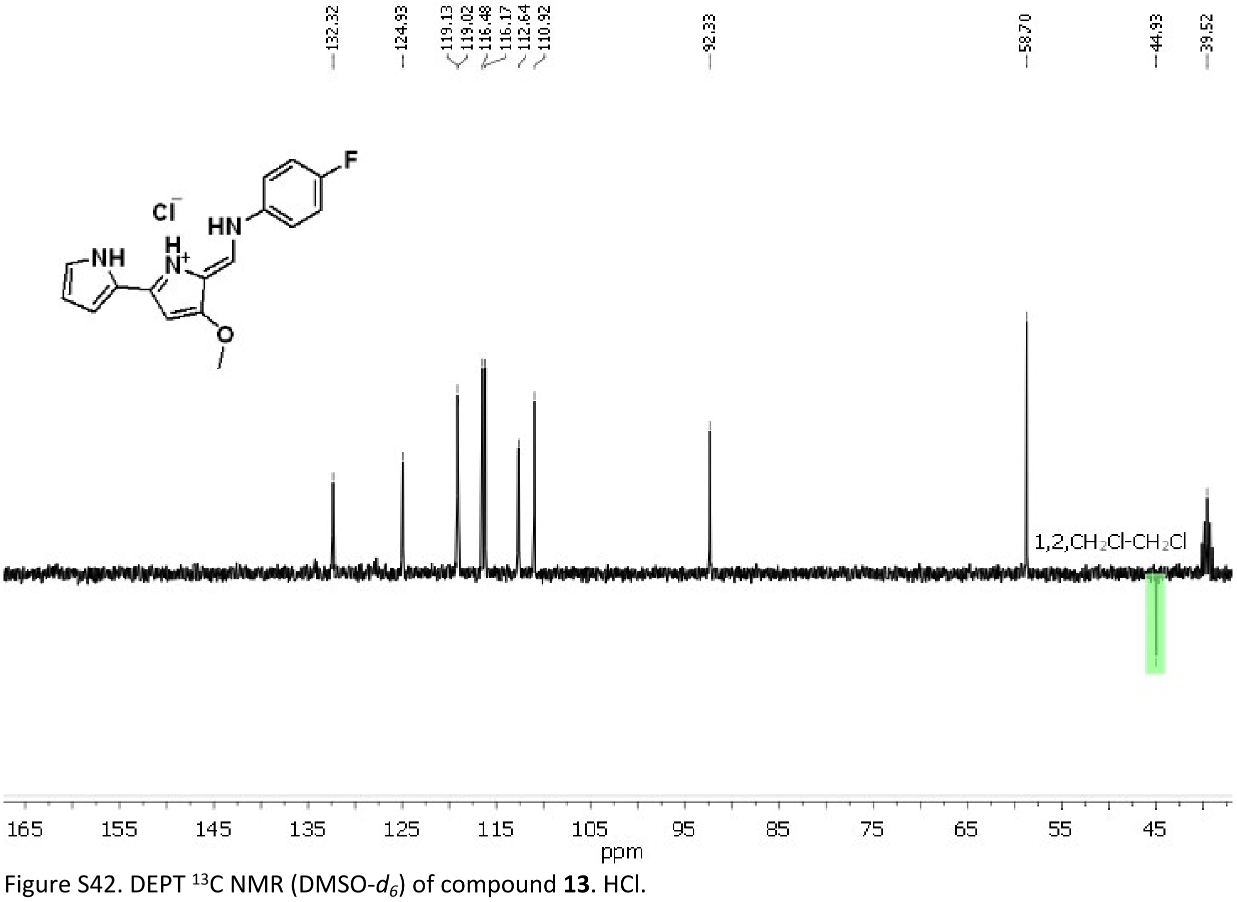

Figure S42. DEPT ^{13}C NMR (DMSO-d_6) of compound **13**. HCl.

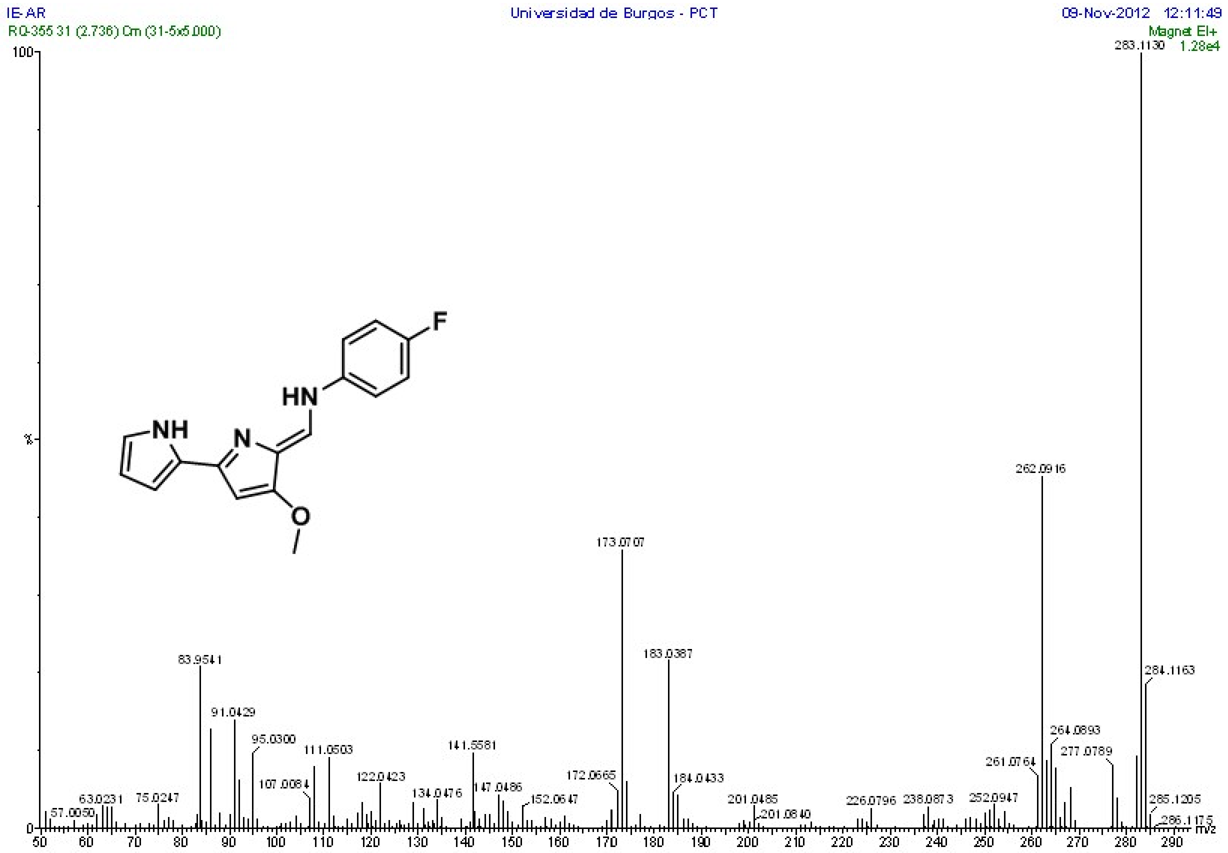

Figure S43. HRMS (EI) of compound **13**.

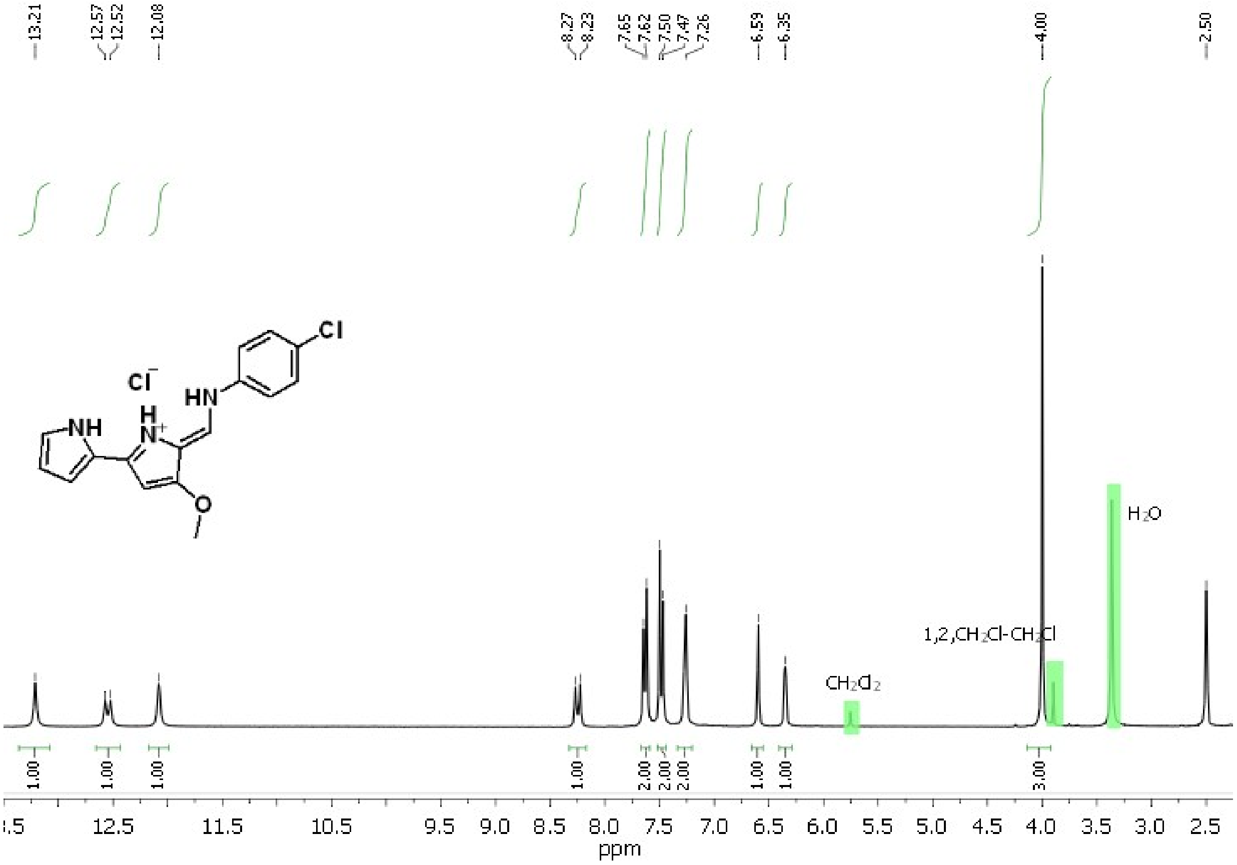

Figure S44. ^{1}H NMR (DMSO-d_6) of compound **14**. HCl.

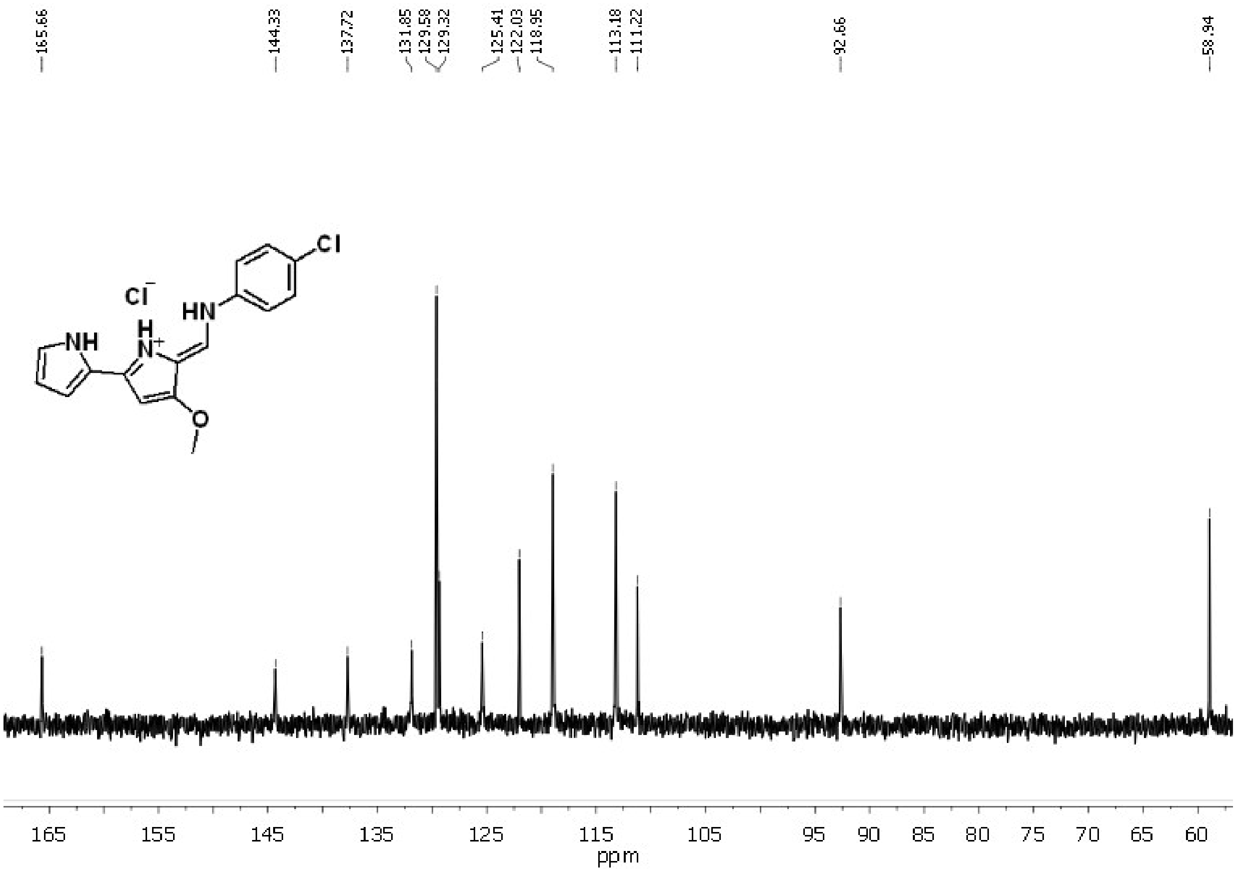

Figure S43. ^{13}C NMR (DMSO-d_6) of compound **14**. HCl.

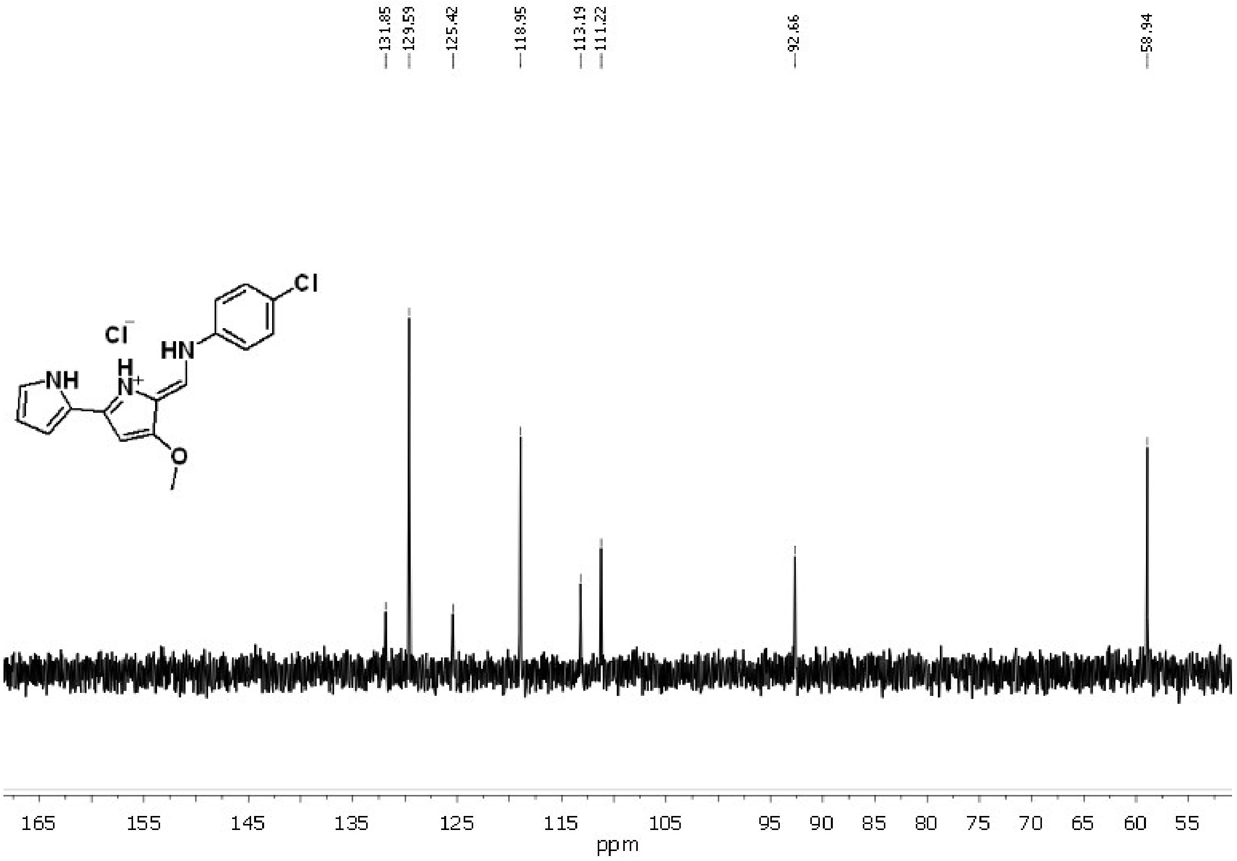

Figure S45. DEPT ^{13}C NMR (DMSO-d_6) of compound **14**. HCl.

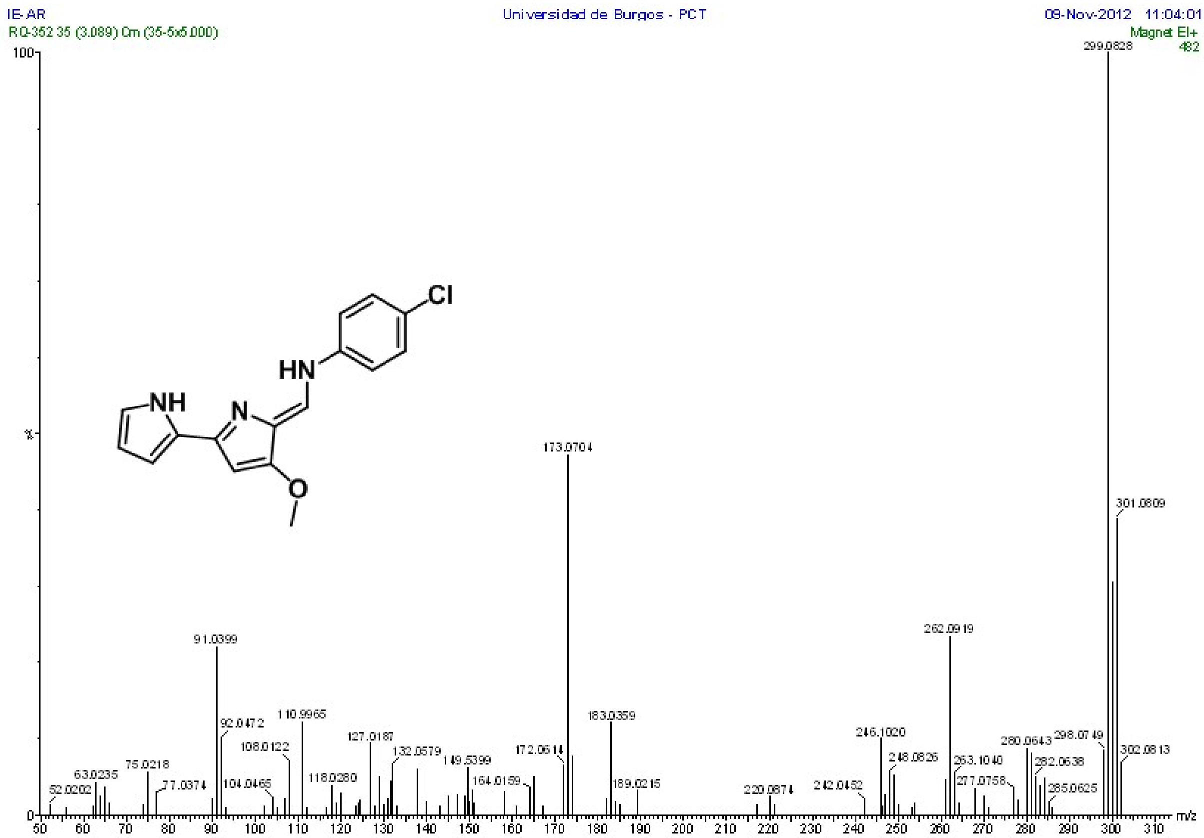

Figure S46. HRMS (EI) of compound **14**.

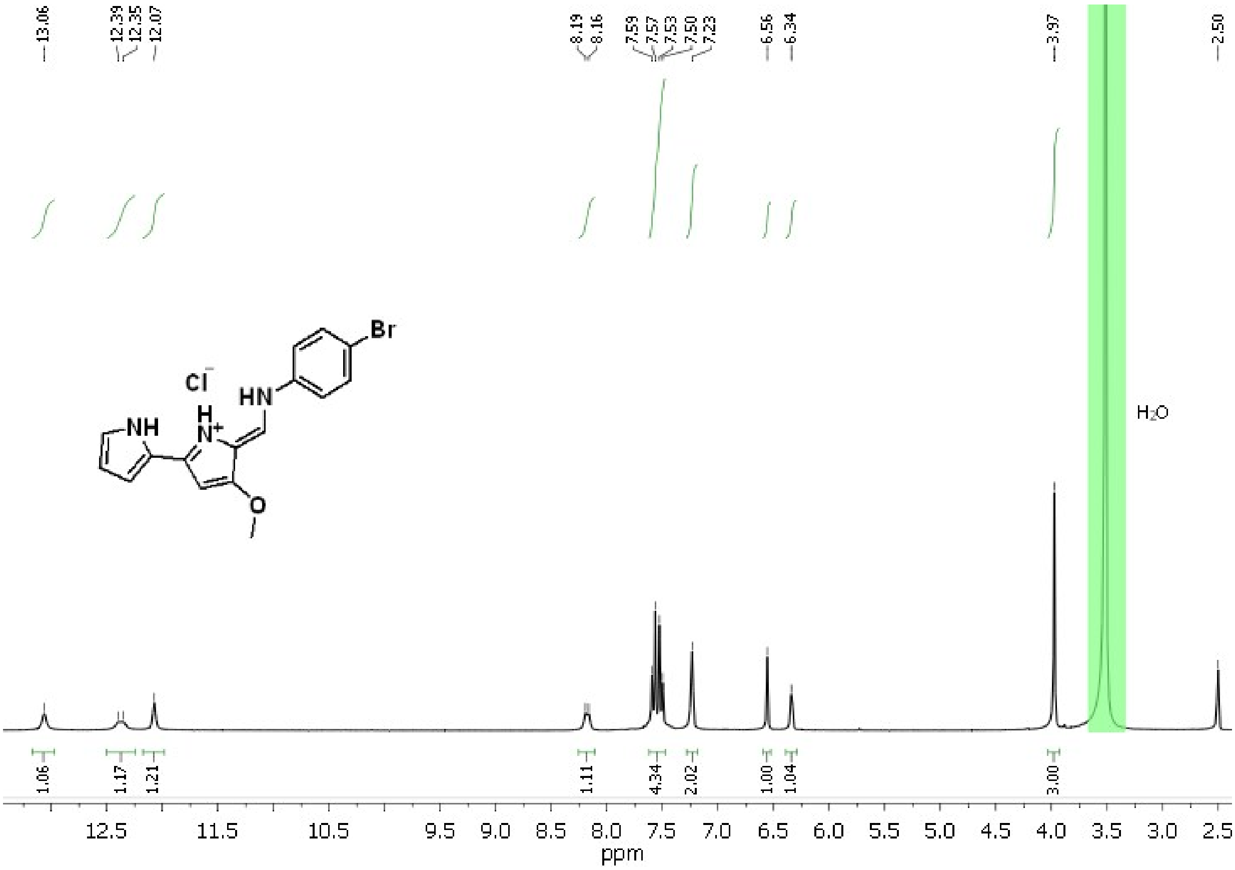

Figure S47. ^{1}H NMR (DMSO-d_6) of compound **15**. HCl.

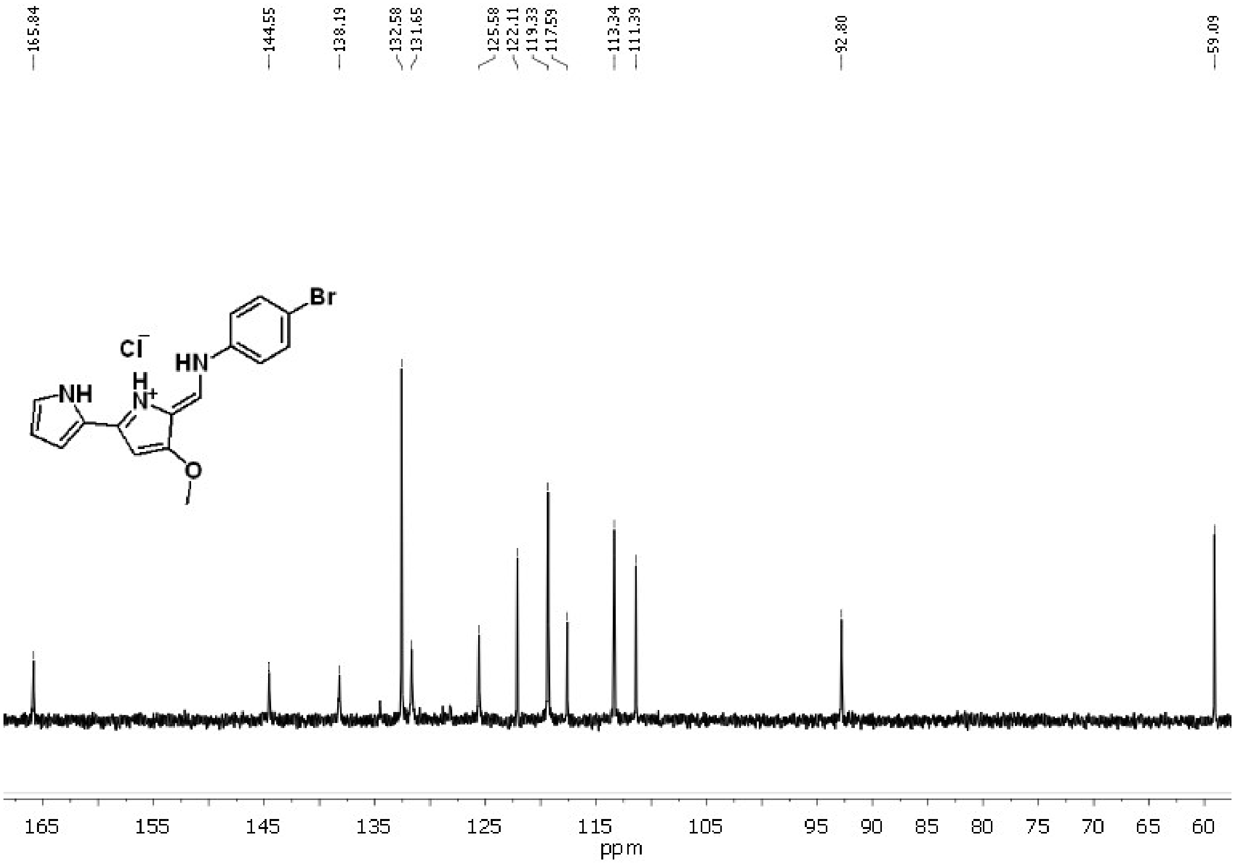

Figure S48. ^{13}C NMR (DMSO-d_6) of compound **15**. HCl.

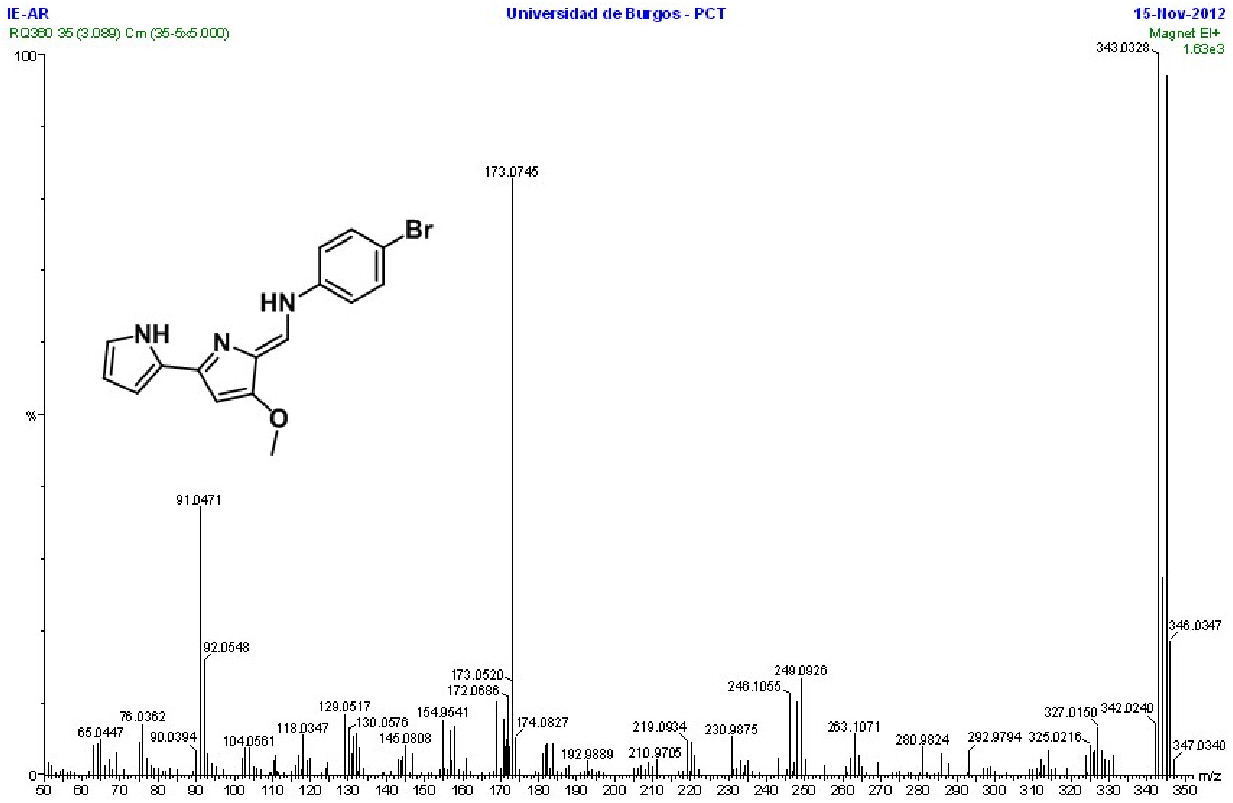

Figure S49. HRMS (EI) of compound **15**.

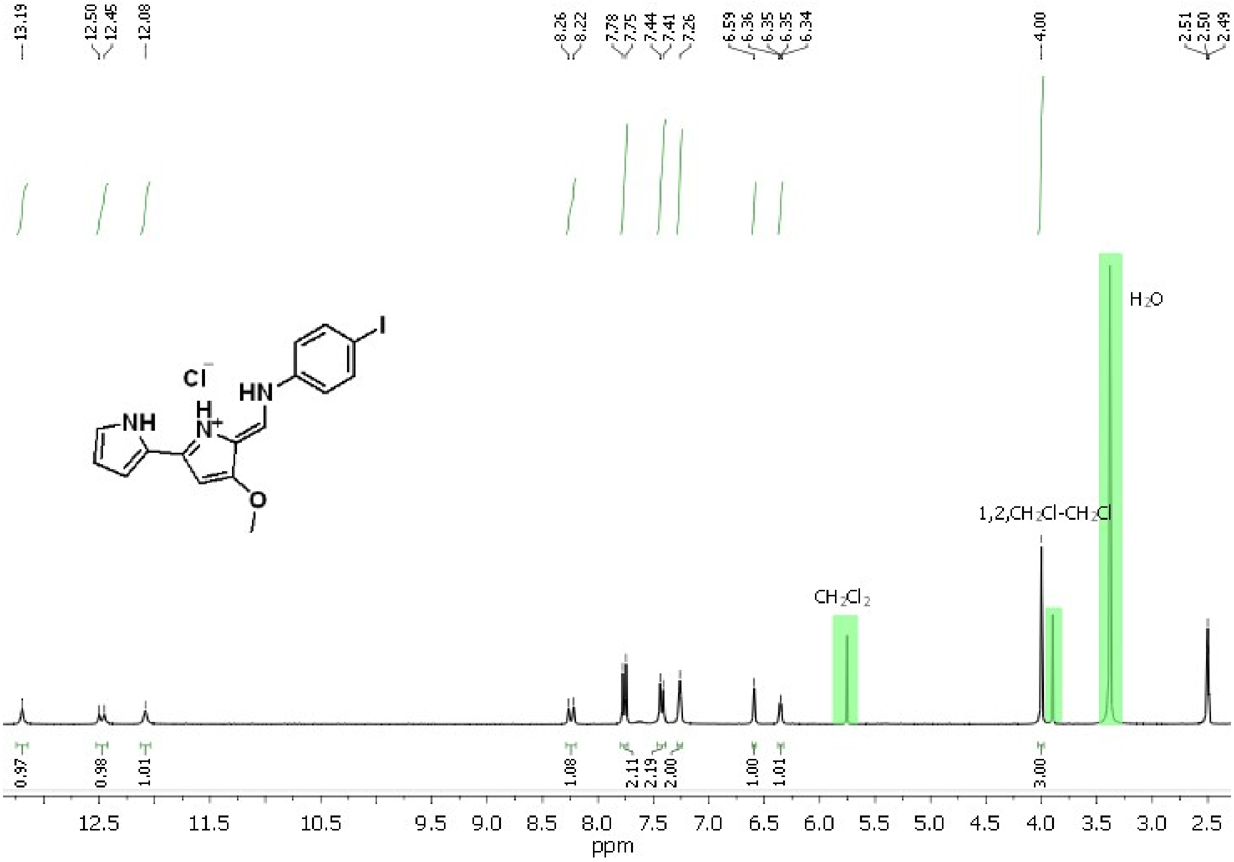

Figure S50. ^{1}H NMR (DMSO-d_6) of compound **16**. HCl.

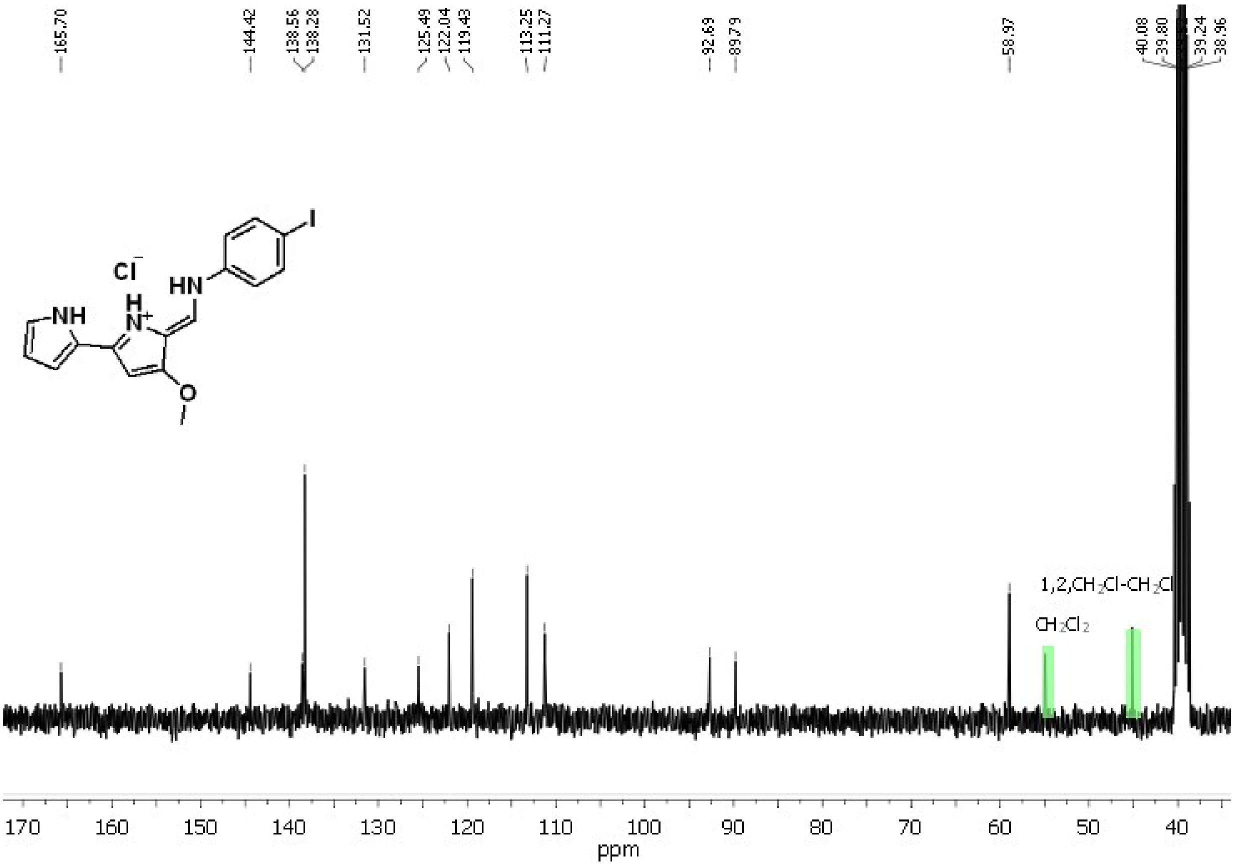

Figure S51. ^{13}C NMR (DMSO-d_6) of compound **16**. HCl.

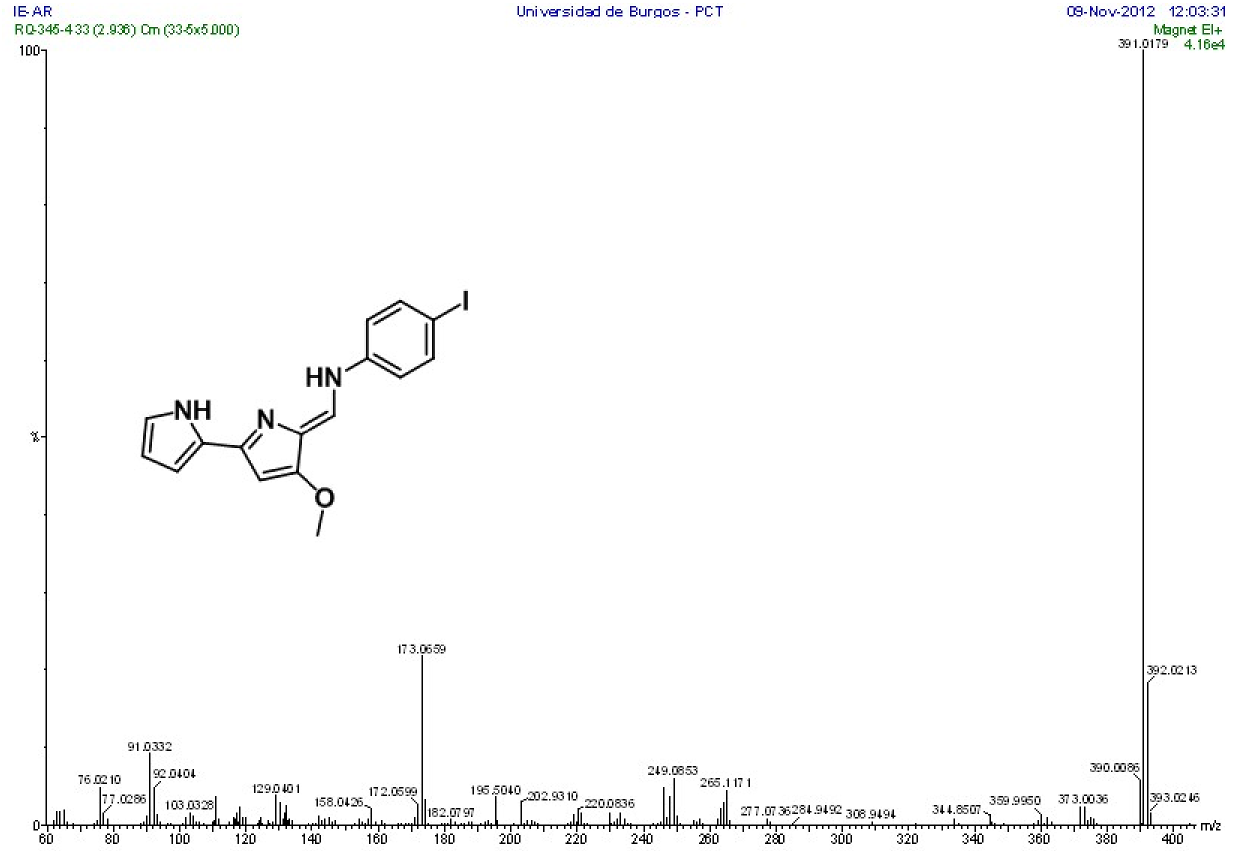

Figure S52. HRMS (EI) of compound **16**.

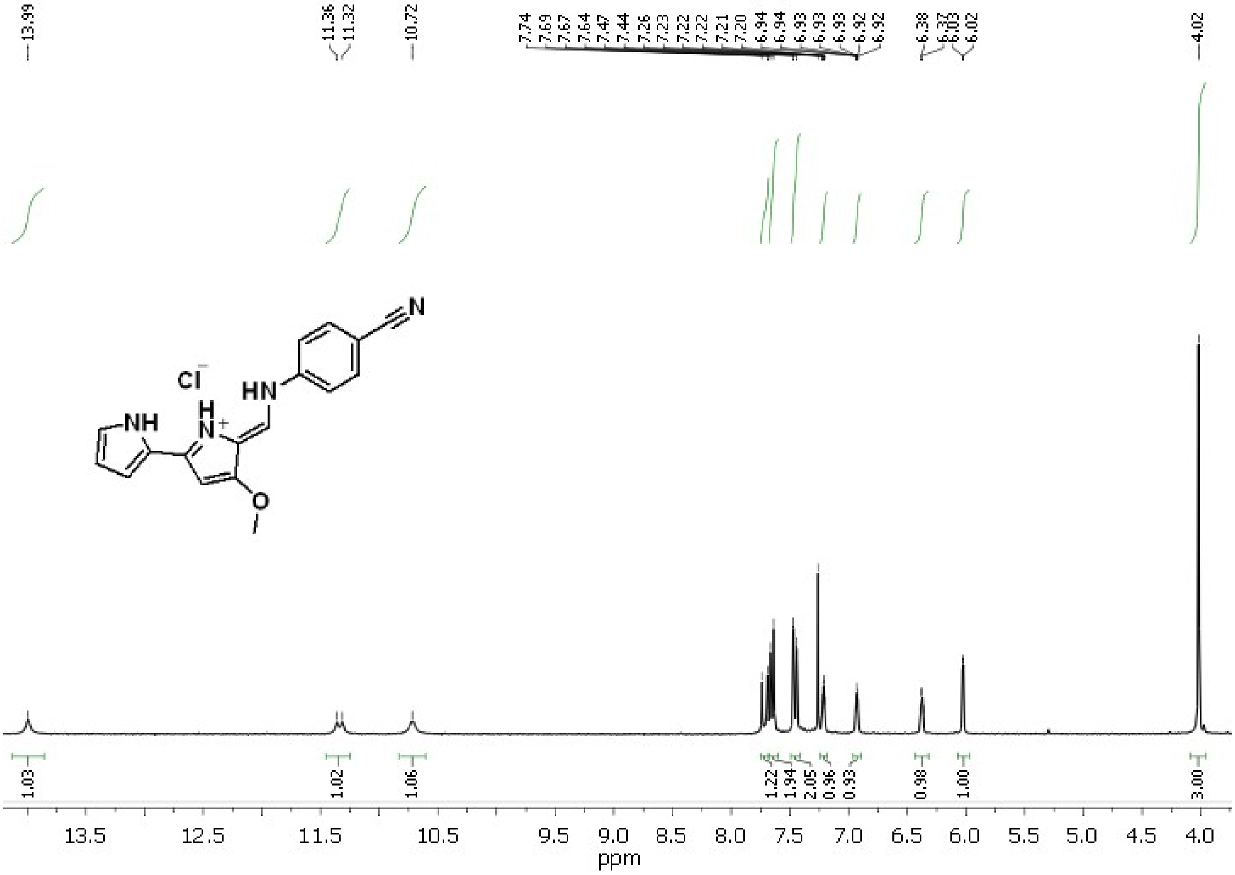

Figure S53. ^{1}H NMR ($CDCl_3$) of compound **17**. HCl.

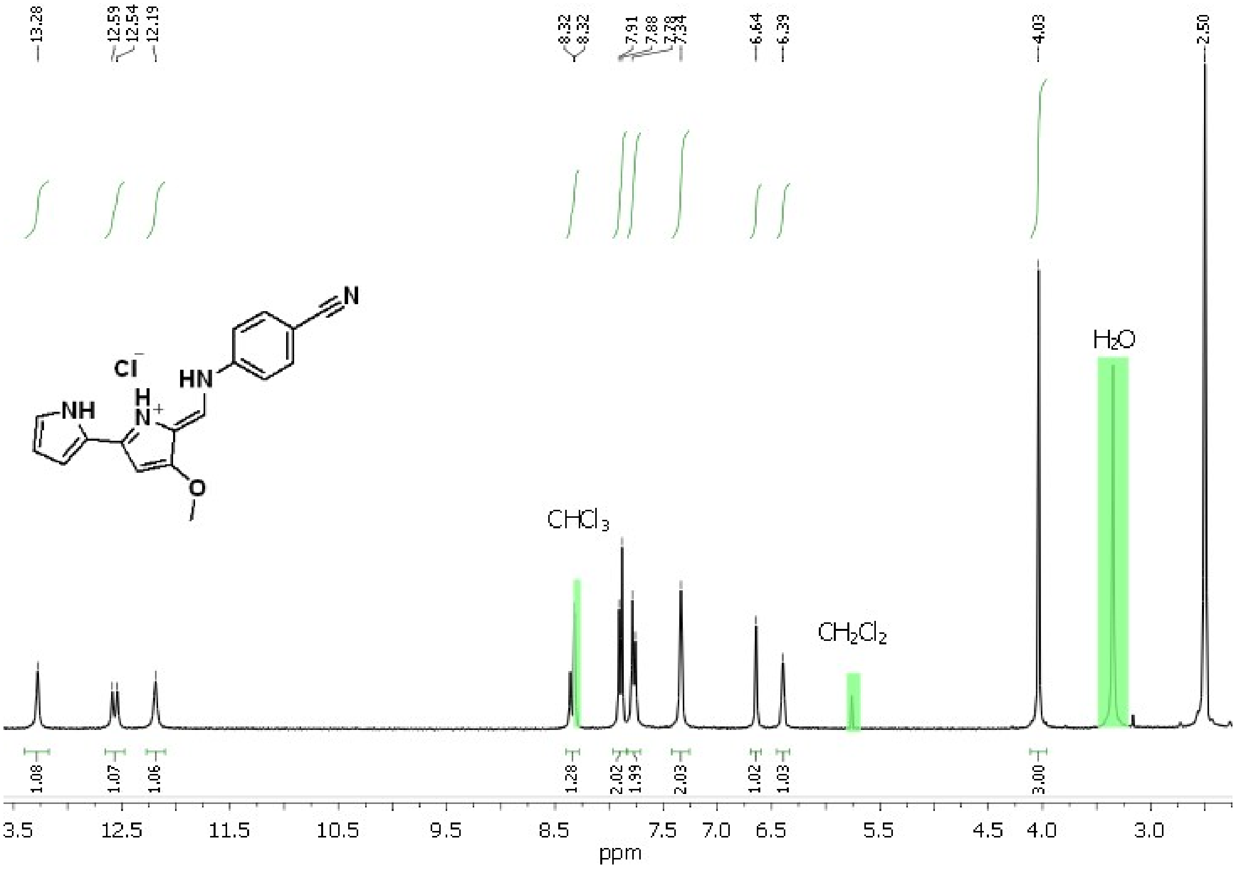

Figure S54. ^{1}H NMR (DMSO-d_6) of compound **17**. HCl.

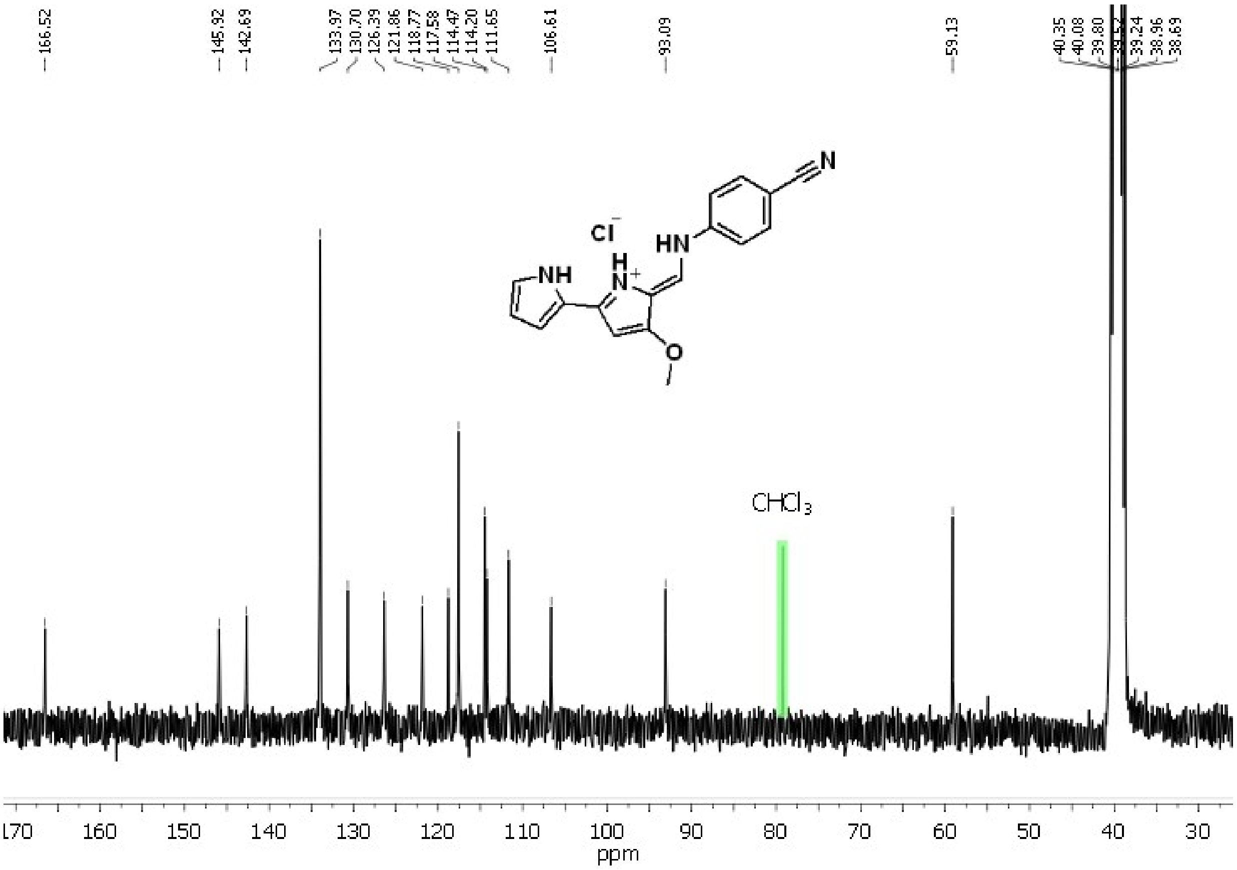

Figure S55. ^{13}C NMR (DMSO-d_6) of compound **17**. HCl.

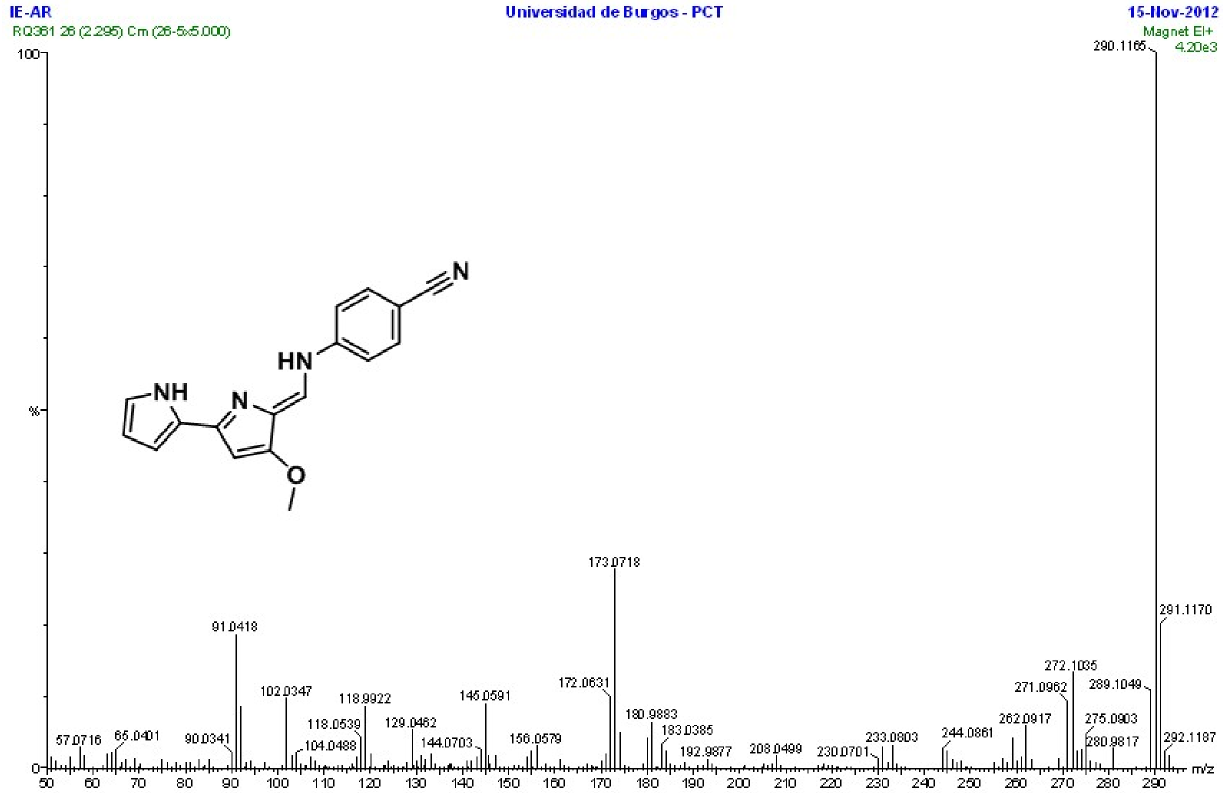

Figure S56. HRMS (EI) of compound **17**.

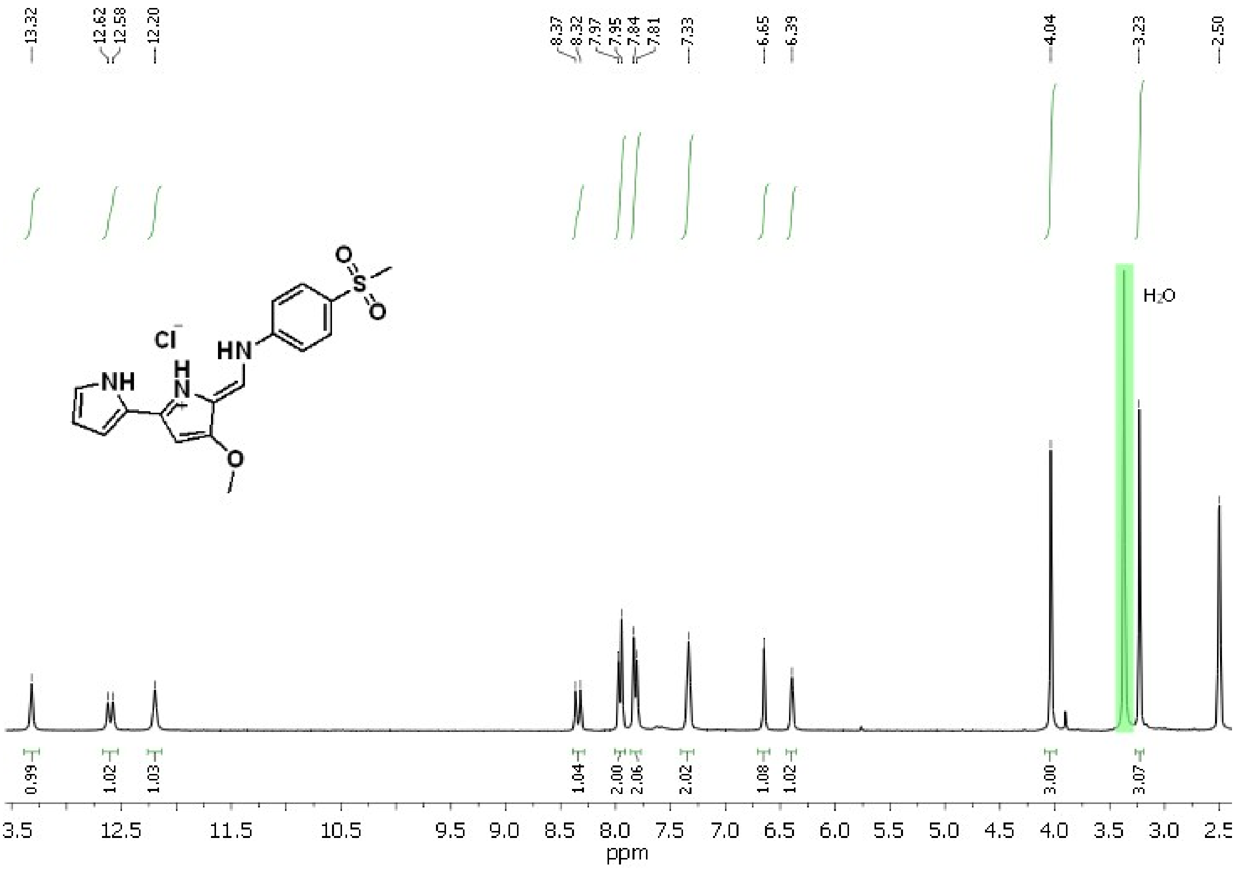

Figure S57. ^{1}H NMR (DMSO-d_6) of compound **18**. HCl.

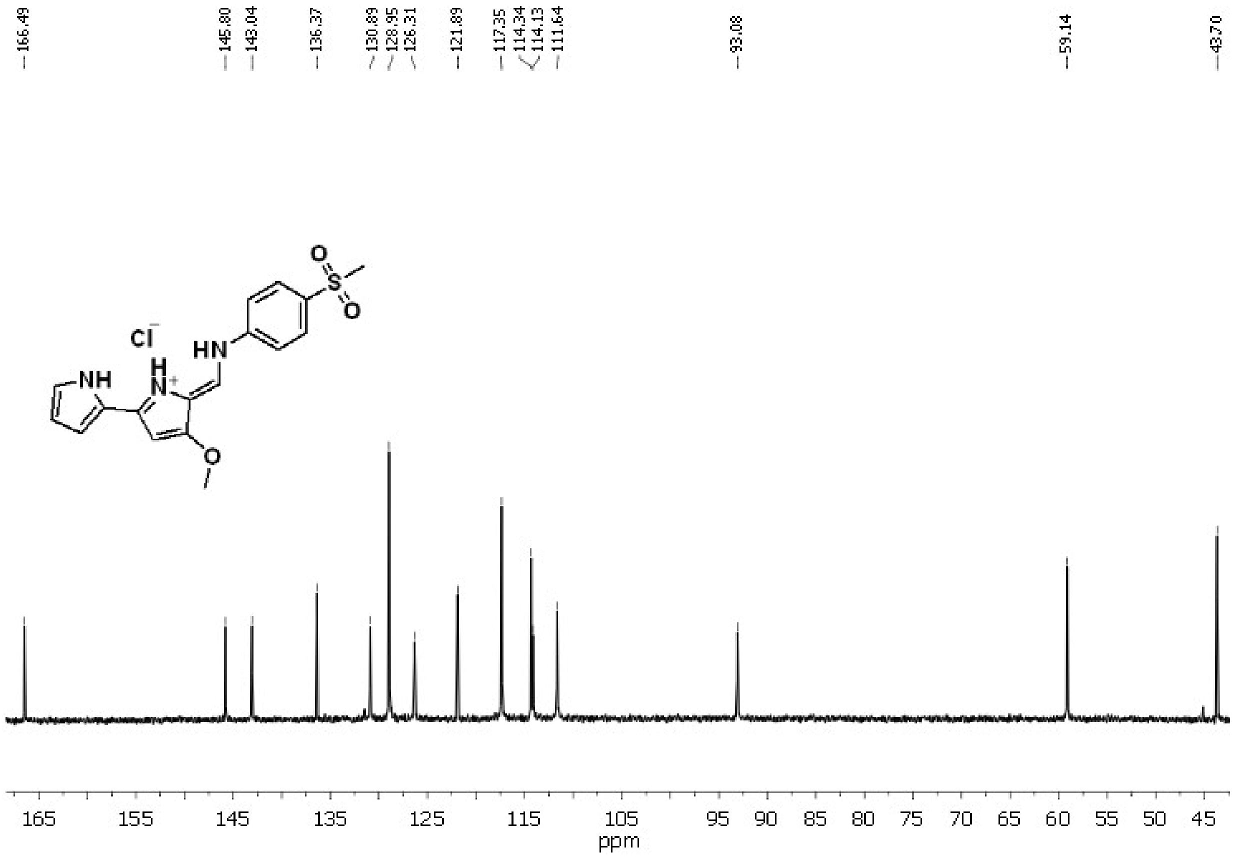

Figure S58. ^{13}C NMR (DMSO-d_6) of compound **18**. HCl.

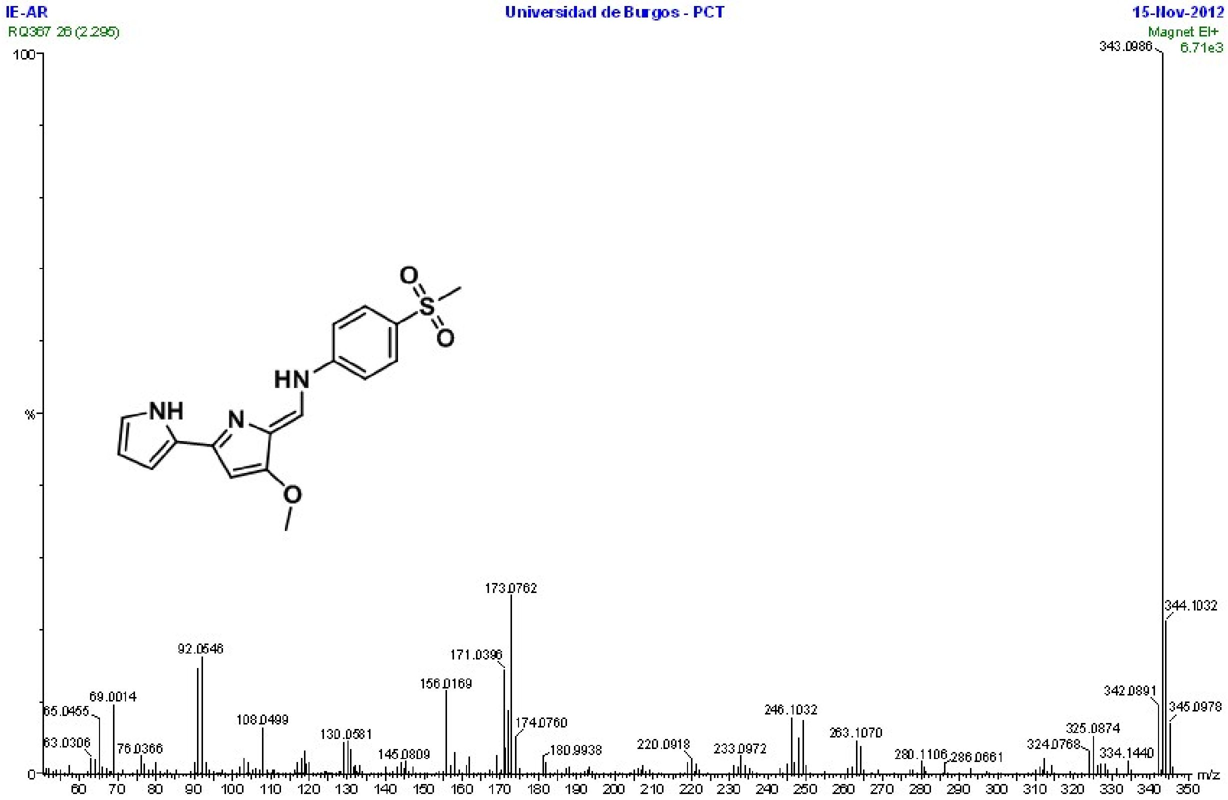

Figure S59. HRMS (EI) of compound **18**.

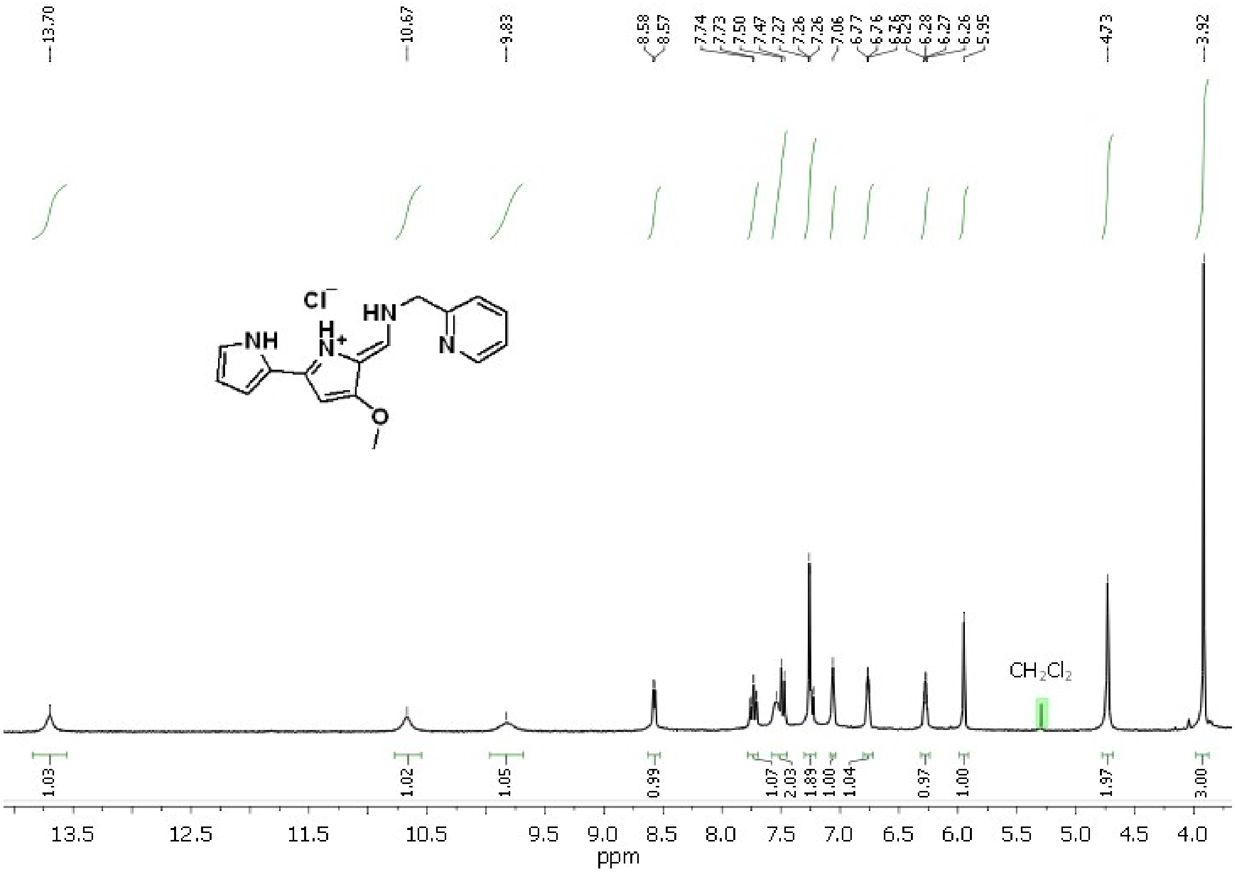

Figure S60. ^{1}H NMR ($CDCl_3$) of compound **19**. HCl.

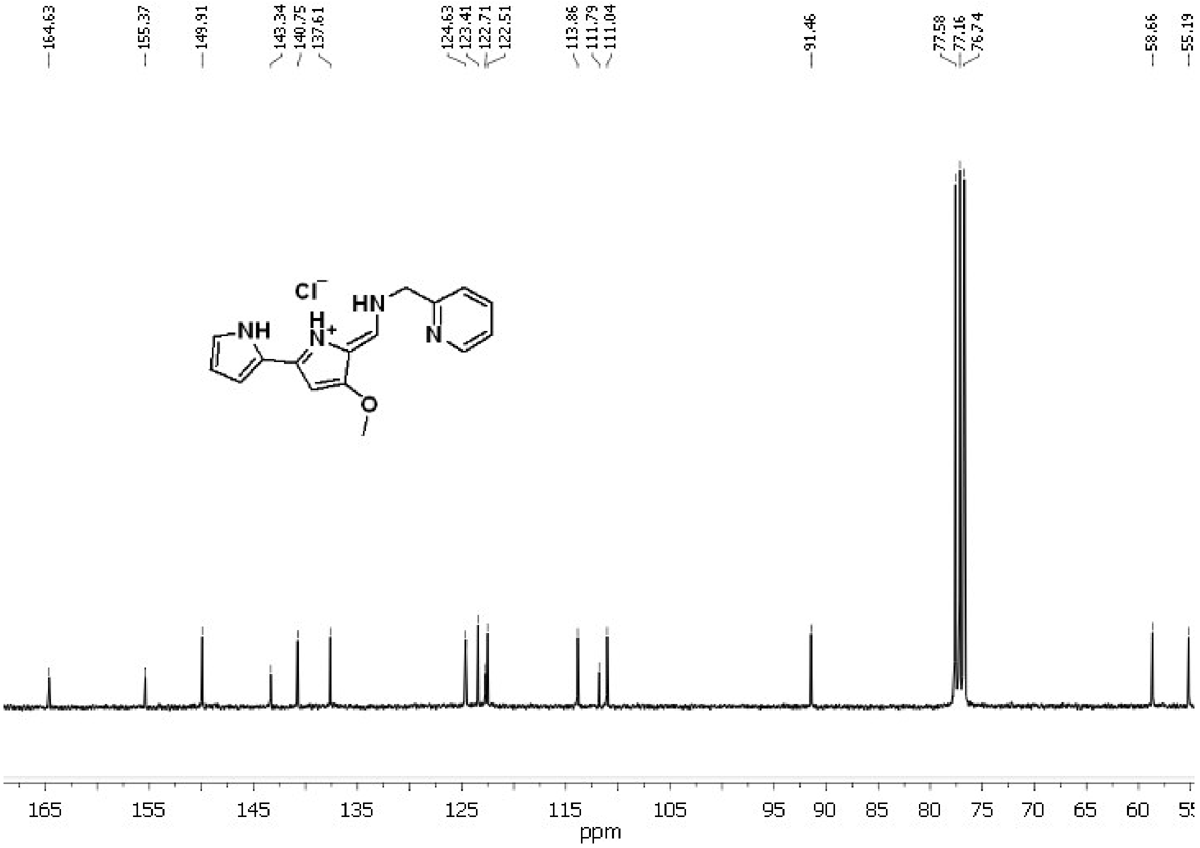

Figure S61. ^{13}C NMR ($CDCl_3$) of compound **19**. HCl.

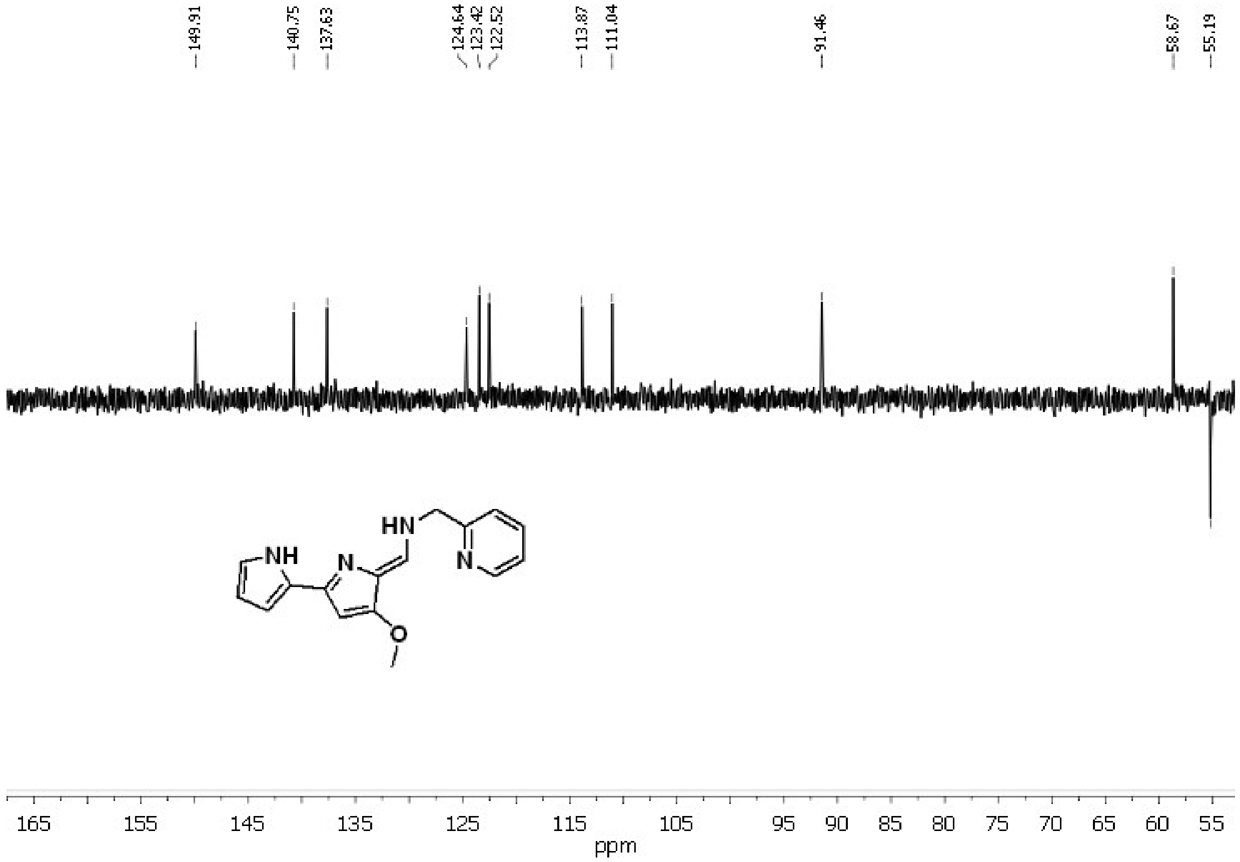

Figure S62. DEPT ^{13}C NMR ($CDCl_3$) of compound **19**. HCl.

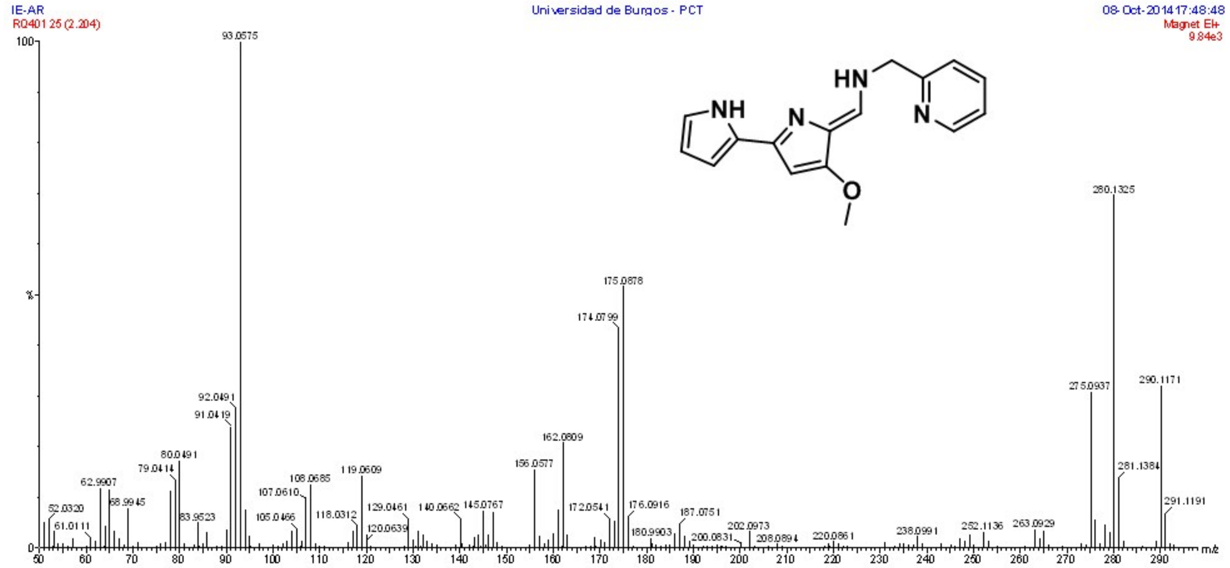

Figure S63. HRMS (EI) of compound **19**.

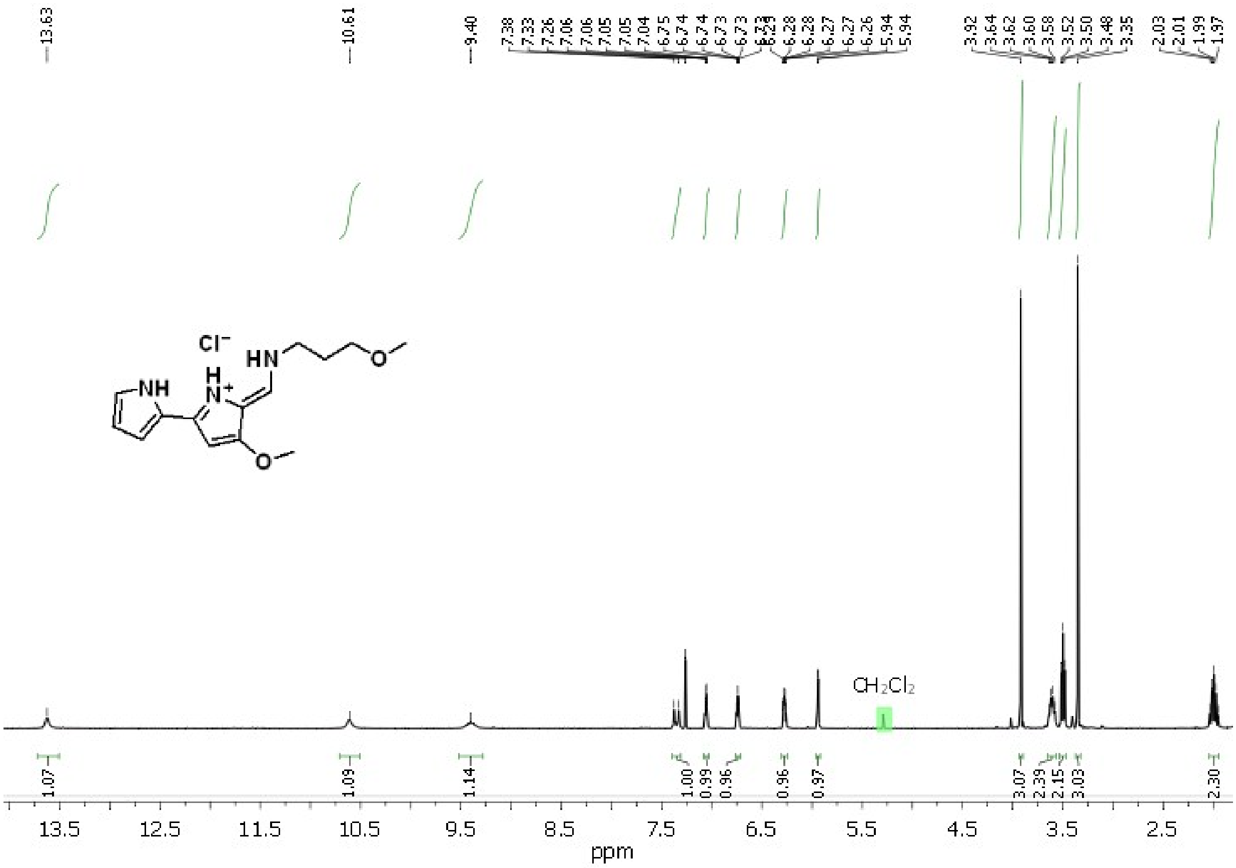

Figure S64. ^{1}H NMR ($CDCl_3$) of compound **33**. HCl.

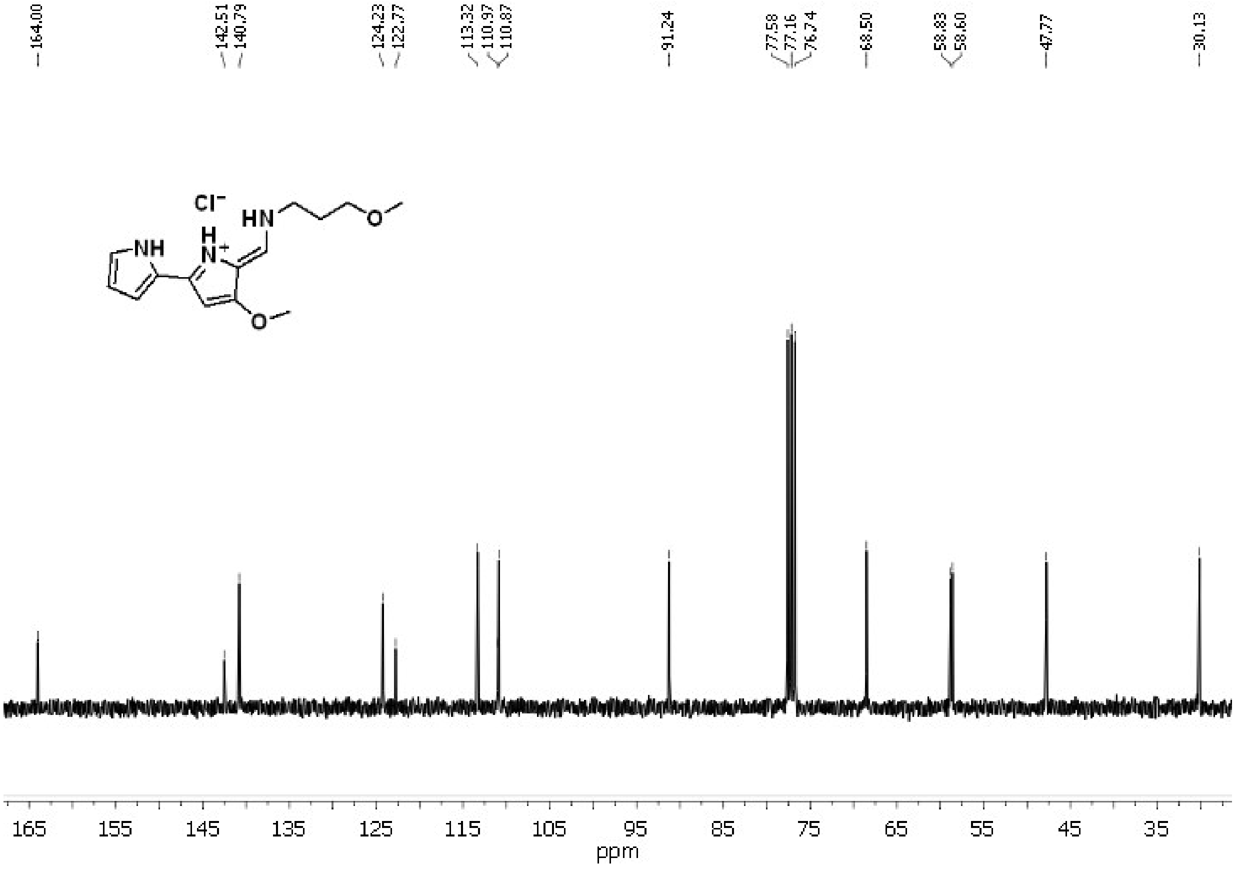

Figure S65. ^{13}C NMR ($CDCl_3$) of compound **33**. HCl.

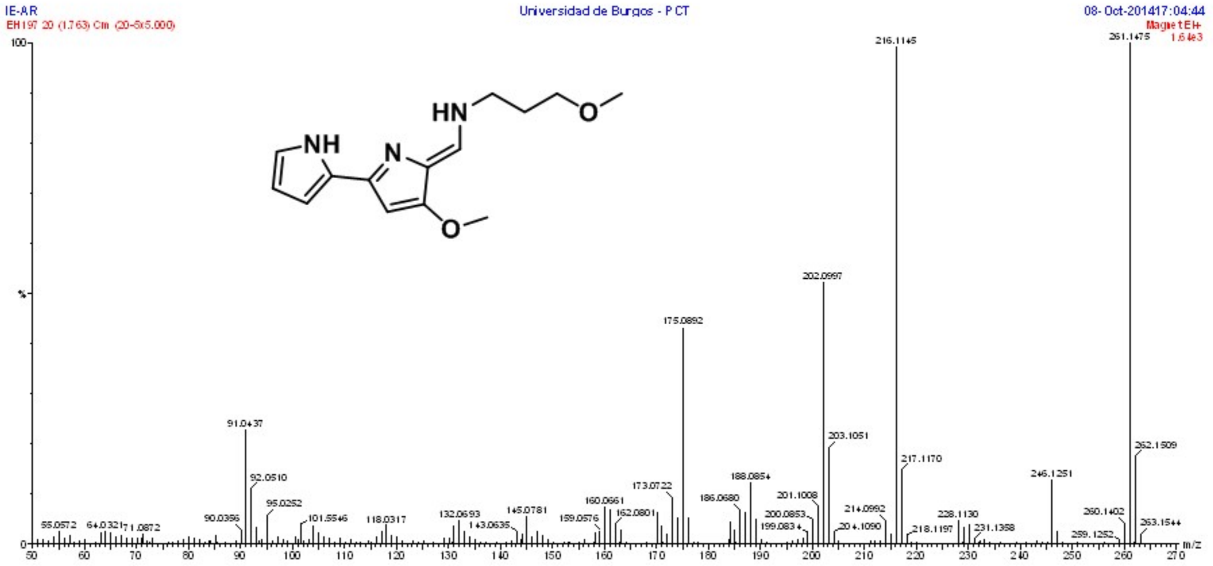

Figure S66. HRMS (EI) of compound **33**.

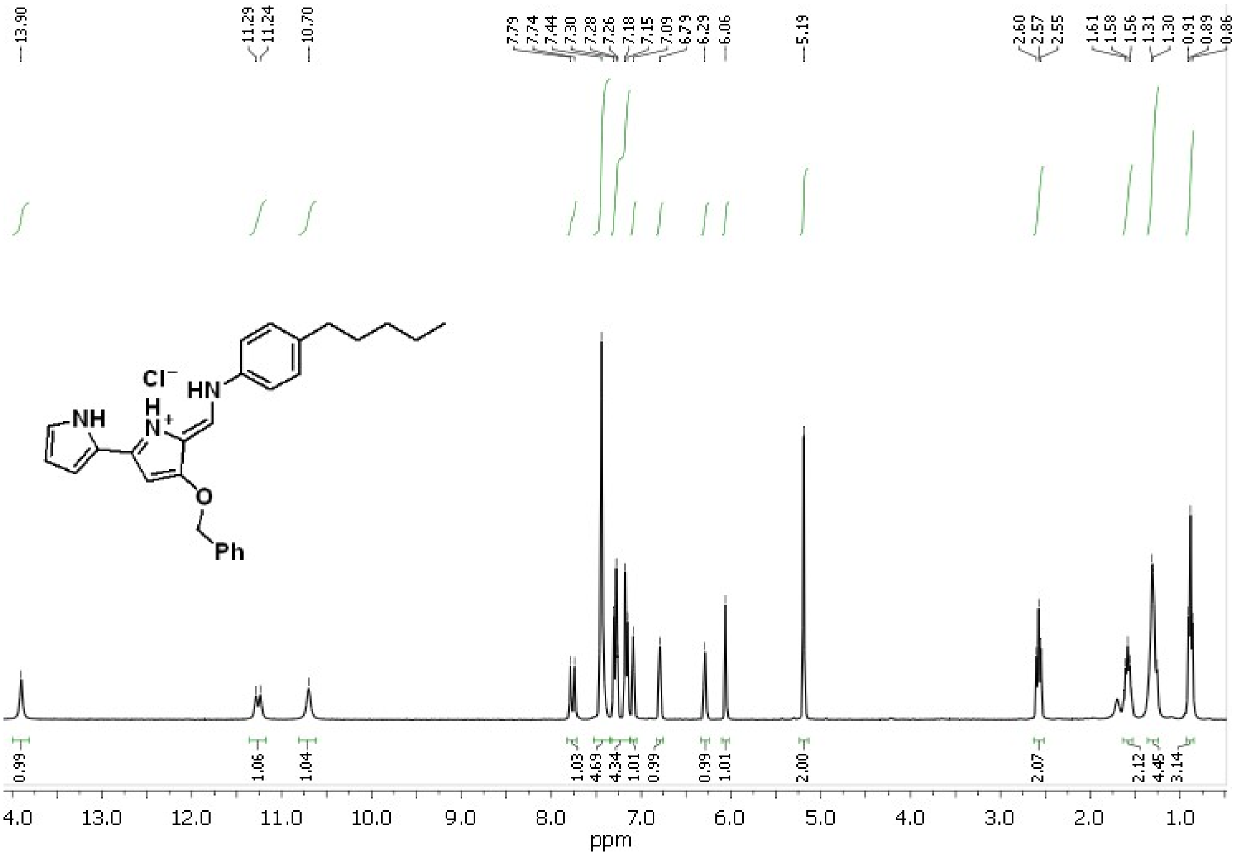

Figure S67. ^{1}H NMR ($CDCl_3$) of compound **36**. HCl.

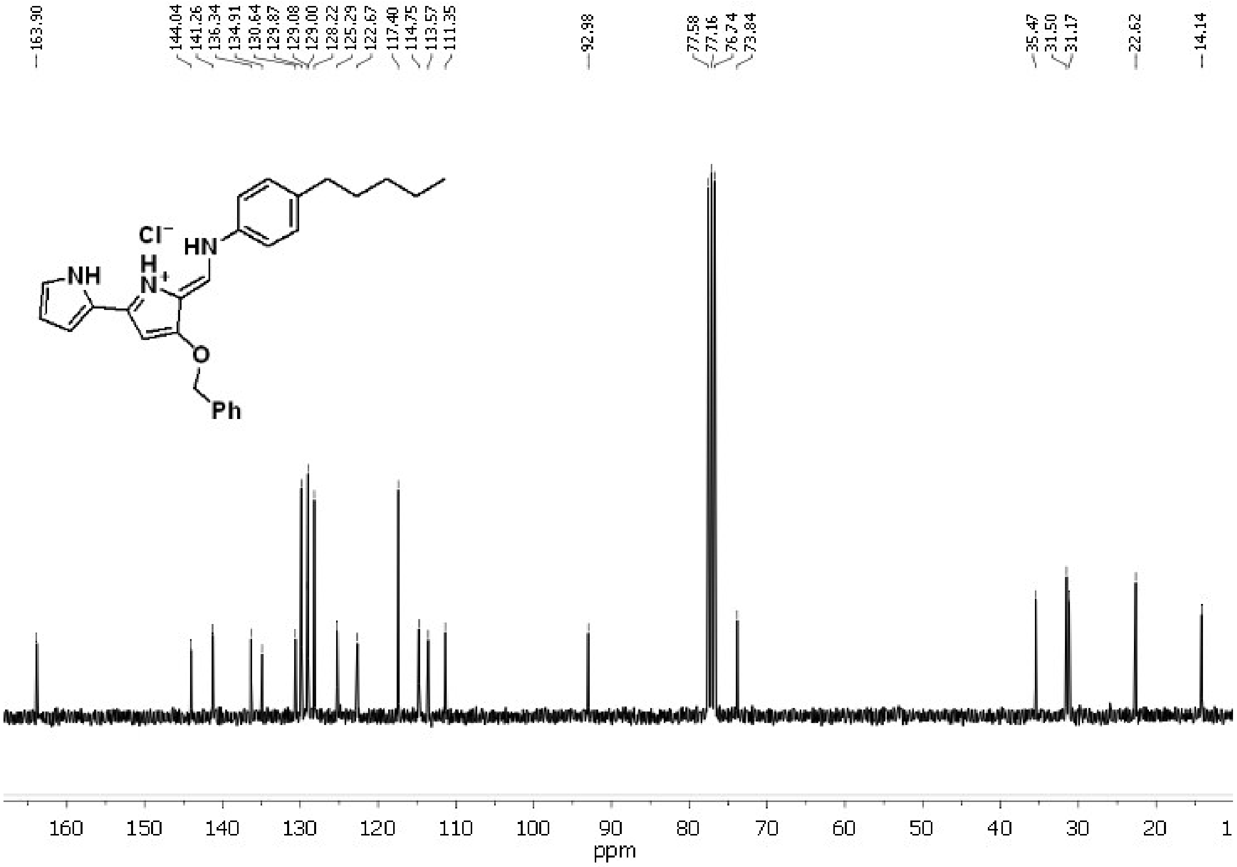

Figure S68. ^{13}C NMR ($CDCl_3$) of compound **36**. HCl.

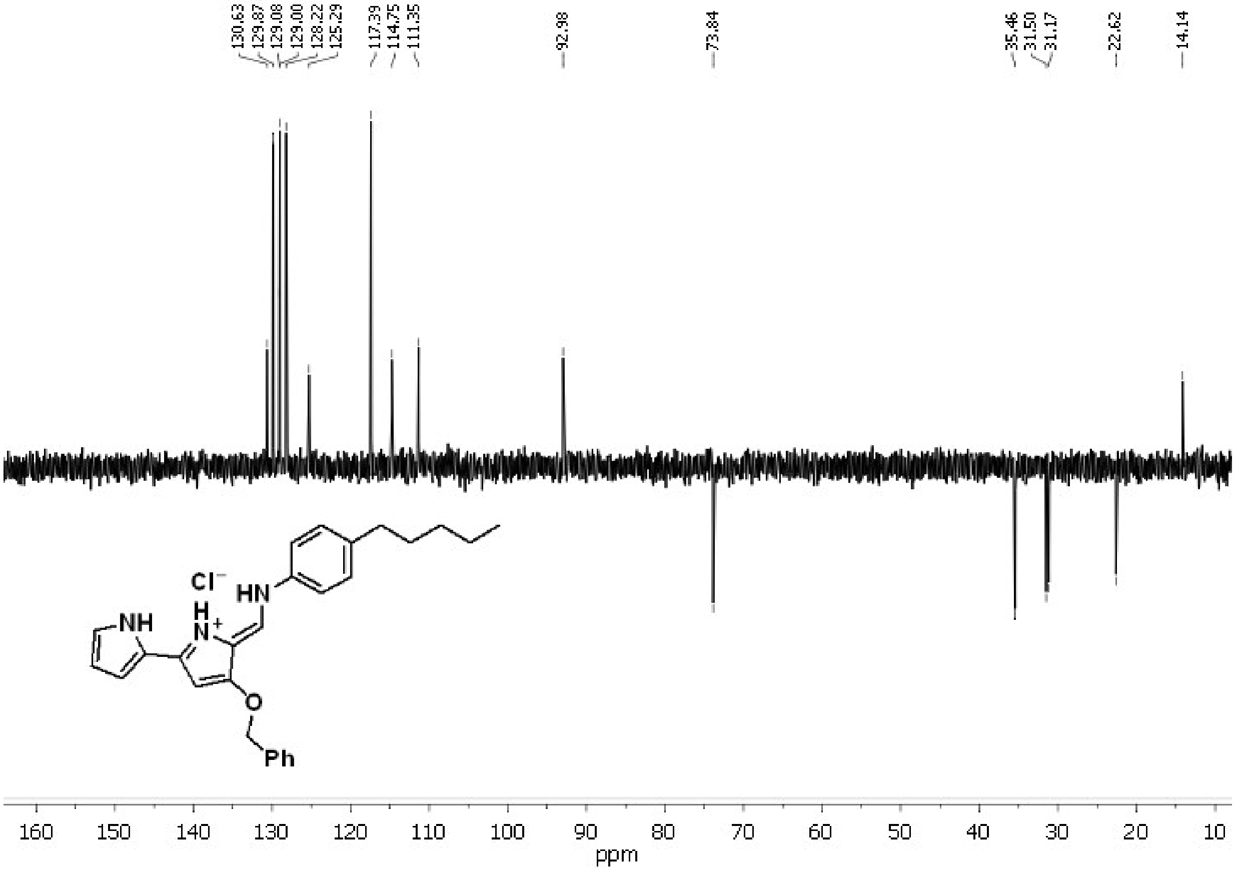

Figure S69. DEPT ^{13}C NMR ($CDCl_3$) of compound **36**. HCl.

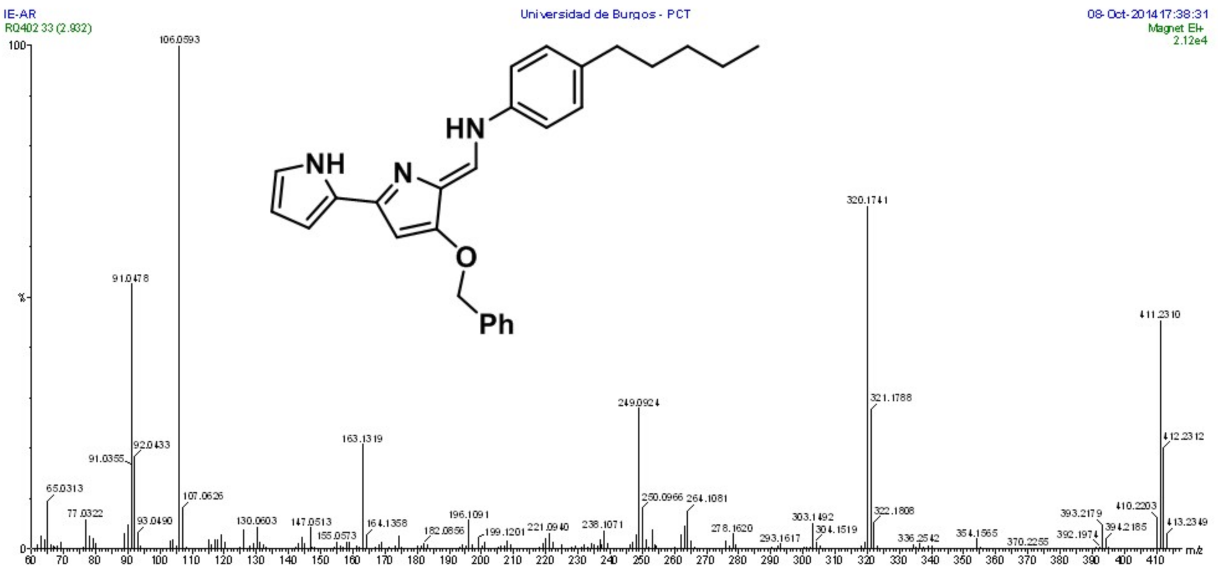

Figure S70. HRMS (EI) of compound **36**.

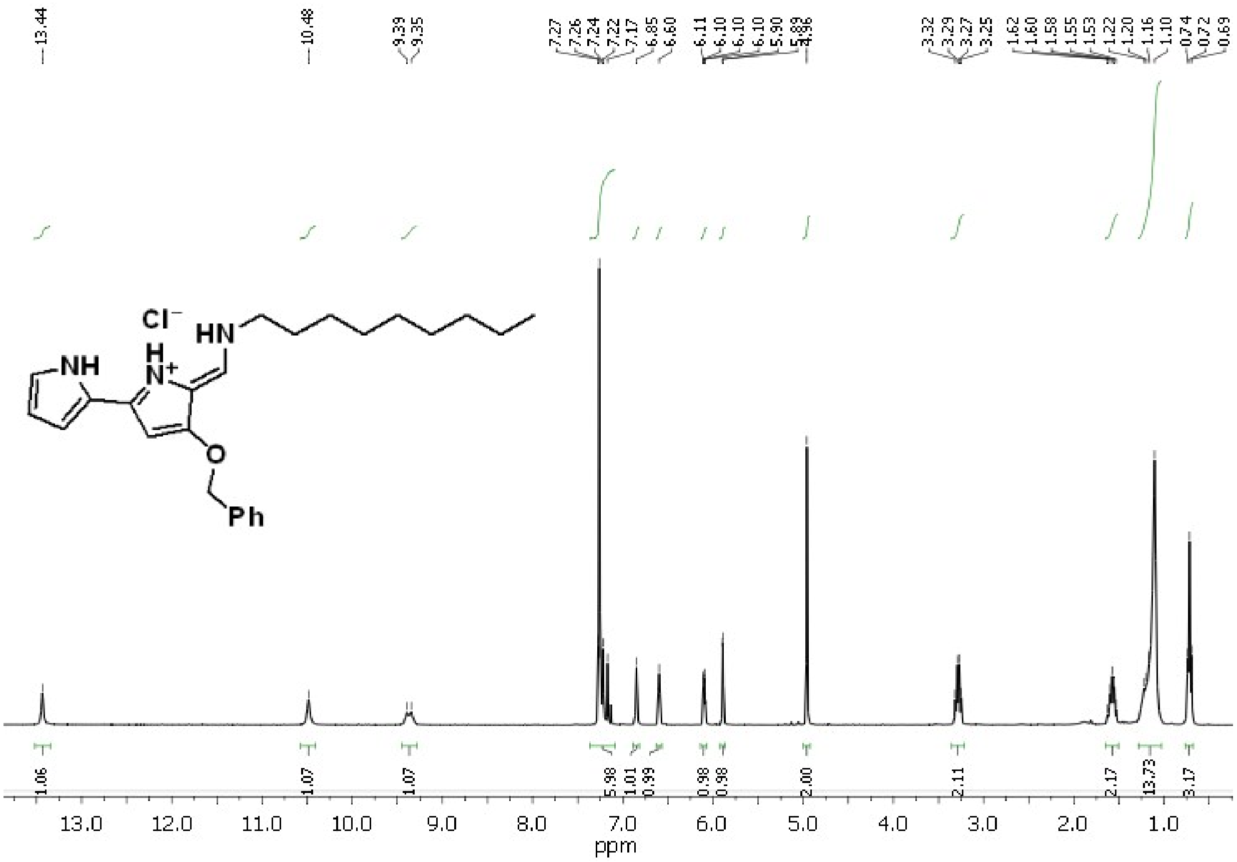

Figure S71. ^{1}H NMR ($CDCl_3$) of compound **41**. HCl.

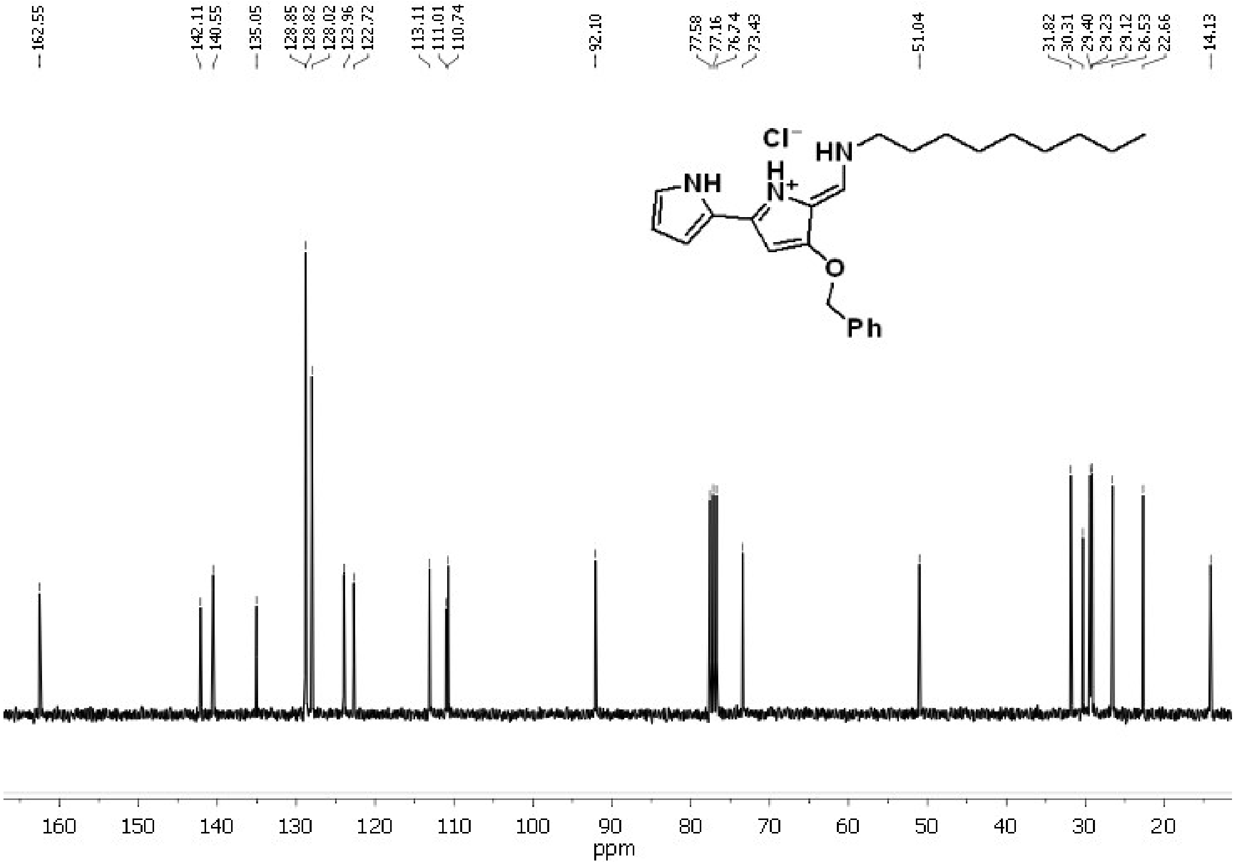

Figure S72. ^{13}C NMR ($CDCl_3$) of compound **41**. HCl.

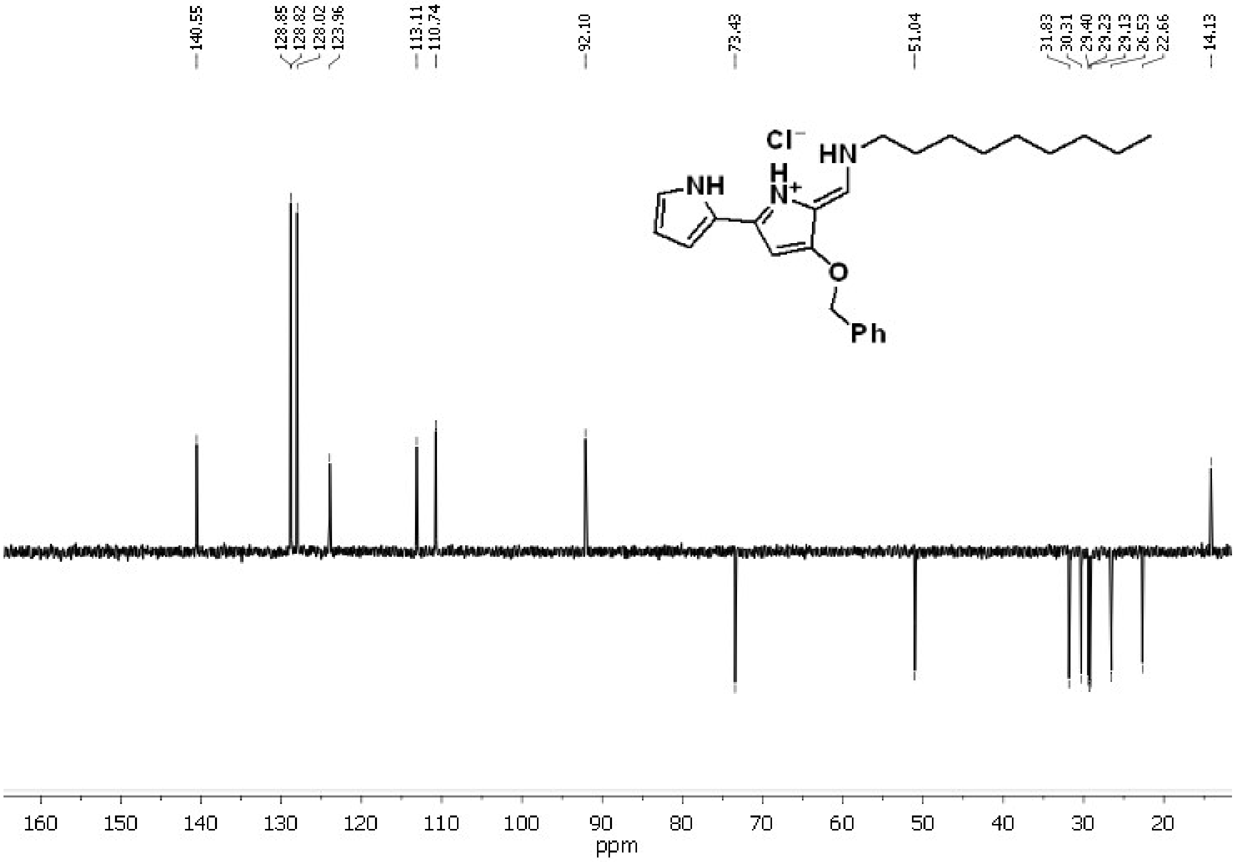

Figure S73. DEPT ^{13}C NMR ($CDCl_3$) of compound **41**. HCl.

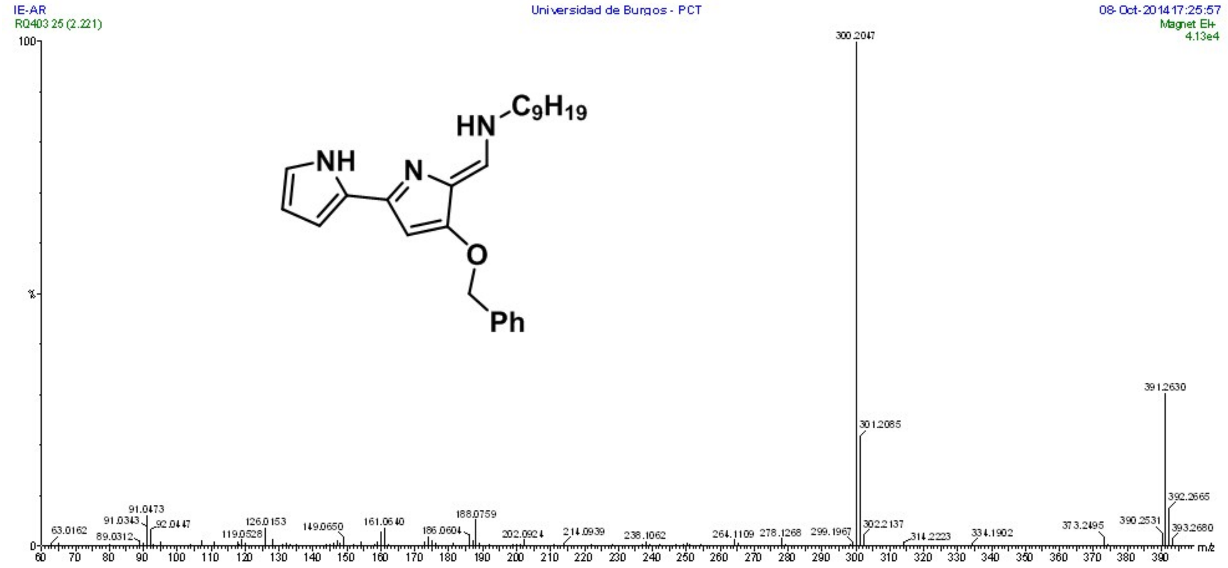

Figure S74. HRMS (EI) of compound **41**.

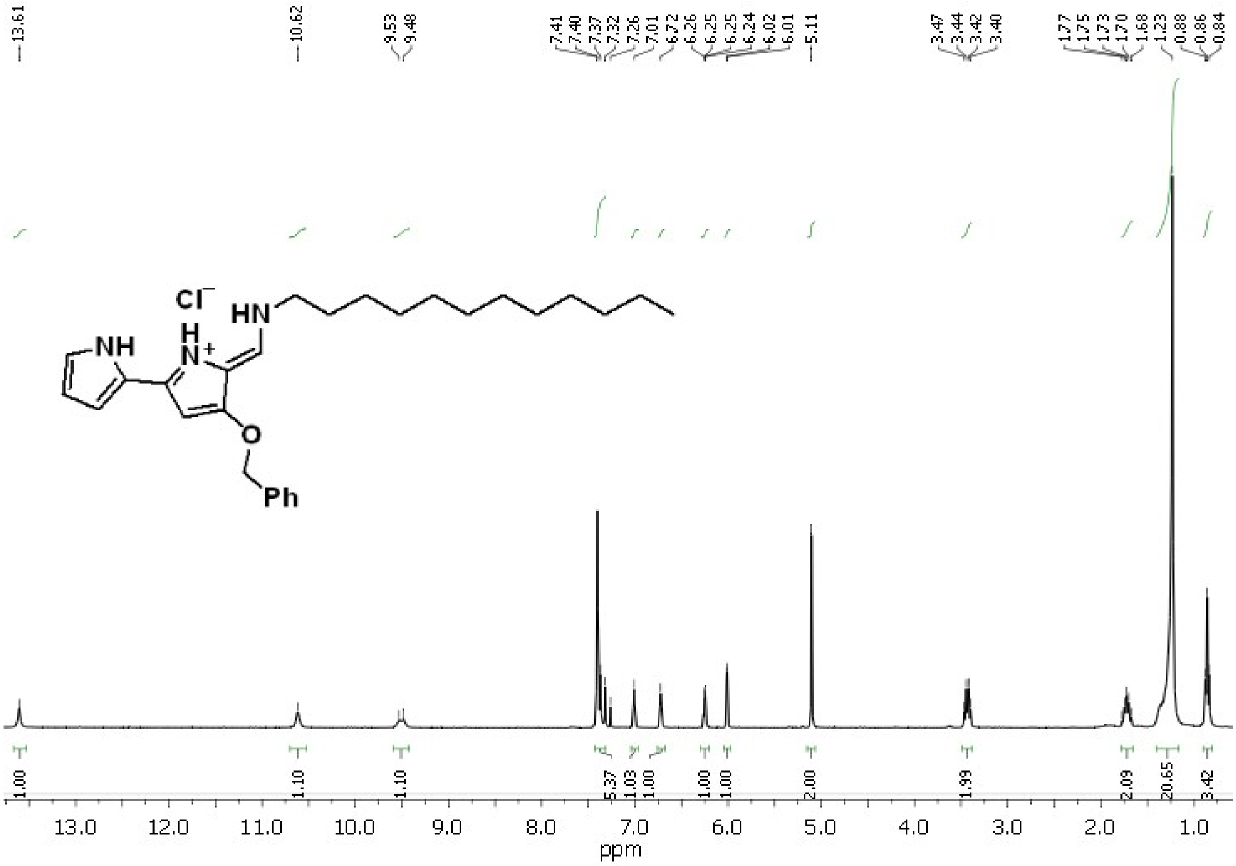

Figure S75. ^{1}H NMR ($CDCl_3$) of compound **43**. HCl.

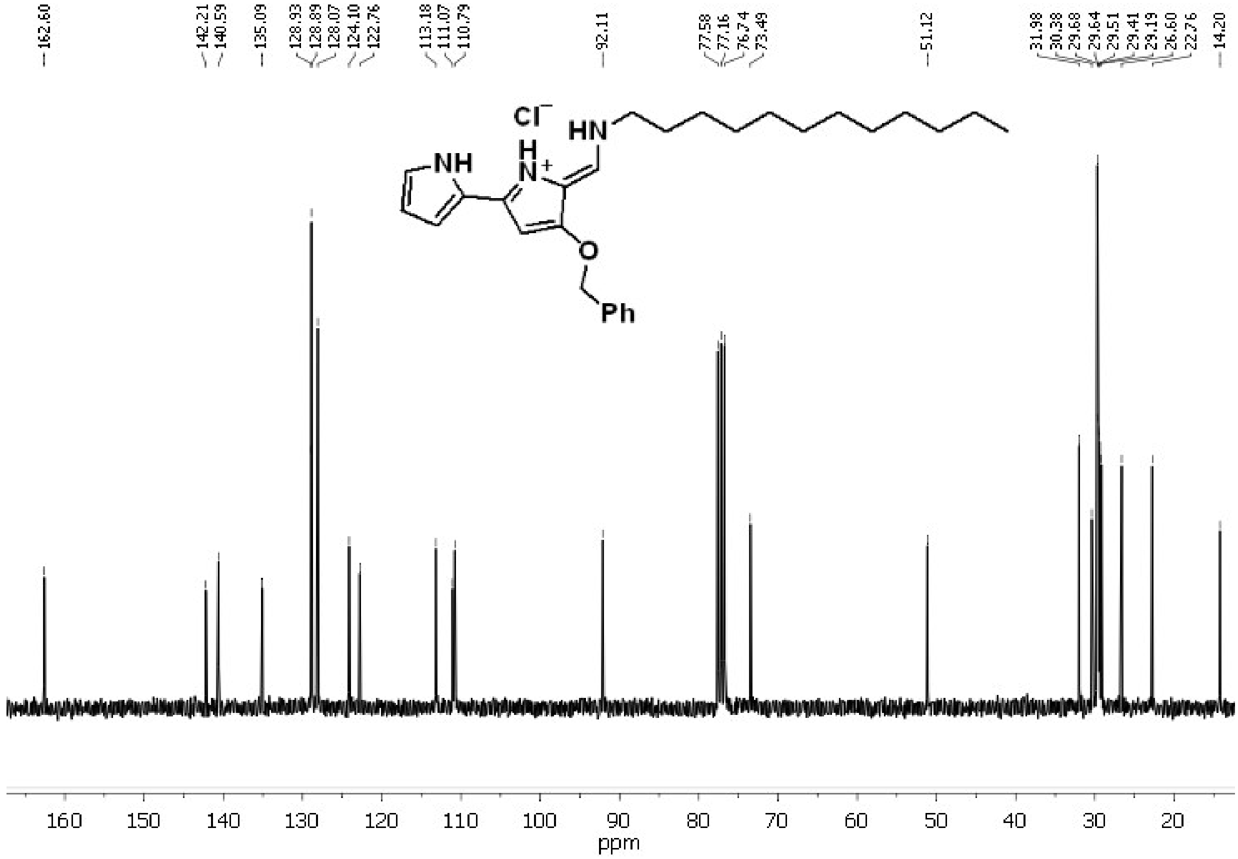

Figure S76. [13] CNMR ($CDCl_3$) of compound **43**. HCl.

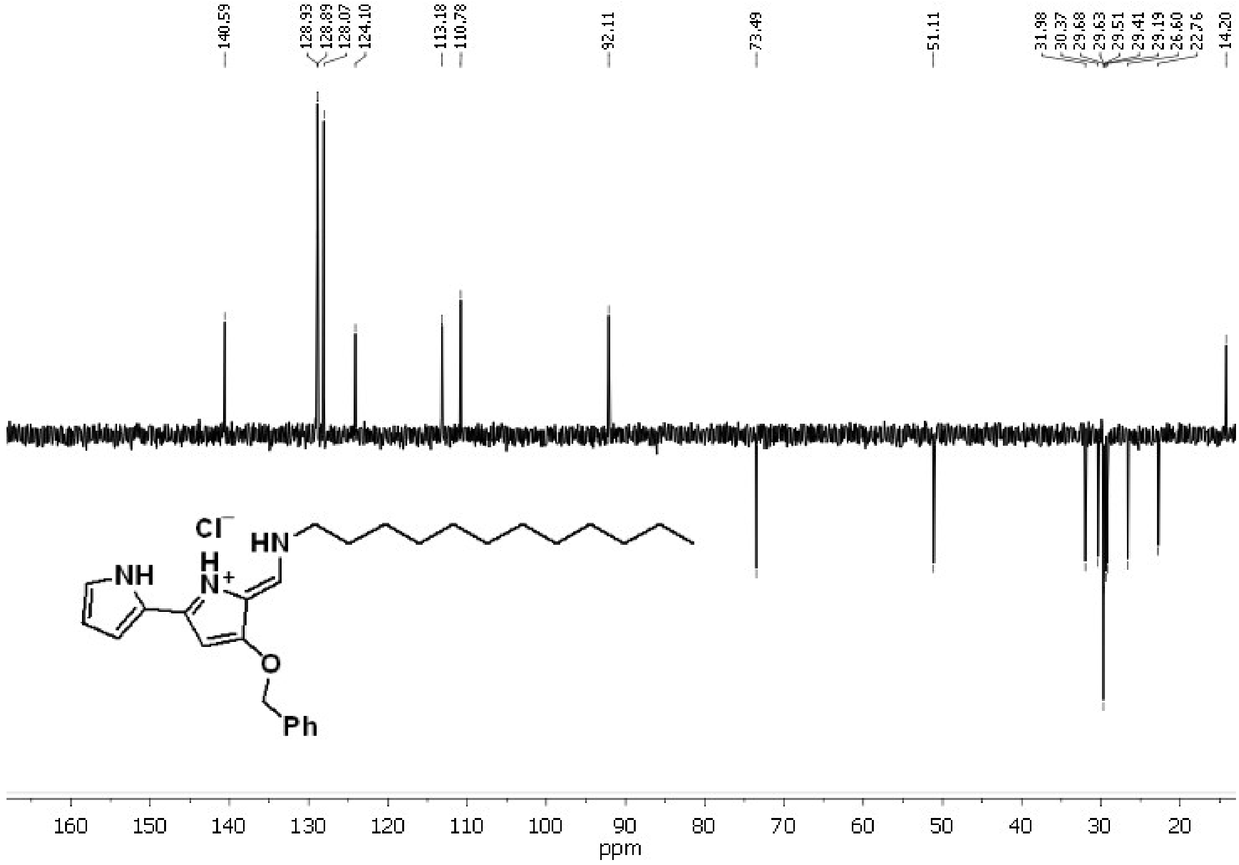

Figure S77. DEPT [13] CNMR ($CDCl_3$) of compound **43**. HCl.

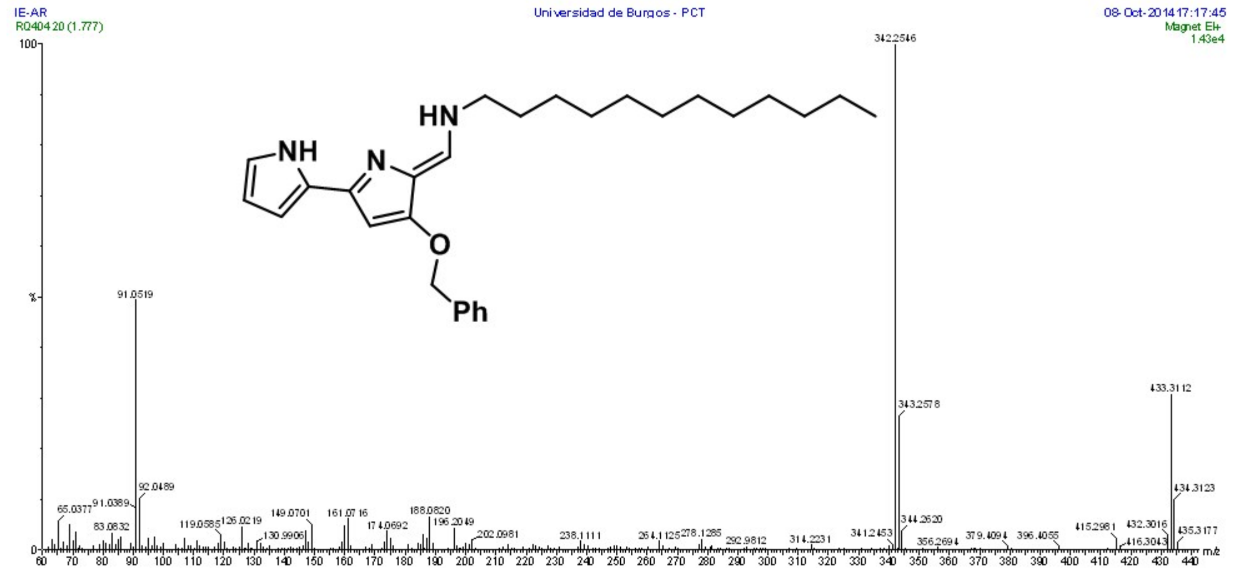

Figure S78. HRMS (EI) of compound **43**.

2. ANION TRANSPORT STUDIES

2.1. Preparation of Vesicles

Using a rotary evaporator a chloroform solution of 1-palmitoyl-2-oleoyl-sn-glycero-3- phosphocholine (POPC) was evaporated in vacuo and the lipid film obtained was dried under high vacuum for at least 4 hours. POPC (1-palmitoyl-2-oleoyl-sn-glycero-3- phosphocholine) was supplied by Sigma-Aldrich or Genzyme and it was stored at –20°C as a solution in chloroform (500 mg POPC in 20 mL chloroform or 1 g POPC in 35 mL chloroform). The lipid film was rehydrated by careful vortexing with a sodium chloride solution (489 mM NaCl and 5 mM phosphate buffer, pH 7.2, ionic strength 500 mM). The lipid suspension was subjected to nine freeze-thaw cycles, where the suspension was alternatively allowed to freeze in a liquid nitrogen bath, followed by thawing in a mild water bath. Finally twenty nine extrusions were carried out through a 200 nm polycarbonate Nucleopore membrane using a LiposoFast Basic extruder (Aves tin, Inc.). The resulting unilamellar vesicles were dialyzed against $NaNO_3$ solution (489 mM $NaNO_3$ and 5 mM phosphate buffer, pH 7.2, ionic strength 500 mM) to remove the unencapsulated chloride.

2.2. Chloride/nitrate transport assays

Unilamellar POPC vesicles containing NaCl (prepared as described above) were suspended in a 489 mM $NaNO_3$ solution buffered to pH 7.2 with sodium phosphate salts (5 mM buffer). The final lipid concentration per sample was 0.5 mM and the total volume 5 mL. A DMSO solution of the carrier molecule, typically 5 or 10 µL to avoid the influence of the solvent molecule in the assay, was added and the chloride release out of vesicles was monitored using a chloride selective electrode for 5 minutes. Then, the vesicles were lysed by adding 20 µl of polyoxyethylene(8)lauryl ether (0.232 mM in 7:1 water:DMSO v/v) or Triton-X (10% dispersion in water) and a total chloride reading was taken at t =

6 min. The initial value was set at 0 % chloride efflux and the final one was set as 100 % chloride efflux. All other data were calibrated to these points.

2.3. Initial rate of chloride efflux (kini) determination

The initial rate of chloride release (k_{ini}) was calculated from chloride/nitrate transport experiments as described above. The chloride release by 0.05% tambjamine was monitored over time. The chloride efflux was fitted with the following asymptotic function using Origin 8.1, where *y* is the chloride efflux (%) and *x* is the time (s):

$$y = a - b \cdot c^{x}$$

The initial rate of chloride release (k_{ini}) is calculated as k_{ini} = -b·ln(c) and is expressed in % s^{-1}. Figures S79-S121 show all of the obtained transport data for 0.05% tambjamine to lipid and the corresponding asymptotic fit. An overview of the obtained initial rates of chloride release (k_{ini}) can be found in Table S1 in S91.

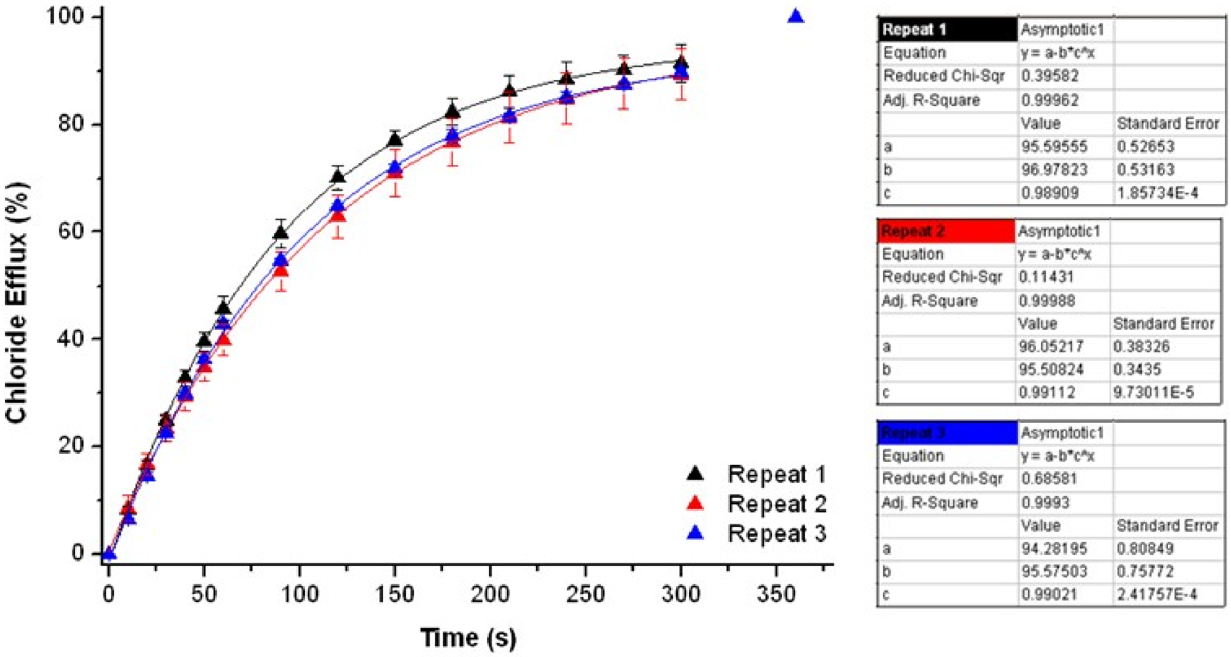

Figure S79: Overview of the initial rate of chloride release (k_{ini}) for compound **1**. For experimental details, see main text.

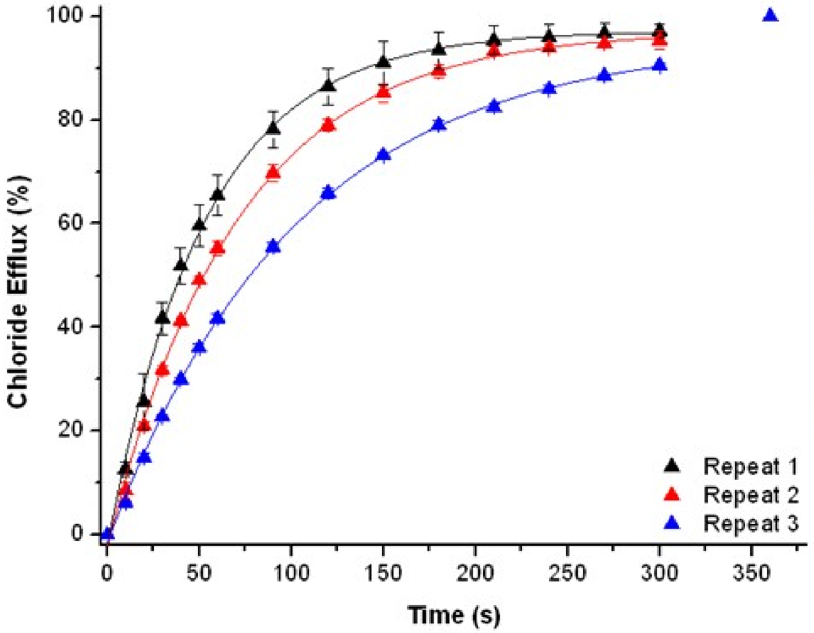

Repeat 1	Asymptotic1	
Equation	y = a-b*c^x	
Reduced Chi-Sqr	2.23174	
Adj. R-Square	0.99796	
	Value	Standard Error
a	97.23197	0.727
b	99.4597	1.22896
c	0.98139	5.57605E-4

Repeat 2	Asymptotic1	
Equation	y = a-b*c^x	
Reduced Chi-Sqr	1.26133	
Adj. R-Square	0.99888	
	Value	Standard Error
a	97.33883	0.69258
b	99.65664	0.89247
c	0.98592	3.5571E-4

Repeat 3	Asymptotic1	
Equation	y = a-b*c^x	
Reduced Chi-Sqr	0.46872	
Adj. R-Square	0.99954	
	Value	Standard Error
a	95.50602	0.67393
b	97.07728	0.62964
c	0.99026	1.96562E-4

Figure S80: Overview of the initial rate of chloride release (k_{ini}) for compound **2**. For experimental details, see main text.

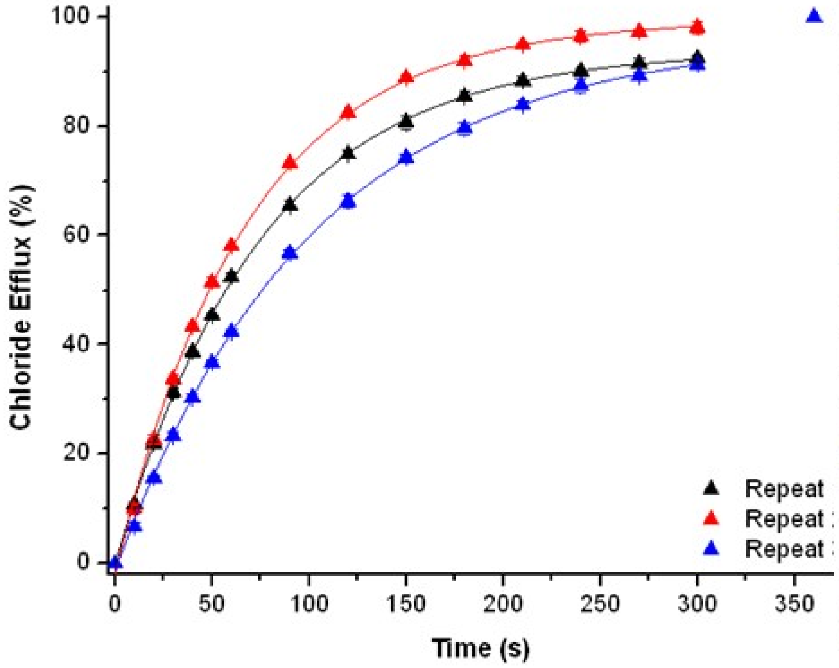

Repeat 1	Asymptotic1	
Equation	y = a-b*c^x	
Reduced Chi-Sqr	0,14241	
Adj. R-Square	0,99986	
	Value	Standard Error
a	94,01036	0,24807
b	94,26402	0,30059
c	0,98673	1,22945E-4

Repeat 2	Asymptotic1	
Equation	y = a-b*c^x	
Reduced Chi-Sqr	0,93043	
Adj. R-Square	0,9992	
	Value	Standard Error
a	99,4893	0,57002
b	101,63729	0,76752
c	0,98531	3,05955E-4

Repeat 3		
Equation	y = a-b*c^x	
Reduced Chi-Sqr	0,34847	
Adj. R-Square	0,99966	
	Value	Standard Error
a	96,45426	0,57622
b	97,77935	0,54007
c	0,99021	1,68448E-4

Figure S81: Overview of the initial rate of chloride release (k_{ini}) for compound **3**. For experimental details, see main text.

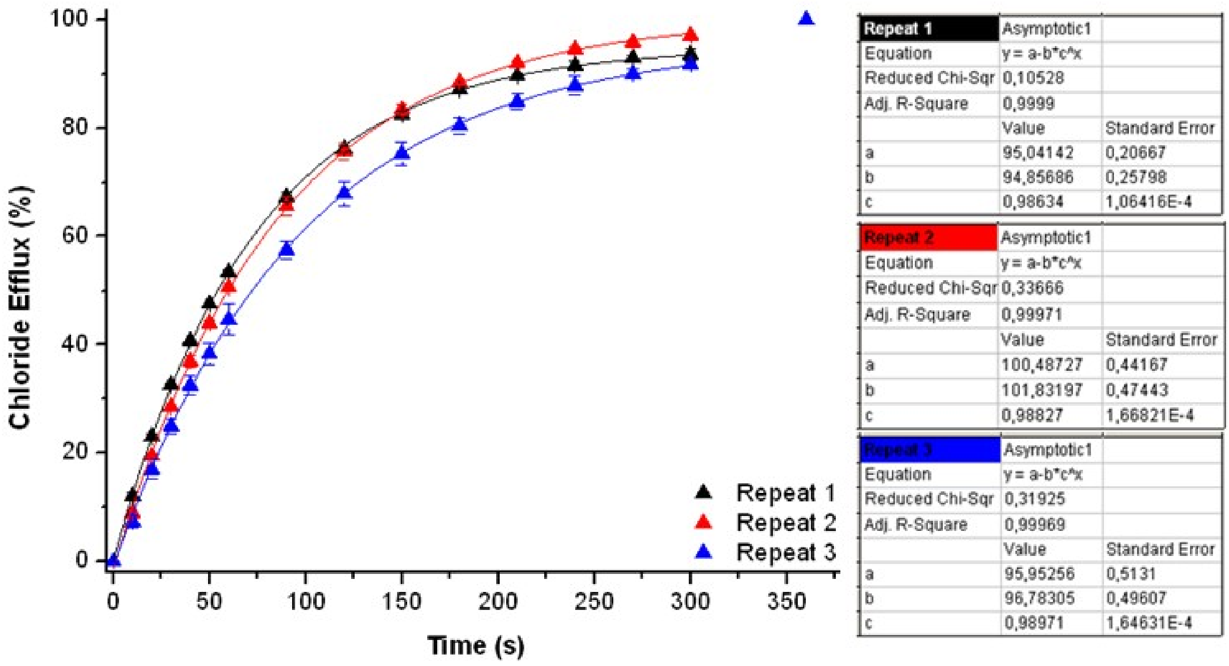

Figure S82: Overview of the initial rate of chloride release (k_{ini}) for compound **4**. For experimental details, see main text.

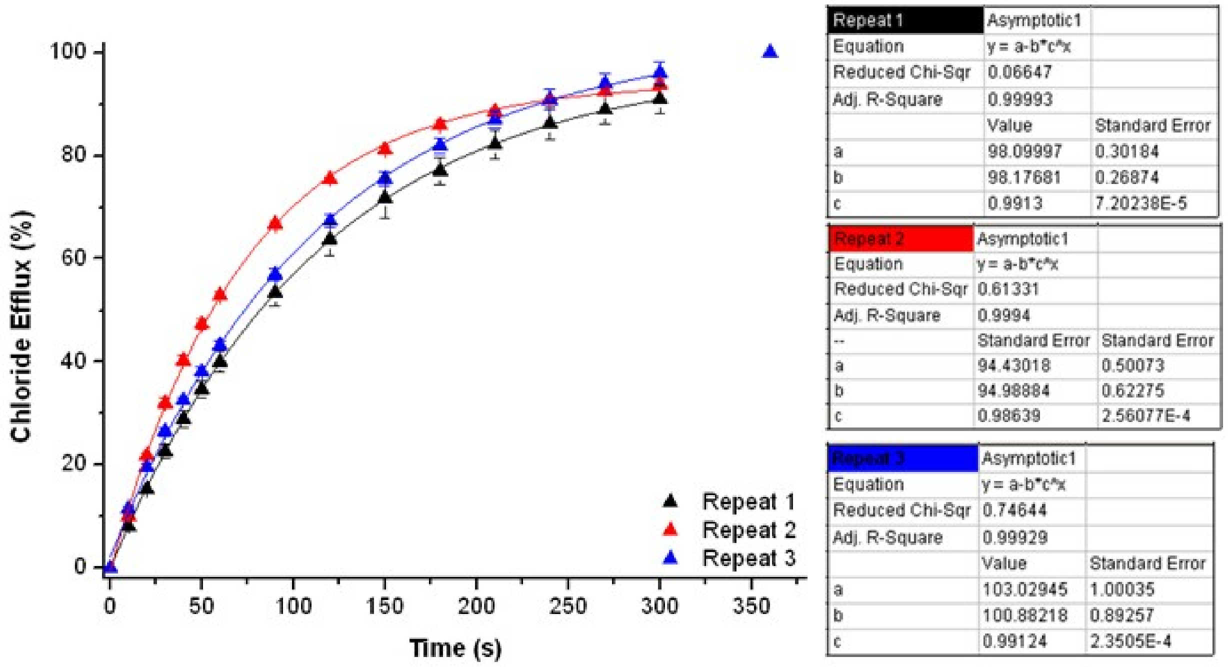

Figure S83: Overview of the initial rate of chloride release (k_{ini}) for compound **5**. For experimental details, see main text.

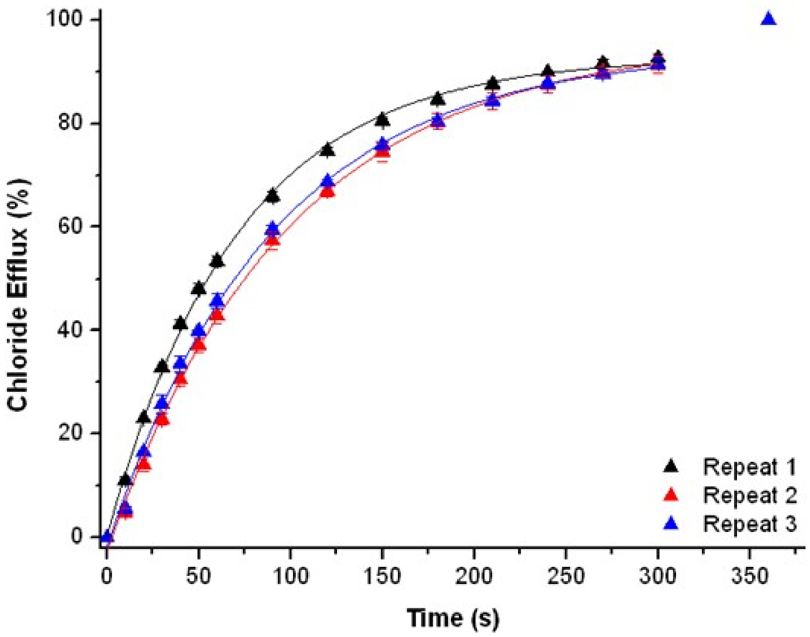

Repeat 1	Asymptotic1	
Equation	y = a-b*c^x	
Reduced Chi-Sqr	0,93108	
Adj. R-Square	0,99904	
	Value	Standard Error
a	92,9009	0,60003
b	92,67925	0,76679
c	0,98603	3,27359E-4

Repeat 2	Asymptotic1	
Equation	y = a-b*c^x	
Reduced Chi-Sqr	1,20785	
Adj. R-Square	0,99886	
	Value	Standard Error
a	96,2809	1,02778
b	98,83727	0,98042
c	0,98992	3,12125E-4

Repeat 3	Asymptotic1	
Equation	y = a-b*c^x	
Reduced Chi-Sqr	1,24358	
Adj. R-Square	0,99878	
	Value	Standard Error
a	94,18636	0,9112
b	96,00155	0,93341
c	0,98889	3,34272E-4

Figure S84: Overview of the initial rate of chloride release (k_{ini}) for compound **6**. For experimental details, see main text.

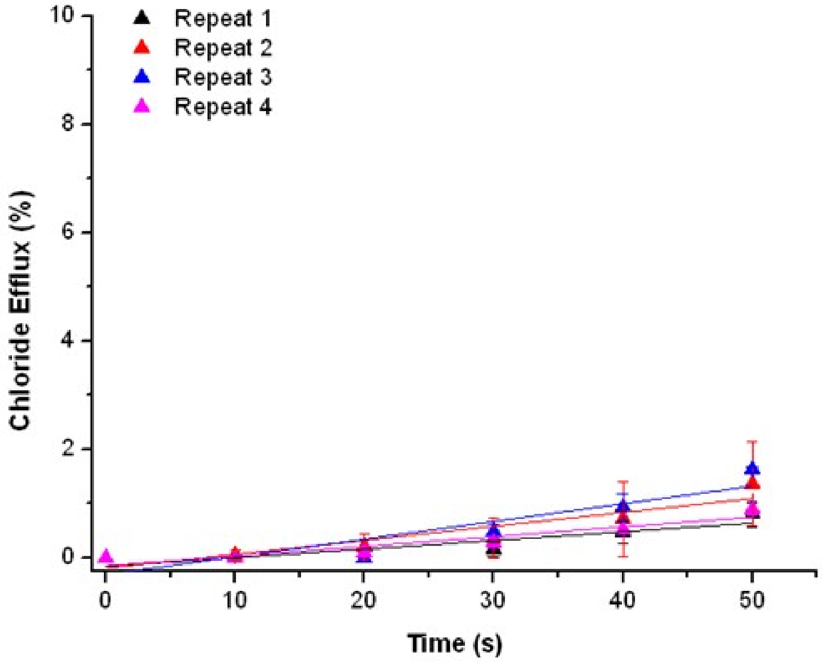

Repeat 1	y = a + b*x	
Weight	No Weighting	
Residual Sum of Squares	0,10527	
Pearson's r	0,89982	
Adj. R-Square	0,7621	
	Value	Standard Error
Intercept	-0,16091	0,11741
Slope	0,016	0,00388

Repeat 2	y = a + b*x	
Weight	No Weighting	
Residual Sum of Squares	0,1905	
Pearson's r	0,92688	
Adj. R-Square	0,82389	
	Value	Standard Error
Intercept	-0,19327	0,15794
Slope	0,02576	0,00522

Repeat 3	y = a + b*x	
Weight	No Weighting	
Residual Sum of Squares	0,34706	
Pearson's r	0,91831	
Adj. R-Square	0,80412	
	Value	Standard Error
Intercept	-0,30902	0,21319
Slope	0,03267	0,00704

Repeat 4	y = a + b*x	
Weight	No Weighting	
Residual Sum of Squares	0,07251	
Pearson's r	0,94078	
Adj. R-Square	0,85633	
	Value	Standard Error
Intercept	-0,14539	0,09744
Slope	0,01786	0,00322

Figure S85: Overview of the initial rate of chloride release (k_{ini}) for compound **7**. For experimental details, see main text.

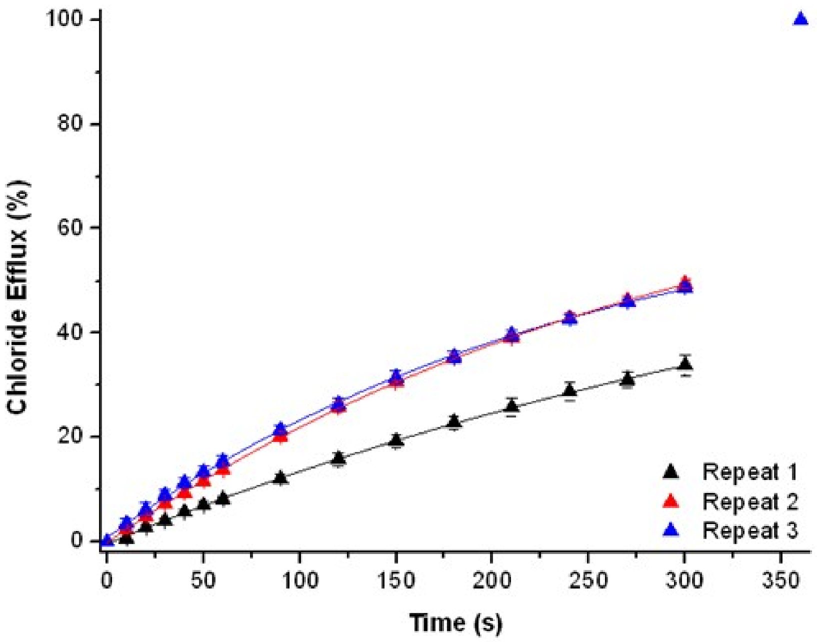

Repeat 1	Asymptotic1	
Equation	y = a-b*c^x	
Reduced Chi-Sqr	0,05194	
Adj. R-Square	0,99962	
	Value	Standard Error
a	72,92935	3,76848
b	73,2978	3,68952
c	0,99791	1,42928E-4

Repeat 2	Asymptotic1	
Equation	y = a-b*c^x	
Reduced Chi-Sqr	0,0272	
Adj. R-Square	0,99991	
	Value	Standard Error
a	78,72448	1,12643
b	78,90186	1,07159
c	0,99671	7,275E-5

Repeat 3	Asymptotic1	
Equation	y = a-b*c^x	
Reduced Chi-Sqr	0,11791	
Adj. R-Square	0,99956	
	Value	Standard Error
a	69,03689	1,61516
b	68,25504	1,50521
c	0,99601	1,59528E-4

Figure S86: Overview of the initial rate of chloride release (k_{ini}) for compound **8**. For experimental details, see main text.

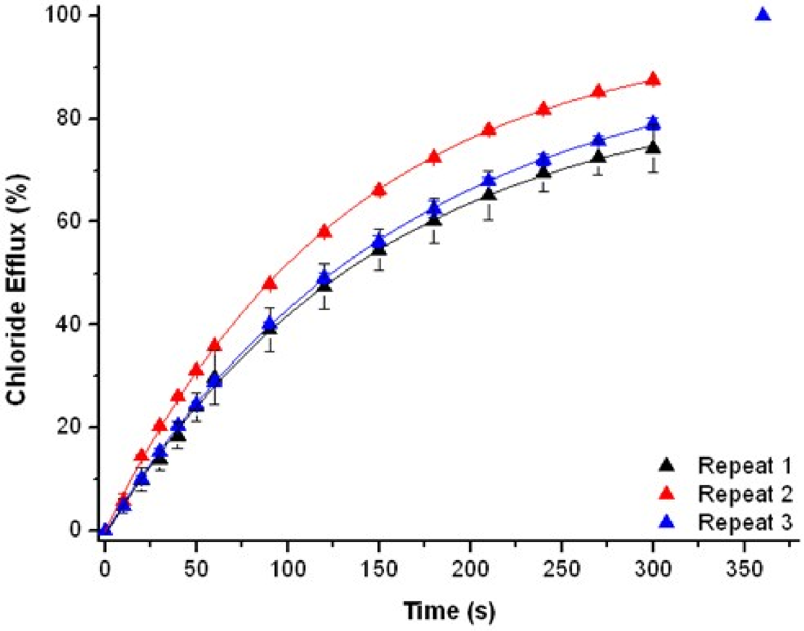

Repeat 1	Asymptotic1	
Equation	y = a-b*c^x	
Reduced Chi-Sqr	0,59179	
Adj. R-Square	0,99914	
	Value	Standard Error
a	86,47023	1,39216
b	87,34245	1,21232
c	0,9933	2,41919E-4

Repeat 2	Asymptotic1	
Equation	y = a-b*c^x	
Reduced Chi-Sqr	0,2842	
Adj. R-Square	0,99969	
	Value	Standard Error
a	97,52551	0,78188
b	97,53545	0,67985
c	0,99242	1,49049E-4

Repeat 3	Asymptotic1	
Equation	y = a-b*c^x	
Reduced Chi-Sqr	0,08852	
Adj. R-Square	0,99988	
	Value	Standard Error
a	93,15793	0,61246
b	93,61817	0,53696
c	0,99377	8,81295E-5

Figure S87: Overview of the initial rate of chloride release (k_{ini}) for compound **9**. For experimental details, see main text.

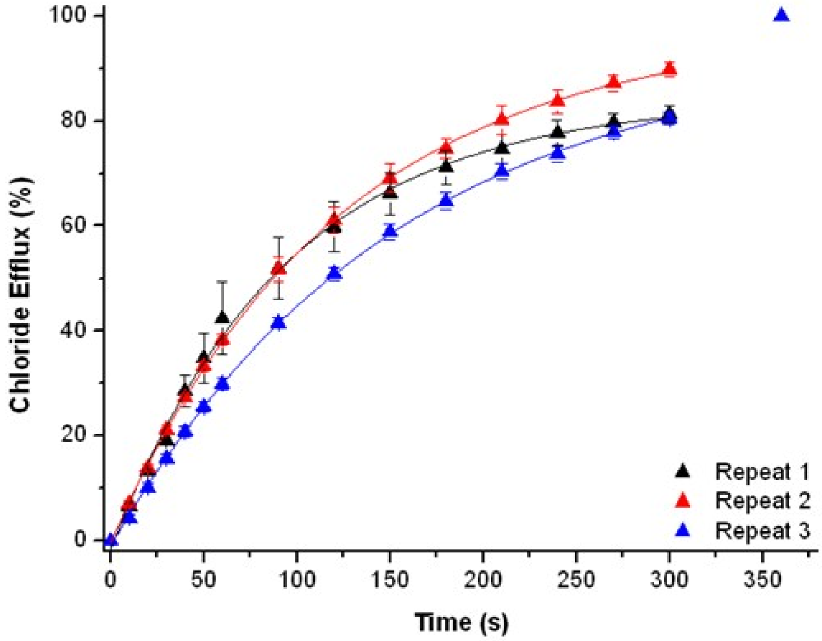

Repeat 1	Asymptotic1	
Equation	y = a-b*c^x	
Reduced Chi-Sqr	2,30628	
Adj. R-Square	0,99716	
	Value	Standard Error
a	84,19745	1,31086
b	85,85877	1,3006
c	0,98933	5,0329E-4

Repeat 2	Asymptotic1	
Equation	y = a-b*c^x	
Reduced Chi-Sqr	0,1958	
Adj. R-Square	0,9998	
	Value	Standard Error
a	97,44289	0,56239
b	97,78789	0,49435
c	0,99174	1,23626E-4

Repeat 3	Asymptotic1	
Equation	y = a-b*c^x	
Reduced Chi-Sqr	0,20461	
Adj. R-Square	0,99974	
	Value	Standard Error
a	93,69498	0,84602
b	94,75804	0,73778
c	0,99342	1,31393E-4

Figure S88: Overview of the initial rate of chloride release (k_{ini}) for compound **10**. For experimental details, see main text.

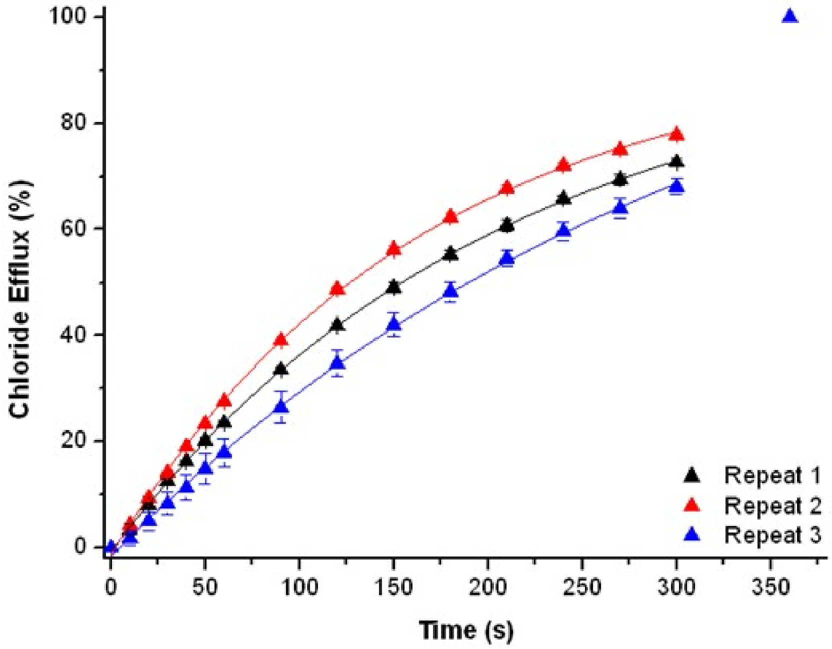

Repeat 1	Asymptotic1	
Equation	y = a-b*c^x	
Reduced Chi-Sqr	0,08455	
Adj. R-Square	0,99987	
	Value	Standard Error
a	94,95333	0,93454
b	95,59893	0,84757
c	0,99511	8,9409E-5

Repeat 2	Asymptotic1	
Equation	y = a-b*c^x	
Reduced Chi-Sqr	0,30515	
Adj. R-Square	0,9996	
	Value	Standard Error
a	93,32628	1,16798
b	94,54354	1,02578
c	0,99386	1,62418E-4

Repeat 3	Asymptotic1	
Equation	y = a-b*c^x	
Reduced Chi-Sqr	0,37541	
Adj. R-Square	0,99935	
	Value	Standard Error
a	114,44022	4,70022
b	115,96201	4,49476
c	0,9969	1,89934E-4

Figure S89: Overview of the initial rate of chloride release (k_{ini}) for compound **11**. For experimental details, see main text.

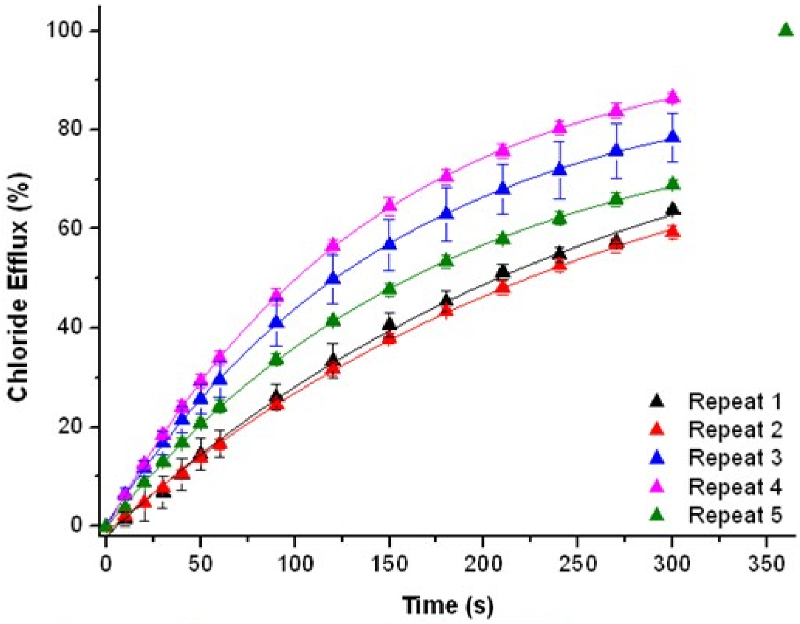

Repeat 1	Asymptotic1	
Equation	y = a-b*c^x	
Reduced Chi-Sqr	1,2061	
Adj. R-Square	0,99759	
	Value	Standard Error
a	93,77851	5,7546
b	95,73083	5,39833
c	0,99623	3,73058E-4

Repeat 2	Asymptotic1	
Equation	y = a-b*c^x	
Reduced Chi-Sqr	0,28447	
Adj. R-Square	0,99936	
	Value	Standard Error
a	90,5735	2,96632
b	91,81552	2,79218
c	0,99634	1,91671E-4

Repeat 3	Asymptotic1	
Equation	y = a-b*c^x	
Reduced Chi-Sqr	0,07607	
Adj. R-Square	0,9999	
	Value	Standard Error
a	90,95473	0,52293
b	90,49033	0,45633
c	0,99347	8,39694E-5

Repeat 4	Asymptotic1	
Equation	y = a-b*c^x	
Reduced Chi-Sqr	0,05226	
Adj. R-Square	0,99994	
	Value	Standard Error
a	97,88477	0,37157
b	98,19177	0,32269
c	0,99287	6,36265E-5

Repeat 5	Asymptotic1	
Equation	y = a-b*c^x	
Reduced Chi-Sqr	0,10654	
Adj. R-Square	0,99981	
	Value	Standard Error
a	84,05305	0,8051
b	84,25348	0,71505
c	0,99436	1,09516E-4

Figure S90: Overview of the initial rate of chloride release (k_{ini}) for compound **12**. For experimental details, see main text.

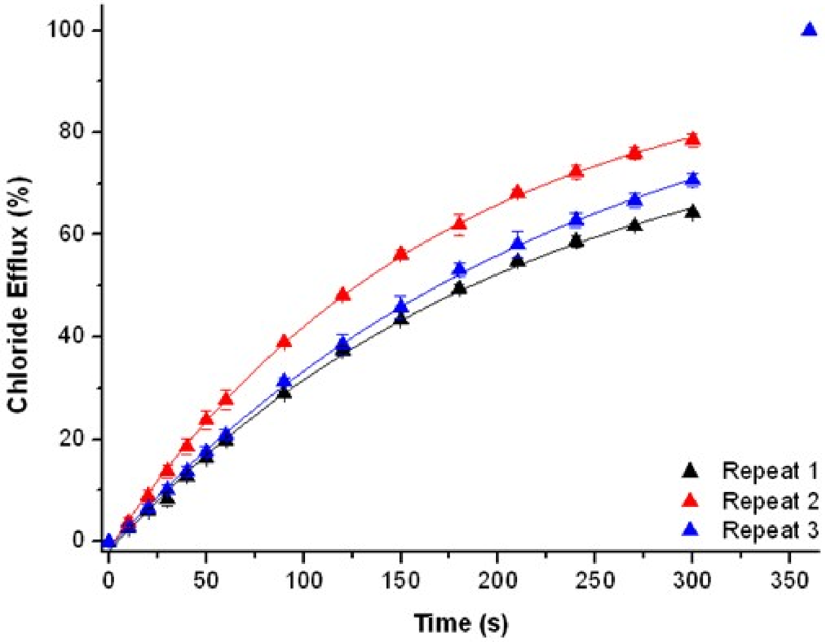

Repeat 1	Asymptotic1	
Equation	y = a-b*c^x	
Reduced Chi-Sqr	0,82526	
Adj. R-Square	0,99846	
	Value	Standard Error
a	86,35687	3,07031
b	88,16706	2,79543
c	0,99524	3,05537E-4

Repeat 2	Asymptotic1	
Equation	y = a-b*c^x	
Reduced Chi-Sqr	0,36106	
Adj. R-Square	0,99954	
	Value	Standard Error
a	95,19524	1,34154
b	96,59907	1,18264
c	0,99404	1,73849E-4

Repeat 3	Asymptotic1	
Equation	y = a-b*c^x	
Reduced Chi-Sqr	0,35906	
Adj. R-Square	0,99942	
	Value	Standard Error
a	99,28405	2,54954
b	100,49071	2,36038
c	0,99579	1,84969E-4

Figure S91: Overview of the initial rate of chloride release (k_{ini}) for compound **13**. For experimental details, see main text.

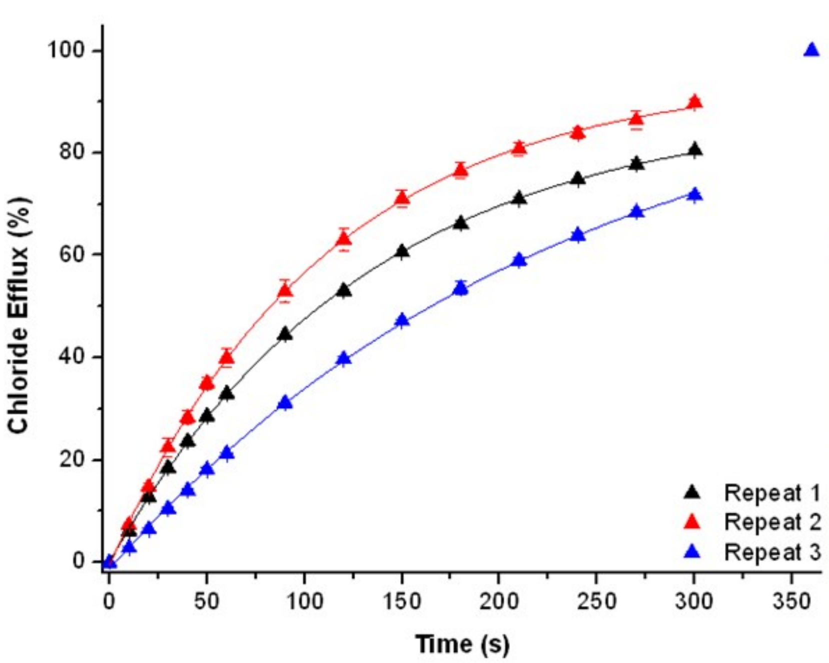

Repeat 1	Asymptotic1	
Equation	y = a-b*c^x	
Reduced Chi-Sqr	0,08326	
Adj. R-Square	0,99989	
	Value	Standard Error
a	89,22549	0,42316
b	89,09265	0,36794
c	0,99242	8,83178E-5

Repeat 2	Asymptotic1	
Equation	y = a-b*c^x	
Reduced Chi-Sqr	0,2409	
Adj. R-Square	0,99975	
	Value	Standard Error
a	95,14042	0,5375
b	95,69821	0,48569
c	0,99093	1,41349E-4

Repeat 3	Asymptotic1	
Equation	y = a-b*c^x	
Reduced Chi-Sqr	0,2788	
Adj. R-Square	0,99957	
	Value	Standard Error
a	100,70822	2,21479
b	102,01423	2,04847
c	0,99576	1,60072E-4

Figure S92: Overview of the initial rate of chloride release (k_{ini}) for compound **14**. For experimental details, see main text.

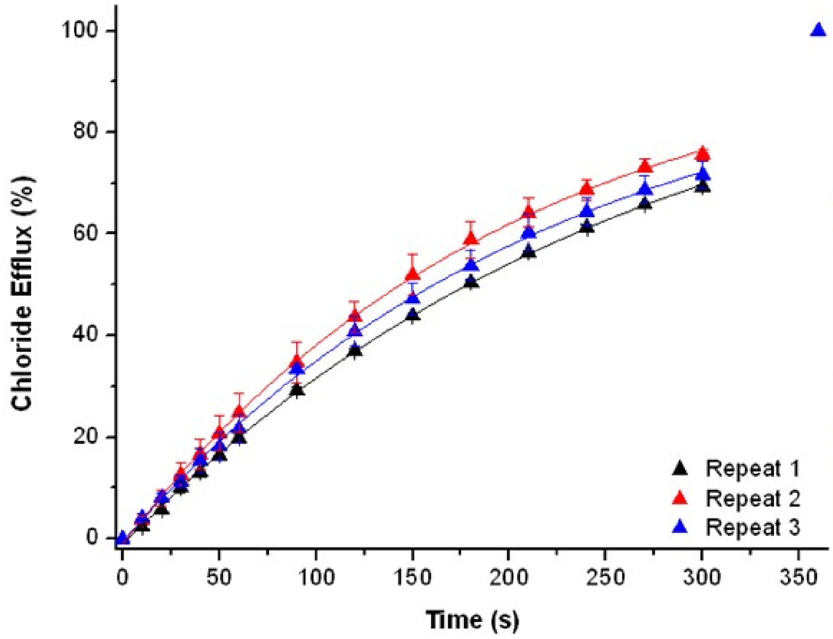

Repeat 1	Asymptotic1	
Equation	y = a-b*c^x	
Reduced Chi-Sqr	0,27519	
Adj. R-Square	0,99962	
	Value	Standard Error
a	99,09386	1,64633
b	100,09068	1,49034
c	0,99505	1,53445E-4

Repeat 2	Asymptotic1	
Equation	y = a-b*c^x	
Reduced Chi-Sqr	0,4786	
Adj. R-Square	0,99924	
	Value	Standard Error
a	98,08585	2,64063
b	98,64113	2,42614
c	0,99554	2,12772E-4

Repeat 3	Asymptotic1	
Equation	y = a-b*c^x	
Reduced Chi-Sqr	0,2276	
Adj. R-Square	0,99962	
	Value	Standard Error
a	104,08284	2,56031
b	105,24484	2,40514
c	0,99627	1,48264E-4

Figure S93: Overview of the initial rate of chloride release (k_{ini}) for compound **15**. For experimental details, see main text.

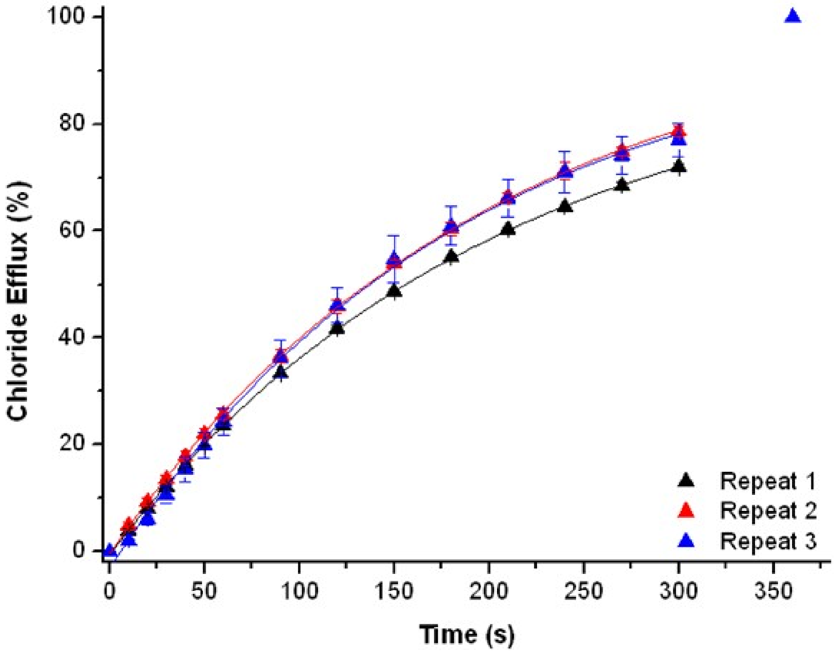

Repeat 1	Asymptotic1	
Equation	y = a-b*c^x	
Reduced Chi-Sqr	0,06485	
Adj. R-Square	0,9999	
	Value	Standard Error
a	93,00889	0,7928
b	93,56915	0,71723
c	0,99503	7,95756E-5

Repeat 2	Asymptotic1	
Equation	y = a-b*c^x	
Reduced Chi-Sqr	0,11913	
Adj. R-Square	0,99984	
	Value	Standard Error
a	101,62883	1,05957
b	102,0379	0,95751
c	0,99499	9,86751E-5

Repeat 3	Asymptotic1	
Equation	y = a-b*c^x	
Reduced Chi-Sqr	1,82724	
Adj. R-Square	0,9977	
	Value	Standard Error
a	98,01088	3,57839
b	101,19707	3,19584
c	0,99457	3,81101E-4

Figure S94: Overview of the initial rate of chloride release (k_{ini}) for compound **16**. For experimental details, see main text.

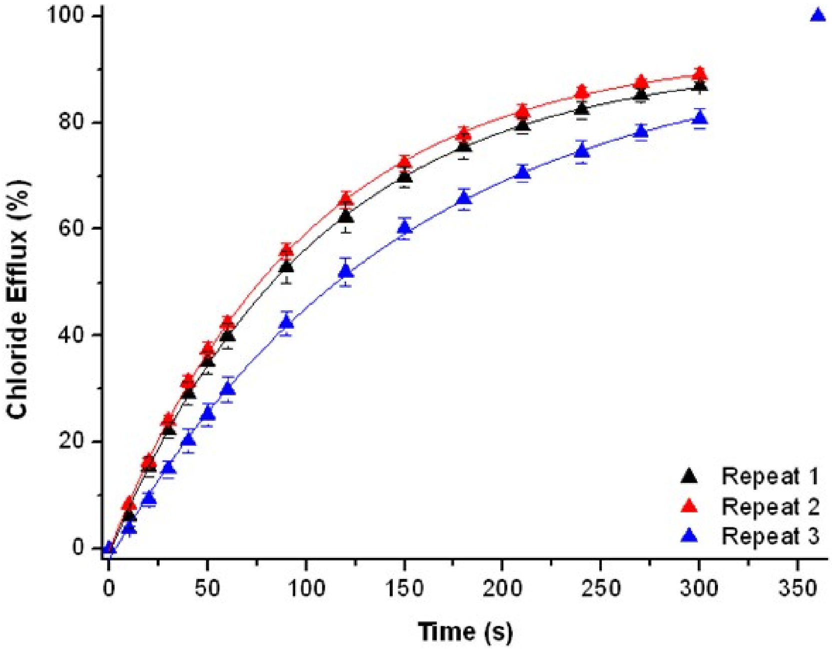

Repeat 1	Asymptotic1	
Equation	y = a-b*c^x	
Reduced Chi-Sqr	0,30062	
Adj. R-Square	0,99967	
	Value	Standard Error
a	91,93174	0,55475
b	92,77318	0,51321
c	0,99044	1,64172E-4

Repeat 2	Asymptotic1	
Equation	y = a-b*c^x	
Reduced Chi-Sqr	0,15015	
Adj. R-Square	0,99984	
	Value	Standard Error
a	93,61756	0,36568
b	93,85484	0,34746
c	0,98998	1,15737E-4

Repeat 3	Asymptotic1	
Equation	y = a-b*c^x	
Reduced Chi-Sqr	0,67927	
Adj. R-Square	0,99918	
	Value	Standard Error
a	93,14986	1,43874
b	95,17036	1,25128
c	0,99316	2,3739E-4

Figure S95: Overview of the initial rate of chloride release (k_{ini}) for compound **17**. For experimental details, see main text.

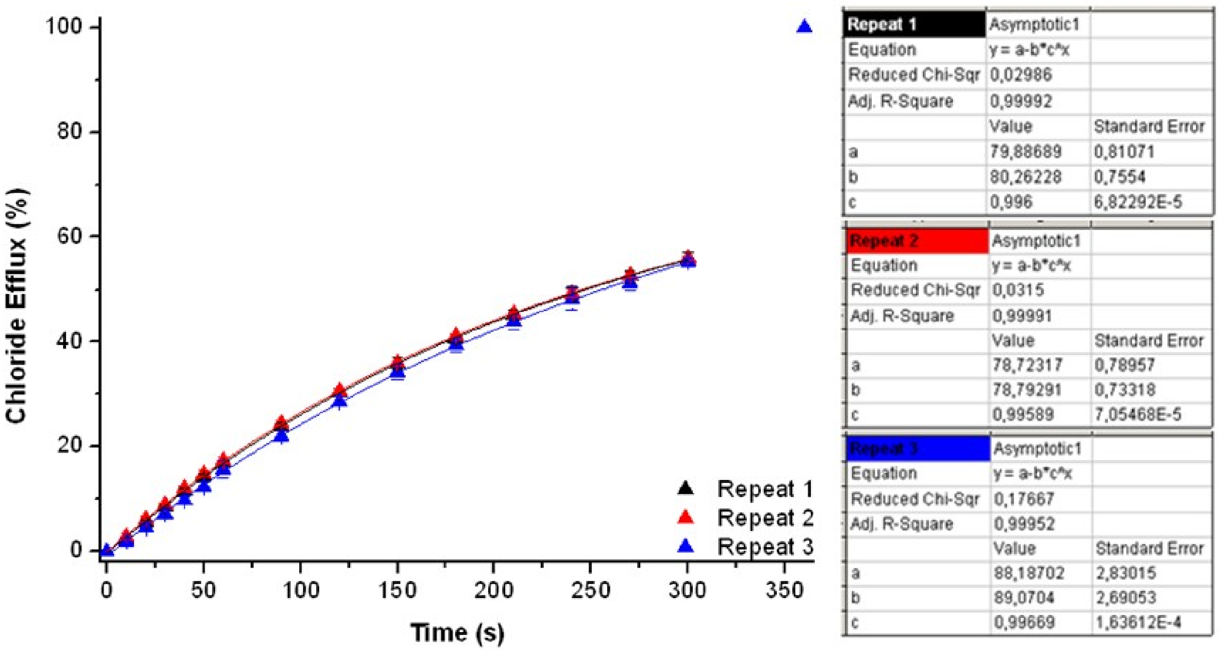

Repeat 1	Asymptotic1	
Equation	y = a-b*c^x	
Reduced Chi-Sqr	0,02986	
Adj. R-Square	0,99992	
	Value	Standard Error
a	79,88689	0,81071
b	80,26228	0,7554
c	0,996	6,82292E-5

Repeat 2	Asymptotic1	
Equation	y = a-b*c^x	
Reduced Chi-Sqr	0,0315	
Adj. R-Square	0,99991	
	Value	Standard Error
a	78,72317	0,78957
b	78,79291	0,73318
c	0,99589	7,05468E-5

Repeat 3	Asymptotic1	
Equation	y = a-b*c^x	
Reduced Chi-Sqr	0,17667	
Adj. R-Square	0,99952	
	Value	Standard Error
a	88,18702	2,83015
b	89,0704	2,69053
c	0,99669	1,63612E-4

Figure S96: Overview of the initial rate of chloride release (k_{ini}) for compound **18**. For experimental details, see main text.

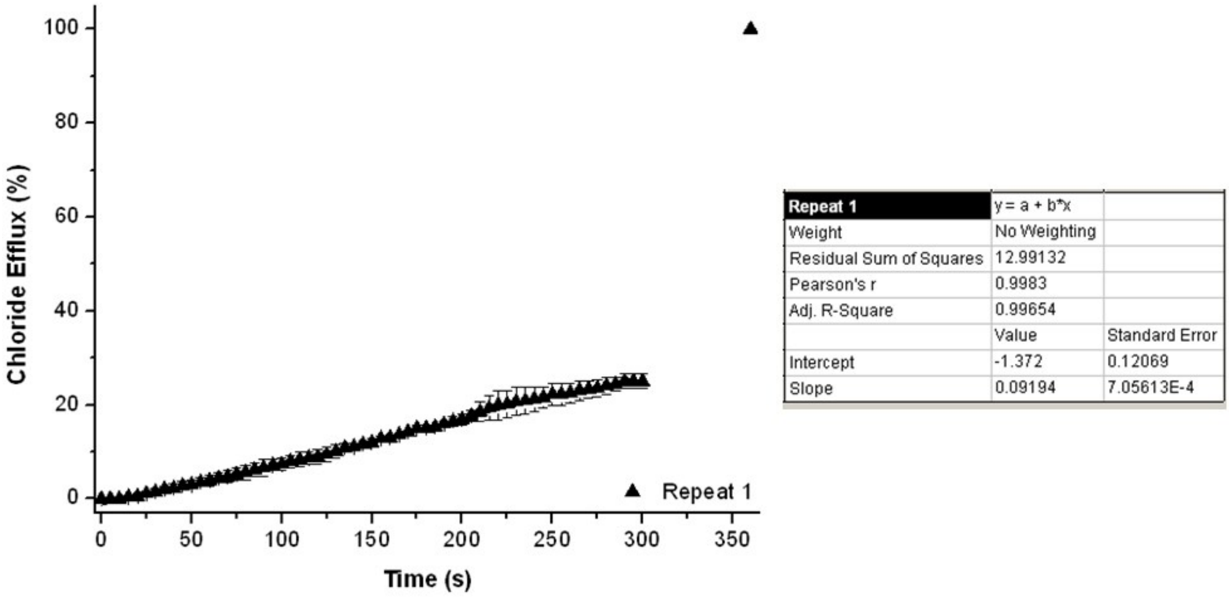

Repeat 1	y = a + b*x	
Weight	No Weighting	
Residual Sum of Squares	12.99132	
Pearson's r	0.9983	
Adj. R-Square	0.99654	
	Value	Standard Error
Intercept	-1.372	0.12069
Slope	0.09194	7.05613E-4

Figure S97: Overview of the initial rate of chloride release (k_{ini}) for compound **19**. For experimental details, see main text.

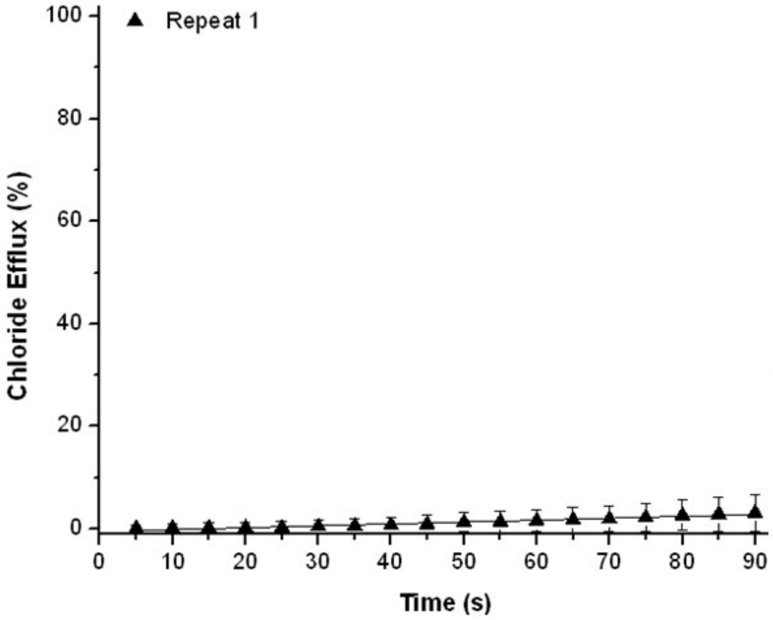

Repeat 1	y = a + b*x	
Weight	No Weighting	
Residual Sum of Squares	0.73686	
Pearson's r	0.9783	
Adj. R-Square	0.95438	
	Value	Standard Error
Intercept	-0.52607	0.10553
Slope	0.03683	0.00195

Figure S98: Overview of the initial rate of chloride release (k_{ini}) for compound **20**. For experimental details, see main text.

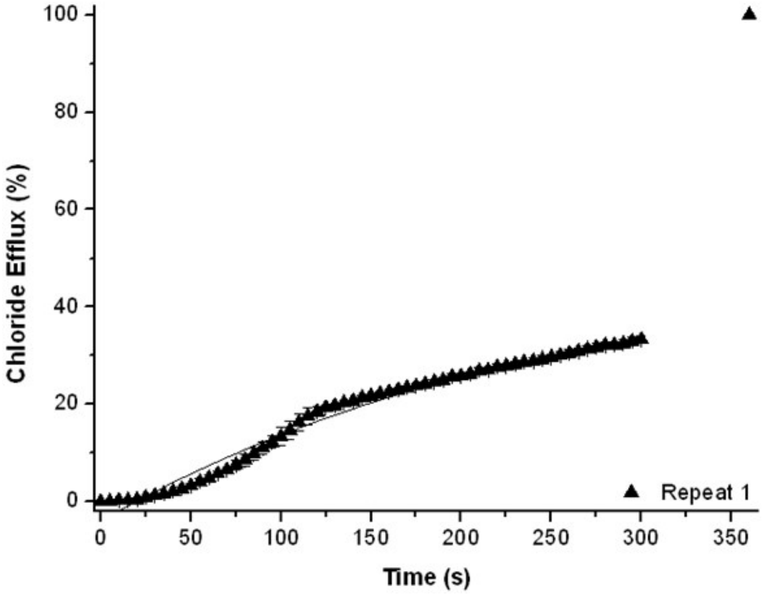

Repeat 1	Asymptotic1	
Equation	y = a-b*c^x	
Reduced Chi-Sqr	2.16493	
Adj. R-Square	0.98269	
	Value	Standard Error
a	50.50915	3.6283
b	54.59432	3.2768
c	0.99607	4.48389E-4

Figure S99: Overview of the initial rate of chloride release (k_{ini}) for compound **21**. For experimental details, see main text.

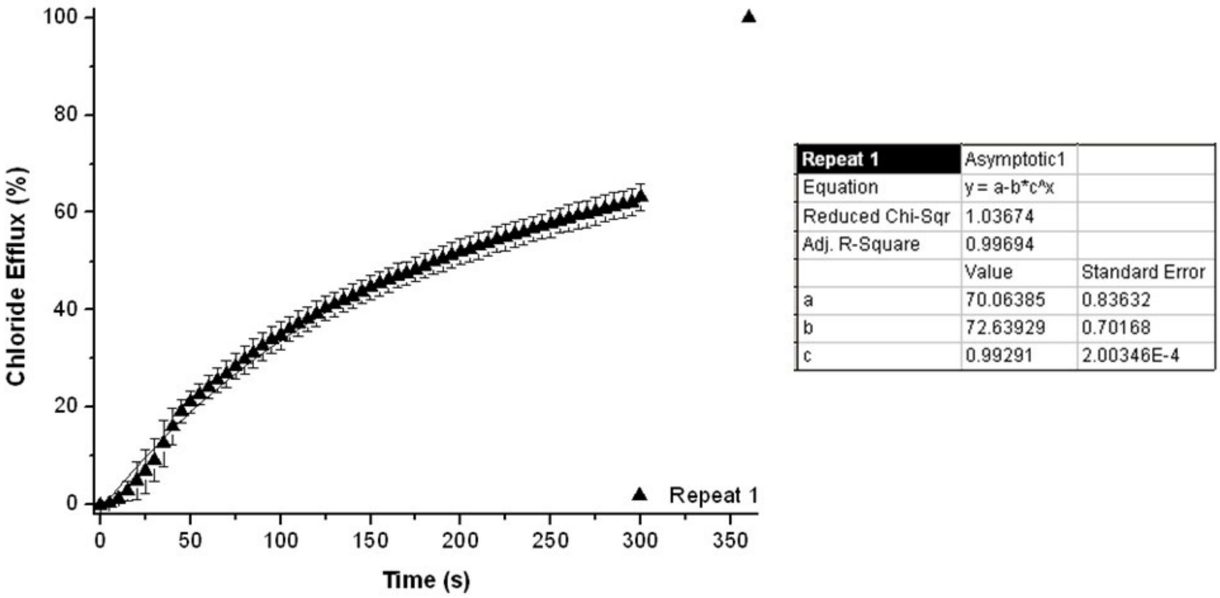

Figure S100: Overview of the initial rate of chloride release (k_{ini}) for compound **22**. For experimental details, see main text.

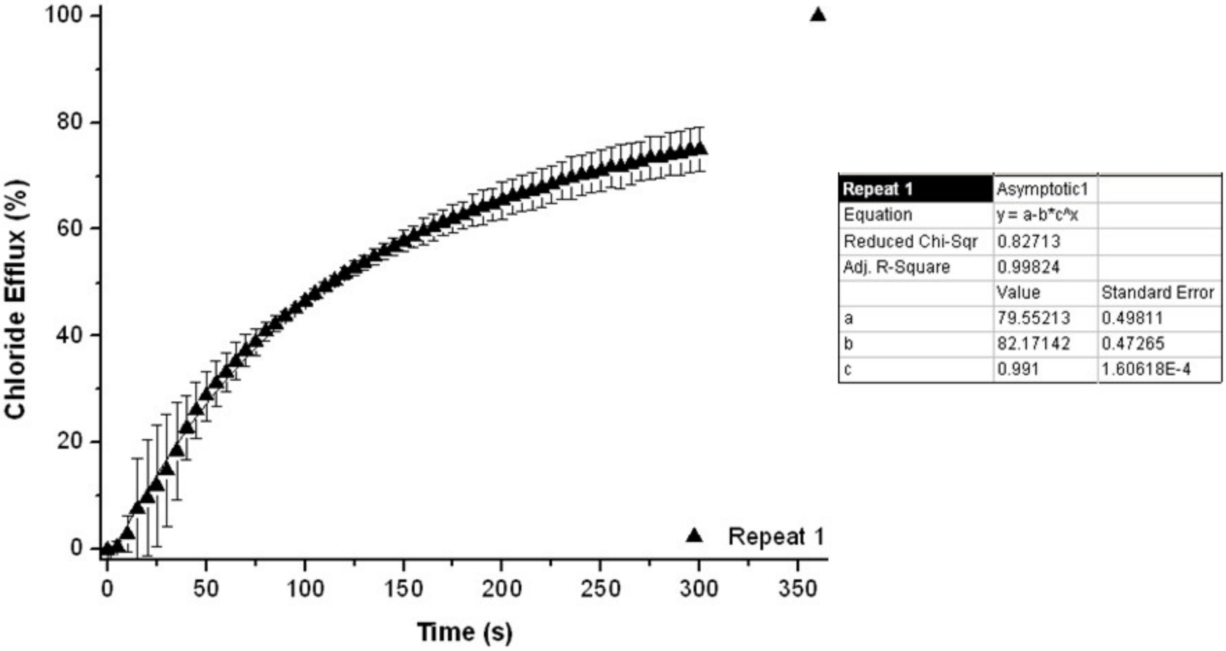

Figure S101. Overview of the initial rate of chloride release (k_{ini}) for compound **23**. For experimental details, see main text.

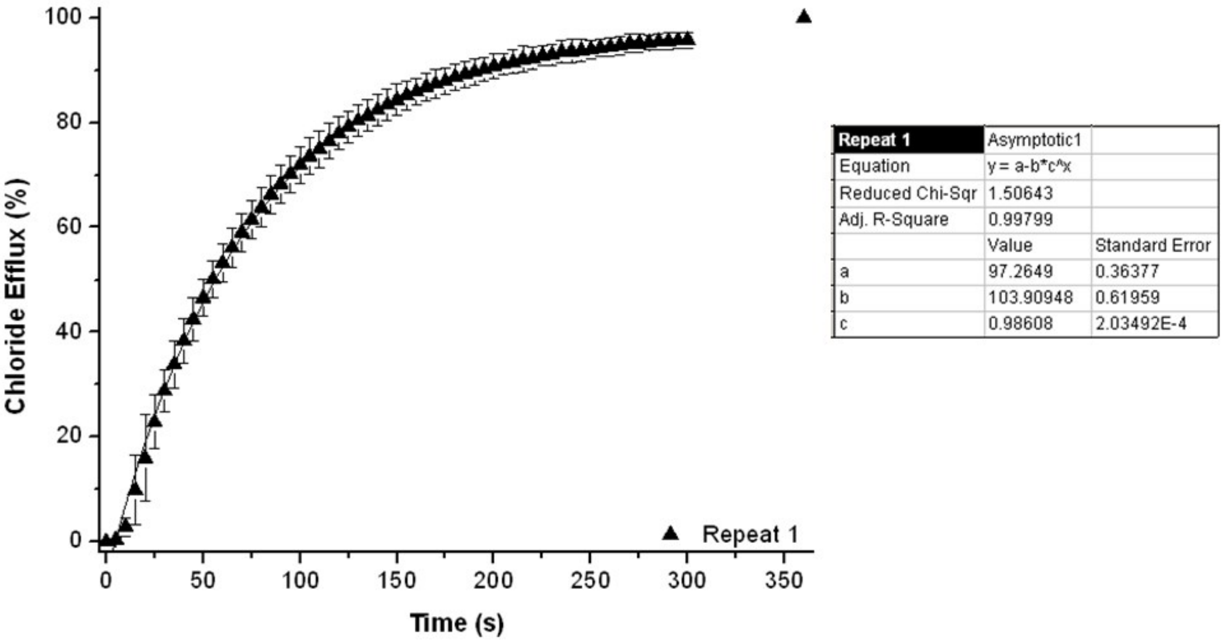

Repeat 1	Asymptotic1	
Equation	y = a-b*c^x	
Reduced Chi-Sqr	1.50643	
Adj. R-Square	0.99799	
	Value	Standard Error
a	97.2649	0.36377
b	103.90948	0.61959
c	0.98608	2.03492E-4

Figure S102. Overview of the initial rate of chloride release (k_{ini}) for compound **24**. For experimental details, see main text.

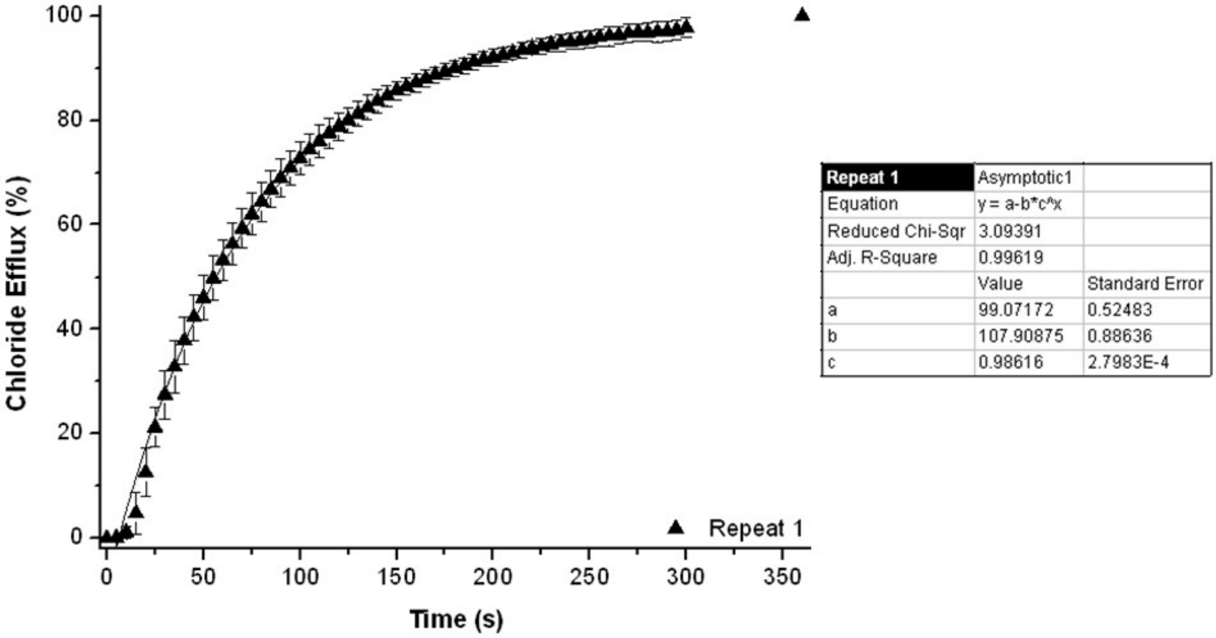

Repeat 1	Asymptotic1	
Equation	y = a-b*c^x	
Reduced Chi-Sqr	3.09391	
Adj. R-Square	0.99619	
	Value	Standard Error
a	99.07172	0.52483
b	107.90875	0.88636
c	0.98616	2.7983E-4

Figure S103. Overview of the initial rate of chloride release (k_{ini}) for compound **25**. For experimental details, see main text.

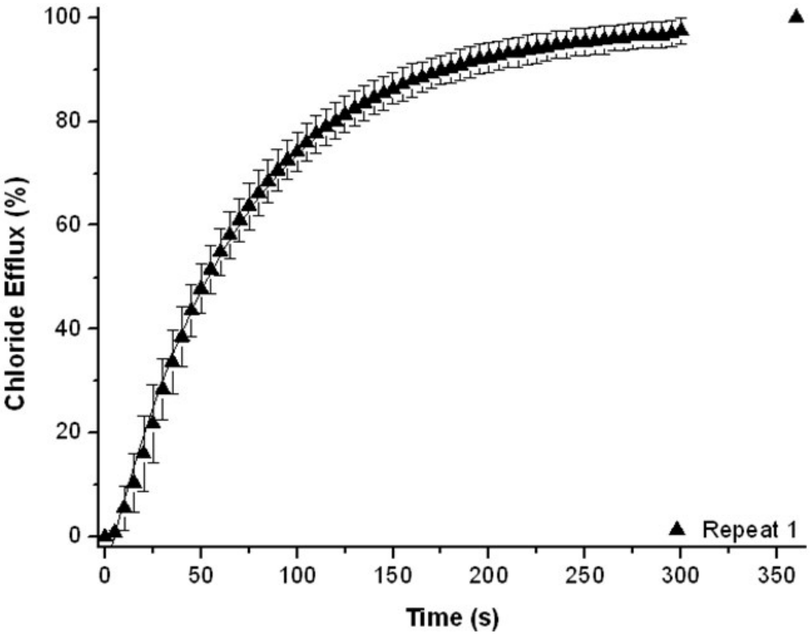

Repeat 1	Asymptotic1	
Equation	y = a-b*c^x	
Reduced Chi-Sqr	1.84907	
Adj. R-Square	0.9976	
	Value	Standard Error
a	98.56497	0.38997
b	105.58521	0.69281
c	0.98567	2.25926E-4

Figure S104. Overview of the initial rate of chloride release (k_{ini}) for compound **26**. For experimental details, see main text.

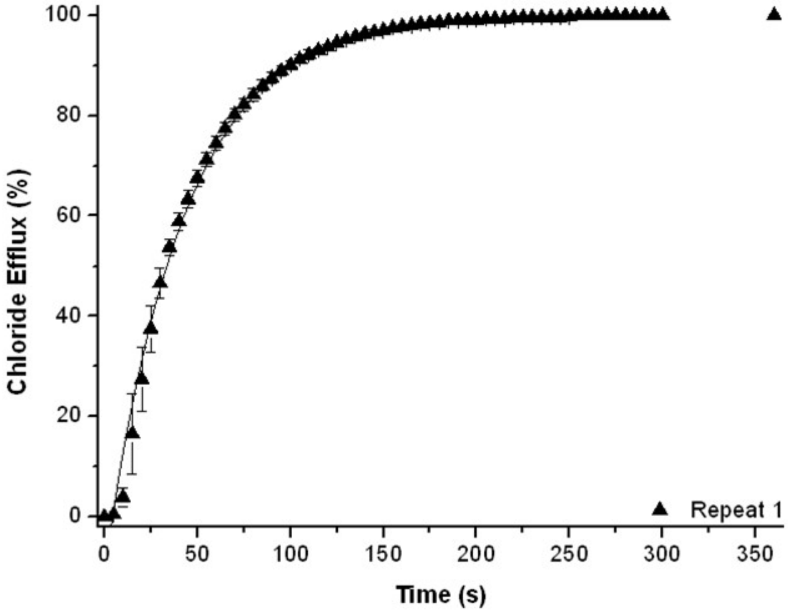

Repeat 1	Asymptotic1	
Equation	y = a-b*c^x	
Reduced Chi-Sqr	4.51312	
Adj. R-Square	0.99365	
	Value	Standard Error
a	100.35266	0.40468
b	111.17412	1.30777
c	0.97661	5.1413E-4

Figure S105. Overview of the initial rate of chloride release (k_{ini}) for compound **27**. For experimental details, see main text.

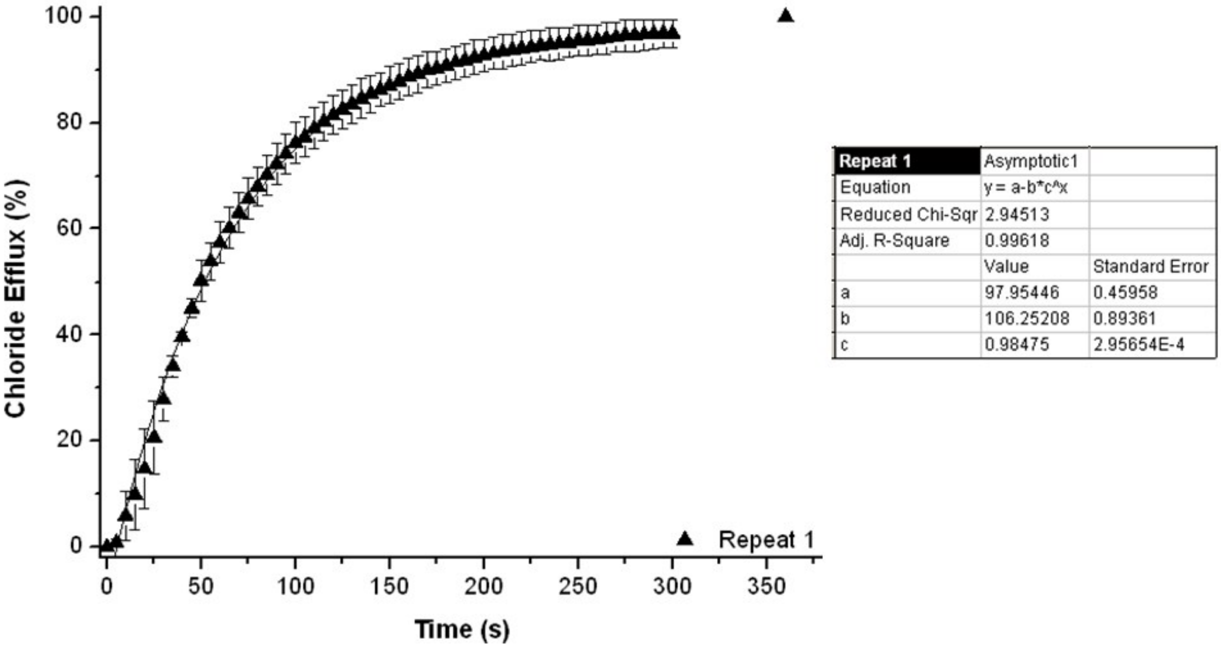

Figure S106. Overview of the initial rate of chloride release (k_{ini}) for compound **28**. For experimental details, see main text.

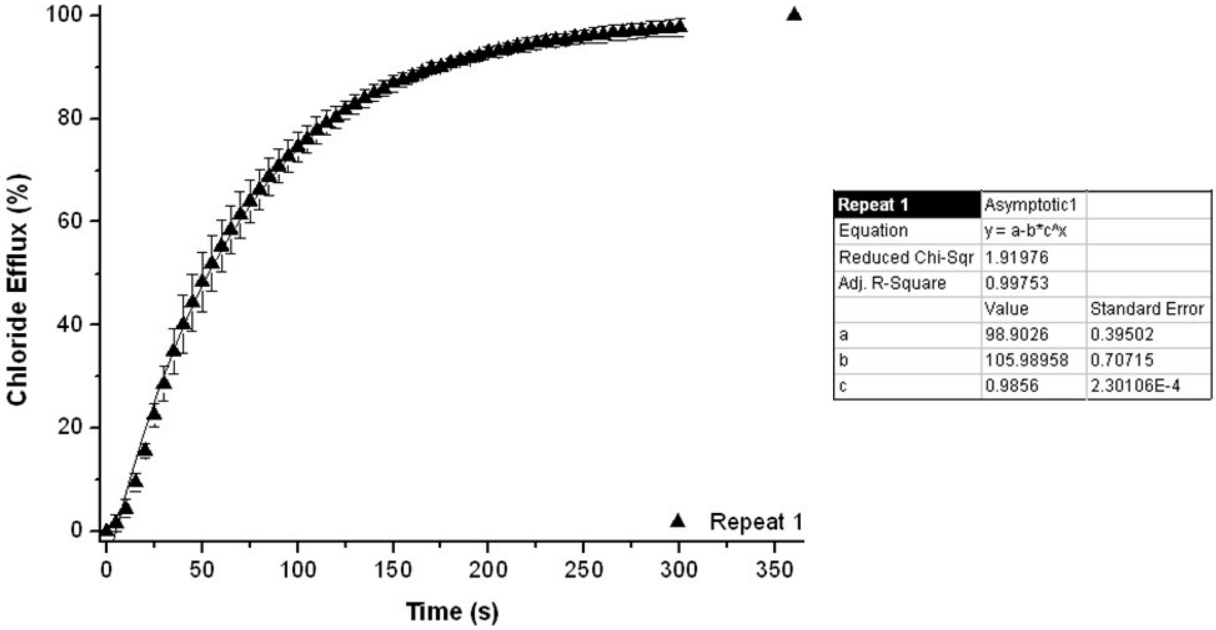

Figure S107. Overview of the initial rate of chloride release (k_{ini}) for compound **29**. For experimental details, see main text.

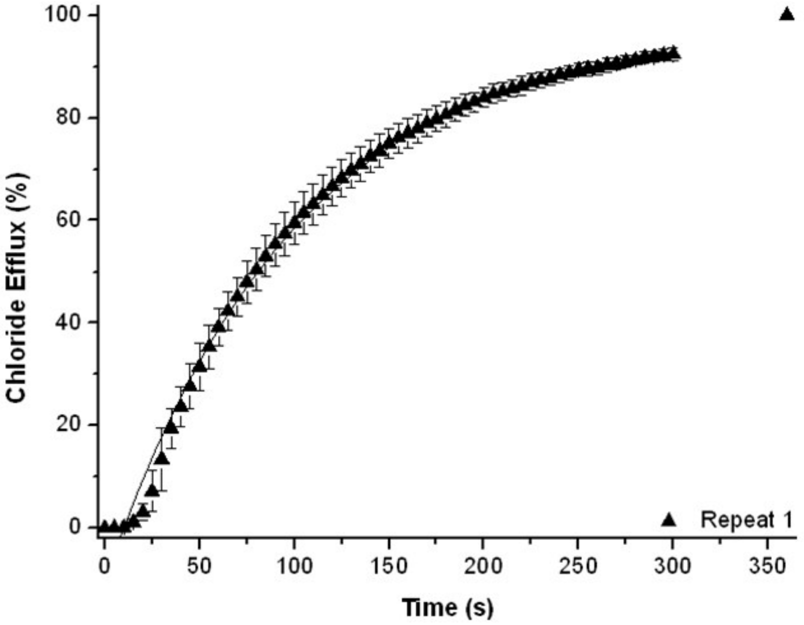

Repeat 1	Asymptotic1	
Equation	y = a-b*c^x	
Reduced Chi-Sqr	4.92182	
Adj. R-Square	0.99416	
	Value	Standard Error
a	98.64119	1.07576
b	109.04577	1.11093
c	0.99006	3.07865E-4

Figure S108. Overview of the initial rate of chloride release (k_{ini}) for compound **30**. For experimental details, see main text.

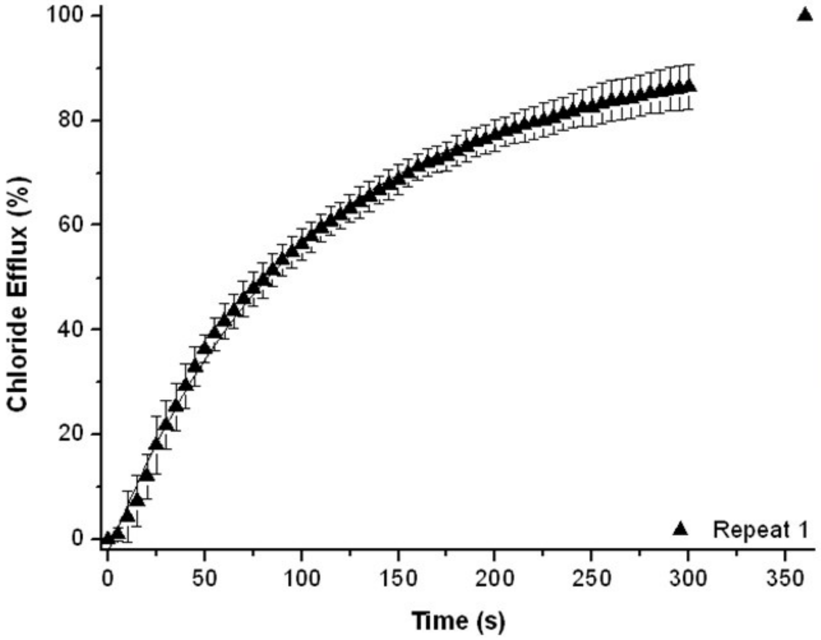

Repeat 1	Asymptotic1	
Equation	y = a-b*c^x	
Reduced Chi-Sqr	1.17466	
Adj. R-Square	0.99805	
	Value	Standard Error
a	89.79033	0.49316
b	92.28417	0.5319
c	0.98986	1.75176E-4

Figure S109. Overview of the initial rate of chloride release (k_{ini}) for compound **31**. For experimental details, see main text.

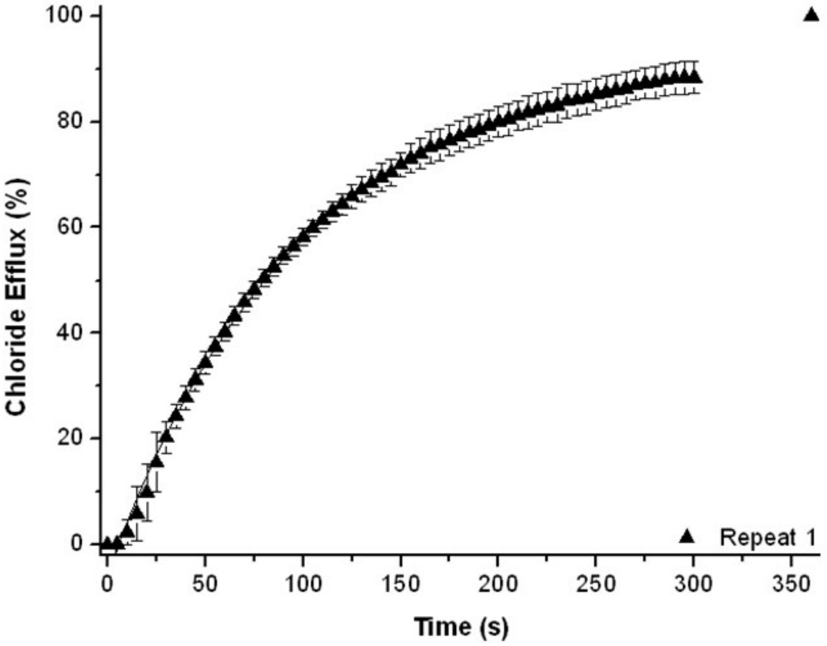

Repeat 1	Asymptotic1	
Equation	y = a-b*c^x	
Reduced Chi-Sqr	1.01923	
Adj. R-Square	0.9985	
	Value	Standard Error
a	92.70083	0.44888
b	98.08471	0.49358
c	0.98971	1.54228E-4

Figure S110. Overview of the initial rate of chloride release (k_{ini}) for compound **32**. For experimental details, see main text.

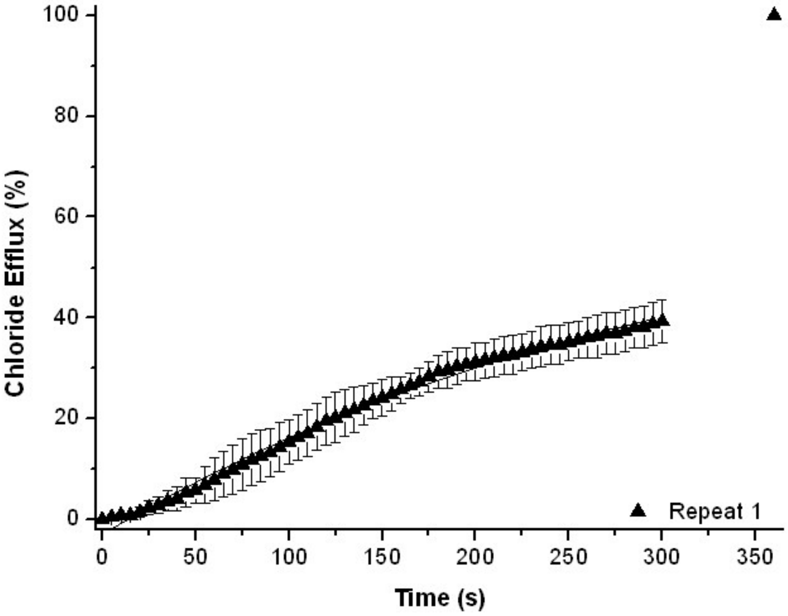

Repeat 1	Asymptotic1	
Equation	y = a-b*c^x	
Reduced Chi-Sqr	1.07228	
Adj. R-Square	0.99328	
	Value	Standard Error
a	67.41148	4.04937
b	70.39749	3.78628
c	0.99683	2.81761E-4

Figure S111. Overview of the initial rate of chloride release (k_{ini}) for compound **33**. For experimental details, see main text.

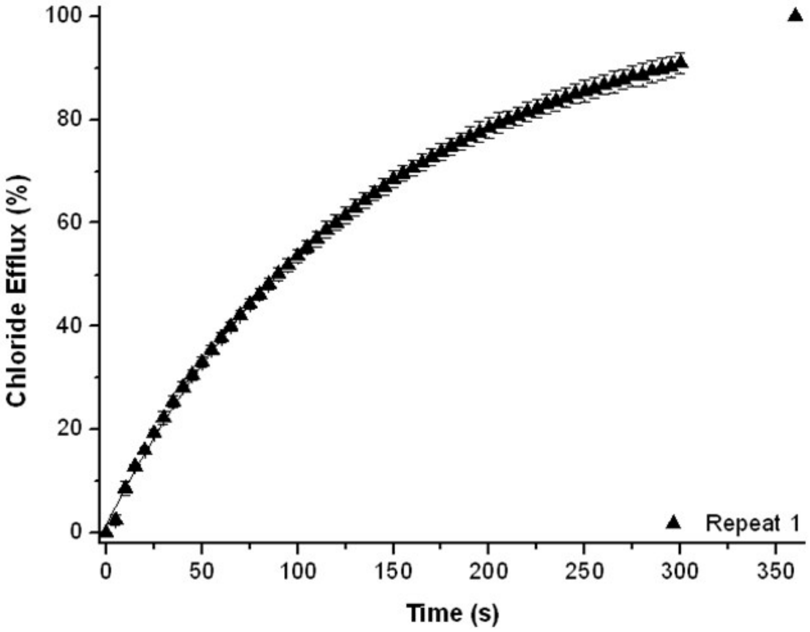

Repeat 1	Asymptotic1	
Equation	y = a-b*c^x	
Reduced Chi-Sqr	0.30449	
Adj. R-Square	0.99953	
	Value	Standard Error
a	100.83331	0.41452
b	99.33124	0.35187
c	0.99254	7.93177E-5

Figure S112. Overview of the initial rate of chloride release (k_{ini}) for compound **34**. For experimental details, see main text.

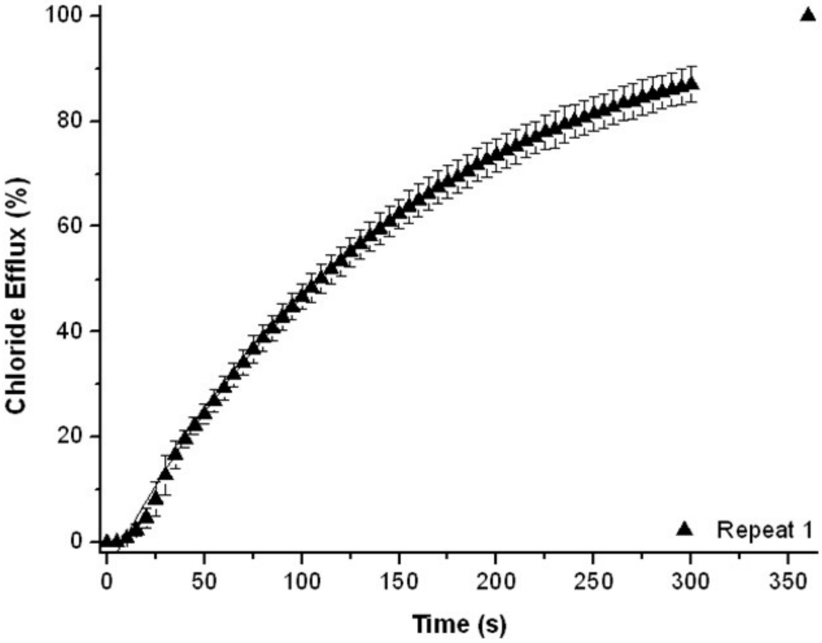

Repeat 1	Asymptotic1	
Equation	y = a-b*c^x	
Reduced Chi-Sqr	1.03576	
Adj. R-Square	0.99856	
	Value	Standard Error
a	102.47027	0.96589
b	107.93235	0.80519
c	0.99346	1.35596E-4

Figure S113. Overview of the initial rate of chloride release (k_{ini}) for compound **35**. For experimental details, see main text.

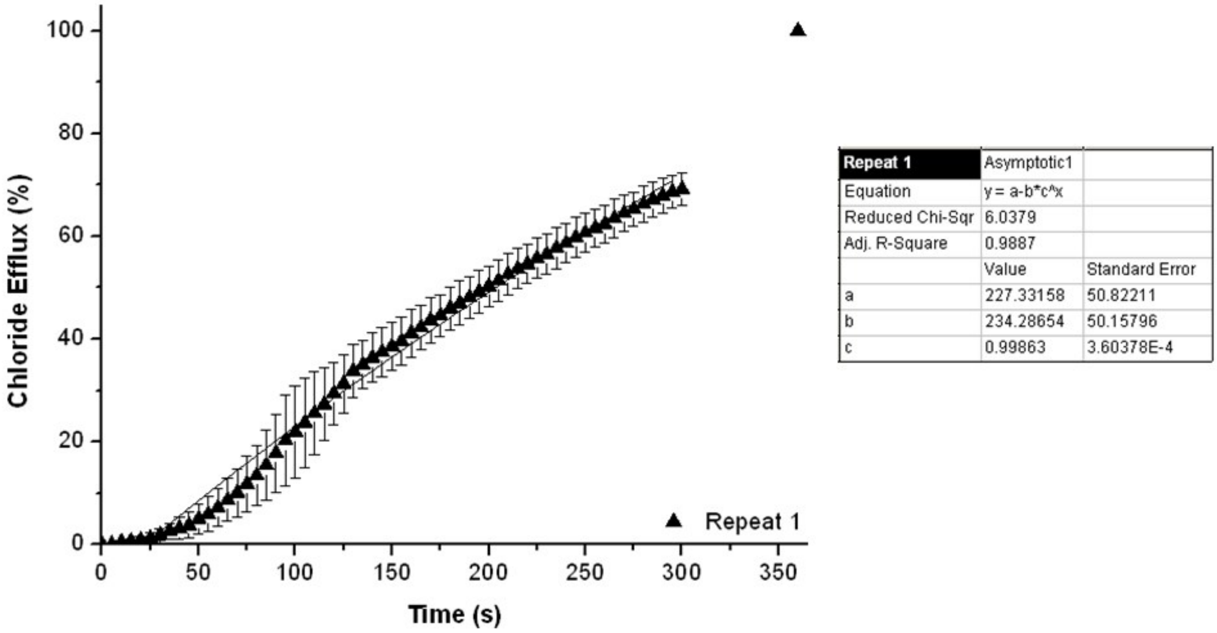

Repeat 1	Asymptotic1	
Equation	y = a-b*c^x	
Reduced Chi-Sqr	6.0379	
Adj. R-Square	0.9887	
	Value	Standard Error
a	227.33158	50.82211
b	234.28654	50.15796
c	0.99863	3.60378E-4

Figure S114. Overview of the initial rate of chloride release (k_{ini}) for compound **36**. For experimental details, see main text.

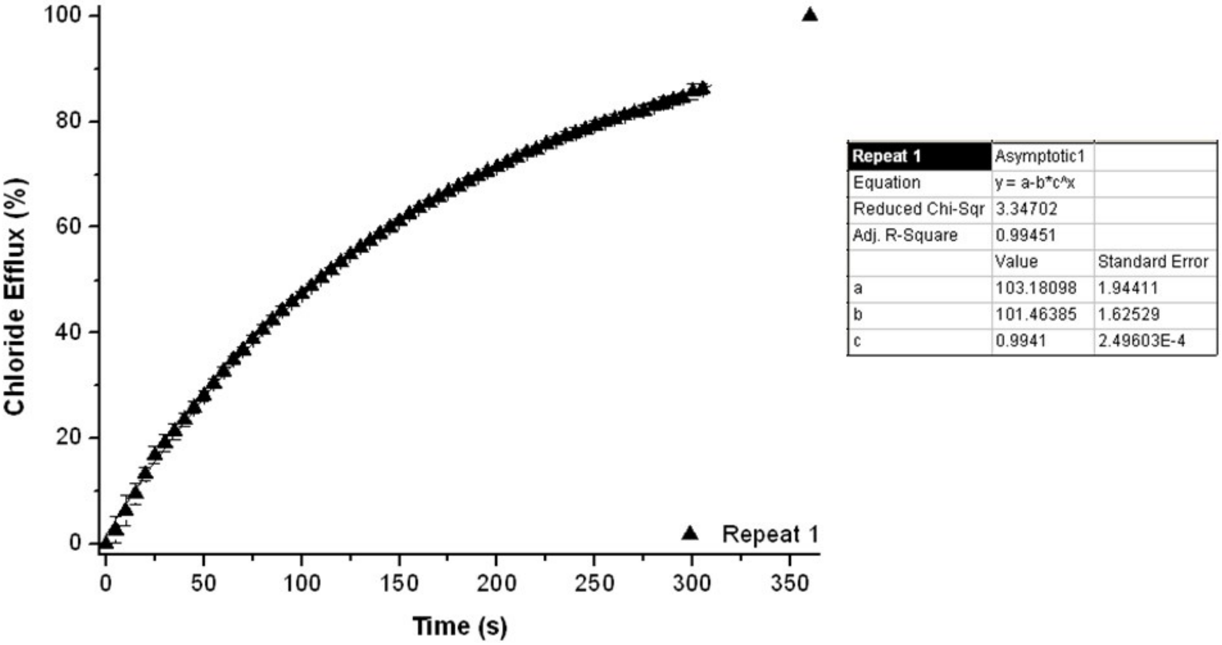

Repeat 1	Asymptotic1	
Equation	y = a-b*c^x	
Reduced Chi-Sqr	3.34702	
Adj. R-Square	0.99451	
	Value	Standard Error
a	103.18098	1.94411
b	101.46385	1.62529
c	0.9941	2.49603E-4

Figure S115. Overview of the initial rate of chloride release (k_{ini}) for compound **37**. For experimental details, see main text.

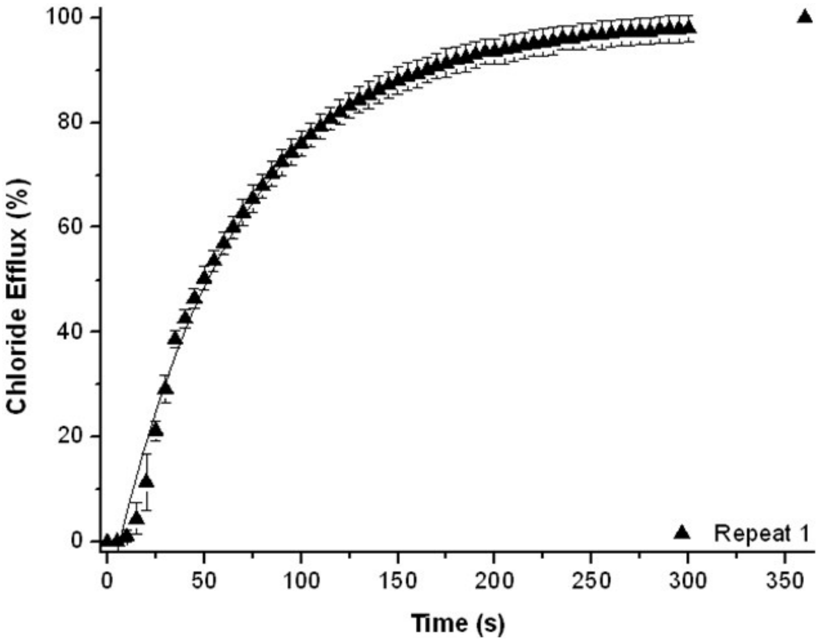

Repeat 1	Asymptotic1	
Equation	y = a-b*c^x	
Reduced Chi-Sqr	5.05922	
Adj. R-Square	0.99377	
	Value	Standard Error
a	98.8967	0.59644
b	109.08472	1.17524
c	0.9846	3.7999E-4

Figure S116. Overview of the initial rate of chloride release (k_{ini}) for compound **38**. For experimental details, see main text.

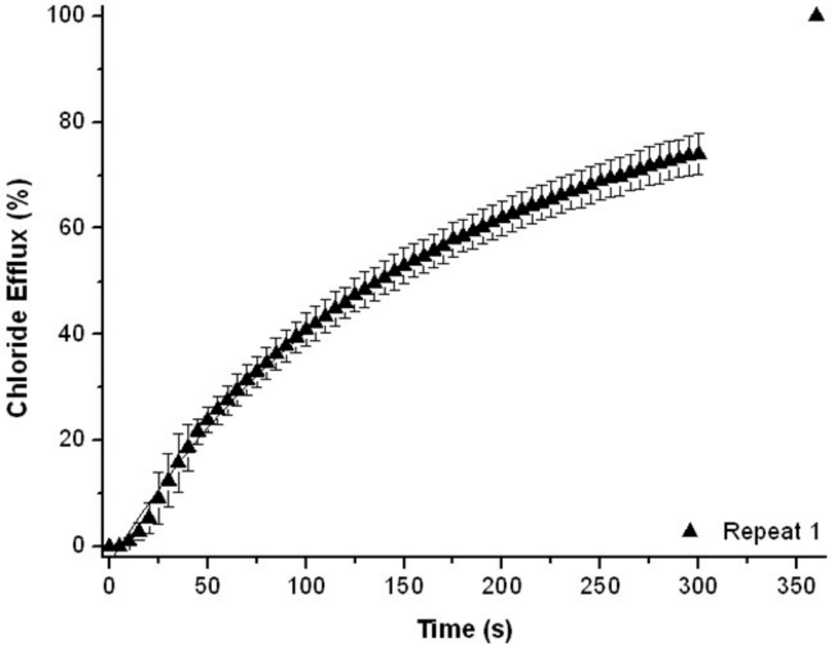

Repeat 1	Asymptotic1	
Equation	y = a-b*c^x	
Reduced Chi-Sqr	0.85197	
Adj. R-Square	0.99822	
	Value	Standard Error
a	84.22209	0.82608
b	87.35702	0.68976
c	0.9931	1.5516E-4

Figure S117. Overview of the initial rate of chloride release (k_{ini}) for compound **39**. For experimental details, see main text.

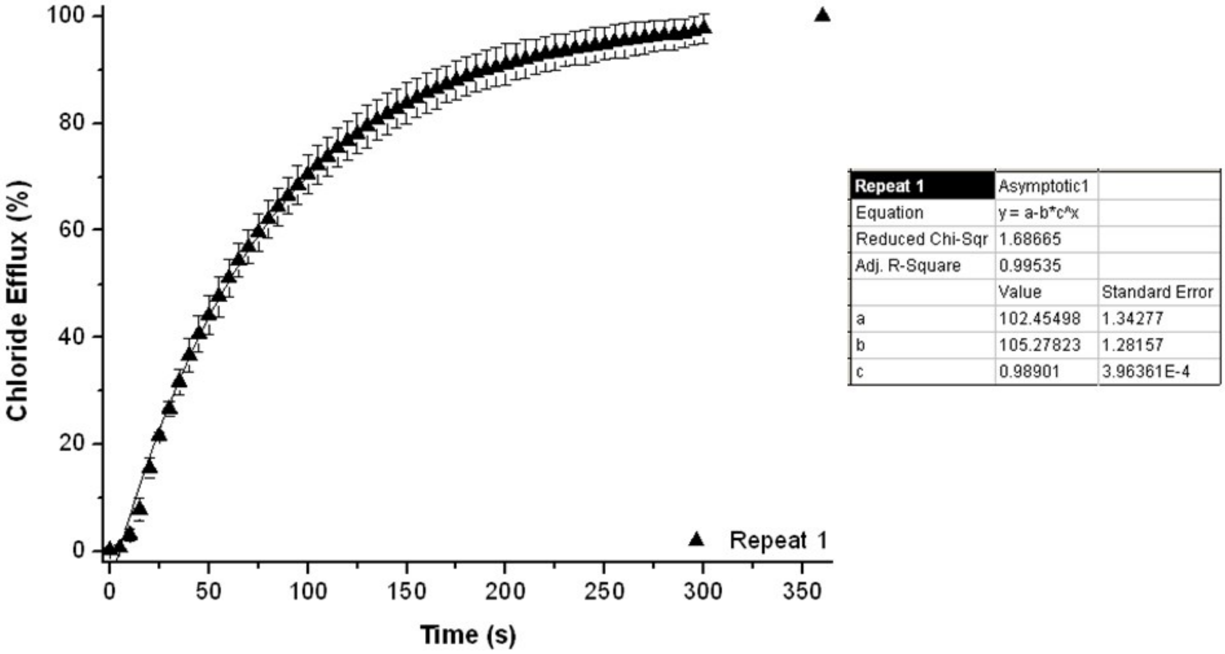

Figure S118. Overview of the initial rate of chloride release (k_{ini}) for compound **40**. For experimental details, see main text.

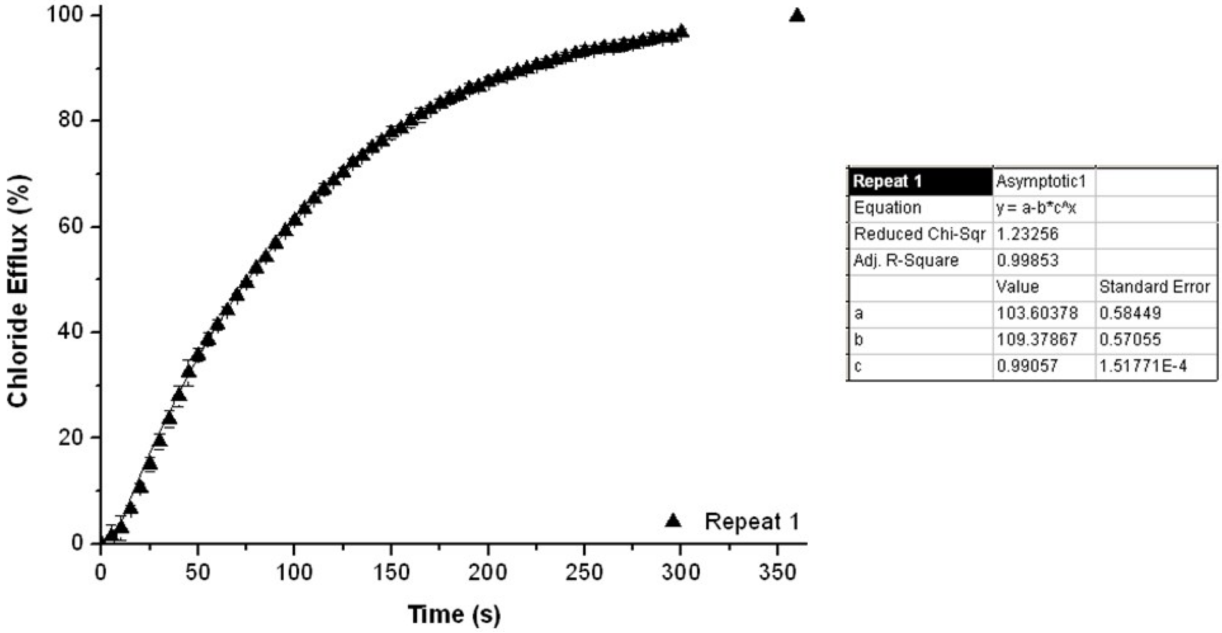

Figure S119. Overview of the initial rate of chloride release (k_{ini}) for compound **41**. For experimental details, see main text.

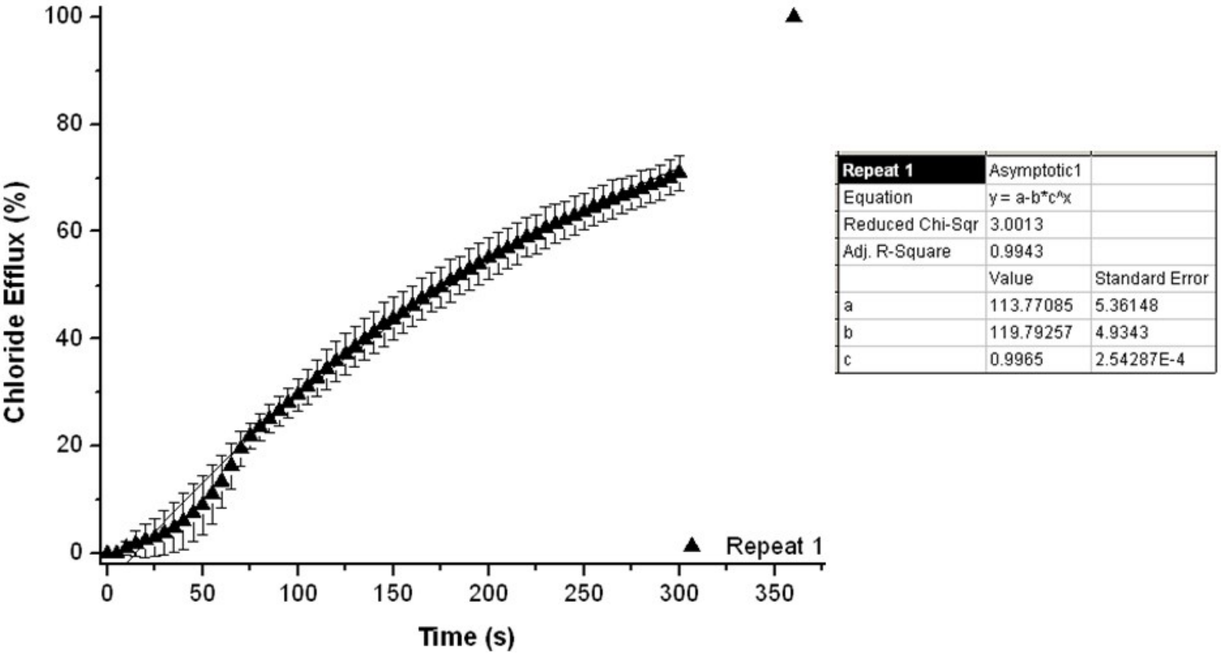

Figure S120. Overview of the initial rate of chloride release (k_{ini}) for compound **42**. For experimental details, see main text.

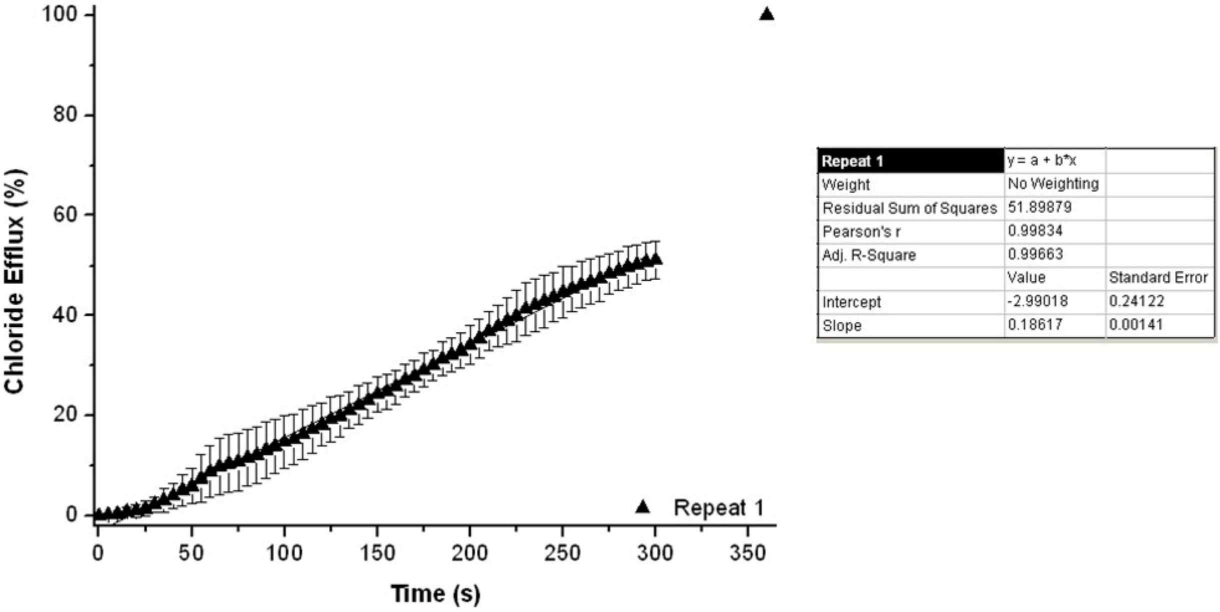

Figure S121. Overview of the initial rate of chloride release (k_{ini}) for compound **43**. For experimental details, see main text.

2.4. Hill plot analyses. EC_{50} determination

Chloride/nitrate transport assays were carried out using various concentrations of carriers as described. The chloride efflux (%) at 290 s was plotted as a function of the carrier concentration and the obtained results fitted with the Hill equation using Origin 8.1:

$$y = V_{máx} \cdot \frac{x^n}{k^n + x^n} = 100\% \cdot \frac{x^n}{(EC_{50})^n + x^n}$$

where x is the anion carrier concentration, V_{max} is the maximum chloride efflux (100%), y is the chloride efflux at 290 s (%), n is the Hill coefficient and k is the anion carrier concentration needed to achieve $V_{max}/2$ (when V_{max} is fixed to 100%, k equals EC_{50}); k and n are the parameters to be fitted. EC_{50}, defined as the anion carrier concentration (molar % carrier to lipid) needed to induce 50% release of the total of the chloride encapsulated in the time scale of our experiments, can be obtained directly from the graphs. Figures S122-S164 show all of the obtained transport data. An overview of the EC_{50} values can be found in Table S1 in S91.

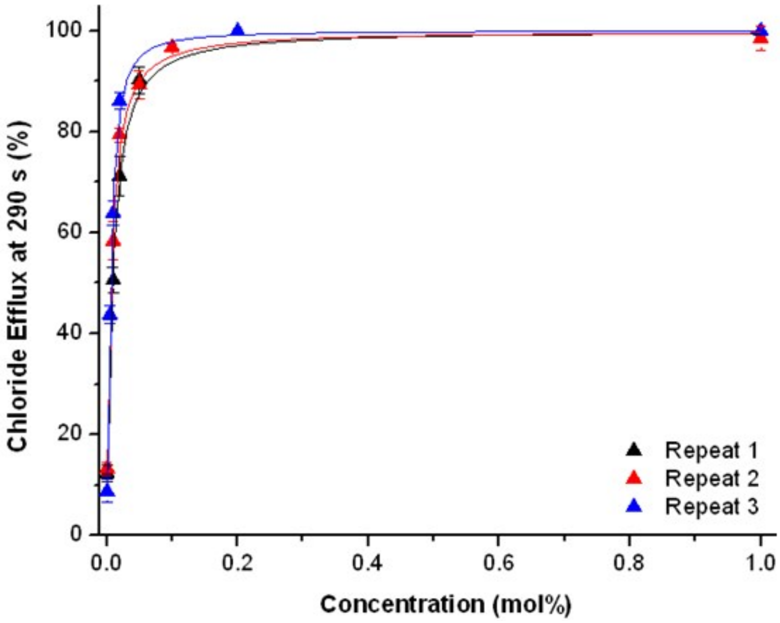

Repeat 1	Hill	
Equation	y=Vmax*x^n/(k^n+x^n)	
Reduced Chi-Sqr	5.31049	
Adj. R-Square	0.99501	
	Value	Standard Error
Vmax	100	0
EC_{50}	0.00659	4.93957E-4
n	1.08188	0.07244

Repeat 2	Hill	
Equation	y=Vmax*x^n/(k^n+x^n)	
Reduced Chi-Sqr	10.92251	
Adj. R-Square	0.99077	
	Value	Standard Error
Vmax	100	0
EC_{50}	0.00889	8.5596E-4
n	1.12442	0.12086

Repeat 3	Hill	
Equation	y=Vmax*x^n/(k^n+x^n)	
Reduced Chi-Sqr	3.57765	
Adj. R-Square	0.99724	
	Value	Standard Error
Vmax	100	0
EC_{50}	0.0061	2.40192E-4
n	1.3737	0.08196

Figure S122. Overview of the Hill plots for compound **1**. For experimental details, see main text.

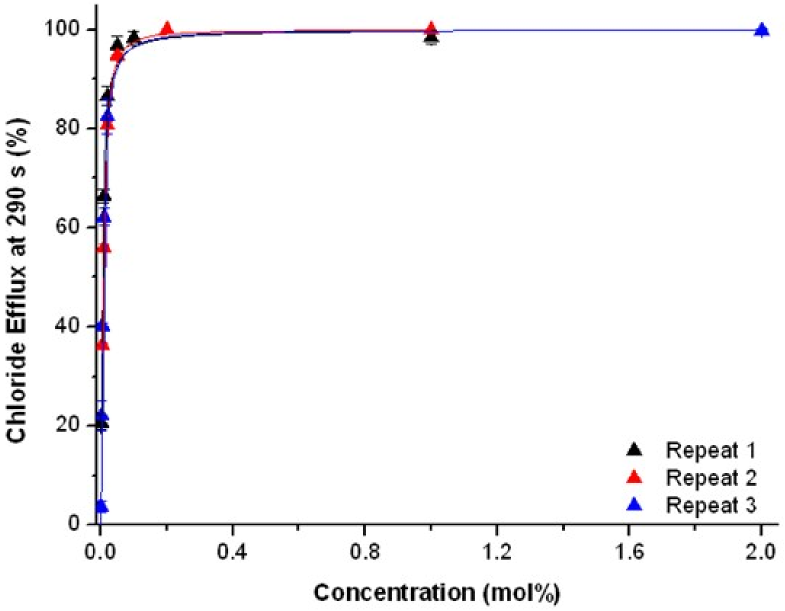

Repeat 1	Hill	
Equation	y=Vmax*x^n/(k^n+x^n)	
Reduced Chi-Sqr	3.79903	
Adj. R-Square	0.99455	
	Value	Standard Error
Vmax	100	0
EC_{50}	0.00781	2.94103E-4
n	1.44234	0.09034

Repeat 2	Hill	
Equation	y=Vmax*x^n/(k^n+x^n)	
Reduced Chi-Sqr	14.08731	
Adj. R-Square	0.98493	
	Value	Standard Error
Vmax	100	0
EC_{50}	0.00416	5.49134E-4
n	1.04295	0.10179

Repeat 3	Hill	
Equation	y=Vmax*x^n/(k^n+x^n)	
Reduced Chi-Sqr	8.16403	
Adj. R-Square	0.9939	
	Value	Standard Error
Vmax	100	0
EC_{50}	0.00642	3.72818E-4
n	1.21948	0.10087

Figure S123. Overview of the Hill plots for compound **2**. For experimental details, see main text.

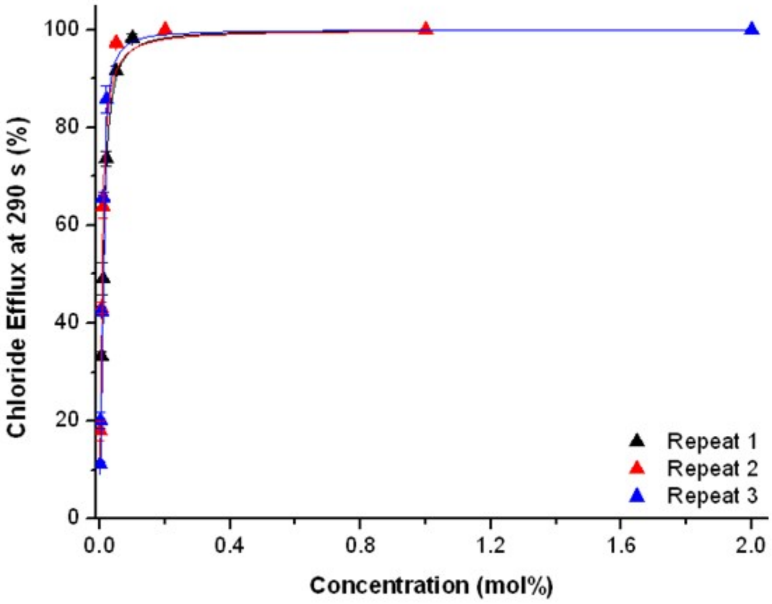

Repeat 1	Hill	
Equation	y=Vmax*x^n/(k^n+x^n)	
Reduced Chi-Sqr	19.00154	
Adj. R-Square	0.98421	
	Value	Standard Error
Vmax	100	0
EC_{50}	0.00573	6.25008E-4
n	1.10757	0.1494

Repeat 2	Hill	
Equation	y=Vmax*x^n/(k^n+x^n)	
Reduced Chi-Sqr	6.72559	
Adj. R-Square	0.99129	
	Value	Standard Error
Vmax	100	0
EC_{50}	0.0093	4.72154E-4
n	1.33186	0.10042

Repeat 3	Hill	
Equation	y=Vmax*x^n/(k^n+x^n)	
Reduced Chi-Sqr	4.4425	
Adj. R-Square	0.99652	
	Value	Standard Error
Vmax	100	0
EC_{50}	0.00593	2.37438E-4
n	1.30967	0.06872

Figure S124. Overview of the Hill plots for compound **3**. For experimental details, see main text.

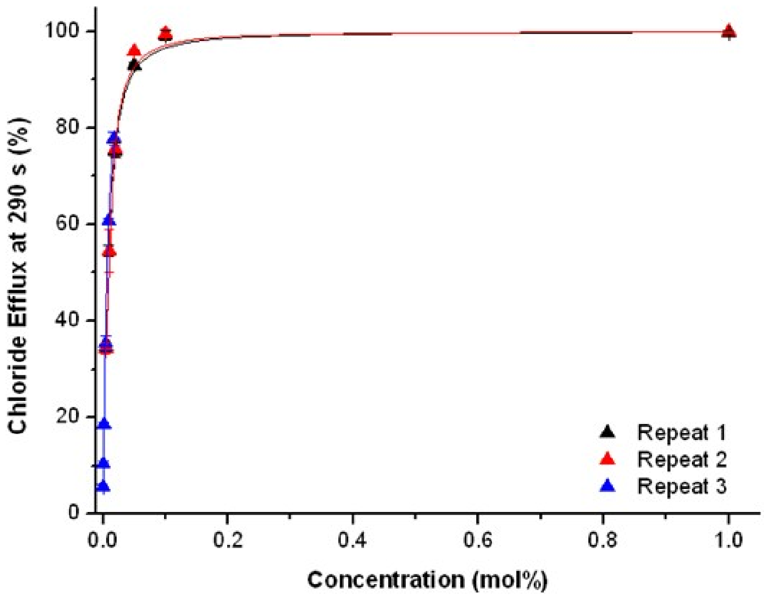

Repeat 1	Hill	
Equation	y=Vmax*x^n/(k^n+x^n)	
Reduced Chi-Sqr	3.27498	
Adj. R-Square	0.99541	
	Value	Standard Error
Vmax	100	0
EC_{50}	0.00841	3.02516E-4
n	1.35033	0.0739

Repeat 2	Hill	
Equation	y=Vmax*x^n/(k^n+x^n)	
Reduced Chi-Sqr	6.45893	
Adj. R-Square	0.99134	
	Value	Standard Error
Vmax	100	0
EC_{50}	0.0084	4.07134E-4
n	1.2016	0.1098

Repeat 3	Hill	
Equation	y=Vmax*x^n/(k^n+x^n)	
Reduced Chi-Sqr	5.57725	
Adj. R-Square	0.99343	
	Value	Standard Error
Vmax	100	0
EC_{50}	0.00656	3.12255E-4
n	1.0056	0.06974

Figure S125. Overview of the Hill plots for compound **4**. For experimental details, see main text.

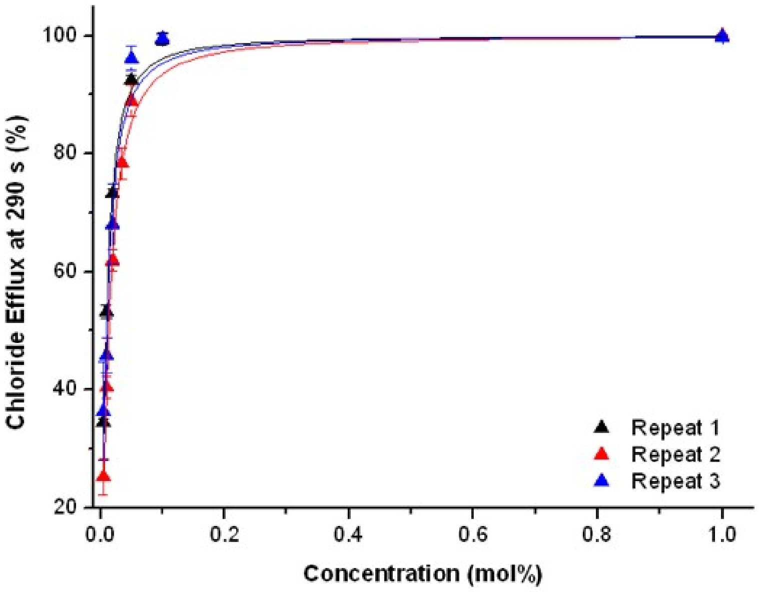

Repeat 1	Hill	
Equation	y=Vmax*x^n/(k^n+x^n)	
Reduced Chi-Sqr	5.48225	
Adj. R-Square	0.99246	
	Value	Standard Error
Vmax	100	0
EC_{50}	0.00866	4.08901E-4
n	1.2932	0.0917

Repeat 2	Hill	
Equation	y=Vmax*x^n/(k^n+x^n)	
Reduced Chi-Sqr	6.5851	
Adj. R-Square	0.99208	
	Value	Standard Error
Vmax	100	0
EC_{50}	0.01282	6.00013E-4
n	1.29861	0.08354

Repeat 3	Hill	
Equation	y=Vmax*x^n/(k^n+x^n)	
Reduced Chi-Sqr	37.08727	
Adj. R-Square	0.95419	
	Value	Standard Error
Vmax	100	0
EC_{50}	0.0096	0.00116
n	1.3142	0.22538

Figure S126. Overview of the Hill plots for compound **5**. For experimental details, see main text.

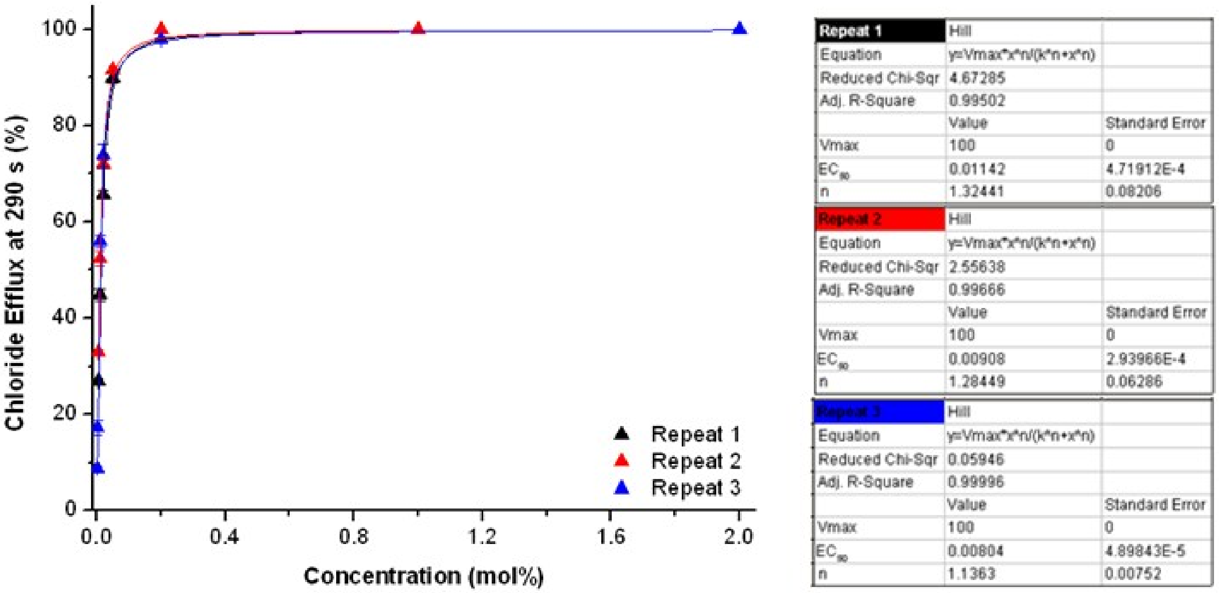

Repeat 1	Hill	
Equation	y=Vmax*x^n/(k^n+x^n)	
Reduced Chi-Sqr	4.67285	
Adj. R-Square	0.99502	
	Value	Standard Error
Vmax	100	0
EC_{50}	0.01142	4.71912E-4
n	1.32441	0.08206

Repeat 2	Hill	
Equation	y=Vmax*x^n/(k^n+x^n)	
Reduced Chi-Sqr	2.55638	
Adj. R-Square	0.99666	
	Value	Standard Error
Vmax	100	0
EC_{50}	0.00908	2.93966E-4
n	1.28449	0.06286

Repeat 3	Hill	
Equation	y=Vmax*x^n/(k^n+x^n)	
Reduced Chi-Sqr	0.05946	
Adj. R-Square	0.99996	
	Value	Standard Error
Vmax	100	0
EC_{50}	0.00804	4.89843E-5
n	1.1363	0.00752

Figure S127. Overview of the Hill plots for compound **6**. For experimental details, see main text.

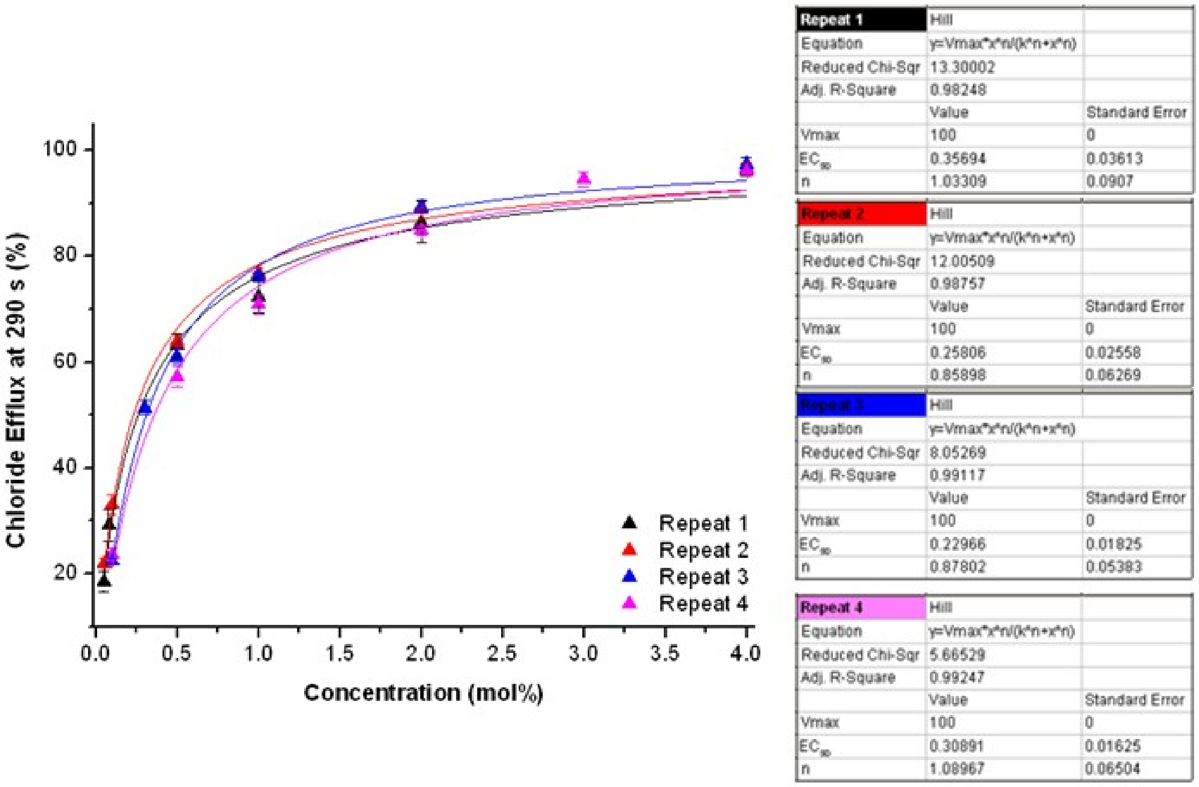

Repeat 1	Hill	
Equation	y=Vmax*x^n/(k^n+x^n)	
Reduced Chi-Sqr	13.30002	
Adj. R-Square	0.98248	
	Value	Standard Error
Vmax	100	0
EC_{50}	0.35694	0.03613
n	1.03309	0.0907

Repeat 2	Hill	
Equation	y=Vmax*x^n/(k^n+x^n)	
Reduced Chi-Sqr	12.00509	
Adj. R-Square	0.98757	
	Value	Standard Error
Vmax	100	0
EC_{50}	0.25806	0.02558
n	0.85898	0.06269

Repeat 3	Hill	
Equation	y=Vmax*x^n/(k^n+x^n)	
Reduced Chi-Sqr	8.05269	
Adj. R-Square	0.99117	
	Value	Standard Error
Vmax	100	0
EC_{50}	0.22966	0.01825
n	0.87802	0.05383

Repeat 4	Hill	
Equation	y=Vmax*x^n/(k^n+x^n)	
Reduced Chi-Sqr	5.66529	
Adj. R-Square	0.99247	
	Value	Standard Error
Vmax	100	0
EC_{50}	0.30891	0.01625
n	1.08967	0.06504

Figure S128. Overview of the Hill plots for compound **7**. For experimental details, see main text.

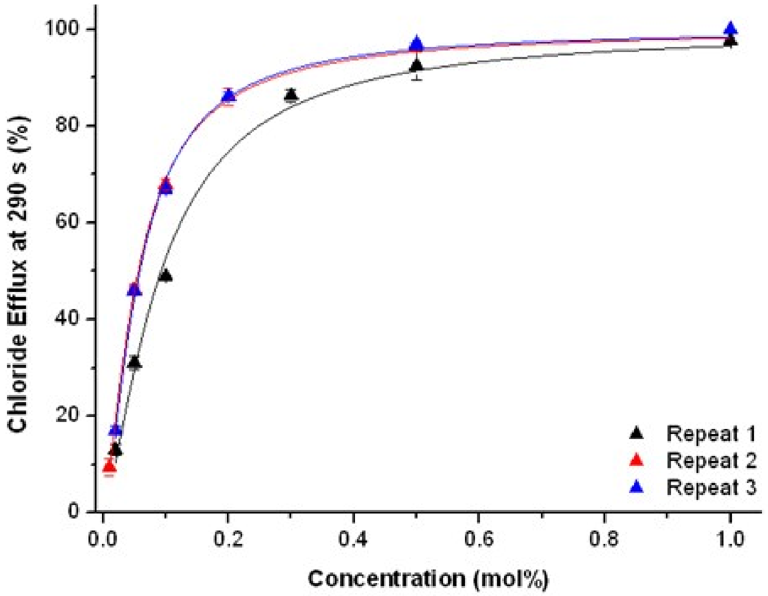

Repeat 1	Hill	
Equation	y=Vmax*x^n/(k^n+x^n)	
Reduced Chi-Sqr	1.76757	
Adj. R-Square	0.99854	
	Value	Standard Error
Vmax	100	0
EC_{50}	0.05599	0.00158
n	1.3884	0.05358
Repeat 2	Hill	
Equation	y=Vmax*x^n/(k^n+x^n)	
Reduced Chi-Sqr	7.62172	
Adj. R-Square	0.99399	
	Value	Standard Error
Vmax	100	0
EC_{50}	0.09242	0.00486
n	1.40538	0.09422
Repeat 3	Hill	
Equation	y=Vmax*x^n/(k^n+x^n)	
Reduced Chi-Sqr	2.38235	
Adj. R-Square	0.99775	
		Standard Error
Vmax	100	0
EC_{50}	0.05812	0.00164
n	1.46544	0.05931

Figure S129. Overview of the Hill plots for compound **8**. For experimental details, see main text.

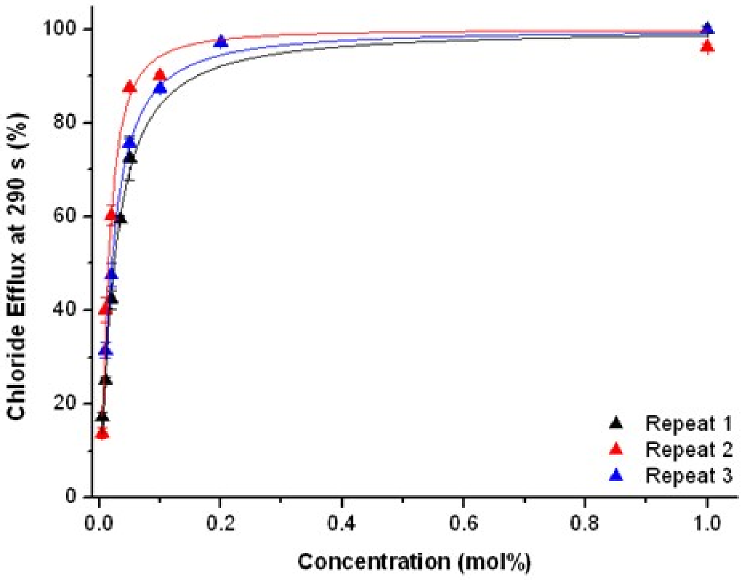

Repeat 1	Hill	
Equation	y=Vmax*x^n/(k^n+x^n)	
Reduced Chi-Sqr	6.95985	
Adj. R-Square	0.99276	
	Value	Standard Error
Vmax	100	0
EC_{50}	0.02426	0.00119
n	1.16701	0.08402
Repeat 2	Hill	
Equation	y=Vmax*x^n/(k^n+x^n)	
Reduced Chi-Sqr	15.00821	
Adj. R-Square	0.98607	
	Value	Standard Error
Vmax	100	0
EC_{50}	0.01456	0.00101
n	1.45527	0.14591
Repeat 3	Hill	
Equation	y=Vmax*x^n/(k^n+x^n)	
Reduced Chi-Sqr	4.19347	
Adj. R-Square	0.99464	
	Value	Standard Error
Vmax	100	0
EC_{50}	0.0202	8.66197E-4
n	1.24388	0.0686

Figure S130. Overview of the Hill plots for compound **9**. For experimental details, see main text.

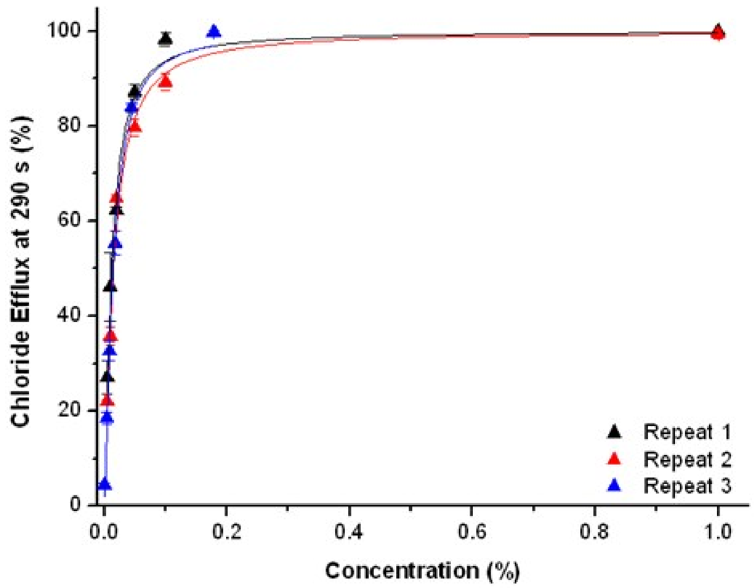

Repeat 1	Hill	
Equation	y=Vmax*x^n/(k^n+x^n)	
Reduced Chi-Sqr	10.39466	
Adj. R-Square	0.98838	
	Value	Standard Error
Vmax	100	0
EC_{50}	0.01166	7.3979E-4
n	1.26026	0.10955

Repeat 2	Hill	
Equation	y=Vmax*x^n/(k^n+x^n)	
Reduced Chi-Sqr	11.26535	
Adj. R-Square	0.988	
	Value	Standard Error
Vmax	100	0
EC_{50}	0.01443	9.69604E-4
n	1.20388	0.10321

Repeat 3	Hill	
Equation	y=Vmax*x^n/(k^n+x^n)	
Reduced Chi-Sqr	6.1138	
Adj. R-Square	0.99566	
	Value	Standard Error
Vmax	100	0
EC_{50}	0.0146	6.75053E-4
n	1.38397	0.09056

Figure S131. Overview of the Hill plots for compound **10**. For experimental details, see main text.

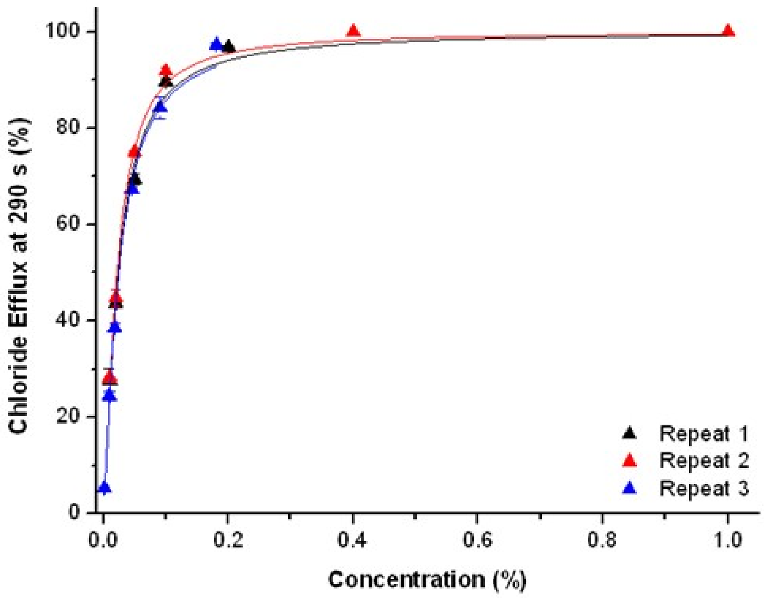

Repeat 1	Hill	
Equation	y=Vmax*x^n/(k^n+x^n)	
Reduced Chi-Sqr	6.09931	
Adj. R-Square	0.99339	
	Value	Standard Error
Vmax	100	0
EC_{50}	0.02165	0.00104
n	1.37868	0.09378

Repeat 2	Hill	
Equation	y=Vmax*x^n/(k^n+x^n)	
Reduced Chi-Sqr	9.11859	
Adj. R-Square	0.98984	
	Value	Standard Error
Vmax	100	0
EC_{50}	0.02336	0.00142
n	1.28918	0.10006

Repeat 3	Hill	
Equation	y=Vmax*x^n/(k^n+x^n)	
Reduced Chi-Sqr	8.70105	
Adj. R-Square	0.99325	
	Value	Standard Error
Vmax	100	0
EC_{50}	0.02439	0.00143
n	1.27516	0.09012

Figure S132. Overview of the Hill plots for compound **11**. For experimental details, see main text.

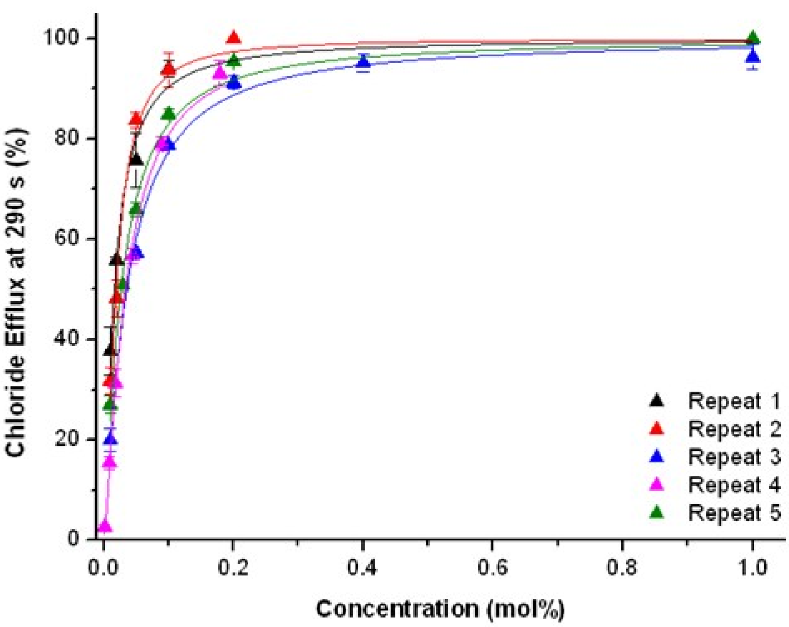

Repeat 1	Hill	
Equation	y=Vmax*x^n/(k^n+x^n)	
Reduced Chi-Sqr	13.84026	
Adj. R-Square	0.97928	
	Value	Standard Error
Vmax	100	0
EC_{50}	0.01605	0.00134
n	1.22613	0.13501

Repeat 2	Hill	
Equation	y=Vmax*x^n/(k^n+x^n)	
Reduced Chi-Sqr	5.58824	
Adj. R-Square	0.99374	
	Value	Standard Error
Vmax	100	0
EC_{50}	0.03534	0.00217
n	1.19223	0.0732

Repeat 3	Hill	
Equation	y=Vmax*x^n/(k^n+x^n)	
Reduced Chi-Sqr	4.3397	
Adj. R-Square	0.99663	
	Value	Standard Error
Vmax	100	0
EC_{50}	0.03363	0.00134
n	1.32737	0.06523

Repeat 5	Hill	
Equation	y=Vmax*x^n/(k^n+x^n)	
Reduced Chi-Sqr	10.57941	
Adj. R-Square	0.98678	
	Value	Standard Error
Vmax	100	0
EC_{50}	0.02657	0.00186
n	1.19799	0.10312

Repeat 4	Hill	
Equation	y=Vmax*x^n/(k^n+x^n)	
Reduced Chi-Sqr	11.3976	
Adj. R-Square	0.98661	
	Value	Standard Error
Vmax	100	0
EC_{50}	0.01855	0.00115
n	1.52177	0.14664

Figure S133. Overview of the Hill plots for compound **12**. For experimental details, see main text.

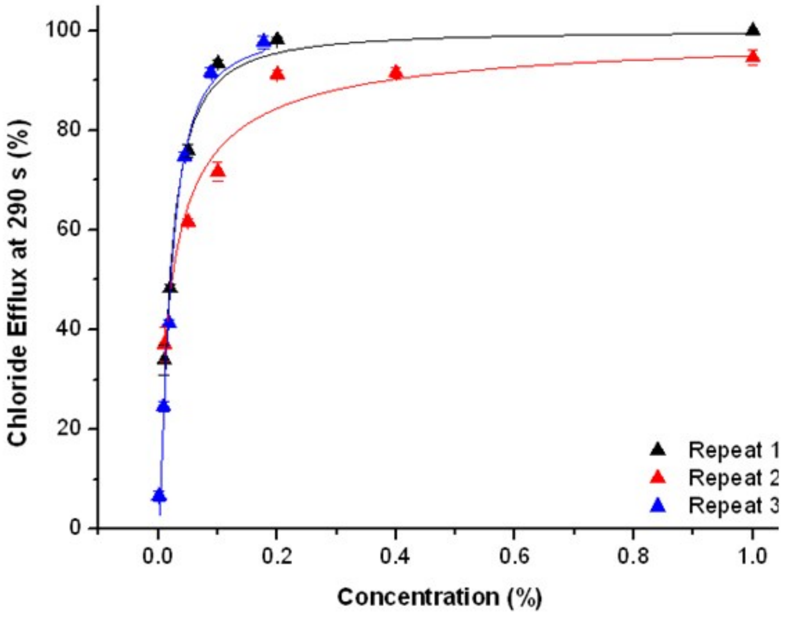

Repeat 1	Hill	
Equation	y=Vmax*x^n/(k^n+x^n)	
Reduced Chi-Sqr	12,36779	
Adj. R-Square	0,98418	
	Value	Standard Error
Vmax	100	0
EC_{50}	0,01894	0,00136
n	1,3006	0,12661

Repeat 2	Hill	
Equation	y=Vmax*x^n/(k^n+x^n)	
Reduced Chi-Sqr	20,57035	
Adj. R-Square	0,95964	
	Value	Standard Error
Vmax	100	0
EC_{50}	0,02259	0,0038
n	0,77631	0,09366

Repeat 3	Hill	
Equation	y=Vmax*x^n/(k^n+x^n)	
Reduced Chi-Sqr	9,54181	
Adj. R-Square	0,99319	
	Value	Standard Error
Vmax	100	0
EC_{50}	0,02077	0,00119
n	1,45963	0,11351

Figure S134. Overview of the Hill plots for compound **13**. For experimental details, see main text.

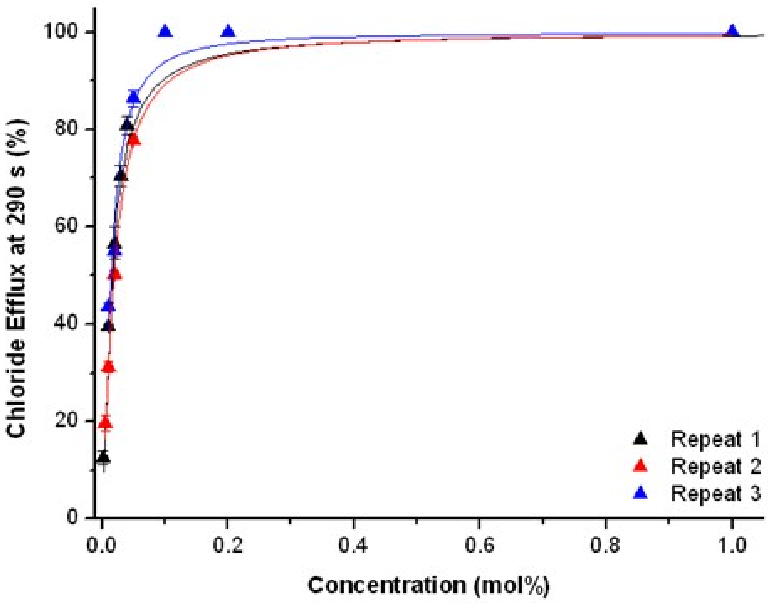

Repeat 1	Hill	
Equation	y=Vmax*x^n/(k^n+x^n)	
Reduced Chi-Sqr	29,78615	
Adj. R-Square	0,95327	
	Value	Standard Error
Vmax	100	0
EC_{50}	0,01395	0,00161
n	1,38855	0,24672

Repeat 2	Hill	
Equation	y=Vmax*x^n/(k^n+x^n)	
Reduced Chi-Sqr	9,96522	
Adj. R-Square	0,99172	
	Value	Standard Error
Vmax	100	0
EC_{50}	0,01843	0,00117
n	1,25231	0,10455

Repeat 3	Hill	
Equation	y=Vmax*x^n/(k^n+x^n)	
Reduced Chi-Sqr	9,14205	
Adj. R-Square	0,99054	
	Value	Standard Error
Vmax	100	0
EC_{50}	0,01412	8,92549E-4
n	1,1548	0,1021

Figure S135. Overview of the Hill plots for compound **14**. For experimental details, see main text.

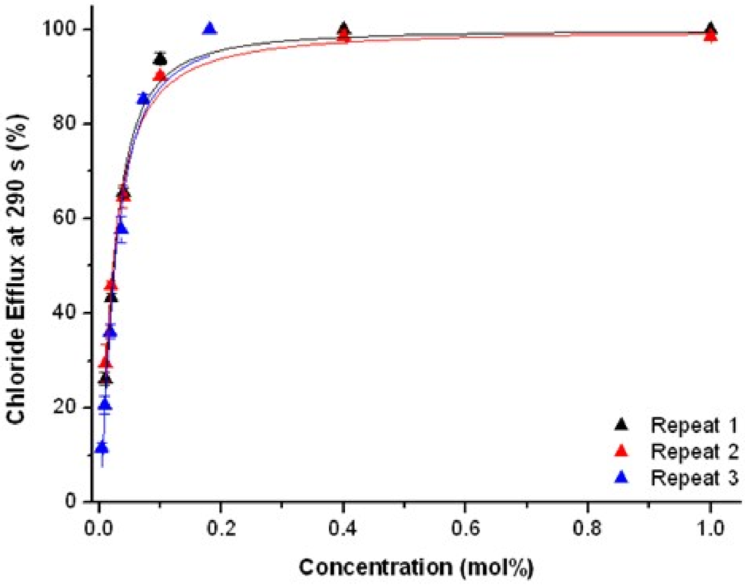

Repeat 1	Hill	
Equation	y=Vmax*x^n/(k^n+x^n)	
Reduced Chi-Sqr	11,68451	
Adj. R-Square	0,98834	
	Value	Standard Error
Vmax	100	0
EC_{50}	0,02315	0,00143
n	1,4328	0,13951

Repeat 2	Hill	
Equation	y=Vmax*x^n/(k^n+x^n)	
Reduced Chi-Sqr	7,12998	
Adj. R-Square	0,99168	
	Value	Standard Error
Vmax	100	0
EC_{50}	0,02213	0,00119
n	1,23769	0,09522

Repeat 3	Hill	
Equation	y=Vmax*x^n/(k^n+x^n)	
Reduced Chi-Sqr	22,08933	
Adj. R-Square	0,98252	
	Value	Standard Error
Vmax	100	0
k	0,02559	0,00206
n	1,44738	0,16015

Figure S136. Overview of the Hill plots for compound **15**. For experimental details, see main text.

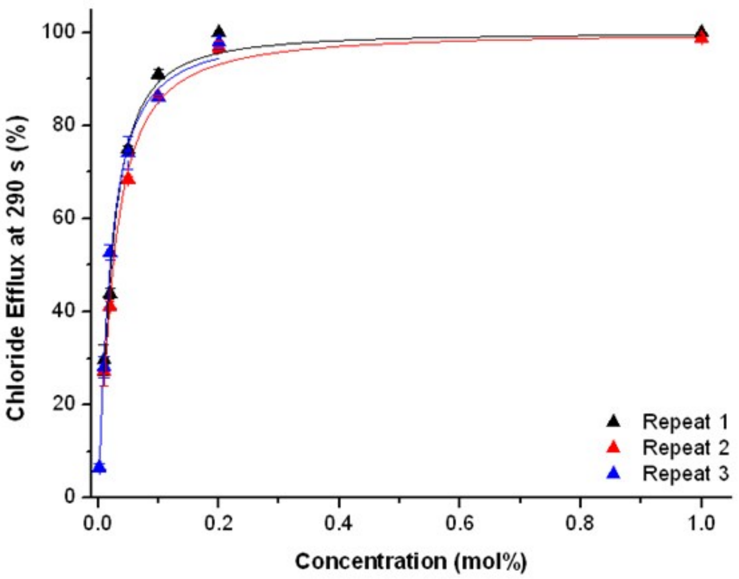

Repeat 1	Hill	
Equation	y=Vmax*x^n/(k^n+x^n)	
Reduced Chi-Sqr	13,01562	
Adj. R-Square	0,98559	
	Value	Standard Error
Vmax	100	0
EC_{50}	0,02156	0,0015
n	1,37972	0,13181

Repeat 2	Hill	
Equation	y=Vmax*x^n/(k^n+x^n)	
Reduced Chi-Sqr	8,1843	
Adj. R-Square	0,99088	
	Value	Standard Error
Vmax	100	0
EC_{50}	0,02488	0,00145
n	1,25293	0,09052

Repeat 3	Hill	
Equation	y=Vmax*x^n/(k^n+x^n)	
Reduced Chi-Sqr	7,11869	
Adj. R-Square	0,99427	
	Value	Standard Error
Vmax	100	0
EC_{50}	0,01992	0,0011
n	1,2302	0,08197

Figure S137. Overview of the Hill plots for compound **16**. For experimental details, see main text.

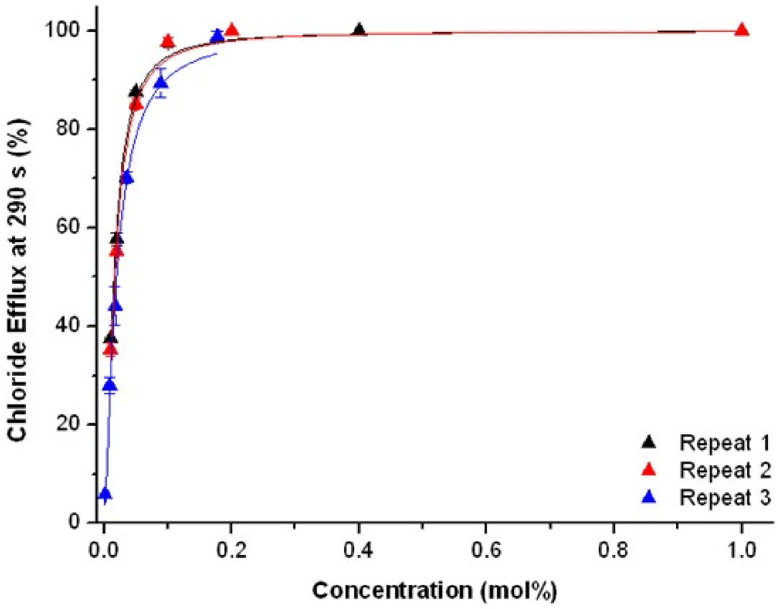

Repeat 1	Hill	
Equation	y=Vmax*x^n/(k^n+x^n)	
Reduced Chi-Sqr	6,47087	
Adj. R-Square	0,9907	
	Value	Standard Error
Vmax	100	0
EC_{50}	0,01491	7,21927E-4
n	1,53124	0,12712

Repeat 2	Hill	
Equation	y=Vmax*x^n/(k^n+x^n)	
Reduced Chi-Sqr	8,28266	
Adj. R-Square	0,98896	
	Value	Standard Error
Vmax	100	0
EC_{50}	0,01601	8,59309E-4
n	1,53541	0,13607

Repeat 3	Hill	
Equation	y=Vmax*x^n/(k^n+x^n)	
Reduced Chi-Sqr	7,7698	
Adj. R-Square	0,99412	
	Value	Standard Error
Vmax	100	0
EC_{50}	0,01929	9,99015E-4
n	1,36867	0,10029

Figure S138. Overview of the Hill plots for compound **17**. For experimental details, see main text.

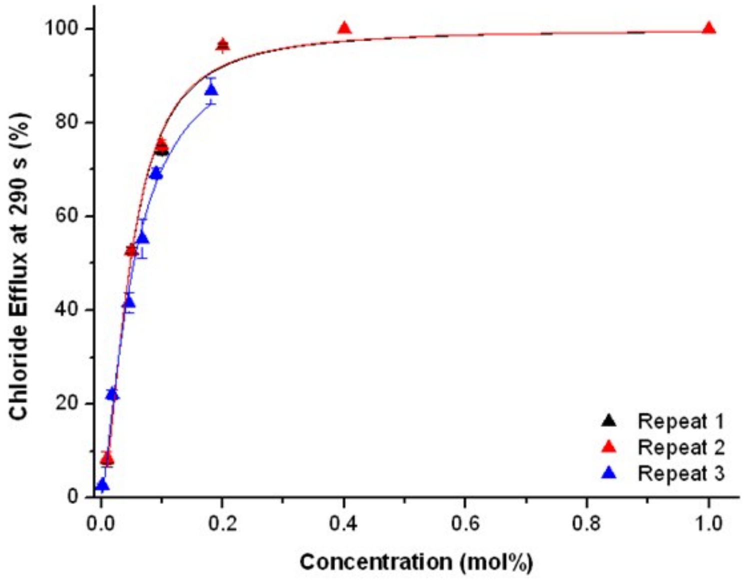

Repeat 1	Hill	
Equation	y=Vmax*x^n/(k^n+x^n)	
Reduced Chi-Sqr	10,9273	
Adj. R-Square	0,99174	
	Value	Standard Error
Vmax	100	0
EC_{50}	0,0475	0,00324
n	1,69769	0,18392

Repeat 2	Hill	
Equation	y=Vmax*x^n/(k^n+x^n)	
Reduced Chi-Sqr	9,65403	
Adj. R-Square	0,99268	
	Value	Standard Error
Vmax	100	0
EC_{50}	0,04725	0,00302
n	1,71367	0,17586

Repeat 3	Hill	
Equation	y=Vmax*x^n/(k^n+x^n)	
Reduced Chi-Sqr	10,53413	
Adj. R-Square	0,98892	
	Value	Standard Error
Vmax	100	0
EC_{50}	0,05345	0,00284
n	1,36293	0,12275

Figure S139. Overview of the Hill plots for compound **18**. For experimental details, see main text.

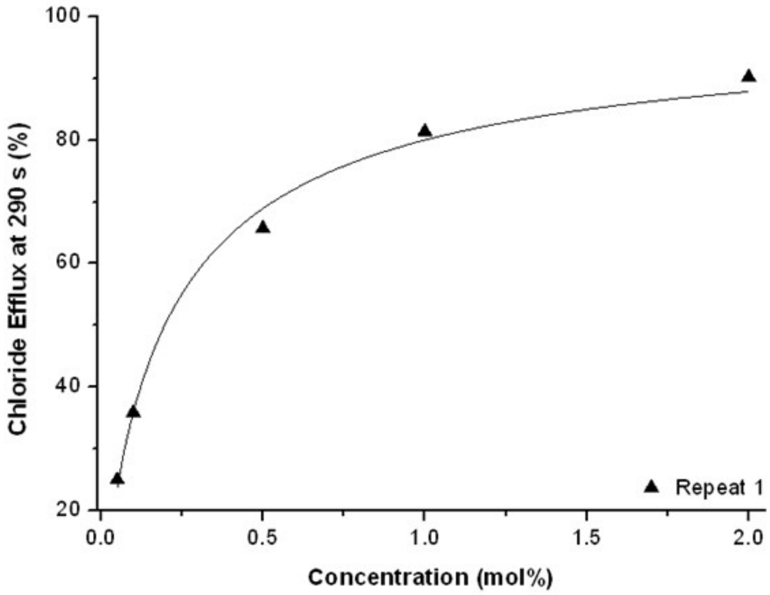

Repeat 1	Hill	
Equation	y=Vmax*x^n/(k^n+x^n)	
Reduced Chi-Sqr	6.47696	
Adj. R-Square	0.99193	
	Value	Standard Error
V_{max}	100	0
EC_{50}	0.19654	0.01443
n	0.85324	0.051

Figure S140. Overview of the Hill plots for compound **19**. For experimental details, see main text.

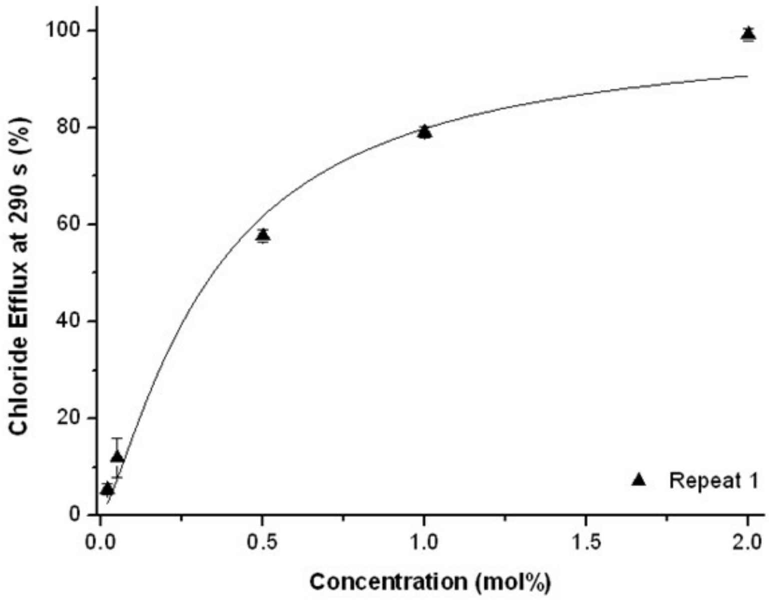

Repeat 1	Hill	
Equation	y=Vmax*x^n/(k^n+x^n)	
Reduced Chi-Sqr	39.09966	
Adj. R-Square	0.9769	
	Value	Standard Error
Vmax	100	0
EC_{50}	0.34586	0.06591
n	1.29565	0.26545

Figure S141. Overview of the Hill plots for compound **20**. For experimental details, see main text.

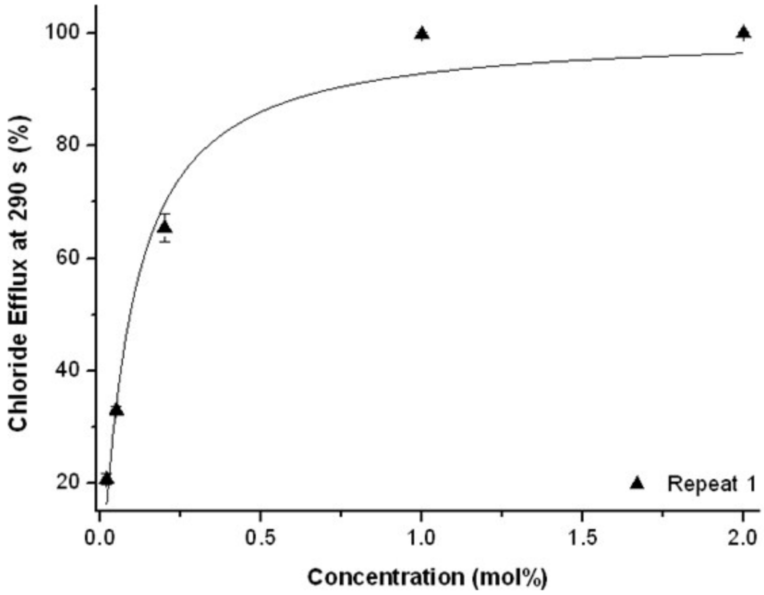

Repeat 1	Hill	
Equation	y=Vmax*x^n/(k^n+x^n)	
Reduced Chi-Sqr	33.63459	
Adj. R-Square	0.97519	
	Value	Standard Error
Vmax	100	0
EC_{50}	0.09208	0.01444
n	1.07582	0.16493

Figure S142. Overview of the Hill plots for compound **21**. For experimental details, see main text.

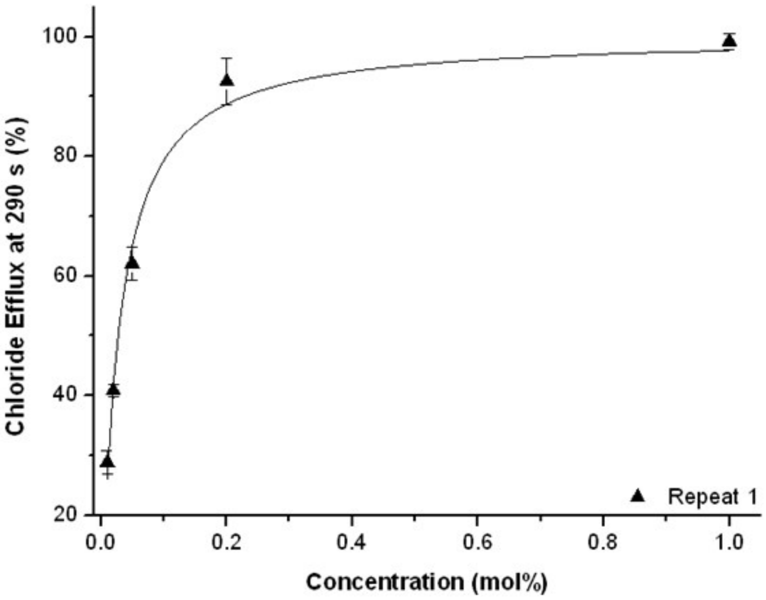

Repeat 1	Hill	
Equation	y=Vmax*x^n/(k^n+x^n)	
Reduced Chi-Sqr	11.70507	
Adj. R-Square	0.98776	
	Value	Standard Error
Vmax	100	0
EC_{50}	0.02736	0.00227
n	1.03739	0.10393

Figure S143. Overview of the Hill plots for compound **22**. For experimental details, see main text.

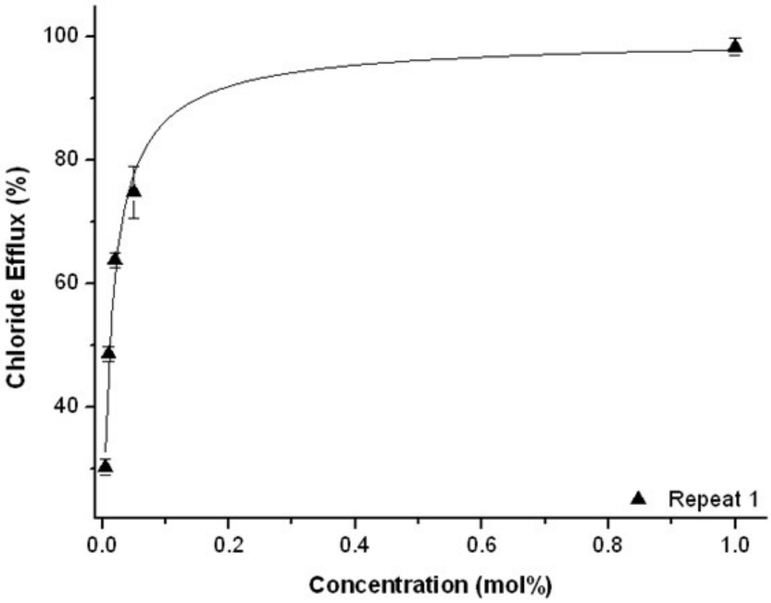

Repeat 1	Hill	
Equation	y=Vmax*x^n/(k^n+x^n)	
Reduced Chi-Sqr	7.8153	
Adj. R-Square	0.98827	
	Value	Standard Error
Vmax	100	0
EC_{50}	0.0116	8.65088E-4
n	0.85699	0.08023

Figure S144. Overview of the Hill plots for compound **23**. For experimental details, see main text.

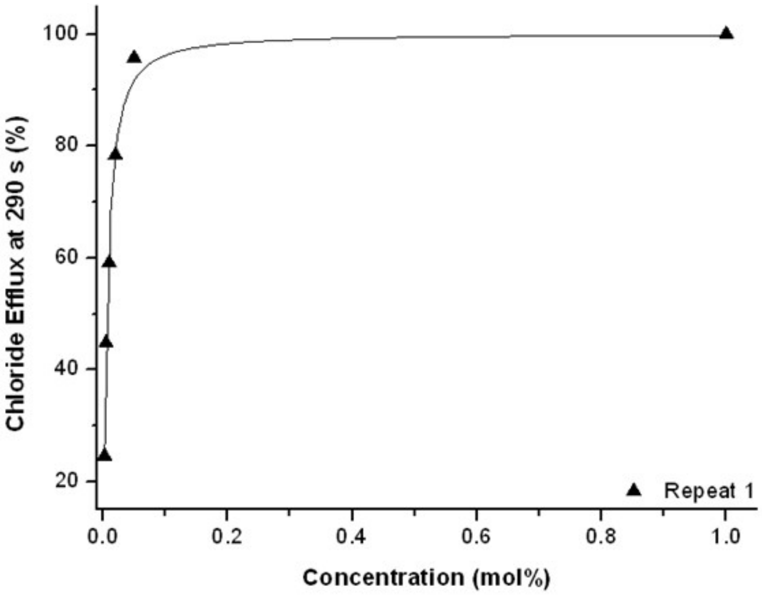

Repeat 1	Hill	
Equation	y=Vmax*x^n/(k^n+x^n)	
Reduced Chi-Sqr	8.43833	
Adj. R-Square	0.9904	
	Value	Standard Error
V_{max}	100	0
EC_{50}	0.00648	3.77774E-4
n	1.17753	0.09063

Figure S145. Overview of the Hill plots for compound **24**. For experimental details, see main text.

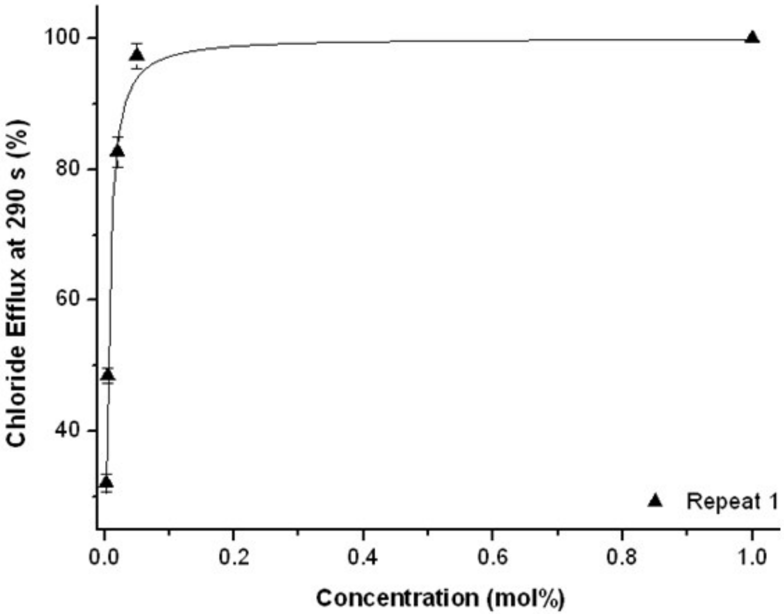

Repeat 1	Hill	
Equation	y=Vmax*x^n/(k^n+x^n)	
Reduced Chi-Sqr	5.941	
Adj. R-Square	0.99355	
	Value	Standard Error
Vmax	100	0
EC_{50}	0.005	2.85426E-4
n	1.18867	0.08951

Figure S146. Overview of the Hill plots for compound **25**. For experimental details, see main text.

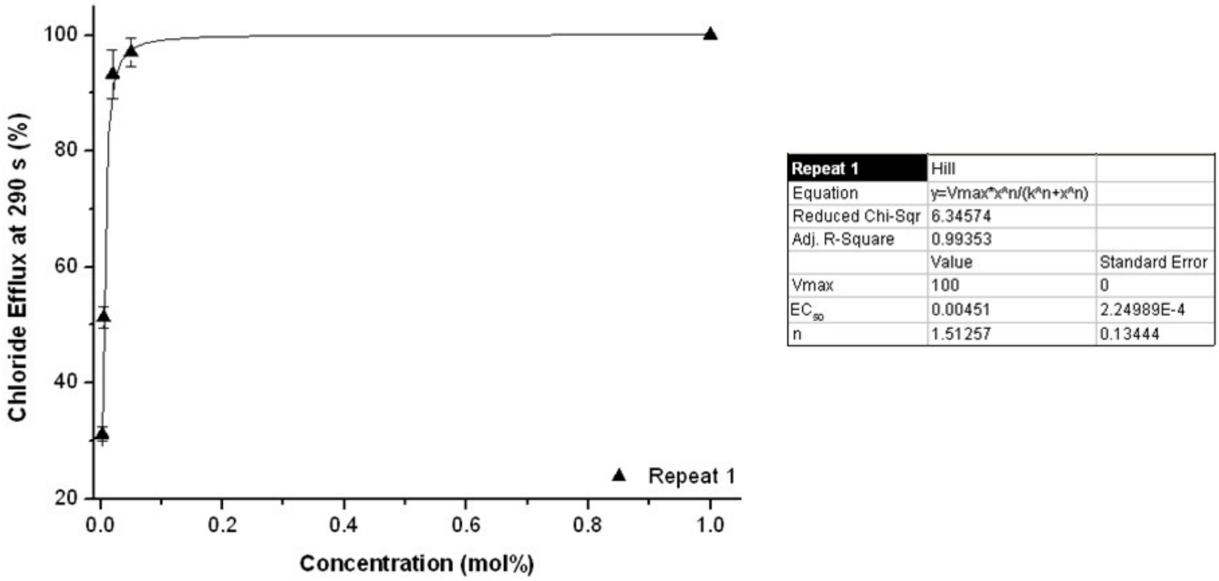

Figure S147. Overview of the Hill plots for compound **26**. For experimental details, see main text.

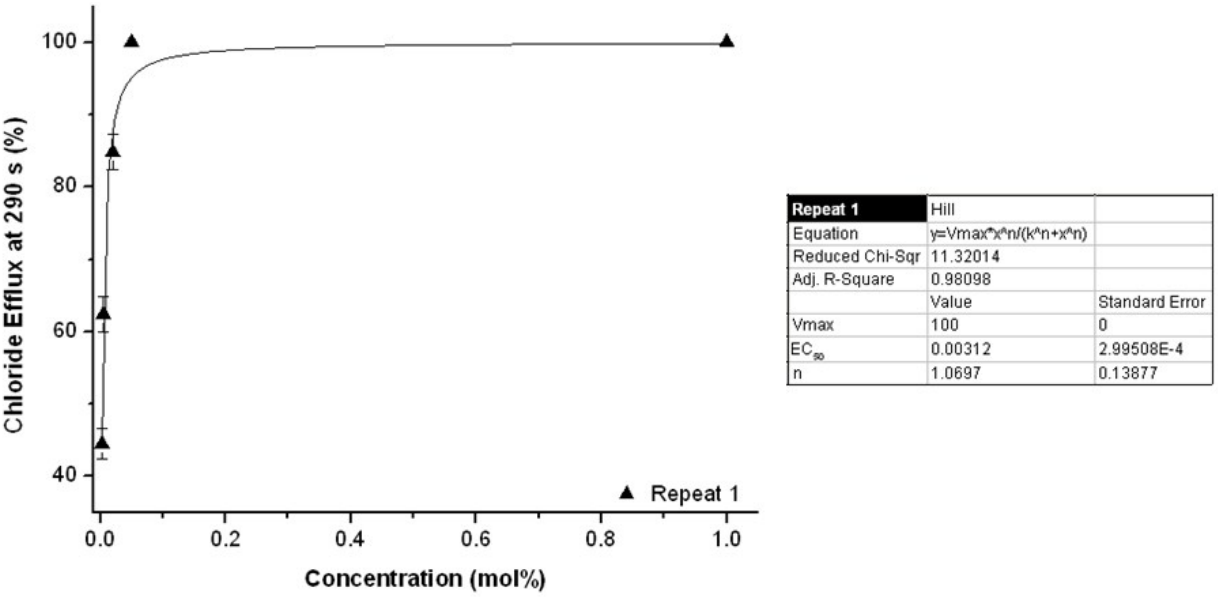

Figure S148. Overview of the Hill plots for compound **27**. For experimental details, see main text.

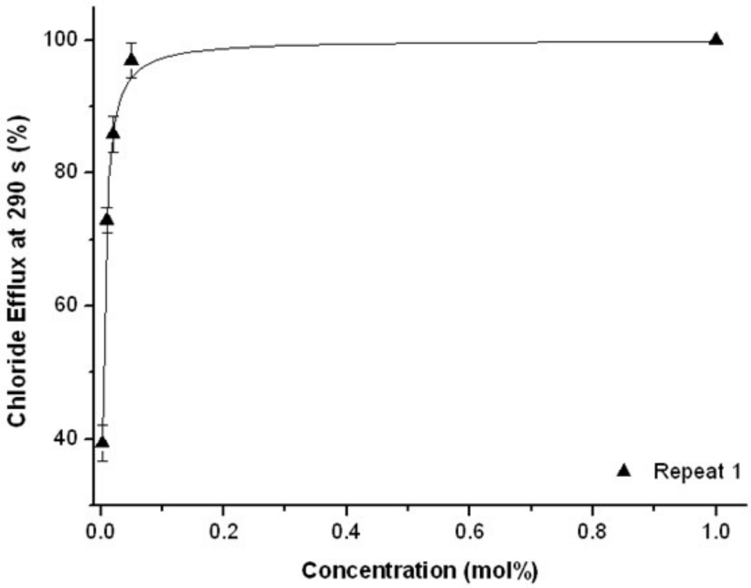

Repeat 1	Hill	
Equation	y=Vmax*x^n/(k^n+x^n)	
Reduced Chi-Sqr	2.96968	
Adj. R-Square	0.99509	
	Value	Standard Error
Vmax	100	0
EC_{50}	0.0038	1.96921E-4
n	1.09531	0.05883

Figure S149. Overview of the Hill plots for compound **28**. For experimental details, see main text.

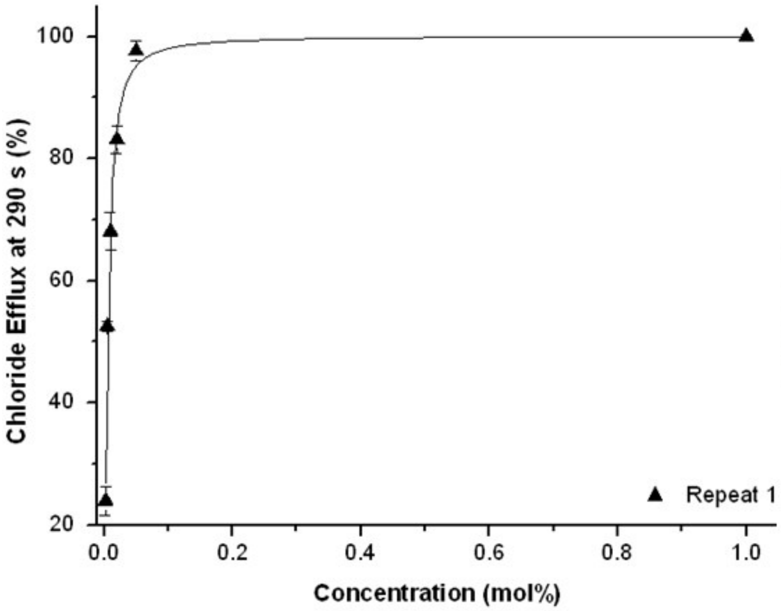

Repeat 1	Hill	
Equation	y=Vmax*x^n/(k^n+x^n)	
Reduced Chi-Sqr	10.82234	
Adj. R-Square	0.98727	
	Value	Standard Error
Vmax	100	0
EC_{50}	0.0053	3.3078E-4
n	1.32819	0.11979

Figure S150. Overview of the Hill plots for compound **29**. For experimental details, see main text.

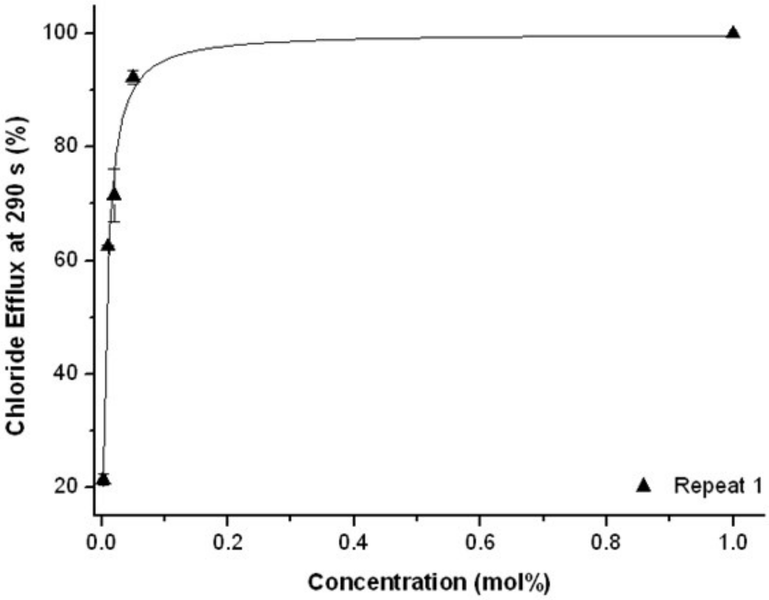

Repeat 1	Hill	
Equation	y=Vmax*x^n/(k^n+x^n)	
Reduced Chi-Sqr	13.34365	
Adj. R-Square	0.98605	
	Value	Standard Error
Vmax	100	0
EC_{50}	0.00731	6.72424E-4
n	1.15405	0.11892

Figure S151. Overview of the Hill plots for compound **30**. For experimental details, see main text.

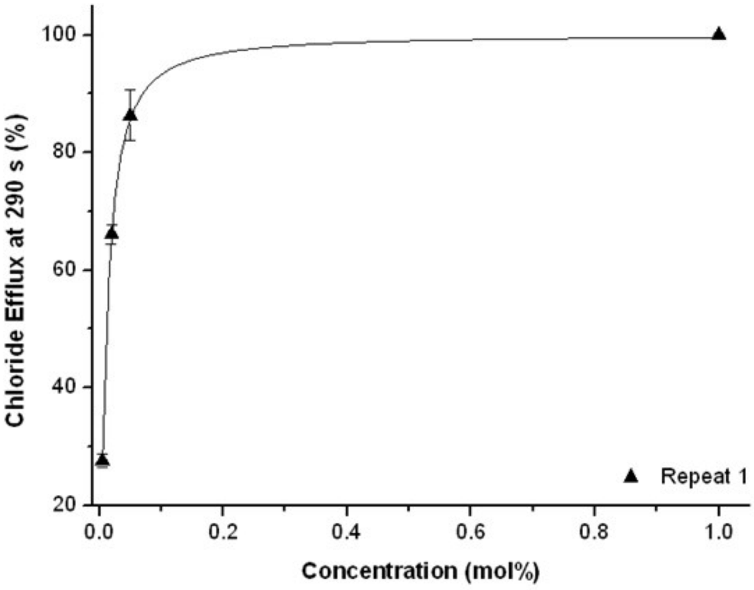

Repeat 1	Hill	
Equation	y=Vmax*x^n/(k^n+x^n)	
Reduced Chi-Sqr	0.41221	
Adj. R-Square	0.99958	
	Value	Standard Error
Vmax	100	0
EC_{50}	0.01125	1.9057E-4
n	1.20205	0.02381

Figure S152. Overview of the Hill plots for compound **31**. For experimental details, see main text.

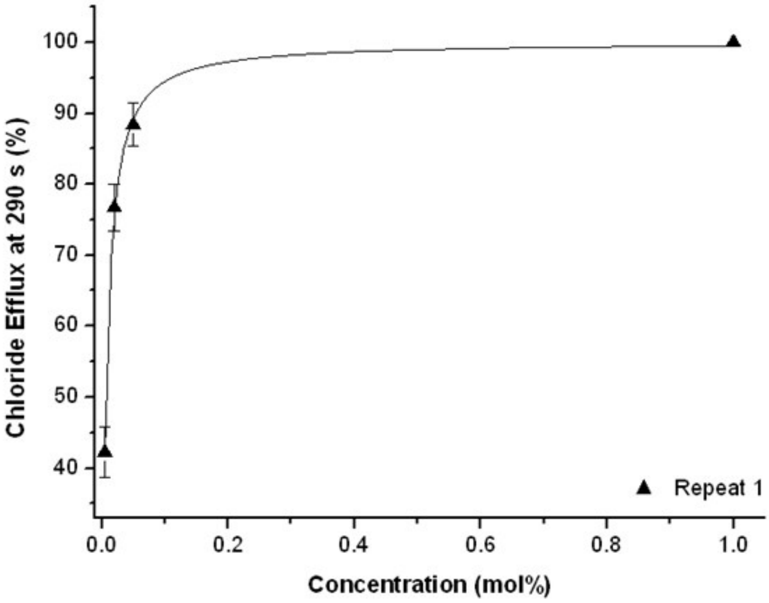

Repeat 1	Hill	
Equation	y=Vmax*x^n/(k^n+x^n)	
Reduced Chi-Sqr	0.7522	
Adj. R-Square	0.99879	
	Value	Standard Error
Vmax	100	0
EC_{50}	0.00668	1.89177E-4
n	1.05064	0.03302

Figure S153. Overview of the Hill plots for compound **32**. For experimental details, see main text.

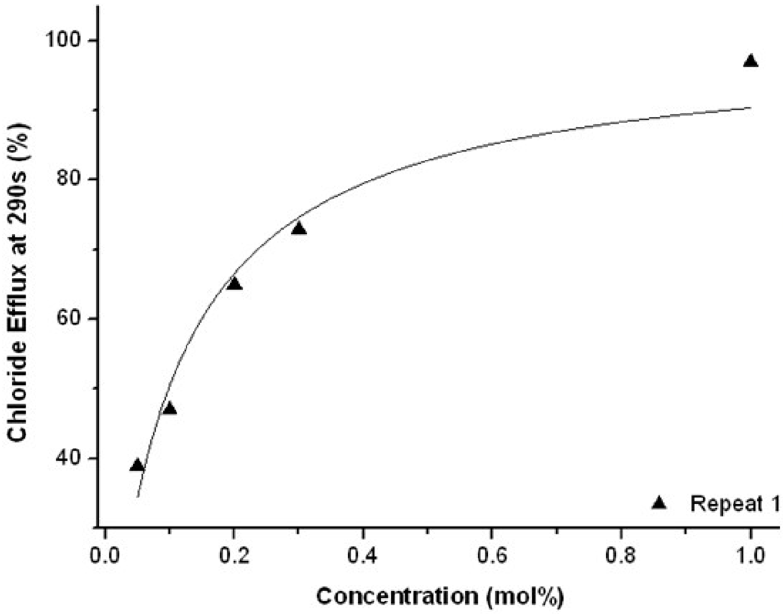

Repeat 1	Hill	
Equation	y=Vmax*x^n/(k^n+x^n)	
Reduced Chi-Sqr	27.22858	
Adj. R-Square	0.94774	
	Value	Standard Error
Vmax	100	0
EC_{50}	0.09767	0.01252
n	0.96266	0.15285

Figure S154. Overview of the Hill plots for compound **33**. For experimental details, see main text.

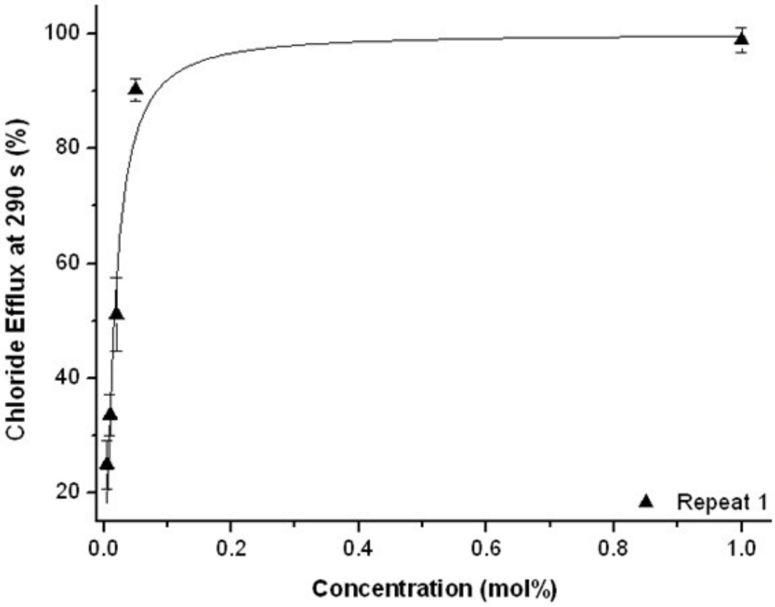

Repeat 1	Hill	
Equation	y=Vmax*x^n/(k^n+x^n)	
Reduced Chi-Sqr	54.44441	
Adj. R-Square	0.95098	
	Value	Standard Error
Vmax	100	0
EC_{50}	0.01565	0.00223
n	1.32058	0.27634

Figure S155. Overview of the Hill plots for compound **34**. For experimental details, see main text.

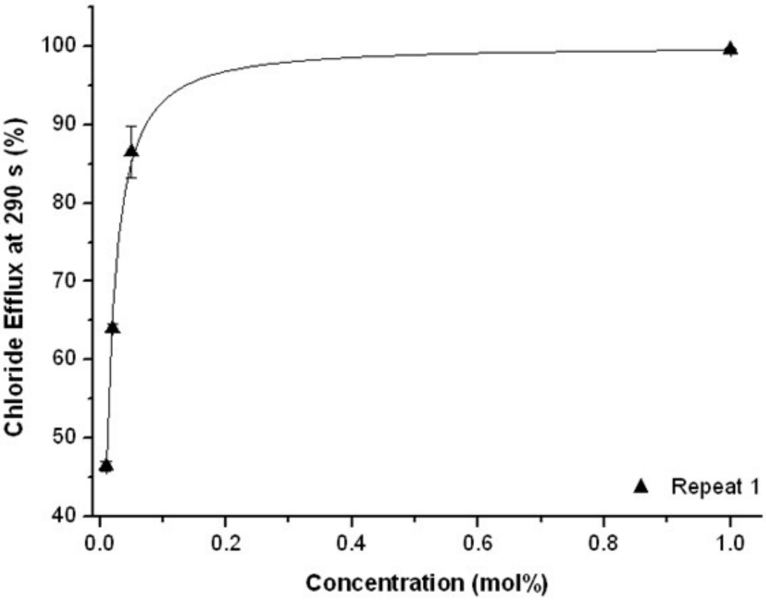

Repeat 1	Hill	
Equation	y=Vmax*x^n/(k^n+x^n)	
Reduced Chi-Sqr	2.81645	
Adj. R-Square	0.99496	
	Value	Standard Error
Vmax	100	0
EC_{50}	0.01162	5.30281E-4
n	1.19969	0.08711

Figure S156. Overview of the Hill plots for compound **35**. For experimental details, see main text.

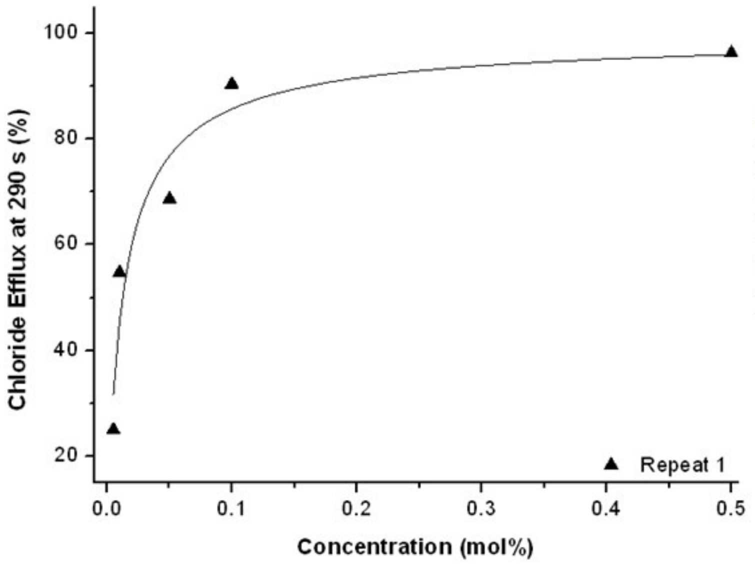

Repeat 1	Hill	
Equation	y=Vmax*x^n/(k^n+x^n)	
Reduced Chi-Sqr	72.56585	
Adj. R-Square	0.9127	
	Value	Standard Error
Vmax	100	0
EC_{50}	0.01234	0.00314
n	0.85732	0.19845

Figure S157. Overview of the Hill plots for compound **36**. For experimental details, see main text.

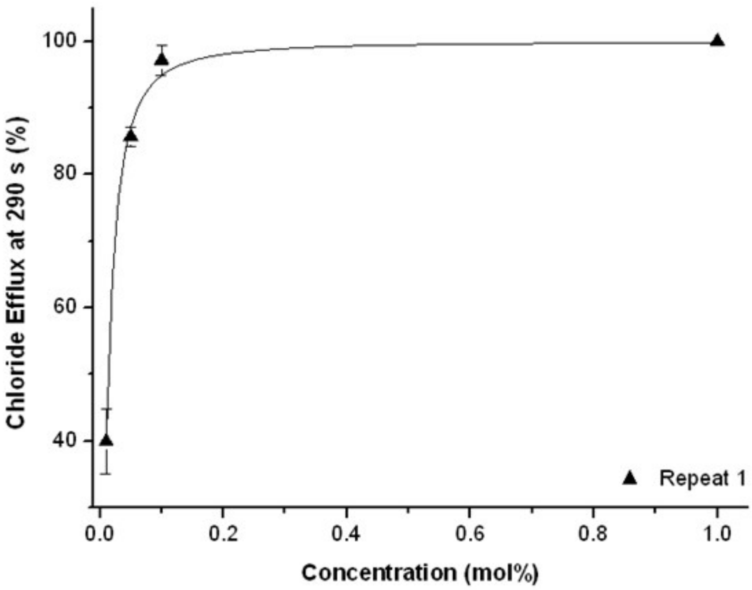

Repeat 1	Hill	
Equation	y=Vmax*x^n/(k^n+x^n)	
Reduced Chi-Sqr	3.68851	
Adj. R-Square	0.99526	
	Value	Standard Error
Vmax	100	0
EC_{50}	0.01334	6.60831E-4
n	1.44971	0.1013

Figure S158. Overview of the Hill plots for compound **37**. For experimental details, see main text.

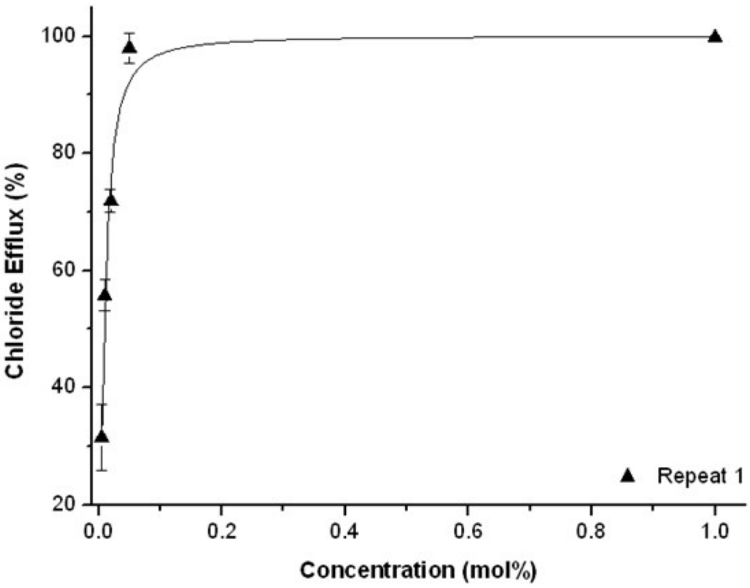

Repeat 1	Hill	
Equation	y=Vmax*x^n/(k^n+x^n)	
Reduced Chi-Sqr	18.09083	
Adj. R-Square	0.97836	
	Value	Standard Error
Vmax	100	0
EC_{50}	0.00878	7.03168E-4
n	1.42571	0.18881

Figure S159. Overview of the Hill plots for compound **38**. For experimental details, see main text.

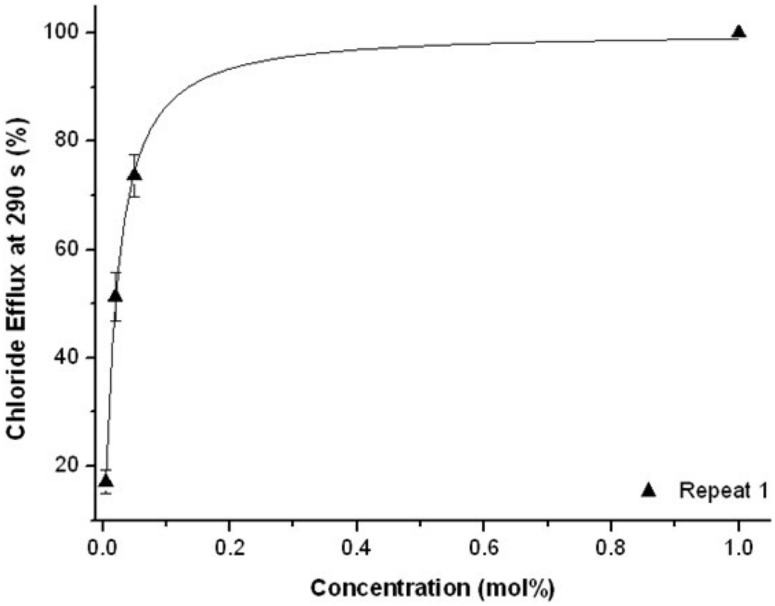

Repeat 1	Hill	
Equation	y=Vmax*x^n/(k^n+x^n)	
Reduced Chi-Sqr	1.14712	
Adj. R-Square	0.99907	
	Value	Standard Error
Vmax	100	0
EC_{50}	0.01957	5.34622E-4
n	1.13632	0.03984

Figure S160. Overview of the Hill plots for compound **39**. For experimental details, see main text.

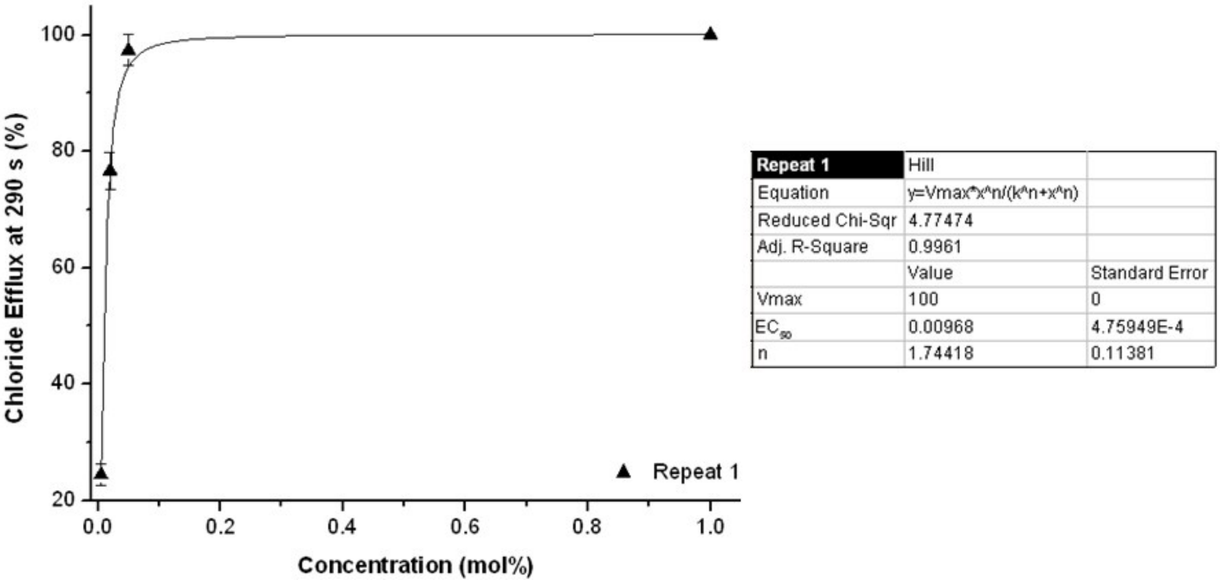

Repeat 1	Hill	
Equation	y=Vmax*x^n/(k^n+x^n)	
Reduced Chi-Sqr	4.77474	
Adj. R-Square	0.9961	
	Value	Standard Error
Vmax	100	0
EC_{50}	0.00968	4.75949E-4
n	1.74418	0.11381

Figure S161. Overview of the Hill plots for compound **40**. For experimental details, see main text.

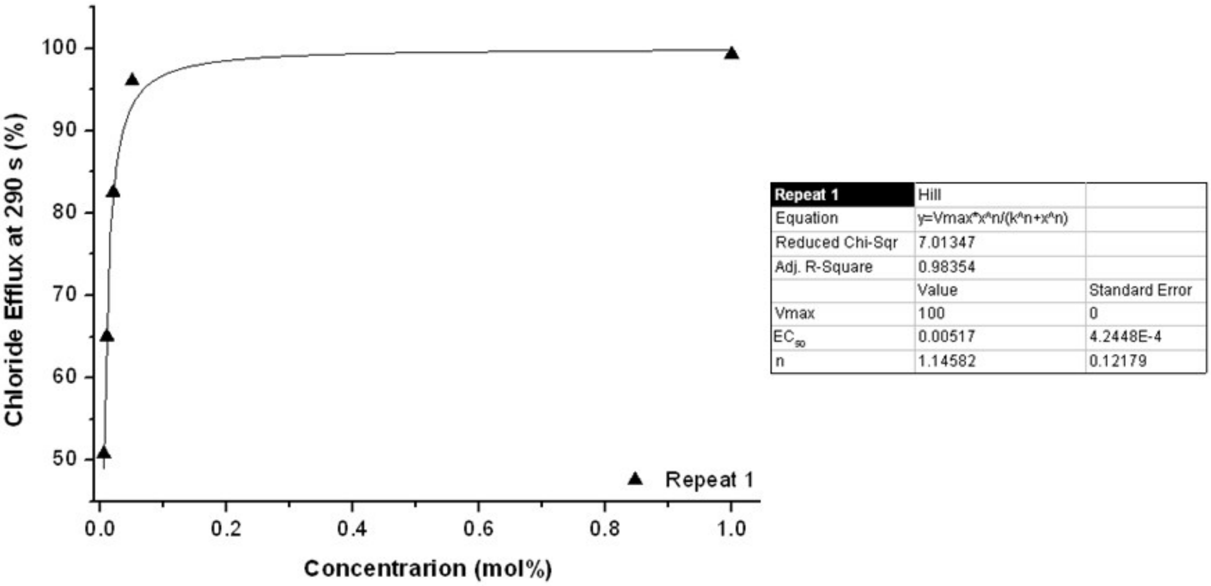

Repeat 1	Hill	
Equation	y=Vmax*x^n/(k^n+x^n)	
Reduced Chi-Sqr	7.01347	
Adj. R-Square	0.98354	
	Value	Standard Error
Vmax	100	0
EC_{50}	0.00517	4.2448E-4
n	1.14582	0.12179

Figure S162. Overview of the Hill plots for compound **41**. For experimental details, see main text.

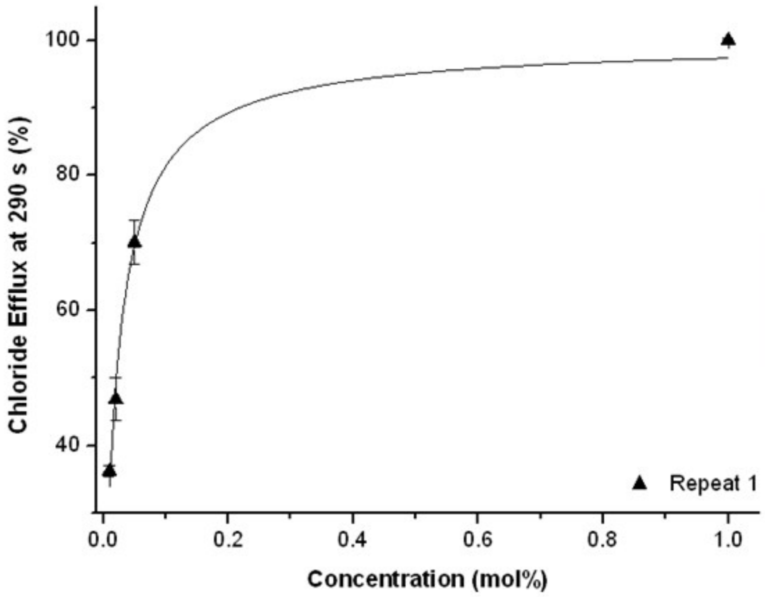

Repeat 1	Hill	
Equation	y=Vmax*x^n/(k^n+x^n)	
Reduced Chi-Sqr	9.59058	
Adj. R-Square	0.988	
	Value	Standard Error
Vmax	100	0
EC_{50}	0.02044	0.00172
n	0.92909	0.11606

Figure S163. Overview of the Hill plots for compound **42**. For experimental details, see main text.

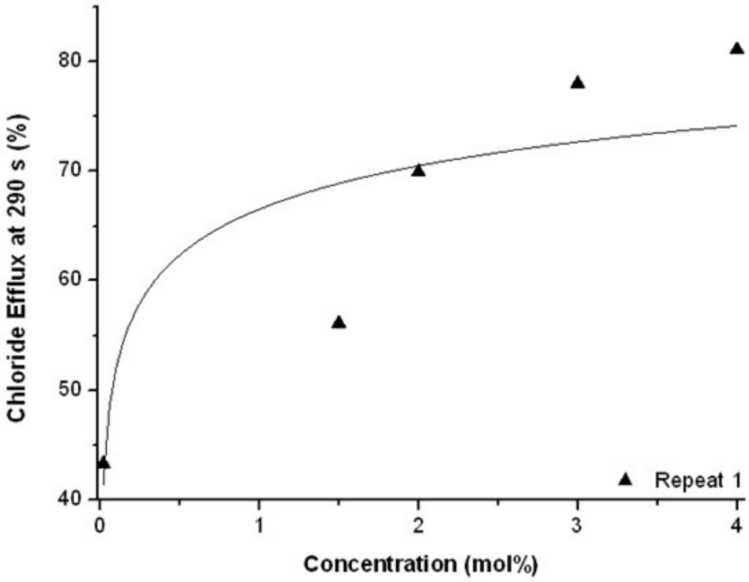

Repeat 1	Hill	
Equation	y=Vmax*x^n/(k^n+x^n)	
Reduced Chi-Sqr	81.29946	
Adj. R-Square	0.67502	
	Value	Standard Error
Vmax	100	0
EC_{50}	0.07442	0.07719
n	0.26473	0.08959

Figure S164. Overview of the Hill plots for compound **43**. For experimental details, see main text.

2.5. K_{ini} EC_{50} correlation for EC_{50} Prediction

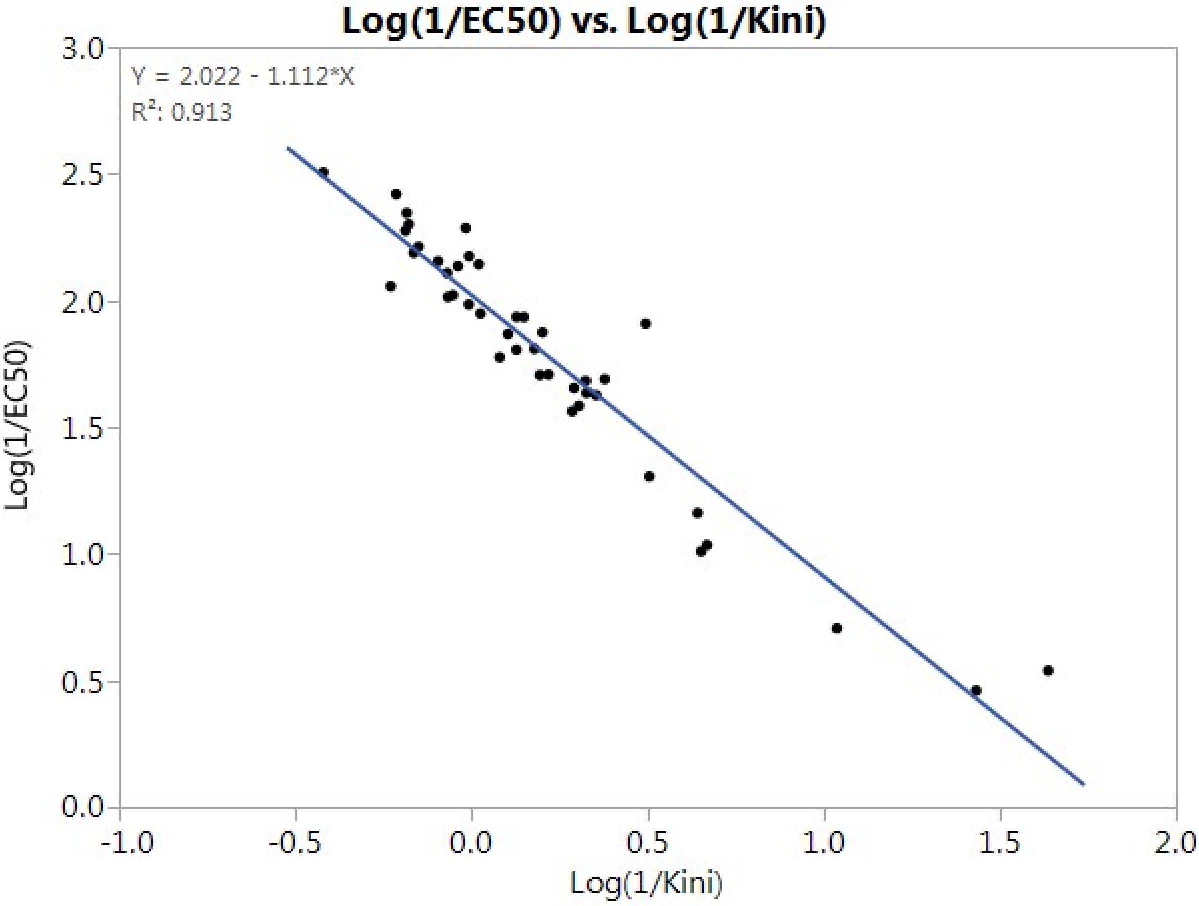

Figure S165 –Correlation Plot for Log(1/k_{ini}) and Log(1/EC_{50}) excluding compound **43**. Used in the prediction of EC_{50} for compound **43**

2.6. Overview of anion transport results

Compound	EC_{50} (mol%)	Error (EC_{50})	n	Error (n)	k_{ini} (% s^{-1})	Error (k_{ini})
1	0.0072	0.0015	1.19	0.16	0.95	0.11
2	0.0061	0.0018	1.23	0.20	1.41	0.46
3	0.0070	0.0020	1.25	0.12	1.24	0.27
4	0.0078	0.0011	1.19	0.17	1.17	0.15
5	0.0104	0.0022	1.30	0.01	1.02	0.25
6	0.0095	0.0017	1.25	0.10	1.13	0.16
7	0.2884	0.0402	0.96	0.13	0.02	0.01
8	0.0688	0.0204	1.42	0.04	0.23	0.07
9	0.0197	0.0049	1.29	0.15	0.64	0.09
10	0.0136	0.0017	1.28	0.09	0.79	0.15
11	0.0231	0.0014	1.31	0.06	0.47	0.11
12	0.0262	0.0075	1.29	0.16	0.49	0.15
13	0.0208	0.0018	1.18	0.36	0.47	0.09
14	0.0155	0.0025	1.27	0.12	0.66	0.22
15	0.0236	0.0018	1.37	0.12	0.44	0.05
16	0.0221	0.0025	1.29	0.08	0.51	0.04
17	0.0167	0.0023	1.48	0.10	0.83	0.16
18	0.0494	0.0035	1.59	0.20	0.31	0.02
19	0.1965	0.0144	0.85	0.05	0.09	0.01
20	0.3459	0.0659	1.30	0.27	0.04	0.01
21	0.0921	0.0144	1.08	0.16	0.21	0.01
22	0.0274	0.0023	1.04	0.10	0.52	0.02
23	0.0116	0.0009	0.86	0.08	0.74	0.05
24	0.0065	0.0004	1.18	0.09	1.46	0.09
25	0.0050	0.0003	1.19	0.09	1.50	0.17
26	0.0045	0.0002	1.51	0.13	1.52	0.12
27	0.0031	0.0003	1.07	0.14	2.63	0.11
28	0.0038	0.0002	1.10	0.16	1.63	0.25
29	0.0053	0.0003	1.33	0.12	1.54	0.27
30	0.0073	0.0007	1.15	0.12	1.09	0.12
31	0.0113	0.0002	1.20	0.02	0.94	0.10
32	0.0067	0.0002	1.05	0.03	1.01	0.06
33	0.0977	0.0125	0.96	0.15	0.22	0.04
34	0.0157	0.0022	1.32	0.28	0.74	0.01
35	0.0116	0.0005	1.20	0.09	0.71	0.03
36	0.0123	0.0031	0.86	0.20	0.32	0.08
37	0.0133	0.0007	1.45	0.10	0.60	0.03
38	0.0088	0.0007	1.43	0.19	1.69	0.09
39	0.0196	0.0005	1.14	0.04	0.60	0.04
40	0.0097	0.0005	1.74	0.11	1.16	0.11
41	0.0052	0.0004	1.15	0.12	1.04	0.01
42	0.0204	0.0017	0.93	0.12	0.42	0.03
43	0.0616[a]	0.0241	-[a]	-	0.19	0.03

Table S1. Overview of average of the obtained EC_{50}, n and k_{ini} values. [a] Large error in Hill plot due to solubility issues. EC_{50} calculated from correlation with k_{ini} (see Figure S165).

3. QSAR ANALYSIS

3.1. Fit all Models – 3 and 4 parameters

No. Des.	Descriptors				R^2
3	ALOGPs	ALOGPs-sq	Mv	–	0.7901
3	ALOGPs	ALOGPs-sq	J3D	–	0.7892
3	ALOGPs	ALOGPs-sq	Mp	–	0.7836
3	ALOGPs	ALOGPs-sq	nH	–	0.7822
3	ALOGPs	ALOGPs-sq	AMW	–	0.7768
3	ALOGPs	ALOGPs-sq	J	–	0.7680
3	ALOGPs	ALOGPs-sq	E3u	–	0.7672
3	ALOGPs	ALOGPs-sq	ARR	–	0.7654
3	ALOGPs	ALOGPs-sq	Density (g/cm^3)	–	0.7615
3	ALOGPs	ALOGPs-sq	Surface tension (dyne/cm)	–	0.7571
3	ALOGPs	ALOGPs-sq	Lop	–	0.7560
3	ALOGPs	ALOGPs-sq	Du	–	0.7558
3	ALOGPs	ALOGPs-sq	E3e	–	0.7554
3	ALOGPs	ALOGPs-sq	Ui	–	0.7533
3	ALOGPs	ALOGPs-sq	LogD duodenum (pH 4.6)	–	0.7516
3	ALOGPs	ALOGPs-sq	LogD Jejunum & Ileum (pH 6.5)	–	0.7513
3	ALOGPs	ALOGPs-sq	LogD (pH 7.2) blood	–	0.7503
3	ALOGPs	ALOGPs-sq	LogD (pH 7.4) blood	–	0.7497
3	ALOGPs	ALOGPs-sq	LogD (pH 8) colon	–	0.7458
3	ALOGPs	ALOGPs-sq	Me	–	0.7394
4	ALOGPs	ALOGPs-sq	nClC	J3D	0.8160
4	ALOGPs	ALOGPs-sq	nH	J	0.8152
4	ALOGPs	ALOGPs-sq	AMW	J	0.8151
4	ALOGPs	ALOGPs-sq	AMW	J3D	0.8141
4	ALOGPs	ALOGPs-sq	J3D	Ui	0.8140
4	ALOGPs	ALOGPs-sq	Density (g/cm3)	J3D	0.8138
4	ALOGPs	ALOGPs-sq	Density (g/cm3)	J	0.8121
4	ALOGPs	ALOGPs-sq	Parachor (cm3)	nH	0.8099
4	ALOGPs	ALOGPs-sq	Molar refractivity (cm3)	nH	0.8085
4	ALOGPs	ALOGPs-sq	Polarizability (cm3)	nH	0.8084
4	ALOGPs	ALOGPs-sq	Molecular Volume (cm3)	nH	0.8084
4	ALOGPs	ALOGPs-sq	Me	Mp	0.8080
4	ALOGPs	ALOGPs-sq	LogD (pH 6.5)	AMW	0.8069
4	ALOGPs	ALOGPs-sq	LogD (pH 7.2)	AMW	0.8068
4	ALOGPs	ALOGPs-sq	LogD (pH 7.4)	AMW	0.8068
4	ALOGPs	ALOGPs-sq	LogD (pH 8)	nH	0.8063
4	ALOGPs	ALOGPs-sq	LogD (pH 8)	AMW	0.8062
4	ALOGPs	ALOGPs-sq	LogD (pH 4.6)	AMW	0.8062
4	ALOGPs	ALOGPs-sq	LogD (pH 7.4)	nH	0.8056
4	ALOGPs	ALOGPs-sq	LogD (pH 7.2)	nH	0.8054

Table S2 - 20 Best fitted 3 and 4 parameter models, ranked by R^2 values.
4 parameter models are fitted with a subset of descriptors.

3.2. Descriptor Definitions

ALOGPs – octanol/water partition coefficient

ALOGPs-sq – octanol/water partition coefficient squared

AMW - average molecular weight

ARR – aromatic ratio

De - D total accessibility index / weighted by Sanderson electronegativity

Density (g/cm3)

Du - D total accessibility index / unweighted

E3e - 3rd component accessibility directional WHIM index / weighted by Sanderson electronegativity

E3u - 3rd component accessibility directional WHIM index / unweighted

J - Balaban distance connectivity index

J3D - 3D-Balaban index

LogD (pH 7.2) – distribution constant, calculated at pH 7.2

LogD (pH 7.4) blood – distribution constant, calculated at pH 7.4

LogD (pH 8) colon – distribution constant, calculated at pH 8

LogD duodenum (pH 4.6) – distribution constant, calculated at pH 4.6

LogD Jejunum & Ileum (pH 6.5) – distribution constant, calculated at pH 6.5

Lop - Lopping centric index

Molecular Volume (cm3)

Mp - mean atomic polarizability (scaled on Carbon atom)

Mv - mean atomic van der Waals volume (scaled on Carbon atom)

nCIC - number of rings (cyclomatic number)

nH - number of Hydrogen atoms

Parachor (cm3)

Polarizability (cm3)

Surface Tension (dyne/cm) - surface free energy

Ui - unsaturation index

3.3. Model Fits

Coefficients

			Linear Fit		Bootstrap			Linear Fit		Bootstrap			Linear Fit		Bootstrap			Linear Fit		Bootstrap			Linear Fit		Bootstrap	
**Model parameters**	**R^2 value**	**Intercept**	**2.5% CI**	**97.5% CI**	**2.5% CI**	**97.5% CI**	**ALogPs**	**2.5% CI**	**97.5% CI**	**2.5% CI**	**97.5% CI**	**ALogPs ^2**	**2.5% CI**	**97.5% CI**	**2.5% CI**	**97.5% CI**	**3rd Param**	**2.5% CI**	**97.5% CI**	**2.5% CI**	**97.5% CI**	**4th param**	**2.5% CI**	**97.5% CI**	**2.5% CI**	**97.5% CI**
ALogPs, ALogPs^2 **(1)**	0.6292	-0.579	-1.165	0.008	-1.108	-0.086	1.203	0.903	1.504	0.904	1.470	-0.133	-0.168	-0.098	-0.166	-0.093										
ALogPs, ALogPs^2, nH **(2)**	0.782	-1.659	-2.276	-1.041	-2.454	-1.033	1.310	1.073	1.547	1.038	1.601	-0.176	-0.207	-0.144	-0.213	-0.130	0.065	0.040	0.090	0.043	0.087					
ALogPs, ALogPs^2, Mv **(3)**	0.7901	3.362	1.838	4.887	2.159	4.419	1.372	1.135	1.61	1.126	1.579	-0.158	-0.186	-0.129	-0.19	-0.123	-6.616	-9.063	-4.168	-8.432	-4.473					
ALogPs, ALogPs^2, J3D **(4)**	0.789	-2.372	-3.176	-1.569	-3.071	-1.648	1.339	1.104	1.574	1.109	1.556	-0.148	-0.175	-0.120	-0.177	-0.115	0.733	0.461	1.006	0.495	0.960					
ALogPs, ALogPs^2, ARR **(5)**	0.765	-0.222	-0.719	0.274	-0.647	0.070	1.366	1.114	1.618	1.117	1.605	-0.153	-0.182	-0.123	-0.186	-0.116	-1.675	-2.387	-0.963	-2.290	-1.065					
AlogPs, ALogPs^2, AMW **(6)**	0.777	0.731	0.035	1.427	-0.113	1.368	1.330	1.089	1.572	1.104	1.536	-0.154	-0.183	-0.125	-0.182	-0.121	-0.200	-0.279	-0.120	-0.293	-0.133					
ALogPs, ALogPs^2, nCIC, J3D (**7**)	0.816	-5.105	-7.579	-2.632	-7.681	-2.694	1.284	1.056	1.511	1.087	1.493	-0.146	-0.172	-0.120	-0.173	-0.116	0.411	0.057	0.764	0.064	0.796	1.587	0.808	2.367	0.796	2.330
ALogPs, ALogP^2, AMW, J3D (**8**)	0.814	-1.018	-2.453	0.417	-2.309	0.429	1.361	1.137	1.586	1.123	1.591	-0.154	-0.181	-0.127	-0.185	-0.119	-0.110	-0.209	-0.011	-0.218	-0.051	0.475	0.127	0.823	0.148	0.781
ALogPs, ALogPs^2, nH, J (**9**)	0.815	-3.067	-4.304	-1.830	-4.133	-1.767	1.432	1.191	1.673	1.196	1.653	-0.172	-0.202	-0.142	-0.205	-0.128	0.044	0.015	0.728	0.014	0.071	0.904	0.202	1.607	0.173	1.475
ALogPs, ALogPs^2, Polarizability, nH (**10**)	0.808	-0.983	-1.822	-0.144	-1.736	0.078	1.307	1.082	1.532	1.032	1.519	-0.165	-0.197	-0.132	-0.199	-0.110	-0.022	-0.041	-0.002	-0.036	0.005	0.063	0.039	0.087	0.040	0.087

Table S3. Coefficients & Confidence intervals for Linear and Bootstrap fits for 2, 3 and 4 parameter models.

3.4. Model fit plots

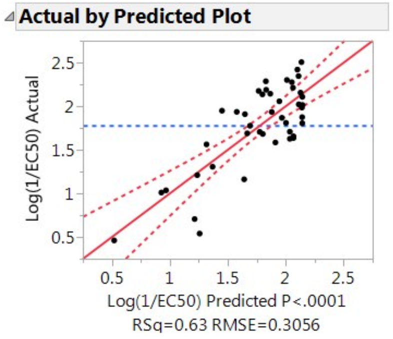

1) ALOGPs, ALogPs-sq

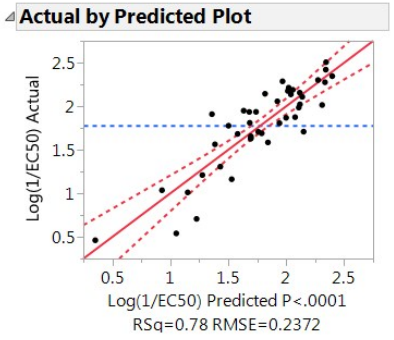

2) ALogPs, ALogPs-sq, NH

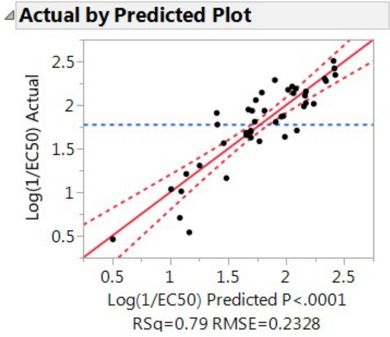

3) ALogPs, AlogPs-sq, Mv

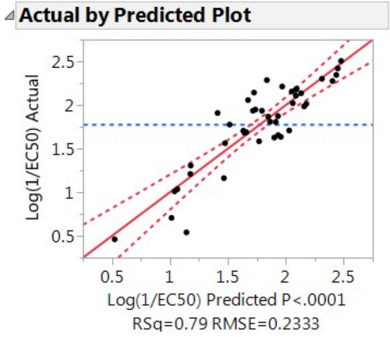

4) ALogPs, ALogPs-sq, J3D

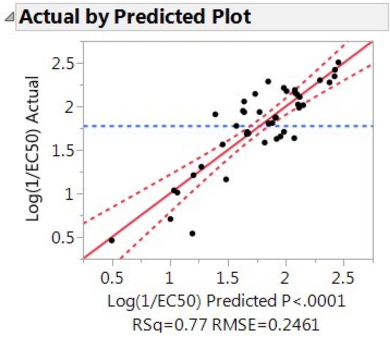

5) ALogPs, AlogPs-sq, ARR

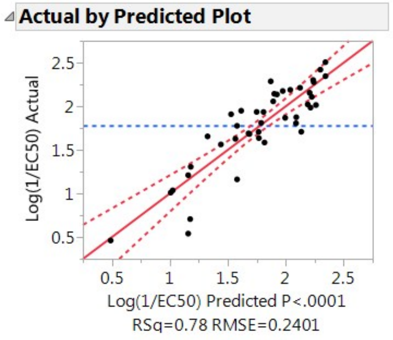

6) ALogPs, AlogPs-sq, AMW

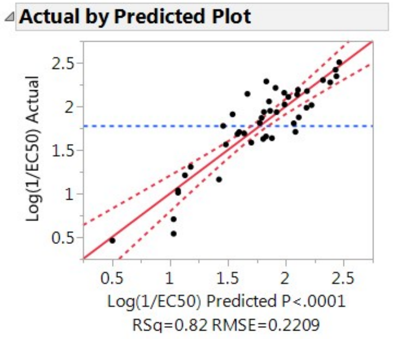

7) ALOGPs, ALogPs-sq, nCIC, J3D

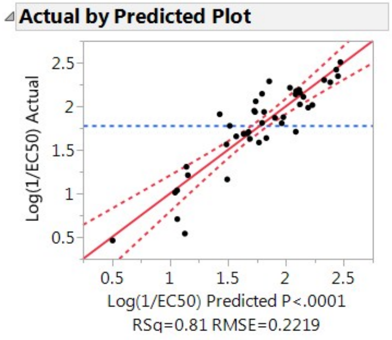

8) ALOGPs, ALogPs-sq, AMW, J3D

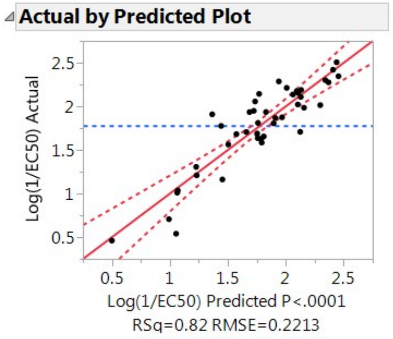

9) ALOGPs, ALogPs-sq, nH, J

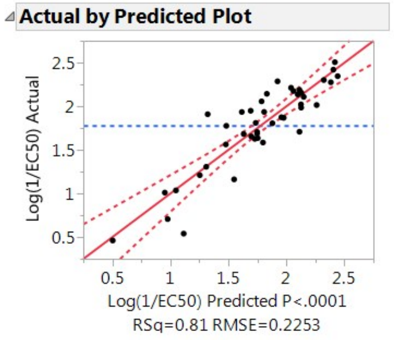

10) ALOGPs, ALogPs-sq, Polarizability nH

Figure S166. Plots showing the predicted vs actual values for Log(1/EC_{50}) for 10 of the models fitted

3.5. R Code from linear fits

R Code and Results from linear fits of 2, 3 and 4 parameter models

```
library(boot)

dataset<-read.csv("Tambjamines_New_numbers_classified_cleaned.csv")
ds.sb<-dataset[,c('ï..Compound.no','Ring.substituent',
'NH.substituent','Enamine.Substituent','R.type','Log.1.EC50.','ALOGPs','ALOGPs.sq','nH','Mv','J3D','AMW','J','ARR',
'nCIC','Polarizability..cm3.')]


plot(ds.sb$ALOGPs, ds.sb$Log.1.EC50., main="Simple plot of Log(1/EC50) against ALOGPs", xlab="ALOGPs",
ylab="Log(1/EC50)")
#plots ALOGPs against log(1/ec50)

# define stats function to give to boot strap, at this stage just the quadratic fit
bs <- function(formula, data, indices) {
d <- data[indices,] # allows boot to select sample
fit <- lm(formula, data=d)
return(coef(fit))
}

# define simpler data frame and give x and y variables
# gives dataframe with only 2 columns (the necessary columns)
df1 <-data.frame(x=ds.sb$ALOGPs,y=ds.sb$Log.1.EC50.)

# define the strata
# limits of ALOGPs are below 1.5 and above 6 in this set
strata <- ifelse(ds.sb$ALOGPs > 1.5, 1,0)+ ifelse(ds.sb$ALOGPs > 6, 1,0)
```

#ALOGPs, ALOGPS-sq

```
# do the bootstrap
res_plainfit <-boot(data = df1, statistic = bs, strata = strata, R=999, formula = y ~ x + I(x^2))

# now evaluate the confidence limit from the boot strap, index = 1 is the intercept, 2 the
# coefficient of ALOGPs and 3 the coefficient of ALOGPs^2
# bca - Adjusted Bootstrap percentile interval


> boot.ci(res_plainfit, type="bca",index=1)
BOOTSTRAP CONFIDENCE INTERVAL CALCULATIONS
Based on 999 bootstrap replicates
CALL :
boot.ci(boot.out = res_plainfit, type = "bca", index = 1)

Intervals :
Level      BCa
95%   (-1.1079, -0.0859 )
Calculations and Intervals on Original Scale

> boot.ci(res_plainfit, type="bca",index=2)
BOOTSTRAP CONFIDENCE INTERVAL CALCULATIONS
Based on 999 bootstrap replicates
CALL :
boot.ci(boot.out = res_plainfit, type = "bca", index = 2)

Intervals :
Level      BCa
```

```
95%   ( 0.904,  1.470 )
Calculations and Intervals on Original Scale

> boot.ci(res_plainfit, type="bca",index=3)
BOOTSTRAP CONFIDENCE INTERVAL CALCULATIONS
Based on 999 bootstrap replicates
CALL :
boot.ci(boot.out = res_plainfit, type = "bca", index = 3)

Intervals :
Level     BCa
95%   (-0.1658, -0.0926 )
Calculations and Intervals on Original Scale

# do the usual linear least squares for the quadratic
# lm is linear model using df1 as the data source
fitplain <- lm(y ~ x + I(x^2), data = df1)

# look at the coefficients and confidence limit from the least squares

> summary(fitplain)

Call:
lm(formula = y ~ x + I(x^2), data = df1)

Residuals:
     Min       1Q   Median       3Q      Max
-0.71723 -0.17356  0.02163  0.25389  0.49909

Coefficients:
            Estimate Std. Error t value Pr(>|t|)
(Intercept) -0.57873    0.29008  -1.995   0.0529 .
x            1.20333    0.14861   8.097 5.83e-10 ***
I(x^2)      -0.13293    0.01746  -7.613 2.67e-09 ***
---
Signif. codes:  0 ‘***’ 0.001 ‘**’ 0.01 ‘*’ 0.05 ‘.’ 0.1 ‘ ’ 1

Residual standard error: 0.3056 on 40 degrees of freedom
Multiple R-squared:  0.6292,	Adjusted R-squared:  0.6106
F-statistic: 33.93 on 2 and 40 DF,  p-value: 2.42e-09confint(fitplain)
2.5 % 97.5 %
(Intercept) -1.1829892 -0.008978968
x 0.9142820 1.515710944
I(x^2) -0.1699811 -0.099315781
>
> confint(fitplain)
                 2.5 %      97.5 %
(Intercept) -1.1650147  0.00754727
x            0.9029908  1.50367778
I(x^2)      -0.1682162 -0.09763807
```

#ALOGPs, ALOGPs-sq, nH

```
# now extend to include nH
dfnH <-data.frame(x=ds.sb$ALOGPs,y=ds.sb$Log.1.EC50., z=ds.sb$nH)
# usual least square fit and estimates
fitnH <- lm(y ~ x + I(x^2) + z, data = dfnH)
```

```
> summary(fitnH)

Call:
lm(formula = y ~ x + I(x^2) + z, data = dfnH)

Residuals:
     Min       1Q   Median       3Q      Max
-0.52009 -0.12461 -0.02989  0.14692  0.54953

Coefficients:
            Estimate Std. Error t value Pr(>|t|)
(Intercept) -1.65858    0.30541  -5.431 3.19e-06 ***
x            1.30991    0.11713  11.183 9.83e-14 ***
I(x^2)      -0.17580    0.01584 -11.101 1.23e-13 ***
z            0.06525    0.01247   5.233 5.98e-06 ***
---
Signif. codes:  0 '***' 0.001 '**' 0.01 '*' 0.05 '.' 0.1 ' ' 1

Residual standard error: 0.2372 on 39 degrees of freedom
Multiple R-squared:  0.7822,	Adjusted R-squared:  0.7654
F-statistic: 46.67 on 3 and 39 DF,  p-value: 5.615e-13

> confint(fitnH)
                  2.5 %     97.5 %
(Intercept) -2.27633322 -1.0408314
x            1.07298335  1.5468355
I(x^2)      -0.20783293 -0.1437676
z            0.04002894  0.0904637

# now bootstrap
res_fitnH <-boot(data = dfnH, statistic = bs, strata = strata, R=999, formula = y ~ x + I(x^2)+z)
#res_fitnH is including nH

# look at confidence intervals (now seem a little different to above)
#uses the bootstrap function
# index 1 - intercept?
# index 2 - ALOGPs
# index 3 - ALOGPs^2
# index 4 - nH
> boot.ci(res_fitnH, type="bca",index=1)
BOOTSTRAP CONFIDENCE INTERVAL CALCULATIONS
Based on 999 bootstrap replicates

CALL :
boot.ci(boot.out = res_fitnH, type = "bca", index = 1)

Intervals :
Level      BCa
95%   (-2.454, -1.033 )
Calculations and Intervals on Original Scale
> boot.ci(res_fitnH, type="bca",index=2)
BOOTSTRAP CONFIDENCE INTERVAL CALCULATIONS
Based on 999 bootstrap replicates

CALL :
boot.ci(boot.out = res_fitnH, type = "bca", index = 2)
```

Intervals :
Level   BCa
95%   (1.038, 1.601)
Calculations and Intervals on Original Scale
> boot.ci(res_fitnH, type="bca",index=3)
BOOTSTRAP CONFIDENCE INTERVAL CALCULATIONS
Based on 999 bootstrap replicates

CALL :
boot.ci(boot.out = res_fitnH, type = "bca", index = 3)

Intervals :
Level   BCa
95%   (-0.2132, -0.1302)
Calculations and Intervals on Original Scale
> boot.ci(res_fitnH, type="bca",index=4)
BOOTSTRAP CONFIDENCE INTERVAL CALCULATIONS
Based on 999 bootstrap replicates

CALL :
boot.ci(boot.out = res_fitnH, type = "bca", index = 4)

Intervals :
Level   BCa
95%   (0.0433, 0.0874)
Calculations and Intervals on Original Scale

#ALOGPs, ALOGPs-sq, Mv

```
# now extend to include Mv
dfMv <-data.frame(x=ds.sb$ALOGPs,y=ds.sb$Log.1.EC50., z=ds.sb$Mv)
# usual least square fit and estimates
fitMv <- lm(y ~ x + I(x^2) + z, data = dfMv)
#lines(ds.sb$ALOGPs[ord],fitted(fitMv)[ord],col="green")

summary(fitMv)
Call: lm(formula = y ~ x + I(x^2) + z, data = dfMv)

Residuals:
    Min       1Q   Median       3Q      Max
-0.62519 -0.09623 -0.00267  0.10307  0.50699

Coefficients:
            Estimate Std. Error t value Pr(>|t|)
(Intercept)  3.36237    0.75384   4.460 6.76e-05 ***
x            1.37214    0.11736  11.692 2.56e-14 ***
I(x^2)      -0.15793    0.01407 -11.227 8.75e-14 ***
z           -6.61570    1.20981  -5.468 2.83e-06 ***
---
Signif. codes:  0 '***' 0.001 '**' 0.01 '*' 0.05 '.' 0.1 ' ' 1

Residual standard error: 0.2328 on 39 degrees of freedom
Multiple R-squared:  0.7901,    Adjusted R-squared:  0.774
F-statistic: 48.93 on 3 and 39 DF,  p-value: 2.732e-13

confint(fitMv)
```

```
                2.5 %     97.5 %
(Intercept)  1.8375883  4.8871494
x           1.1347599  1.6095207
I(x^2)      -0.1863835 -0.1294758
z          -9.0627723 -4.1686238

# now bootstrap
res_fitMv <-boot(data = dfMv, statistic = bs, strata = strata, R=999, formula = y ~ x + I(x^2)+z)
#res_fitMv is including Mv

#uses the bootstrap function
# index 1 - intercept?
# index 2 - ALOGPs
# index 3 - ALOGPs^2
# index 4 - Mv

> boot.ci(res_fitMv, type="bca",index=1)
BOOTSTRAP CONFIDENCE INTERVAL CALCULATIONS
Based on 999 bootstrap replicates

CALL :
boot.ci(boot.out = res_fitMv, type = "bca", index = 1)

Intervals :
Level       BCa
95%   ( 2.159,  4.419 )
Calculations and Intervals on Original Scale
> boot.ci(res_fitMv, type="bca",index=2)
BOOTSTRAP CONFIDENCE INTERVAL CALCULATIONS
Based on 999 bootstrap replicates

CALL :
boot.ci(boot.out = res_fitMv, type = "bca", index = 2)

Intervals :
Level       BCa
95%   ( 1.126,  1.579 )
Calculations and Intervals on Original Scale
> boot.ci(res_fitMv, type="bca",index=3)
BOOTSTRAP CONFIDENCE INTERVAL CALCULATIONS
Based on 999 bootstrap replicates

CALL :
boot.ci(boot.out = res_fitMv, type = "bca", index = 3)

Intervals :
Level       BCa
95%   (-0.1895, -0.1229 )
Calculations and Intervals on Original Scale
> boot.ci(res_fitMv, type="bca",index=4)
BOOTSTRAP CONFIDENCE INTERVAL CALCULATIONS
Based on 999 bootstrap replicates

CALL :
boot.ci(boot.out = res_fitMv, type = "bca", index = 4)

Intervals :
```

```
Level      BCa
95%   (-8.432, -4.473 )
Calculations and Intervals on Original Scale
```

#ALOGPs, ALOGPs-sq, J3D

```
# now extend to include J3D
dfJ3D <-data.frame(x=ds.sb$ALOGPs,y=ds.sb$Log.1.EC50., z=ds.sb$J3D)
# usual least square fit and estimates
fitJ3D <- lm(y ~ x + I(x^2) + z, data = dfJ3D)
#lines(ds.sb$ALOGPs[ord],fitted(fitJ3D)[ord],col="green")

> summary(fitJ3D)
Call: lm(formula = y ~ x + I(x^2) + z, data = dfJ3D)

Residuals:
    Min       1Q   Median       3Q      Max
-0.60320 -0.11738  0.00310  0.09931  0.49719

Coefficients:
            Estimate Std. Error t value Pr(>|t|)
(Intercept)  -2.3723     0.3971  -5.973 5.64e-07 ***
x             1.3394     0.1162  11.527 3.94e-14 ***
I(x^2)       -0.1476     0.0136 -10.850 2.42e-13 ***
z             0.7332     0.1348   5.441 3.09e-06 ***
---
Signif. codes:  0 '***' 0.001 '**' 0.01 '*' 0.05 '.' 0.1 ' ' 1

Residual standard error: 0.2333 on 39 degrees of freedom
Multiple R-squared:  0.7892,          Adjusted R-squared:  0.773
F-statistic: 48.67 on 3 and 39 DF,  p-value: 2.971e-13

> confint(fitJ3D)
                 2.5 %     97.5 %
(Intercept) -3.1755219 -1.5689714
x            1.1043710  1.5744203
I(x^2)      -0.1750866 -0.1200634
z            0.4606703  1.0058329

# now bootstrap
res_fitJ3D <-boot(data = dfJ3D, statistic = bs, strata = strata, R=999, formula = y ~ x + I(x^2)+z)
#res_fitJ3D is including J3D

# look at confidence intervals (now seem a little different to above)
#uses the bootstrap function
# index 1 - intercept?
# index 2 - ALOGPs
# index 3 - ALOGPs^2
# index 4 - J3D
> boot.ci(res_fitJ3D, type="bca",index=1)
BOOTSTRAP CONFIDENCE INTERVAL CALCULATIONS
Based on 999 bootstrap replicates

CALL :
boot.ci(boot.out = res_fitJ3D, type = "bca", index = 1)
```

Intervals :
Level   BCa
95%   (-3.071, -1.648)
Calculations and Intervals on Original Scale
> boot.ci(res_fitJ3D, type="bca",index=2)
BOOTSTRAP CONFIDENCE INTERVAL CALCULATIONS
Based on 999 bootstrap replicates

CALL :
boot.ci(boot.out = res_fitJ3D, type = "bca", index = 2)

Intervals :
Level   BCa
95%   (1.109, 1.556)
Calculations and Intervals on Original Scale
> boot.ci(res_fitJ3D, type="bca",index=3)
BOOTSTRAP CONFIDENCE INTERVAL CALCULATIONS
Based on 999 bootstrap replicates

CALL :
boot.ci(boot.out = res_fitJ3D, type = "bca", index = 3)

Intervals :
Level   BCa
95%   (-0.1770, -0.1145)
Calculations and Intervals on Original Scale
> boot.ci(res_fitJ3D, type="bca",index=4)
BOOTSTRAP CONFIDENCE INTERVAL CALCULATIONS
Based on 999 bootstrap replicates

CALL :
boot.ci(boot.out = res_fitJ3D, type = "bca", index = 4)

Intervals :
Level   BCa
95%   (0.4952, 0.9602)
Calculations and Intervals on Original Scale
>

#ALOGPs, ALOGPs-sq, ARR

```
# now extend to include ARR
dfARR <-data.frame(x=ds.sb$ALOGPs,y=ds.sb$Log.1.EC50., z=ds.sb$ARR)
# usual least square fit and estimates
fitARR <- lm(y ~ x + I(x^2) + z, data = dfARR)

> summary(fitARR)
Call: lm(formula = y ~ x + I(x^2) + z, data = dfARR)

Residuals:
     Min       1Q   Median       3Q      Max
-0.65423 -0.09241  0.00022  0.10878  0.51673

Coefficients:
            Estimate Std. Error t value Pr(>|t|)
(Intercept) -0.22238    0.24537  -0.906     0.37
```

```
x           1.36602    0.12449  10.973 1.73e-13 ***
I(x^2)      -0.15271    0.01467 -10.412 8.05e-13 ***
z          -1.67499    0.35198  -4.759 2.67e-05 ***
---
Signif. codes:  0 '***' 0.001 '**' 0.01 '*' 0.05 '.' 0.1 ' ' 1

Residual standard error: 0.2461 on 39 degrees of freedom
Multiple R-squared:  0.7654,           Adjusted R-squared:  0.7473
F-statistic: 42.41 on 3 and 39 DF,  p-value: 2.358e-12

> confint(fitARR)
                 2.5 %     97.5 %
(Intercept) -0.7186996  0.2739332
x           1.1142092  1.6178228
I(x^2)      -0.1823700 -0.1230402
z          -2.3869397 -0.9630480

# now bootstrap
res_fitARR <-boot(data = dfARR, statistic = bs, strata = strata, R=999, formula = y ~ x + I(x^2)+z)
#res_fitARR is including ARR

# look at confidence intervals (now seem a little different to above)
#uses the bootstrap function
# index 1 - intercept?
# index 2 - ALOGPs
# index 3 - ALOGPs^2
# index 4 - ARR
> boot.ci(res_fitARR, type="bca",index=1)
BOOTSTRAP CONFIDENCE INTERVAL CALCULATIONS
Based on 999 bootstrap replicates

CALL :
boot.ci(boot.out = res_fitARR, type = "bca", index = 1)

Intervals :
Level       BCa
95%   (-0.6466,  0.0697 )
Calculations and Intervals on Original Scale
Some BCa intervals may be unstable
> boot.ci(res_fitARR, type="bca",index=2)
BOOTSTRAP CONFIDENCE INTERVAL CALCULATIONS
Based on 999 bootstrap replicates

CALL :
boot.ci(boot.out = res_fitARR, type = "bca", index = 2)

Intervals :
Level       BCa
95%   ( 1.117,  1.605 )
Calculations and Intervals on Original Scale
> boot.ci(res_fitARR, type="bca",index=3)
BOOTSTRAP CONFIDENCE INTERVAL CALCULATIONS
Based on 999 bootstrap replicates

CALL :
boot.ci(boot.out = res_fitARR, type = "bca", index = 3)
```

```
Intervals :
Level      BCa
95%   (-0.1863, -0.1160 )
Calculations and Intervals on Original Scale
> boot.ci(res_fitARR, type="bca",index=4)
BOOTSTRAP CONFIDENCE INTERVAL CALCULATIONS
Based on 999 bootstrap replicates

CALL :
boot.ci(boot.out = res_fitARR, type = "bca", index = 4)

Intervals :
Level      BCa
95%   (-2.290, -1.065 )
Calculations and Intervals on Original Scale
```

#ALOGPs, ALOGPs-sq, AMW

```
# now extend to include AMW
dfAMW <-data.frame(x=ds.sb$ALOGPs,y=ds.sb$Log.1.EC50., z=ds.sb$AMW)
# usual least square fit and estimates
fitAMW <- lm(y ~ x + I(x^2) + z, data = dfAMW)

> summary(fitAMW)
Call: lm(formula = y ~ x + I(x^2) + z, data = dfAMW)

Residuals:
    Min       1Q   Median       3Q      Max
-0.61861 -0.12959  0.02152  0.15637  0.41613

Coefficients:
            Estimate Std. Error t value Pr(>|t|)
(Intercept)  0.73091    0.34409   2.124     0.04 *
x            1.33046    0.11940  11.143 1.09e-13 ***
I(x^2)      -0.15404    0.01433 -10.747 3.20e-13 ***
z           -0.19952    0.03928  -5.080 9.72e-06 ***
---
Signif. codes:  0 ‘***’ 0.001 ‘**’ 0.01 ‘*’ 0.05 ‘.’ 0.1 ‘ ’ 1

Residual standard error: 0.2401 on 39 degrees of freedom
Multiple R-squared:  0.7768,          Adjusted R-squared:  0.7597
F-statistic: 45.25 on 3 and 39 DF,  p-value: 8.955e-13

> confint(fitAMW)
                  2.5 %     97.5 %
(Intercept)  0.03492571  1.4268963
x            1.08895355  1.5719752
I(x^2)      -0.18302806 -0.1250454
z           -0.27896332 -0.1200808

# now bootstrap
res_fitAMW <-boot(data = dfAMW, statistic = bs, strata = strata, R=999, formula = y ~ x + I(x^2)+z)
#res_fitAMW is including AMW

# look at confidence intervals (now seem a little different to above)
```

```
#uses the bootstrap function
# index 1 - intercept?
# index 2 - ALOGPs
# index 3 - ALOGPs^2
# index 4 - AMW
> boot.ci(res_fitAMW, type="bca",index=1)
BOOTSTRAP CONFIDENCE INTERVAL CALCULATIONS
Based on 999 bootstrap replicates

CALL :
boot.ci(boot.out = res_fitAMW, type = "bca", index = 1)

Intervals :
Level      BCa
95%   (-0.1126,  1.3684 )
Calculations and Intervals on Original Scale
Some BCa intervals may be unstable
> boot.ci(res_fitAMW, type="bca",index=2)
BOOTSTRAP CONFIDENCE INTERVAL CALCULATIONS
Based on 999 bootstrap replicates

CALL :
boot.ci(boot.out = res_fitAMW, type = "bca", index = 2)

Intervals :
Level      BCa
95%   ( 1.104,  1.536 )
Calculations and Intervals on Original Scale
> boot.ci(res_fitAMW, type="bca",index=3)
BOOTSTRAP CONFIDENCE INTERVAL CALCULATIONS
Based on 999 bootstrap replicates

CALL :
boot.ci(boot.out = res_fitAMW, type = "bca", index = 3)

Intervals :
Level      BCa
95%   (-0.1820, -0.1212 )
Calculations and Intervals on Original Scale
> boot.ci(res_fitAMW, type="bca",index=4)
BOOTSTRAP CONFIDENCE INTERVAL CALCULATIONS
Based on 999 bootstrap replicates

CALL :
boot.ci(boot.out = res_fitAMW, type = "bca", index = 4)

Intervals :
Level      BCa
95%   (-0.2931, -0.1331 )
Calculations and Intervals on Original Scale
Some BCa intervals may be unstable
```

#ALOGPS, ALOGPs-sq, nCIC, J3D

```
#ALogPs, ALogPs^2, nCIC, J3D
dfnCIC_J3D <-data.frame(x=ds.sb$ALOGPs,y=ds.sb$Log.1.EC50., z=ds.sb$nCIC, w=ds.sb$J3D)
# usual least square fit and estimates
```

```
fitnCIC_J3D <- lm(y ~ x + I(x^2) + z + w, data = dfnCIC_J3D)

> summary(fitnCIC_J3D)
Call: lm(formula = y ~ x + I(x^2) + z + w, data = dfnCIC_J3D)

Residuals:
     Min       1Q   Median       3Q      Max
-0.49204 -0.14596  0.03286  0.09284  0.47415

Coefficients:
            Estimate Std. Error t value Pr(>|t|)
(Intercept)  -5.1054     1.2220  -4.178 0.000166 ***
x             1.2837     0.1125  11.409 7.73e-14 ***
I(x^2)       -0.1457     0.0129 -11.295 1.04e-13 ***
z             0.4105     0.1746   2.351 0.024036 *
w             1.5873     0.3851   4.122 0.000196 ***
---
Signif. codes:  0 ‘***’ 0.001 ‘**’ 0.01 ‘*’ 0.05 ‘.’ 0.1 ‘ ’ 1

Residual standard error: 0.2209 on 38 degrees of freedom
Multiple R-squared:  0.816,	Adjusted R-squared:  0.7966
F-statistic: 42.12 on 4 and 38 DF,  p-value: 1.784e-13

> confint(fitnCIC_J3D)
                  2.5 %     97.5 %
(Intercept) -7.57929116 -2.6315691
x            1.05592808  1.5114640
I(x^2)      -0.17181680 -0.1195890
z            0.05696876  0.7640868
w            0.80774675  2.3668022


# now bootstrap
res_fitnCIC_J3D <-boot(data = dfnCIC_J3D, statistic = bs, strata = strata, R=999, formula = y ~ x + I(x^2)+z +w)
#res_fitnCIC_J3D is including nCIC_J3D

# look at confidence intervals - uses the bootstrap function
# index 1 - intercept?
# index 2 - ALOGPs
# index 3 - ALOGPs^2
# index 4 - nCIC
# index 5 - J3D
> boot.ci(res_fitnCIC_J3D, type="bca",index=1)
BOOTSTRAP CONFIDENCE INTERVAL CALCULATIONS
Based on 999 bootstrap replicates

CALL :
boot.ci(boot.out = res_fitnCIC_J3D, type = "bca", index = 1)

Intervals :
Level       BCa
95%   (-7.681, -2.694 )
Calculations and Intervals on Original Scale
> boot.ci(res_fitnCIC_J3D, type="bca",index=2)
BOOTSTRAP CONFIDENCE INTERVAL CALCULATIONS
Based on 999 bootstrap replicates
```

```
CALL :
boot.ci(boot.out = res_fitnCIC_J3D, type = "bca", index = 2)

Intervals :
Level      BCa
95%   ( 1.087,  1.493 )
Calculations and Intervals on Original Scale
> boot.ci(res_fitnCIC_J3D, type="bca",index=3)
BOOTSTRAP CONFIDENCE INTERVAL CALCULATIONS
Based on 999 bootstrap replicates

CALL :
boot.ci(boot.out = res_fitnCIC_J3D, type = "bca", index = 3)

Intervals :
Level      BCa
95%   (-0.1730, -0.1161 )
Calculations and Intervals on Original Scale
> boot.ci(res_fitnCIC_J3D, type="bca",index=4)
BOOTSTRAP CONFIDENCE INTERVAL CALCULATIONS
Based on 999 bootstrap replicates

CALL :
boot.ci(boot.out = res_fitnCIC_J3D, type = "bca", index = 4)

Intervals :
Level      BCa
95%   ( 0.0638,  0.7957 )
Calculations and Intervals on Original Scale
> boot.ci(res_fitnCIC_J3D, type="bca",index=5)
BOOTSTRAP CONFIDENCE INTERVAL CALCULATIONS
Based on 999 bootstrap replicates

CALL :
boot.ci(boot.out = res_fitnCIC_J3D, type = "bca", index = 5)

Intervals :
Level      BCa
95%   ( 0.796,  2.330 )
Calculations and Intervals on Original Scale
```

#ALOGPs, ALOGPs-sq, AMW, J3D

```
#ALogPs, ALogPs^2, AMW, J3D
dfAMW_J3D <-data.frame(x=ds.sb$ALOGPs,y=ds.sb$Log.1.EC50., z=ds.sb$AMW, w=ds.sb$J3D)
# usual least square fit and estimates
fitAMW_J3D <- lm(y ~ x + I(x^2) + z + w, data = dfAMW_J3D)

> summary(fitAMW_J3D)
Call: lm(formula = y ~ x + I(x^2) + z + w, data = dfAMW_J3D)

Residuals:
    Min       1Q   Median       3Q      Max
-0.58907 -0.10352  0.01421  0.08473  0.47939

Coefficients:
```

```
            Estimate Std. Error t value Pr(>|t|)
(Intercept) -1.01799    0.70868  -1.436  0.15905
x            1.36148    0.11096  12.270 8.67e-15 ***
I(x^2)      -0.15404    0.01325 -11.625 4.43e-14 ***
z           -0.10996    0.04868  -2.259  0.02973 *
w            0.47467    0.17187   2.762  0.00881 **
---
Signif. codes:  0 ‘***’ 0.001 ‘**’ 0.01 ‘*’ 0.05 ‘.’ 0.1 ‘ ’ 1

Residual standard error: 0.2219 on 38 degrees of freedom
Multiple R-squared:  0.8141,	Adjusted R-squared:  0.7946
F-statistic: 41.61 on 4 and 38 DF,  p-value: 2.144e-13

> confint(fitAMW_J3D)
                 2.5 %      97.5 %
(Intercept) -2.4526500  0.41666156
x            1.1368500  1.58610083
I(x^2)      -0.1808690 -0.12721712
z           -0.2085162 -0.01140107
w            0.1267242  0.82260733

# now bootstrap
res_fitAMW_J3D <-boot(data = dfAMW_J3D, statistic = bs, strata = strata, R=999, formula = y ~ x + I(x^2)+z +w)
#res_fitAMW_J3D is including AMW_J3D

# look at confidence intervals - uses the bootstrap function
# index 1 - intercept?
# index 2 - ALOGPs
# index 3 - ALOGPs^2
# index 4 - AMW
# index 5 - J3D
> boot.ci(res_fitAMW_J3D, type="bca",index=1)
BOOTSTRAP CONFIDENCE INTERVAL CALCULATIONS
Based on 999 bootstrap replicates

CALL :
boot.ci(boot.out = res_fitAMW_J3D, type = "bca", index = 1)

Intervals :
Level       BCa
95%   (-2.309,  0.429 )
Calculations and Intervals on Original Scale
> boot.ci(res_fitAMW_J3D, type="bca",index=2)
BOOTSTRAP CONFIDENCE INTERVAL CALCULATIONS
Based on 999 bootstrap replicates

CALL :
boot.ci(boot.out = res_fitAMW_J3D, type = "bca", index = 2)

Intervals :
Level       BCa
95%   ( 1.123,  1.591 )
Calculations and Intervals on Original Scale
> boot.ci(res_fitAMW_J3D, type="bca",index=3)
BOOTSTRAP CONFIDENCE INTERVAL CALCULATIONS
Based on 999 bootstrap replicates
```

```
CALL :
boot.ci(boot.out = res_fitAMW_J3D, type = "bca", index = 3)

Intervals :
Level      BCa
95%   (-0.1852, -0.1194 )
Calculations and Intervals on Original Scale
> boot.ci(res_fitAMW_J3D, type="bca",index=4)
BOOTSTRAP CONFIDENCE INTERVAL CALCULATIONS
Based on 999 bootstrap replicates

CALL :
boot.ci(boot.out = res_fitAMW_J3D, type = "bca", index = 4)

Intervals :
Level      BCa
95%   (-0.2182, -0.0512 )
Calculations and Intervals on Original Scale
> boot.ci(res_fitAMW_J3D, type="bca",index=5)
BOOTSTRAP CONFIDENCE INTERVAL CALCULATIONS
Based on 999 bootstrap replicates

CALL :
boot.ci(boot.out = res_fitAMW_J3D, type = "bca", index = 5)

Intervals :
Level      BCa
95%   ( 0.1479,  0.7805 )
Calculations and Intervals on Original Scale
```

#ALOGPs, ALOGPs-sq, nH, J

```
#ALogPs, ALogPs^2, nH, J
dfnH_J <-data.frame(x=ds.sb$ALOGPs,y=ds.sb$Log.1.EC50., z=ds.sb$nH, w=ds.sb$J)
# usual least square fit and estimates
fitnH_J <- lm(y ~ x + I(x^2) + z + w, data = dfnH_J)

> summary(fitnH_J)
Call: lm(formula = y ~ x + I(x^2) + z + w, data = dfnH_J)

Residuals:
    Min       1Q   Median       3Q      Max
-0.51391 -0.10098 -0.02125  0.07999  0.54360

Coefficients:
            Estimate Std. Error t value Pr(>|t|)
(Intercept) -3.06707    0.61117  -5.018 1.25e-05 ***
x            1.43233    0.11898  12.039 1.55e-14 ***
I(x^2)      -0.17239    0.01484 -11.620 4.49e-14 ***
z            0.04414    0.01418   3.113  0.00351 **
w            0.90448    0.34719   2.605  0.01304 *
---
Signif. codes:  0 '***' 0.001 '**' 0.01 '*' 0.05 '.' 0.1 ' ' 1

Residual standard error: 0.2213 on 38 degrees of freedom
Multiple R-squared:  0.8152,	Adjusted R-squared:  0.7957
```

```
F-statistic:  41.9 on 4 and 38 DF,  p-value: 1.933e-13

> confint(fitnH_J)
                 2.5 %      97.5 %
(Intercept) -4.30432090 -1.82982567
x            1.19146973  1.67318501
I(x^2)      -0.20242439 -0.14235652
z            0.01543404  0.07283738
w            0.20163869  1.60732816
>

# now bootstrap
res_fitnH_J <-boot(data = dfnH_J, statistic = bs, strata = strata, R=999, formula = y ~ x + I(x^2)+z +w)
#res_fitnH_J is including nH_J

# look at confidence intervals - uses the bootstrap function
# index 1 - intercept?
# index 2 - ALOGPs
# index 3 - ALOGPs^2
# index 4 - nH
# index 5 - J
> boot.ci(res_fitnH_J, type="bca",index=1)
BOOTSTRAP CONFIDENCE INTERVAL CALCULATIONS
Based on 999 bootstrap replicates

CALL :
boot.ci(boot.out = res_fitnH_J, type = "bca", index = 1)

Intervals :
Level       BCa
95%   (-4.133, -1.767 )
Calculations and Intervals on Original Scale
> boot.ci(res_fitnH_J, type="bca",index=2)
BOOTSTRAP CONFIDENCE INTERVAL CALCULATIONS
Based on 999 bootstrap replicates

CALL :
boot.ci(boot.out = res_fitnH_J, type = "bca", index = 2)

Intervals :
Level       BCa
95%   ( 1.196,  1.653 )
Calculations and Intervals on Original Scale
> boot.ci(res_fitnH_J, type="bca",index=3)
BOOTSTRAP CONFIDENCE INTERVAL CALCULATIONS
Based on 999 bootstrap replicates

CALL :
boot.ci(boot.out = res_fitnH_J, type = "bca", index = 3)

Intervals :
Level       BCa
95%   (-0.2047, -0.1278 )
Calculations and Intervals on Original Scale
> boot.ci(res_fitnH_J, type="bca",index=4)
BOOTSTRAP CONFIDENCE INTERVAL CALCULATIONS
Based on 999 bootstrap replicates
```

```
CALL :
boot.ci(boot.out = res_fitnH_J, type = "bca", index = 4)

Intervals :
Level     BCa
95%   ( 0.0139,  0.0705 )
Calculations and Intervals on Original Scale
> boot.ci(res_fitnH_J, type="bca",index=5)
BOOTSTRAP CONFIDENCE INTERVAL CALCULATIONS
Based on 999 bootstrap replicates

CALL :
boot.ci(boot.out = res_fitnH_J, type = "bca", index = 5)

Intervals :
Level     BCa
95%   ( 0.1729,  1.4745 )
Calculations and Intervals on Original Scale
```

#ALOGPs, ALOGPs-sq, Polarizability, nH

```
#ALogPs, ALogPs^2, Polarizability..cm3., nH
dfPol_nH <-data.frame(x=ds.sb$ALOGPs,y=ds.sb$Log.1.EC50., z=ds.sb$Polarizability..cm3., w=ds.sb$nH)
# usual least square fit and estimates
fitPol_nH <- lm(y ~ x + I(x^2) + z + w, data = dfPol_nH)

> summary(fitPol_nH)
Call: lm(formula = y ~ x + I(x^2) + z + w, data = dfPol_nH)

Residuals:
     Min       1Q   Median       3Q      Max
-0.57511 -0.10224 -0.02149  0.08645  0.58694

Coefficients:
             Estimate Std. Error t value Pr(>|t|)
(Intercept) -0.983048   0.414418  -2.372   0.0229 *
x            1.306968   0.111287  11.744 3.26e-14 ***
I(x^2)      -0.164507   0.015837 -10.387 1.18e-12 ***
z           -0.021601   0.009462  -2.283   0.0281 *
w            0.062608   0.011900   5.261 5.86e-06 ***
---
Signif. codes:  0 ‘***’ 0.001 ‘**’ 0.01 ‘*’ 0.05 ‘.’ 0.1 ‘ ’ 1

Residual standard error: 0.2253 on 38 degrees of freedom
Multiple R-squared:  0.8084,     Adjusted R-squared:  0.7883
F-statistic: 40.09 on 4 and 38 DF,  p-value: 3.786e-13

> confint(fitPol_nH)
                  2.5 %       97.5 %
(Intercept) -1.82199334 -0.144102492
x            1.08167950  1.532255543
I(x^2)      -0.19656852 -0.132446274
z           -0.04075559 -0.002446523
w            0.03851716  0.086699013
```

```
fitPol_nH_pred<-fitted(fitPol_nH) #predicted values of fit3
# plot(fitPol_nH, dfPol_nH$y, main="fitPol_nH predicted vs actual") # plots predicted vs actual values

# now bootstrap
res_fitPol_nH <-boot(data = dfPol_nH, statistic = bs, strata = strata, R=999, formula = y ~ x + I(x^2)+z +w)
#res_fitPol_nH is including Pol_nH

# look at confidence intervals - uses the bootstrap function
# index 1 - intercept?
# index 2 - ALOGPs
# index 3 - ALOGPs^2
# index 4 - Molec.Volume..cm3.
# index 5 - nH
> boot.ci(res_fitPol_nH, type="bca",index=1)
BOOTSTRAP CONFIDENCE INTERVAL CALCULATIONS
Based on 999 bootstrap replicates

CALL :
boot.ci(boot.out = res_fitPol_nH, type = "bca", index = 1)

Intervals :
Level      BCa
95%   (-1.7356,  0.0784 )
Calculations and Intervals on Original Scale
> boot.ci(res_fitPol_nH, type="bca",index=2)
BOOTSTRAP CONFIDENCE INTERVAL CALCULATIONS
Based on 999 bootstrap replicates

CALL :
boot.ci(boot.out = res_fitPol_nH, type = "bca", index = 2)

Intervals :
Level      BCa
95%   ( 1.032,  1.519 )
Calculations and Intervals on Original Scale
> boot.ci(res_fitPol_nH, type="bca",index=3)
BOOTSTRAP CONFIDENCE INTERVAL CALCULATIONS
Based on 999 bootstrap replicates

CALL :
boot.ci(boot.out = res_fitPol_nH, type = "bca", index = 3)

Intervals :
Level      BCa
95%   (-0.1990, -0.1102 )
Calculations and Intervals on Original Scale
> boot.ci(res_fitPol_nH, type="bca",index=4)
BOOTSTRAP CONFIDENCE INTERVAL CALCULATIONS
Based on 999 bootstrap replicates

CALL :
boot.ci(boot.out = res_fitPol_nH, type = "bca", index = 4)

Intervals :
Level      BCa
95%   (-0.0363,  0.0050 )
```

```
Calculations and Intervals on Original Scale
> boot.ci(res_fitPol_nH, type="bca",index=5)
BOOTSTRAP CONFIDENCE INTERVAL CALCULATIONS
Based on 999 bootstrap replicates

CALL :
boot.ci(boot.out = res_fitPol_nH, type = "bca", index = 5)

Intervals :
Level       BCa
95%   ( 0.0399,  0.0865 )
Calculations and Intervals on Original Scale
>
```

3.6. R Code from mixed effect fits

R Code & results from mixed effect fits using lme4 library.

```
dataset<-read.csv("Tambjamines_New_numbers_classified_cleaned.csv")
ds_alkyl <- subset(dataset, R.type =="alkyl")

ds_OMe_alkyl <- subset(dataset, R.type =="alkyl" & Ring.substituent == "OMe")
#subset

#plot the points for subset 1 and then label them by their Rgroup (NH.substituent) + colour by top substituent

#plot with labels and title, labels each point with Nh.substituent and colour by top substituent
plot(ds_OMe_alkyl$ALOGPs,ds_OMe_alkyl$Log.1.EC50., main="Plot of Log(1/EC50) against ALOGPs,
alkyl chain only, OMe ring substituent", ylim=c(0.4,3.0), xlab="ALOGPs", ylab="Log(1/EC50)",
text(ds_OMe_alkyl$ALOGPs,ds_OMe_alkyl$Log.1.EC50., ds_OMe_alkyl$NH.substituent, cex=0.5, pos=3),
col=(ifelse(ds_OMe_alkyl$Enamine.Substituent=='NH', "red", "blue")))


plot(ds_OBn_alkyl$ALOGPs,ds_OBn_alkyl$Log.1.EC50., main="Plot of Log(1/EC50) against ALOGPs,
alkyl chain only, OBn ring substituent", ylim=c(0.4,3.0), xlab="ALOGPs", ylab="Log(1/EC50)",
text(ds_OBn_alkyl$ALOGPs,ds_OBn_alkyl$Log.1.EC50., ds_OBn_alkyl$NH.substituent, cex=0.5, pos=3),
col=(ifelse(ds_OBn_alkyl$Enamine.Substituent=='NH', "red", "blue")))

#plot with the 2 different Enamine.Substituents in different colours

plot(ds_alkyl$ALOGPs,ds_alkyl$Log.1.EC50.,col=ds_alkyl$Enamine.Substituent, pch=c(16,
17)[as.numeric(ds_alkyl$Ring.substituent)], main="Plot of Log(1/EC50) vs ALOGPs - alkyl R chain", sub="Points
coloured by Enamine Substituent type", ylim=c(0.4,3.0),xlab="ALOGPs",ylab="Log(1/EC50)")
# triangle – OMe
# circle – OBn
# black – NH
# green - NH-Ph
> # Try a parabolic fit, with the maximum and curator the same for both sets but different intercept
> # This uses fixed and random effects by group so use lme4 library
>
> library(lme4)
>
> # formula has the random or grouping effects of Enamine.Substituent
> fit_l_alkyl <- lmer(data = ds_alkyl, Log.1.EC50. ~ (1|Enamine.Substituent) + ALOGPs + I(ALOGPs^2))
>
> summary(fit_l_alkyl)
Linear mixed model fit by REML ['lmerMod']
Formula: Log.1.EC50. ~ (1 | Enamine.Substituent) + ALOGPs + I(ALOGPs^2)
Data: ds_alkyl
REML criterion at convergence: 16.6

Scaled residuals:
   Min      1Q  Median      3Q     Max
-2.3650 -0.3438  0.1081  0.5481  1.6244

Random effects:
 Groups              Name        Variance Std.Dev.
 Enamine.Substituent (Intercept) 0.03059  0.1749
```

```
 Residual                    0.06238  0.2498
Number of obs: 28, groups:  Enamine.Substituent, 2

Fixed effects:
          Estimate Std. Error t value
(Intercept) -0.73933    0.32341  -2.286
ALOGPs       1.37279    0.14548   9.436
I(ALOGPs^2) -0.15605    0.01661  -9.393

Correlation of Fixed Effects:
          (Intr) ALOGPs
ALOGPs     -0.881
I(ALOGPs^2)  0.814 -0.979
>
> coef(fit_l_alkyl)
$Enamine.Substituent
      (Intercept)   ALOGPs I(ALOGPs^2)
NH     -0.6254821 1.372787  -0.1560464
NH-Ph  -0.8531819 1.372787  -0.1560464

attr(,"class")
[1] "coef.mer"
>
> # calculate their 3 fitted curves and plot
> # define a fine x grid to calculate the curves
> x <- (0:80)/10
>
> # coef(fit_l_alkyl) gives the coefficients for each group (fixed and random combined).
> # (coef(fit_l_alkyl)$Enamine.Substituent)gives the table of pluses in effect a matrix from which we can
> # extract the numbers, first index is the group and can be a numeric index or use the name
> # (i.e. "NH") and second index is the coefficient (intercept, ALOGP etc)
>
> # coef(fit_l_alkyl) is a matrix of coefficients?
>
> # y1 takes the first line - NH
> y1 <-
(coef(fit_l_alkyl)$Enamine.Substituent)[1,1]+(coef(fit_l_alkyl)$Enamine.Substituent)[1,2]*x+(coef(fit_l_alkyl)$En
amine.Substituent)[1,3]*x*x
>
>
> #add line to the plot
> lines(x,y1,col="black")
>
> # y2 takes the second line - NH-Ph
> y2 <-
(coef(fit_l_alkyl)$Enamine.Substituent)[2,1]+(coef(fit_l_alkyl)$Enamine.Substituent)[2,2]*x+(coef(fit_l_alkyl)$En
amine.Substituent)[2,3]*x*x
>
> lines(x,y2,col="green")
>
>
> #
> #Just the OMe subset
> #
> #plot with the 2 different Enamine.Substituents in different colours
> plot(ds_OMe_alkyl$ALOGPs,ds_OMe_alkyl$Log.1.EC50.,col=ds_OMe_alkyl$Enamine.Substituent, pch=c(16,
17)[as.numeric(ds_OMe_alkyl$Ring.substituent)], main="Plot of Log(1/EC50) vs ALOGPs -
```

```
+ alkyl R chain, OMe ring substituent", sub="Points coloured by Enamine Substituent type",
ylim=c(0.4,3.0),xlab="ALOGPs",ylab="Log(1/EC50)")
> # triangle - OMe
> # black - NH
> # green - NH-Ph
>
>
> # Try a parabolic fit, with the maximum and curator the same for both sets but different intercept
> # This uses fixed and random effects by group so use lme4 library
>
> library(lme4)
>
> # formula has the random(?) or grouping effects of Top.Substituent
> fit_l_OMe_alkyl <- lmer(data = ds_OMe_alkyl, Log.1.EC50. ~ (1|Top.Substituent) + ALOGPs + I(ALOGPs^2))
>
> summary(fit_l_OMe_alkyl)
Linear mixed model fit by REML ['lmerMod']
Formula: Log.1.EC50. ~ (1 | Enamine.Substituent) + ALOGPs + I(ALOGPs^2)
Data: ds_OMe_alkyl

REML criterion at convergence: -5.4

Scaled residuals:
   Min      1Q  Median      3Q     Max
-1.3440 -0.5360 -0.1963  0.2020  2.1088

Random effects:
 Groups              Name        Variance Std.Dev.
 Enamine.Substituent (Intercept) 0.06455  0.2541
 Residual                        0.01738  0.1318
Number of obs: 20, groups:  Enamine.Substituent, 2

Fixed effects:
            Estimate Std. Error t value
(Intercept) -1.13221    0.24732  -4.578
ALOGPs       1.63726    0.08750  18.712
I(ALOGPs^2) -0.19202    0.01069 -17.960

Correlation of Fixed Effects:
            (Intr) ALOGPs
ALOGPs      -0.649
I(ALOGPs^2)  0.586 -0.971

> coef(fit_l_OMe_alkyl)
$Enamine.Substituent
      (Intercept)   ALOGPs I(ALOGPs^2)
NH     -0.9556585 1.637261  -0.1920198
NH-Ph  -1.3087691 1.637261  -0.1920198

attr(,"class")
[1] "coef.mer"

>
> # calculate their 3 fitted curves and plot
> # define a fine x grid to calculate the curves
> x <- (0:80)/10
>
> # coef(fit_l_OMe_alkyl) gives the coefficients for each group (fixed and random combined).
> # (coef(fit_l_OMe_alkyl)$Enamine.Substituent)gives the table of pluses in effect a matrix from which we can
```

```
> # extract the numbers, first index is the group and can be a numeric index or use the name
> # (i.e. "NH") and second index is the coefficient (intercept, ALOGP etc)
>
> # coef(fit_l_OMe_alkyl) is a matrix of coefficients?
>
> # y1 takes the first line - NH
> y1 <-
(coef(fit_l_OMe_alkyl)$Enamine.Substituent)[1,1]+(coef(fit_l_OMe_alkyl)$Enamine.Substituent)[1,2]*x+(coef(fit
_l_OMe_alkyl)$Enamine.Substituent)[1,3]*x*x
>
>
> #add line to the plot
> lines(x,y1,col="black")
>
> # y2 takes the second line - NH-Ph
> y2 <-
(coef(fit_l_OMe_alkyl)$Enamine.Substituent)[2,1]+(coef(fit_l_OMe_alkyl)$Enamine.Substituent)[2,2]*x+(coef(fit
_l_OMe_alkyl)$Enamine.Substituent)[2,3]*x*x
>
> lines(x,y2,col="green")
```
