# Supplementary material for: QSAR analysis of substituent effects on tambjamine anion transporters
Source: Chem Sci. 2015 Dec 8;7(2):1600–8. doi: 10.1039/c5sc03932k (PMC5964965; doi:10.1039/c5sc03932k)

27/07/2015 12:25
Data Table=Tambjamines_New_numbers_classified_cleaned

Multivariate

Correlations

	Retention time	TorchLiteSlogP	ALOGPs	miLOGP	AC LogP	ALOGP	MLOGP	KOWWIN	XLOGP2	XLOGP3	Log P	LogP (AB/LogP v2.0)	LogP (ACD/Labs)
Retention time	1.0000	0.9419	0.9858	0.9784	0.9802	0.9585	0.9421	0.9782	0.9760	0.9730	0.9741	0.9616	0.9643
TorchLiteSlogP	0.9419	1.0000	0.9770	0.9693	0.9762	0.9856	0.9298	0.9799	0.9631	0.9655	0.9825	0.9288	0.9301
ALOGPs	0.9858	0.9770	1.0000	0.9921	0.9941	0.9819	0.9656	0.9916	0.9855	0.9897	0.9896	0.9534	0.9648
miLOGP	0.9784	0.9693	0.9921	1.0000	0.9985	0.9776	0.9784	0.9929	0.9935	0.9975	0.9917	0.9674	0.9824
AC LogP	0.9802	0.9762	0.9941	0.9985	1.0000	0.9823	0.9741	0.9964	0.9924	0.9959	0.9946	0.9639	0.9786
ALOGP	0.9585	0.9856	0.9819	0.9776	0.9823	1.0000	0.9358	0.9826	0.9729	0.9807	0.9907	0.9320	0.9379
MLOGP	0.9421	0.9298	0.9656	0.9784	0.9741	0.9358	1.0000	0.9644	0.9789	0.9750	0.9621	0.9557	0.9759
KOWWIN	0.9782	0.9799	0.9916	0.9929	0.9964	0.9826	0.9644	1.0000	0.9862	0.9895	0.9957	0.9513	0.9699
XLOGP2	0.9760	0.9631	0.9855	0.9935	0.9924	0.9729	0.9789	0.9862	1.0000	0.9921	0.9860	0.9622	0.9804
XLOGP3	0.9730	0.9655	0.9897	0.9975	0.9959	0.9807	0.9750	0.9895	0.9921	1.0000	0.9899	0.9652	0.9780
Log P	0.9741	0.9825	0.9896	0.9917	0.9946	0.9907	0.9621	0.9957	0.9860	0.9899	1.0000	0.9485	0.9654
LogP (AB/LogP v2.0)	0.9616	0.9288	0.9534	0.9674	0.9639	0.9320	0.9557	0.9513	0.9622	0.9652	0.9485	1.0000	0.9789
LogP (ACD/Labs)	0.9643	0.9301	0.9648	0.9824	0.9786	0.9379	0.9759	0.9699	0.9804	0.9780	0.9654	0.9789	1.0000

There are 4 missing values. The correlations are estimated by Pairwise method.

Scatterplot Matrix

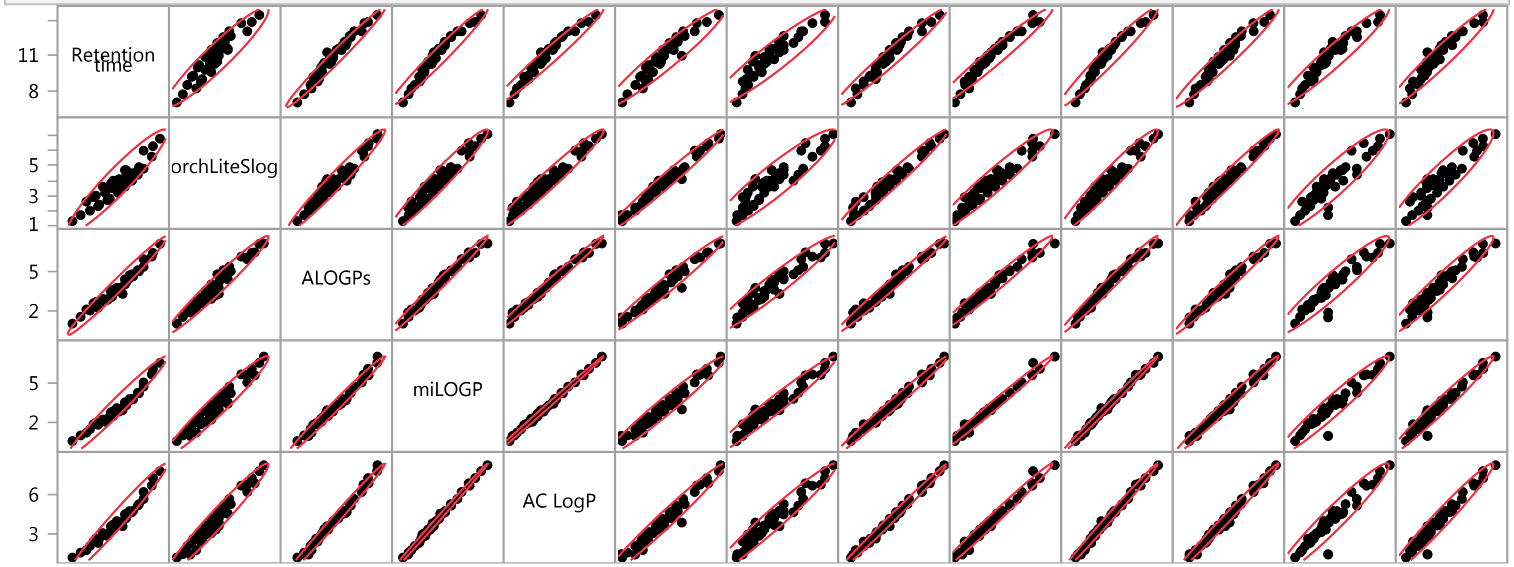

Multivariate

Scatterplot Matrix

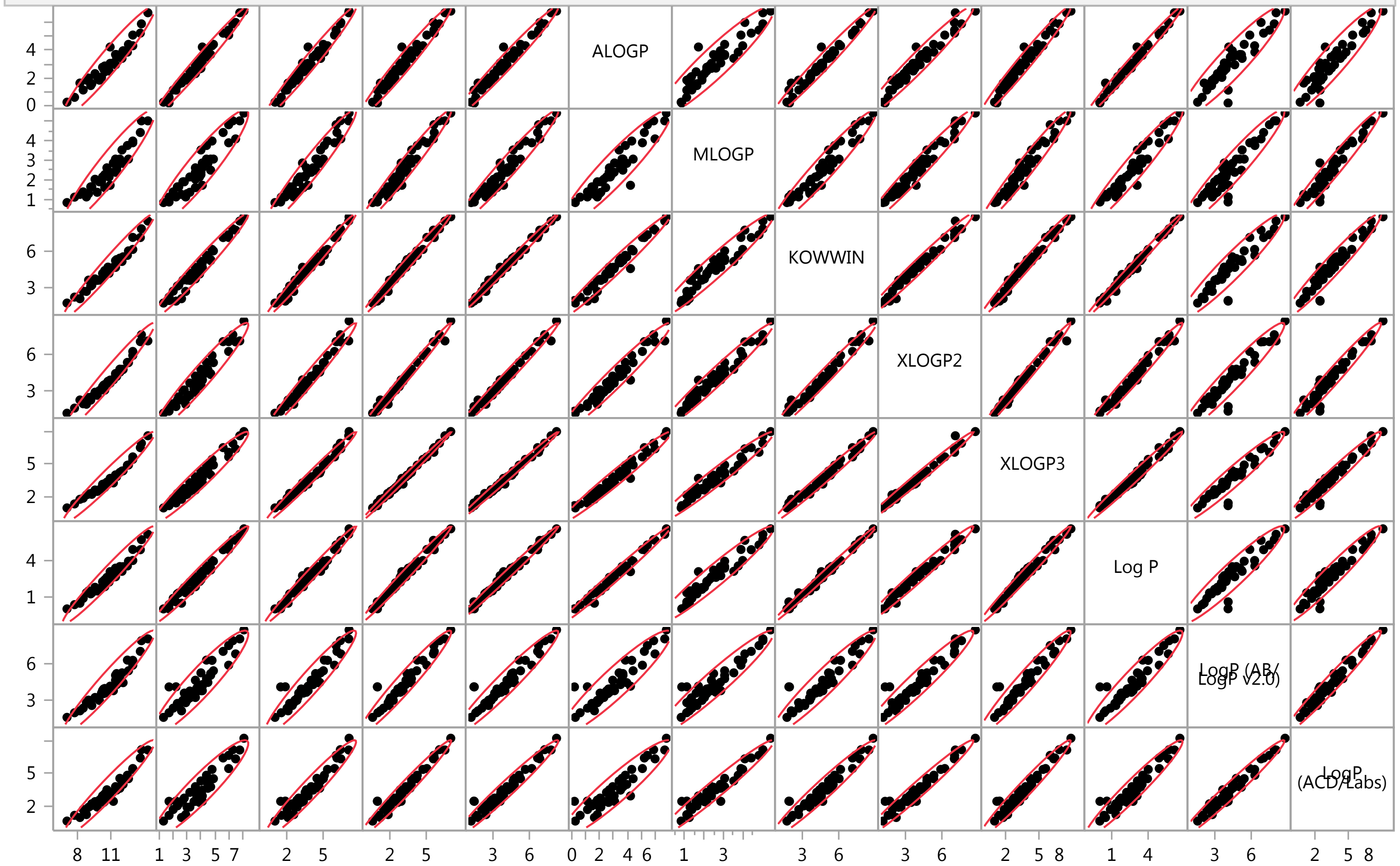

Supplement: Supplementary file 4 [file SC-007-C5SC03932K-s004.zip › logp_rt_correlations.pdf]
