# Supplementary material for: QSAR analysis of substituent effects on tambjamine anion transporters
Source: Chem Sci. 2015 Dec 8;7(2):1600–8. doi: 10.1039/c5sc03932k (PMC5964965; doi:10.1039/c5sc03932k)
Supplement: Supplementary file 4 [file SC-007-C5SC03932K-s004.zip › esi.doc]

**Supporting Information**

QSAR analysis of substituent effects on tambjamine anion transporters

Nicola J. Knight,*^a^* Elsa Hernando,*^b^* Cally J. E. Haynes,*^a^* Nathalie Busschaert, *^a^* Harriet J. Clarke, *^a^* Koji Takimoto, *^c^* María García-Valverde,*^b^* Jeremy G. Frey,*^a^* Roberto Quesada*^b^* and Philip A. Gale*^a^*

^a^ Chemistry, University of Southampton, Southampton, SO17 1BJ, UK. E-mail: philip.gale@soton.ac.uk

^b^ Departamento de Química, Facultad de Ciencias, Universidad de Burgos,09001 Burgos, Spain. E-mail: [rquesada@ubu.es](mailto:rquesada@ubu.es)

*^c^ Organic and Polymeric Materials Tokyo Institute of Technology 2-12-1 O-okayama, Tokyo 152-8552, JAPAN*

1. SYNTHESIS AND CHARACTERIZATION	S1
   1.1. Overview of compounds	S1
   1.2. Experimental procedures	S1
   1.3. Characterization data	S7
2. ANION TRANSPORT STUDIES	S46
   2.1. Preparation of vesicles	S46
   2.2. Chloride/nitrate transport assays	S46
   2.3. Initial rate of chloride efflux (k_ini_) determination	S47
   2.4. Hill plot analyses. EC_50_ determination	S68
   2.5. K_ini_ EC_50_ correlation for EC_50_ Prediction	S91
   2.6. Overview of anion transport results	S92
3. QSAR ANALYSIS	S93
   3.1. Fit all Models – 3 and 4 parameters	S93
   3.2. Descriptor definitions	S94
   3.3. Model fits	S95
   3.4. Model fit plots	S96
   3.5. R Code from linear fits	S97
   3.6. R Code from mixed effect fits	S115
1. **SYNTHESIS AND CHARACTERIZATION**
   1.1. **Overview of compounds**

Chart S1. Overview of tambjamine derivatives included in this study.

1.2. **Experimental procedures**

**General**. NMR spectra were recorded on Varian Mercury-300 MHz and Varian Unity-400 MHz spectrometers. Chemical shifts (δ) are reported in parts per million (ppm) and calibrated to the residual solvent peak in CDCl_3_ and DMSO-*d_6_*. High resolution mass spectra (HRMS) were recorded on a MicromassAutospec S-2 spectrometer using EI at 70 eV. Commercial reagents were used as provided by the supplier. 4-methoxy-1H,1'H-[2,2'-bipyrrole]-5-carbaldehyde and 4-(benzyloxy)-1H,1'H-[2,2'-bipyrrole]-5-carbaldehyde^[1]^ were prepared as described in the bibliography. Compounds **5**, **9**, **34**, **35**, **37**, **38**^[2]^ and **20**, **21**, **22**, **23**, **24**, **25**, **26**, **27**, **28**, **29**, **30**, **31**, **32**, **39**, **40, 42**^[3]^ were previously reported by us.

Compounds **1–43** were synthesized using modifications of the previously reported method.^[4]^ 4-methoxy-1H,1'H-[2,2'-bipyrrole]-5-carbaldehyde (190 mg, 1 mmol) or 4-(benzyloxy)-1H,1'H-[2,2'-bipyrrole]-5-carbaldehyde (266 mg, 1 mmol) and the corresponding amine (1.6–8 mmol, 1.6–8 mmol equivalents) were dissolved in 10 ml chloroform (or 1,2-dichloroethane; compounds **11**, **13**, **14**, **15**, **16**, **17**, **18**, **30**, **31** and **32**), 40 µL of acetic acid were added. The mixture was stirred at 60 ºC until TLC showed disappearance of the staring material. The reaction mixture was diluted with 40 mL of dichloromethane and washed with HCl 1M (3 × 25 mL). The organic fraction was dried over Na_2_SO_4_ and the solvent evaporated to yield **1**–**43** as yellow–orange solids in good to excellent yields.

**4'-methoxy-5'-((p-tolylamino)methylene)-1H,5'H-[2,2'-bipyrrol]-1'-ium chloride (1).** Yield: 100%; ^1^H NMR (300 MHz, CDCl_3_): δ = 13.86 (s, 1H), 11.17 (d, *J* = 14.5 Hz, 1H), 10.67 (s, 1H), 7.75 (d, *J* = 14.7 Hz, 1H), 7.31–7.10 (m, 5H), 6.80 (s, 1H), 6.30 (d, *J* = 3.7 Hz, 1H), 5.98 (d, *J* = 2.1 Hz, 1H), 3.96 (s, 3H, OCH_3_), 2.33 (s, 3H); ^13^C NMR (75 MHz, CDCl_3_): δ = 165.09 (C), 144.12 (C), 136.18 (C), 136.04 (C), 130.53 (2CH), 130.31 (CH), 125.34 (CH), 122.64 (C), 117.19 (2CH), 114.77 (CH), 113.29 (C), 111.39 (CH), 92.01 (CH), 58.85 (OCH_3_), 21.09 (CH_3_); HRMS (EI) m/z [M]^+^ calcd for [C_17_H_17_N_3_O] 279.1372; found: 279.1376.

**5'-(((4-ethylphenyl)amino)methylene)-4'-methoxy-1H,5'H-[2,2'-bipyrrol]-1'-ium chloride (2).** Yield: 95%; ^1^H NMR (300 MHz, CDCl_3_): δ = 13.90 (s, 1H), 11.20 (d, *J* = 14.3 Hz, 1H), 10.69 (s, 1H), 7.76 (d, *J* = 14.7 Hz, 1H), 7.41–7.10 (m, 5H), 6.81 (s, 1H), 6.32 (s, 1H), 5.99 (s, 1H), 3.97 (s, 3H, OCH_3_), 2.64 (app q, 2H), 1.22 (t, *J* = 7.6 Hz, 3H); ^13^C NMR (75 MHz, CDCl_3_): δ = 165.07 (C), 144.05 (C), 142.38 (C), 136.32 (C), 130.29 (CH), 129.31 (2CH), 125.21 (CH), 122.63 (C), 117.26 (2CH), 114.73 (CH), 113.26 (C), 111.35 (CH), 92.04 (CH), 58.84 (OCH_3_), 28.42 (CH_2_), 15.62 (CH_3_); HRMS (EI) m/z [M]^+^ calcd for [C_18_H_19_N_3_O] 293.1528; found: 293.1521.

**4'-methoxy-5'-(((4-propylphenyl)amino)methylene)-1H,5'H-[2,2'-bipyrrol]-1'-ium chloride (3).** Yield: 92%; ^1^H NMR (300 MHz, CDCl_3_): δ = 13.55 (s, 1H), 11.08 (d, *J* = 14.6 Hz, 1H), 10.61 (s, 1H), 7.62 (d, *J* = 14.7 Hz, 1H), 7.26–7.06 (m, 4H), 7.01 (d, *J* = 1.2 Hz, 1H), 6.75 (s, 1H), 6.21 (d, *J* = 3.7 Hz, 1H), 5.95 (d, *J* = 2.1 Hz, 1H), 3.86 (s, 3H, OCH_3_), 2.50 (t, *J* = 7.1 Hz, 2H), 1.65–1.49 (m, 2H), 0.89 (t, *J* = 7.3 Hz, 3H); ^13^C NMR (75 MHz, CDCl_3_): δ = 164.97 (C), 143.87 (C), 140.65 (C), 136.20 (C), 130.05 (CH), 129.75 (2CH), 124.93 (CH), 122.51 (C), 117.00 (2CH), 114.62 (CH), 113.08 (C), 111.24 (CH), 92.06 (CH), 58.78 (OCH_3_), 37.41 (CH_2_), 24.49 (CH_2_), 13.75 (CH_3_); HRMS (EI) m/z [M]^+^ calcd for [C_19_H_21_N_3_O] 307.1685; found: 307.1690.

**5'-(((4-butylphenyl)amino)methylene)-4'-methoxy-1H,5'H-[2,2'-bipyrrol]-1'-ium chloride (4).** Yield: 97%; ^1^H NMR (300 MHz, CDCl_3_): δ = 13.59 (s, 1H), 11.08 (d, *J* = 14.6 Hz, 1H), 10.62 (s, 1H), 7.64 (d, *J* = 14.7 Hz, 1H), 7.26–7.09 (m, 4H), 7.06–7.01 (m, 1H), 6.83–6.71 (m, 1H), 6.23 (d, *J* = 3.8 Hz, 1H), 5.95 (d, *J* = 2.2 Hz, 1H), 3.88 (s, 3H, OCH_3_), 2.54 (t, *J* = 7.8 Hz, 2H), 1.61–1.46 (m, 2H), 1.37–1.24 (m, 2H), 0.89 (t, *J* = 7.3 Hz, 3H); ^13^C NMR (75 MHz, CDCl_3_): δ = 164.99 (C), 143.90 (C), 140.93 (C), 136.18 (C), 130.10 (CH), 129.73 (2CH), 125.01 (CH), 122.53 (C), 117.06 (2CH), 114.65 (CH), 113.10 (C), 111.26 (CH), 92.06 (CH), 58.79 (OCH_3_), 35.09 (CH_2_), 33.57 (CH_2_), 22.33 (CH_2_), 13.98 (CH_3_); HRMS (EI) m/z [M]^+^ calcd for [C_20_H_23_N_3_O] 321.1841; found: 321.1841.

**4'-methoxy-5'-(((4-pentylphenyl)amino)methylene)-1H,5'H-[2,2'-bipyrrol]-1'-ium chloride (6).**

Yield: 100%; ^1^H NMR (300 MHz, CDCl_3_): δ = 13.58 (s, 1H), 11.08 (d, *J* = 14.6 Hz, 1H), 10.62 (s, 1H), 7.62 (d, *J* = 14.6 Hz, 1H), 7.26–7.05 (m, 4H), 7.01 (s, 1H), 6.75 (s, 1H), 6.21 (d, *J* = 1.6 Hz, 1H), 5.94 (s, 1H), 3.86 (s, 3H, OCH_3_), 2.51 (t, *J* = 7.7 Hz, 2H), 1.62–1.47 (m, 2H), 1.30–1.20 (m, 4H), 0.86 (t, *J* = 6.5 Hz, 3H); ^13^C NMR (75 MHz, CDCl_3_): δ = 164.96 (C), 143.86 (C), 140.91 (C), 136.17 (C), 130.06 (CH), 129.68 (2CH), 124.90 (CH), 122.53 (C), 117.01 (2CH), 114.59 (CH), 113.08 (C), 111.24 (CH), 92.05 (CH), 58.77 (OCH_3_), 35.33 (CH_2_), 31.43 (CH_2_), 31.07 (CH_2_), 22.52 (CH_2_), 14.06 (CH_3_); HRMS (EI) m/z [M]^+^ calcd for [C_21_H_25_N_3_O] 335.1998; found: 335.1981.

**5'-(((4-decylphenyl)amino)methylene)-4'-methoxy-1H,5'H-[2,2'-bipyrrol]-1'-ium chloride (7).** Yield: 95%; ^1^H NMR (400 MHz, CDCl_3_): δ = 13.90 (s, 1H), 11.20 (d, *J* = 14.5 Hz, 1H), 10.68 (s, 1H), 7.75 (d, *J* = 14.6 Hz, 1H), 7.32–7.16 (m, 4H), 7.12 (s, 1H), 6.81 (s, 1H), 6.31 (d, *J* = 3.6 Hz, 1H), 5.99 (d, *J* = 2.1 Hz, 1H), 3.97 (s, 3H, OCH_3_), 2.58 (t, *J* = 5.7 Hz, 2H), 1.64–1.52 (m, 4H), 1.30–1.24 (m, 12H), 0.87 (t, *J* = 6.7 Hz, 3H); ^13^C NMR (75 MHz, CDCl_3_): δ = 165.03 (C), 143.98 (C), 141.09 (C), 136.25 (C), 130.23 (CH), 129.81 (2CH), 125.14 (CH), 122.61 (C), 117.15 (2CH), 114.69 (CH), 113.21 (C), 111.32 (CH), 92.03 (CH), 58.82 (OCH_3_), 35.47 (CH_2_), 31.99 (CH_2_), 31.51 (CH_2_), 29.70 (2CH_2_), 29.57 (CH_2_), 29.42 (CH_2_), 29.33 (CH_2_), 22.78 (CH_2_), 14.23 (CH_3_); HRMS (EI) m/z [M]^+^ calcd for [C_26_H_35_N_3_O] 405.2780; found: 405.2776.

**5'-(((4-hydroxyphenyl)amino)methylene)-4'-methoxy-1H,5'H-[2,2'-bipyrrol]-1'-ium chloride (8).** Yield: 38%; ^1^H NMR (300 MHz, DMSO-*d_6_*): δ = 13.09 (s, 1H), 12.52 (d, *J* = 14.1 Hz, 1H), 11.96 (s, 1H), 9.74 (s, 1H), 8.17 (d, *J* = 13.9 Hz, 1H), 7.46 (d, *J* = 8.4 Hz, 2H), 7.19 (s, 2H, OH), 6.83 (d, *J* = 8.4 Hz, 2H), 6.56 (s, 1H), 6.31 (s, 1H), 3.98 (s, 3H, OCH_3_); ^13^C NMR (75 MHz, DMSO-*d_6_*): δ = 164.36 (C), 155.85 (C), 142.16 (C), 132.53 (CH), 130.45 (C), 124.39 (CH), 122.24 (C), 119.09 (2CH), 116.20 (2CH), 111.95 (CH), 111.89 (C), 110.81 (CH), 92.19 (CH), 58.75 (OCH_3_); HRMS (EI) m/z [M]^+^ calcd for [C_16_H_15_N_3_O_2_] 281.1164; found: 281.1166.

**5'-(((4-ethoxyphenyl)amino)methylene)-4'-methoxy-1H,5'H-[2,2'-bipyrrol]-1'-ium chloride**

**(10).** Yield: 92%; ^1^H NMR (300 MHz, CDCl_3_): δ = 13.75 (s, 1H), 11.23 (d, *J* = 14.7 Hz, 1H), 10.61 (s, 1H), 7.69 (d, *J* = 14.8 Hz, 1H), 7.31 (d, *J* = 9.0 Hz, 2H), 7.13–7.07 (m, 1H), 6.88 (d, *J* = 9.0 Hz, 2H), 6.82–6.75 (m, 1H), 6.38–6.22 (m, 1H), 5.97 (d, *J* = 2.2 Hz, 1H), 4.01 (q, *J* = 7.0 Hz, 2H), 3.95 (s, 3H, OCH_3_), 1.40 (t, *J* = 7.0 Hz, 3H); ^13^C NMR (75 MHz, CDCl_3_): δ = 164.75 (C), 157.40 (C), 143.54 (C), 131.83 (C), 130.59 (CH), 125.04 (CH), 122.71 (C), 118.73 (2CH), 115.71 (2CH), 114.40 (CH), 112.99 (C), 111.29 (CH), 91.91 (CH), 63.98 (CH_2_), 58.79 (OCH_3_), 14.88 (CH_3_); HRMS (EI) m/z [M]^+^ calcd for [C_18_H_19_N_3_O_2_] 309.1477; found: 309.1483.

**4'-methoxy-5'-(((4-(trifluoromethoxy)phenyl)amino)methylene)-1H,5'H-[2,2'-bipyrrol]-1'-ium chloride (11).** Yield: 95%; ^1^H NMR (300 MHz, CDCl_3_): δ = 13.68 (s, 1H), 11.18 (d, *J* = 14.1 Hz, 1H), 10.64 (s, 1H), 7.66 (d, *J* = 14.3 Hz, 1H), 7.36 (d, *J* = 8.8 Hz, 2H), 7.17 (d, *J* = 8.5 Hz, 2H), 7.10 (s, 1H), 6.82 (s, 1H), 6.29 (s, 1H), 5.94 (s, 1H), 3.89 (s, 3H, OCH_3_); ^13^C NMR (75 MHz, CDCl_3_): δ = 165.71 (C), 146.51 (q, *J* = 1.7 Hz, C), 145.27 (C), 137.32(C), 129.65 (CH), 125.97 (CH), 122.70 (2CH), 122.43 (C), 120.5 (q, *J* = 256.0 Hz, CF_3_) 118.30 (2CH), 115.69 (CH), 114.06 (C), 111.72 (CH), 92.34 (CH), 58.94 (OCH_3_); HRMS (EI) m/z [M]^+^ calcd for [C_17_H_14_F_3_N_3_O_2_] 349.1038; found: 349.1039.

**4'-methoxy-5'-(((4-(methylthio)phenyl)amino)methylene)-1H,5'H-[2,2'-bipyrrol]-1'-ium chloride (12).** Yield: 87%; ^1^H NMR (300 MHz, DMSO*-d_6_*): δ = 13.16 (s, 1H), 12.52 (d, *J* = 14.1 Hz, 1H), 12.06 (s, 1H), 8.27 (d, *J* = 14.2 Hz, 1H), 7.58 (d, *J* = 8.4 Hz, 2H), 7.34 (d, *J* = 8.4 Hz, 2H), 7.25 (s, 2H), 6.60 (s, 1H), 6.35 (s, 1H), 4.01 (s, 3H, OCH_3_), 2.50 (s, 3H); ^13^C NMR (75 MHz, DMSO*-d_6_*): δ = 165.21 (C), 143.58 (C), 135.94 (C), 135.40 (C), 131.90 (CH), 127.29 (2CH), 125.10 (CH), 122.15 (2C), 118.02 (2CH), 112.79 (CH), 111.14 (CH), 92.54 (CH), 58.92 (OCH_3_), 15.06 (CH_3_); HRMS (EI) m/z [M]^+^ calcd for [C_17_H_17_N_3_OS] 311.1092; found: 311.1098.

**5'-(((4-fluorophenyl)amino)methylene)-4'-methoxy-1H,5'H-[2,2'-bipyrrol]-1'-ium chloride (13).** Yield: 82%; ^1^H NMR (300 MHz, DMSO-*d_6_*): δ = 13.19 (s, 1H), 12.58 (d, *J* = 14.1 Hz, 1H), 12.05 (s, 1H), 8.25 (d, *J* = 14.0 Hz, 1H), 7.72–7.59 (m, 2H), 7.38–7.16 (m, 4H), 6.59 (s, 1H), 6.34 (s, 1H), 4.00 (s, 3H, OCH_3_); ^13^C NMR (75 MHz, DMSO-*d_6_*): δ = 165.53 (C), 159.78 (C, d, *J* = 241.5 Hz ), 143.87 (C), 135.30 (C, d, *J* = 2.3 Hz), 132.58 (CH), 125.21 (CH), 122.12 (C), 119.35 (2CH, d, *J* = 8.3 Hz), 116.60 (2CH, d, *J* = 23.1 Hz), 112.92 (CH), 111.83 (C), 111.20 (CH), 92.61 (CH), 58.99 (OCH_3_); HRMS (EI) m/z [M]+ calcd for [C_16_H_14_FN_3_O] 283.1121; found: 283.1130.

**5'-(((4-chlorophenyl)amino)methylene)-4'-methoxy-1H,5'H-[2,2'-bipyrrol]-1'-ium chloride** **(14).** Yield: 74%; ^1^H NMR (300 MHz, DMSO-*d_6_*): δ = 13.21 (s, 1H), 12.55 (d, *J* = 13.8 Hz, 1H), 12.08 (s, 1H), 8.25 (d, *J* = 13.5 Hz, 1H), 7.63 (d, *J* = 8.8 Hz, 2H), 7.48 (d, *J* = 8.8 Hz, 2H), 7.26 (s, 2H), 6.59 (s, 1H), 6.35 (s, 1H), 4.00 (s, 3H, OCH_3_); ^13^C NMR (75 MHz, DMSO-*d_6_*): δ = 165.66 (C), 144.33 (C), 137.72 (C), 131.85 (CH), 129.58 (2CH), 129.32 (C), 125.41 (CH), 122.03 (C), 118.95 (2CH), 113.18 (CH, C), 111.22 (CH), 92.66 (CH), 58.94 (OCH_3_); HRMS (EI) m/z [M]+ calcd for [C_16_H_14_ClN_3_O] 299.0825; found: 299.0828.

**5'-(((4-bromophenyl)amino)methylene)-4'-methoxy-1H,5'H-[2,2'-bipyrrol]-1'-ium chloride (15).** Yield: 95%; ^1^H NMR (300 MHz, DMSO-*d_6_*): δ = 13.06 (s, 1H), 12.37 (d, *J* = 12.1 Hz, 1H), 12.07 (s, 1H), 8.18 (d, *J* = 8.9 Hz, 1H), 7.62–7.48 (m, 4H), 7.23 (s, 2H), 6.56 (s, 1H), 6.34 (s, 1H), 3.97 (s, 3H, OCH_3_); ^13^C NMR (75 MHz, DMSO-*d_6_*): δ = 165.84 (C), 144.55 (C), 138.19 (C), 132.58 (2CH), 131.65 (CH), 125.58 (CH), 122.11 (C), 119.33 (2CH), 117.59 (C), 113.34 (CH, C), 111.39 (CH), 92.80 (CH), 59.09 (OCH_3_); HRMS (EI) m/z [M]+ calcd for [C_16_H_14_BrN_3_O] 343.0320; found: 343.0328.

**5'-(((4-iodophenyl)amino)methylene)-4'-methoxy-1H,5'H-[2,2'-bipyrrol]-1'-ium chloride (16).** Yield: 72%; ^1^H NMR (300 MHz, DMSO*-d_6_*): δ = 13.19 (s, 1H), 12.47 (d, *J* = 14.1 Hz, 1H), 12.08 (s, 1H), 8.24 (d, *J* = 13.8 Hz, 1H), 7.76 (d, *J* = 8.7 Hz, 2H), 7.43 (d, *J* = 8.7 Hz, 2H), 7.26 (s, 2H), 6.59 (s, 1H), 6.35-6.33 (m, 1H), 4.00 (s, 3H, OCH_3_); ^13^C NMR (75 MHz, DMSO*-d_6_*): δ = 165.70 (C), 144.42 (C), 138.56 (C), 138.28 (2CH), 131.52 (CH), 125.49 (CH), 122.04 (C), 119.43 (2CH), 113.25 (CH, C), 111.27 (CH), 92.69 (CH), 89.79 (C), 58.97 (OCH_3_); HRMS (EI) m/z [M]+ calcd for [C_16_H_14_IN_3_O] 391.0182; found: 391.0179.

**5'-(((4-cyanophenyl)amino)methylene)-4'-methoxy-1H,5'H-[2,2'-bipyrrol]-1'-ium chloride (17).** Yield: 100%; ^1^H NMR (300 MHz, CDCl_3_): δ = 13.99 (s, 1H), 11.34 (d, *J* = 13.7 Hz, 1H), 10.72 (s, 1H), 7.71 (d, *J* = 13.9 Hz, 1H), 7.65 (d, *J* = 8.7 Hz, 2H), 7.46 (d, *J* = 8.8 Hz, 2H), 7.24–7.18 (m, 1H), 6.96–6.89 (m, 1H), 6.43–6.32 (m, 1H), 6.03 (d, *J* = 2.0 Hz, 1H), 4.02 (s, 3H; OCH_3_); ^1^H NMR (300 MHz, DMSO*-d_6_*): δ = 13.28 (s, 1H), 12.56 (d, *J* = 13.7 Hz, 1H), 12.19 (s, 1H), 8.32 (d, *J* = 0.6 Hz, 1H), 7.89 (d, *J* = 8.5 Hz, 2H), 7.77 (d, *J* = 8.4 Hz, 2H), 7.34 (s, 2H), 6.64 (s, 1H), 6.39 (s, 1H), 4.03 (s, 3H; OCH_3_); ^13^C NMR (75 MHz, DMSO*-d_6_*): δ = 166.52 (C), 145.92 (C), 142.69 (C), 133.97 (2CH), 130.70 (CH), 126.39 (CH), 121.86 (C), 118.77 (C), 117.58 (2CH), 114.47 (C), 114.20 (CH), 111.65 (CH), 106.61 (C), 93.09 (CH), 59.13 (OCH_3_); HRMS (EI) m/z [M]^+^ calcd for [C_17_H_14_N_4_O] 290.1168; found: 290.1165.

**4'-methoxy-5'-(((4-(methylsulfonyl)phenyl)amino)methylene)-1H,5'H-[2,2'-bipyrrol]-1'-ium chloride (18).** Yield: 66%; ^1^H NMR (300 MHz, DMSO-*d_6_*): δ = 13.32 (s, 1H), 12.60 (d, *J* = 13.7 Hz, 1H), 12.20 (s, 1H), 8.34 (d, *J* = 13.6 Hz, 1H), 7.95 (d, *J* = 8.6 Hz, 2H), 7.82 (d, *J* = 8.6 Hz, 2H), 7.33 (s, 2H), 6.65 (s, 1H), 6.39 (s, 1H), 4.04 (s, 3H, OCH_3_), 3.23 (s, 3H); ^13^C NMR (75 MHz, DMSO-*d_6_*): δ = 166.49 (C), 145.80 (C), 143.04 (C), 136.37 (C), 130.89 (CH), 128.95 (2CH), 126.31 (CH), 121.89 (C), 117.35 (2CH), 114.34 (CH), 114.13 (C), 111.64 (CH), 93.08 (CH), 59.14 (OCH_3_), 43.70 (CH_3_); HRMS (EI) m/z [M]^+^ calcd for [C_17_H_17_N_3_O_3_S] 343.0991; found: 343.0986.

**1-(4'-methoxy-1H,5'H-[2,2'-bipyrrol]-5'-ylidene)-N-(pyridin-2-ylmethyl)methanamine (19).** Yield: 19%; ^1^H NMR (300 MHz, CDCl_3_): δ = 13.70 (s, 1H), 10.67 (s, 1H), 9.83 (s, 1H), 8.57 (d, *J* = 4.8 Hz, 1H), 7.73 (td, *J* = 7.9, 1.5 Hz, 1H), 7.58–7.45 (m, 2H), 7.30–7.20 (m, 1H), 7.06 (s, 1H), 6.80–6.71 (m, 1H), 6.31–6.24 (m, 1H), 5.95 (s, 1H), 4.73 (s, 2H), 3.92 (s, 3H, OCH_3_). ^13^C NMR (75 MHz, CDCl_3_): δ = 164.63 (C), 155.37 (C), 149.91 (CH), 143.34 (C), 140.75 (CH), 137.61 (CH), 124.63 (CH), 123.41 (CH), 122.71 (C), 122.51 (CH), 113.86 (CH), 111.79 (C), 111.04 (CH), 91.46 (CH), 58.66 (OCH_3_), 55.19 (CH_2_). HRMS (EI) m/z [M]^+^ calcd for [C_16_H_16_N_4_O_4_] 280.1324; found: 280.1325.

**4'-methoxy-5'-(((3-methoxypropyl)amino)methylene)-1H,5'H-[2,2'-bipyrrol]-1'-ium chloride (33).** Yield: 88%; ^1^H NMR (300 MHz, CDCl_3_): δ = 13.63 (s, 1H), 10.61 (s, 1H), 9.40 (s, 1H), 7.35 (d, *J* = 14.4 Hz, 1H), 7.05 (td, *J* = 2.7, 1.4 Hz, 1H), 6.76–6.71 (m, 1H), 6.31–6.25 (m, 1H), 5.94 (d, *J* = 1.9 Hz, 1H), 3.92 (s, 3H, OCH_3_), 3.61 (app q, 2H), 3.50 (t, *J* = 5.7 Hz, 2H), 3.35 (s, 3H, OCH_3_), 1.99 (app quin, 2H). ^13^C NMR (75 MHz, CDCl_3_): δ = 164.00 (C), 142.51 (C), 140.79 (CH), 124.23 (CH), 122.77 (C), 113.32 (CH), 110.97 (C), 110.87 (CH), 91.24 (CH), 68.50 (CH_2_), 58.83 (OCH_3_), 58.60 (OCH_3_), 47.77 (CH_2_), 30.13 (CH_2_). HRMS (EI) m/z [M]^+^ calcd for [C_14_H_19_N_3_O_2_] 261.1417; found: 261.1475.

**4'-(benzyloxy)-5'-(((4-pentylphenyl)amino)methylene)-1H,5'H-[2,2'-bipyrrol]-1'-ium chloride (36).** Yield: 95%; ^1^H NMR (300 MHz, CDCl_3_): δ = 13.90 (s, 1H), 11.26 (d, *J* = 14.7 Hz, 1H), 10.70 (s, 1H), 7.76 (d, *J* = 14.4 Hz, 1H), 7.53–7.35 (m, 5H), 7.33–7.12 (m, 4H), 7.09 (s, 1H), 6.79 (s, 1H), 6.29 (s, 1H), 6.06 (s, 1H), 5.19 (s, 2H), 2.57 (t, *J* = 7.6 Hz, 2H), 1.58 (app quin, 2H), 1.36–1.24 (m, 4H), 0.89 (t, *J* = 6.6 Hz, 3H). ^13^C NMR (75 MHz, CDCl_3_): δ = 163.90 (C), 144.04 (C), 141.26 (C), 136.34 (C), 134.91 (C), 130.64 (CH), 129.87 (2CH), 129.08 (CH), 129.00 (2CH), 128.22 (2CH), 125.29 (CH), 122.67 (C), 117.40 (2CH), 114.75 (CH), 113.57 (C), 111.35 (CH), 92.98 (CH), 73.84 (CH_2_), 35.47 (CH_2_), 31.50 (CH_2_), 31.17 (CH_2_), 22.62 (CH_2_), 14.14 (CH_3_). HRMS (EI) m/z [M]^+^ calcd for [C_27_H_29_N_3_O] 411.2311; found: 411.2310.

**4'-(benzyloxy)-5'-((nonylamino)methylene)-1H,5'H-[2,2'-bipyrrol]-1'-ium chloride (41).** Yield: 74%; ^1^H NMR (300 MHz, CDCl_3_): δ = 13.44 (s, 1H), 10.48 (s, 1H), 9.37 (d, *J* = 14.9 Hz, 1H), 7.37–7.08 (m, 6H), 6.85 (s, 1H), 6.60 (s, 1H), 6.14–6.07 (m, 1H), 5.89 (d, *J* = 2.0 Hz, 1H), 4.96 (s, 2H), 3.28 (app q, 2H), 1.58 (app quin, 2H), 1.28–1.03 (m, 12H), 0.72 (t, *J* = 6.6 Hz, 3H). ^13^C NMR (75 MHz, CDCl_3_): δ = 162.55 (C), 142.11 (C), 140.55 (CH), 135.05 (C), 128.85 (CH), 128.82 (2CH), 128.02 (2CH), 123.96 (CH), 122.72 (C), 113.11 (CH), 111.01 (C), 110.74 (CH), 92.10 (CH), 73.43 (CH_2_), 51.04 (CH_2_), 31.82 (CH_2_), 30.31 (CH_2_), 29.40 (CH_2_), 29.23 (CH_2_), 29.12 (CH_2_), 26.53 (CH_2_), 22.66 (CH_2_), 14.13 (CH_3_). HRMS (EI) m/z [M]^+^ calcd for [C_25_H_33_N_3_O] 391.2624; found: 391.2530.

**4'-(benzyloxy)-5'-((undecylamino)methylene)-1H,5'H-[2,2'-bipyrrol]-1'-ium chloride (43).** Yield: 59%; ^1^H NMR (300 MHz, CDCl_3_): δ = 13.61 (s, 1H), 10.62 (s, 1H), 9.51 (d, *J* = 14.7 Hz, 1H), 7.43–7.31 (m, 6H), 7.01 (s, 1H), 6.72 (s, 1H), 6.29–6.21 (m, 1H), 6.01 (d, *J* = 1.9 Hz, 1H), 5.11 (s, 2H), 3.43 (app q, 2H), 1.72 (app quin, 2H), 1.41–1.17 (m, 18H), 0.86 (t, *J* = 6.6 Hz, 3H). ^13^C NMR (75 MHz, CDCl_3_): δ = 162.60 (C), 142.21 (C), 140.59 (CH), 135.09 (C), 128.93 (CH), 128.89 (2CH), 128.07 (2CH), 124.10 (CH), 122.76 (C), 113.18 (CH), 111.07 (C), 110.79 (CH), 92.11 (CH), 73.49 (CH_2_), 51.12 (CH_2_), 31.98 (CH_2_), 30.38 (CH_2_), 29.68 (2CH_2_), 29.64 (CH_2_), 29.51 (CH_2_), 29.41 (CH_2_), 29.19 (CH_2_), 26.60 (CH_2_), 22.76 (CH_2_), 14.20 (CH_3_). HRMS (EI) m/z [M]^+^ calcd for [C_28_H_39_N_3_O] 433.3093; found: 433.3112.

1.3. **Characterization data**

Figure S1. ^1^H NMR (CDCl_3_) of compound **1**. HCl.

Figure S2. ^13^C NMR (CDCl_3_) of compound **1**. HCl.

Figure S3. DEPT ^13^C NMR (CDCl_3_) of compound **1**. HCl.

Figure S4. HRMS (EI) of compound **1**.

 Figure S5. ^1^H NMR (CDCl_3_) of compound **2**. HCl.

Figure S6. ^13^C NMR (CDCl_3_) of compound **2**. HCl.

Figure S7. DEPT ^13^C NMR (CDCl_3_) of compound **2**. HCl.

Figure S8. HRMS (EI) of compound **2**.

Figure S9. ^1^H NMR (CDCl_3_) of compound **3**. HCl.

 Figure S10. ^13^C NMR (CDCl_3_) of compound **3**. HCl.

 Figure S11. DEPT ^13^C NMR (CDCl_3_) of compound **3**. HCl.

Figure S12. HRMS (EI) of compound **3**.

Figure S13. ^1^H NMR (CDCl_3_) of compound **4**. HCl.

 Figure S14. ^13^C NMR (CDCl_3_) of compound **4**. HCl.

 Figure S15. DEPT ^13^C NMR (CDCl_3_) of compound **4**. HCl.

Figure S16. HRMS (EI) of compound **4**.

Figure S17. ^1^H NMR (CDCl_3_) of compound **6**. HCl.

Figure S18. ^13^C NMR (CDCl_3_) of compound **6**. HCl.

Figure S19. DEPT ^13^C NMR (CDCl_3_) of compound **6**. HCl.

Figure S20. HRMS (EI) of compound **6**.

Figure S21. ^1^H NMR (CDCl_3_) of compound **7**. HCl.

 Figure S22. ^13^C NMR (CDCl_3_) of compound **7**. HCl.

 Figure S23. DEPT ^13^C NMR (CDCl_3_) of compound **7**. HCl.

Figure S24. HRMS (EI) of compound **7**.

 Figure S25. ^1^H NMR (DMSO-*d_6_*) of compound **8**. HCl.

Figure S26. ^13^C NMR (DMSO-*d_6_*) of compound **8**. HCl.

Figure S27. DEPT ^13^C NMR (DMSO-*d_6_*) of compound **8**. HCl.

Figure S28. HRMS (EI) of compound **8**.

Figure S29. ^1^H NMR (CDCl_3_) of compound **10**. HCl.

Figure S30. ^13^C NMR (CDCl_3_) of compound **10**. HCl.

Figure S31. HRMS (EI) of compound **10**.

Figure S32. ^1^H NMR (CDCl_3_) of compound **11**. HCl.

Figure S33. ^13^C NMR (CDCl_3_) of compound **11**. HCl.

Figure S34. DEPT ^13^C NMR (CDCl_3_) of compound **11**. HCl.

Figure S35. HRMS (EI) of compound **11**.

Figure S36. ^1^H NMR (DMSO-*d_6_*) of compound **12**. HCl.

 Figure S37. ^13^C NMR (DMSO-*d_6_*) of compound **12**. HCl.

Figure S38. DEPT ^13^C NMR (DMSO-*d_6_*) of compound **12**. HCl.

Figure S39. HRMS (EI) of compound **12**.

Figure S40. ^1^H NMR (DMSO-*d_6_*) of compound **13**. HCl.

Figure S41. ^13^C NMR (DMSO-*d_6_*) of compound **13**. HCl.

Figure S42. DEPT ^13^C NMR (DMSO-*d_6_*) of compound **13**. HCl.

Figure S43. HRMS (EI) of compound **13**.

Figure S44. ^1^H NMR (DMSO-*d_6_*) of compound **14**. HCl.

Figure S43. ^13^C NMR (DMSO-*d_6_*) of compound **14**. HCl.

Figure S45. DEPT ^13^C NMR (DMSO-*d_6_*) of compound **14**. HCl.

Figure S46. HRMS (EI) of compound **14**.

Figure S47. ^1^H NMR (DMSO-*d_6_*) of compound **15**. HCl.

Figure S48. ^13^C NMR (DMSO-*d_6_*) of compound **15**. HCl.

Figure S49. HRMS (EI) of compound **15**.

Figure S50. ^1^H NMR (DMSO-*d_6_*) of compound **16**. HCl.

Figure S51. ^13^C NMR (DMSO-*d_6_*) of compound **16**. HCl.

Figure S52. HRMS (EI) of compound **16**.

 Figure S53. ^1^H NMR (CDCl_3_) of compound **17**. HCl.

 Figure S54. ^1^H NMR (DMSO-*d_6_*) of compound **17**. HCl.

 Figure S55. ^13^C NMR (DMSO-*d_6_*) of compound **17**. HCl.

Figure S56. HRMS (EI) of compound **17**.

Figure S57. ^1^H NMR (DMSO-*d_6_*) of compound **18**. HCl.

Figure S58. ^13^C NMR (DMSO-*d_6_*) of compound **18**. HCl.

Figure S59. HRMS (EI) of compound **18**.

Figure S60. ^1^H NMR (CDCl_3_) of compound **19**. HCl.

Figure S61. ^13^C NMR (CDCl_3_) of compound **19**. HCl.

Figure S62. DEPT ^13^C NMR (CDCl_3_) of compound **19**. HCl.

Figure S63. HRMS (EI) of compound **19**.

Figure S64. ^1^H NMR (CDCl_3_) of compound **33**. HCl.

Figure S65. ^13^C NMR (CDCl_3_) of compound **33**. HCl.

Figure S66. HRMS (EI) of compound **33**.

Figure S67. ^1^H NMR (CDCl_3_) of compound **36**. HCl.

Figure S68. ^13^C NMR (CDCl_3_) of compound **36**. HCl.

Figure S69. DEPT ^13^C NMR (CDCl_3_) of compound **36**. HCl.

Figure S70. HRMS (EI) of compound **36**.

Figure S71. ^1^H NMR (CDCl_3_) of compound **41**. HCl.

Figure S72. ^13^C NMR (CDCl_3_) of compound **41**. HCl.

Figure S73. DEPT ^13^C NMR (CDCl_3_) of compound **41**. HCl.

Figure S74. HRMS (EI) of compound **41**.

Figure S75. ^1^H NMR (CDCl_3_) of compound **43**. HCl.

Figure S76. ^13^ CNMR (CDCl_3_) of compound **43**. HCl.

Figure S77. DEPT ^13^ CNMR (CDCl_3_) of compound **43**. HCl.

Figure S78. HRMS (EI) of compound **43**.

2. **ANION TRANSPORT STUDIES**
   2.1. **Preparation of Vesicles**

Using a rotary evaporator a chloroform solution of 1-palmitoyl-2-oleoyl-sn-glycero-3- phosphocholine (POPC) was evaporated in vacuo and the lipid film obtained was dried under high vacuum for at least 4 hours. POPC (1-palmitoyl-2-oleoyl-sn-glycero-3- phosphocholine) was supplied by Sigma-Aldrich or Genzyme and it was stored at –20°C as a solution in chloroform (500 mg POPC in 20 mL chloroform or 1 g POPC in 35 mL chloroform). The lipid film was rehydrated by careful vortexing with a sodium chloride solution (489 mM NaCl and 5 mM phosphate buffer, pH 7.2, ionic strength 500 mM). The lipid suspension was subjected to nine freeze-thaw cycles, where the suspension was alternatively allowed to freeze in a liquid nitrogen bath, followed by thawing in a mild water bath. Finally twenty nine extrusions were carried out through a 200 nm polycarbonate Nucleopore membrane using a LiposoFast Basic extruder (Aves tin, Inc.). The resulting unilamellar vesicles were dialyzed against NaNO_3_ solution (489 mM NaNO_3_ and 5 mM phosphate buffer, pH 7.2, ionic strength 500 mM) to remove the unencapsulated chloride.

2.2. **Chloride/nitrate transport assays**

Unilamellar POPC vesicles containing NaCl (prepared as described above) were suspended in a 489 mM NaNO_3_ solution buffered to pH 7.2 with sodium phosphate salts (5 mM buffer). The final lipid concentration per sample was 0.5 mM and the total volume 5 mL. A DMSO solution of the carrier molecule, typically 5 or 10 µL to avoid the influence of the solvent molecule in the assay, was added and the chloride release out of vesicles was monitored using a chloride selective electrode for 5 minutes. Then, the vesicles were lysed by adding 20 μl of polyoxyethylene(8)lauryl ether (0.232 mM in 7:1 water:DMSO v/v) or Triton-X (10% dispersion in water) and a total chloride reading was taken at t = 6 min. The initial value was set at 0 % chloride efflux and the final one was set as 100 % chloride efflux. All other data were calibrated to these points.

2.3. **Initial rate of chloride efflux (kini) determination**

The initial rate of chloride release (k_ini_) was calculated from chloride/nitrate transport experiments as described above. The chloride release by 0.05% tambjamine was monitored over time. The chloride efflux was fitted with the following asymptotic function using Origin 8.1, where *y* is the chloride efflux (%) and *x* is the time (s):

$$y=a-b\cdot c^{x}$$

The initial rate of chloride release (k_ini_) is calculated as k_ini_ = -b·ln(c) and is expressed in % s^-1^. Figures S79-S121 show all of the obtained transport data for 0.05% tambjamine to lipid and the corresponding asymptotic fit. An overview of the obtained initial rates of chloride release (k_ini_) can be found in Table S1 in S91.

 Figure S79: Overview of the initial rate of chloride release (k_ini_) for compound **1**. For experimental details, see main text.

 Figure S80: Overview of the initial rate of chloride release (k_ini_) for compound **2**. For experimental details, see main text.

Figure S81: Overview of the initial rate of chloride release (k_ini_) for compound **3**. For experimental details, see main text.

Figure S82: Overview of the initial rate of chloride release (k_ini_) for compound **4**. For experimental details, see main text.

 Figure S83: Overview of the initial rate of chloride release (k_ini_) for compound **5**. For experimental details, see main text.

Figure S84: Overview of the initial rate of chloride release (k_ini_) for compound **6**. For experimental details, see main text.

Figure S85: Overview of the initial rate of chloride release (k_ini_) for compound **7**. For experimental details, see main text.

Figure S86: Overview of the initial rate of chloride release (k_ini_) for compound **8**. For experimental details, see main text.

Figure S87: Overview of the initial rate of chloride release (k_ini_) for compound **9**. For experimental details, see main text.

Figure S88: Overview of the initial rate of chloride release (k_ini_) for compound **10**. For experimental details, see main text.

Figure S89: Overview of the initial rate of chloride release (k_ini_) for compound **11**. For experimental details, see main text.

Figure S90: Overview of the initial rate of chloride release (k_ini_) for compound **12**. For experimental details, see main text.

Figure S91: Overview of the initial rate of chloride release (k_ini_) for compound **13**. For experimental details, see main text.

Figure S92: Overview of the initial rate of chloride release (k_ini_) for compound **14**. For experimental details, see main text.

Figure S93: Overview of the initial rate of chloride release (k_ini_) for compound **15**. For experimental details, see main text.

Figure S94: Overview of the initial rate of chloride release (k_ini_) for compound **16**. For experimental details, see main text.

Figure S95: Overview of the initial rate of chloride release (k_ini_) for compound **17**. For experimental details, see main text.

Figure S96: Overview of the initial rate of chloride release (k_ini_) for compound **18**. For experimental details, see main text.

Figure S97: Overview of the initial rate of chloride release (k_ini_) for compound **19**. For experimental details, see main text.

Figure S98: Overview of the initial rate of chloride release (k_ini_) for compound **20**. For experimental details, see main text.

Figure S99: Overview of the initial rate of chloride release (k_ini_) for compound **21**. For experimental details, see main text.

Figure S100: Overview of the initial rate of chloride release (k_ini_) for compound **22**. For experimental details, see main text.

Figure S101. Overview of the initial rate of chloride release (k_ini_) for compound **23**. For experimental details, see main text.

Figure S102. Overview of the initial rate of chloride release (k_ini_) for compound **24**. For experimental details, see main text.

Figure S103. Overview of the initial rate of chloride release (k_ini_) for compound **25**. For experimental details, see main text.

Figure S104. Overview of the initial rate of chloride release (k_ini_) for compound **26**. For experimental details, see main text.

Figure S105. Overview of the initial rate of chloride release (k_ini_) for compound **27**. For experimental details, see main text.

Figure S106. Overview of the initial rate of chloride release (k_ini_) for compound **28**. For experimental details, see main text.

Figure S107. Overview of the initial rate of chloride release (k_ini_) for compound **29**. For experimental details, see main text.

Figure S108. Overview of the initial rate of chloride release (k_ini_) for compound **30**. For experimental details, see main text.

Figure S109. Overview of the initial rate of chloride release (k_ini_) for compound **31**. For experimental details, see main text.

Figure S110. Overview of the initial rate of chloride release (k_ini_) for compound **32**. For experimental details, see main text.

Figure S111. Overview of the initial rate of chloride release (k_ini_) for compound **33**. For experimental details, see main text.

Figure S112. Overview of the initial rate of chloride release (k_ini_) for compound **34**. For experimental details, see main text.

Figure S113. Overview of the initial rate of chloride release (k_ini_) for compound **35**. For experimental details, see main text.

Figure S114. Overview of the initial rate of chloride release (k_ini_) for compound **36**. For experimental details, see main text.

Figure S115. Overview of the initial rate of chloride release (k_ini_) for compound **37**. For experimental details, see main text.

Figure S116. Overview of the initial rate of chloride release (k_ini_) for compound **38**. For experimental details, see main text.

Figure S117. Overview of the initial rate of chloride release (k_ini_) for compound **39**. For experimental details, see main text.

Figure S118. Overview of the initial rate of chloride release (k_ini_) for compound **40**. For experimental details, see main text.

Figure S119. Overview of the initial rate of chloride release (k_ini_) for compound **41**. For experimental details, see main text.

Figure S120. Overview of the initial rate of chloride release (k_ini_) for compound **42**. For experimental details, see main text.

Figure S121. Overview of the initial rate of chloride release (k_ini_) for compound **43**. For experimental details, see main text.

2.4. **Hill plot analyses. EC_50_ determination**

Chloride/nitrate transport assays were carried out using various concentrations of carriers as described. The chloride efflux (%) at 290 s was plotted as a function of the carrier concentration and the obtained results fitted with the Hill equation using Origin 8.1:

$$y=V_{máx}\cdot\frac{x^{n}}{k^{n}+x^{n}}=100\%\cdot\frac{x^{n}}{\left(EC_{50}\right)^{n}+x^{n}}$$

where *x* is the anion carrier concentration, *V_max_* is the maximum chloride efflux (100%), *y* is the chloride efflux at 290 s (%), *n* is the Hill coefficient and *k* is the anion carrier concentration needed to achieve *V_max_/2 (*when *V_max_* is fixed to 100%, *k* equals *EC_50_);* *k* and *n* are the parameters to be fitted. EC_50_, defined as the anion carrier concentration (molar % carrier to lipid) needed to induce 50% release of the total of the chloride encapsulated in the time scale of our experiments, can be obtained directly from the graphs. Figures S122-S164 show all of the obtained transport data. An overview of the EC_50_ values can be found in Table S1 in S91.

Figure S122. Overview of the Hill plots for compound **1**. For experimental details, see main text.

Figure S123. Overview of the Hill plots for compound **2**. For experimental details, see main text.

Figure S124. Overview of the Hill plots for compound **3**. For experimental details, see main text.

Figure S125. Overview of the Hill plots for compound **4**. For experimental details, see main text.

Figure S126. Overview of the Hill plots for compound **5**. For experimental details, see main text.

Figure S127. Overview of the Hill plots for compound **6**. For experimental details, see main text.

Figure S128. Overview of the Hill plots for compound **7**. For experimental details, see main text.

Figure S129. Overview of the Hill plots for compound **8**. For experimental details, see main text.

Figure S130. Overview of the Hill plots for compound **9**. For experimental details, see main text.

Figure S131. Overview of the Hill plots for compound **10**. For experimental details, see main text.

Figure S132. Overview of the Hill plots for compound **11**. For experimental details, see main text.

Figure S133. Overview of the Hill plots for compound **12**. For experimental details, see main text.

Figure S134. Overview of the Hill plots for compound **13**. For experimental details, see main text.

Figure S135. Overview of the Hill plots for compound **14**. For experimental details, see main text.

Figure S136. Overview of the Hill plots for compound **15**. For experimental details, see main text.

Figure S137. Overview of the Hill plots for compound **16**. For experimental details, see main text.

Figure S138. Overview of the Hill plots for compound **17**. For experimental details, see main text.

Figure S139. Overview of the Hill plots for compound **18**. For experimental details, see main text.

Figure S140. Overview of the Hill plots for compound **19**. For experimental details, see main text.

Figure S141. Overview of the Hill plots for compound **20**. For experimental details, see main text.

Figure S142. Overview of the Hill plots for compound **21**. For experimental details, see main text.

Figure S143. Overview of the Hill plots for compound **22**. For experimental details, see main text.

Figure S144. Overview of the Hill plots for compound **23**. For experimental details, see main text.

Figure S145. Overview of the Hill plots for compound **24**. For experimental details, see main text.

Figure S146. Overview of the Hill plots for compound **25**. For experimental details, see main text.

Figure S147. Overview of the Hill plots for compound **26**. For experimental details, see main text.

Figure S148. Overview of the Hill plots for compound **27**. For experimental details, see main text.

Figure S149. Overview of the Hill plots for compound **28**. For experimental details, see main text.

Figure S150. Overview of the Hill plots for compound **29**. For experimental details, see main text.

Figure S151. Overview of the Hill plots for compound **30**. For experimental details, see main text.

Figure S152. Overview of the Hill plots for compound **31**. For experimental details, see main text.

Figure S153. Overview of the Hill plots for compound **32**. For experimental details, see main text.

Figure S154. Overview of the Hill plots for compound **33**. For experimental details, see main text.

Figure S155. Overview of the Hill plots for compound **34**. For experimental details, see main text.

Figure S156. Overview of the Hill plots for compound **35**. For experimental details, see main text.

Figure S157. Overview of the Hill plots for compound **36**. For experimental details, see main text.

Figure S158. Overview of the Hill plots for compound **37**. For experimental details, see main text.

Figure S159. Overview of the Hill plots for compound **38**. For experimental details, see main text.

Figure S160. Overview of the Hill plots for compound **39**. For experimental details, see main text.

Figure S161. Overview of the Hill plots for compound **40**. For experimental details, see main text.

Figure S162. Overview of the Hill plots for compound **41**. For experimental details, see main text.

Figure S163. Overview of the Hill plots for compound **42**. For experimental details, see main text.

Figure S164. Overview of the Hill plots for compound **43**. For experimental details, see main text.

2.5. **K_ini_ EC_50_ correlation for EC_50_ Prediction**

Figure S165 –Correlation Plot for Log(1/k_ini_) and Log(1/EC_50_) excluding compound **43**.

Used in the prediction of EC_50_ for compound **43**

2.6. **Overview of anion transport results**

| **Compound** | **EC_50_ (mol%)** | **Error**  **(EC_50_)** | **n** | **Error**  **(n)** | **k_ini_ (% s^-1^)** | **Error**  **(k_ini_)** |
| --- | --- | --- | --- | --- | --- | --- |
| 1 | 0.0072 | 0.0015 | 1.19 | 0.16 | 0.95 | 0.11 |
| 2 | 0.0061 | 0.0018 | 1.23 | 0.20 | 1.41 | 0.46 |
| 3 | 0.0070 | 0.0020 | 1.25 | 0.12 | 1.24 | 0.27 |
| 4 | 0.0078 | 0.0011 | 1.19 | 0.17 | 1.17 | 0.15 |
| 5 | 0.0104 | 0.0022 | 1.30 | 0.01 | 1.02 | 0.25 |
| 6 | 0.0095 | 0.0017 | 1.25 | 0.10 | 1.13 | 0.16 |
| 7 | 0.2884 | 0.0402 | 0.96 | 0.13 | 0.02 | 0.01 |
| 8 | 0.0688 | 0.0204 | 1.42 | 0.04 | 0.23 | 0.07 |
| 9 | 0.0197 | 0.0049 | 1.29 | 0.15 | 0.64 | 0.09 |
| 10 | 0.0136 | 0.0017 | 1.28 | 0.09 | 0.79 | 0.15 |
| 11 | 0.0231 | 0.0014 | 1.31 | 0.06 | 0.47 | 0.11 |
| 12 | 0.0262 | 0.0075 | 1.29 | 0.16 | 0.49 | 0.15 |
| 13 | 0.0208 | 0.0018 | 1.18 | 0.36 | 0.47 | 0.09 |
| 14 | 0.0155 | 0.0025 | 1.27 | 0.12 | 0.66 | 0.22 |
| 15 | 0.0236 | 0.0018 | 1.37 | 0.12 | 0.44 | 0.05 |
| 16 | 0.0221 | 0.0025 | 1.29 | 0.08 | 0.51 | 0.04 |
| 17 | 0.0167 | 0.0023 | 1.48 | 0.10 | 0.83 | 0.16 |
| 18 | 0.0494 | 0.0035 | 1.59 | 0.20 | 0.31 | 0.02 |
| 19 | 0.1965 | 0.0144 | 0.85 | 0.05 | 0.09 | 0.01 |
| 20 | 0.3459 | 0.0659 | 1.30 | 0.27 | 0.04 | 0.01 |
| 21 | 0.0921 | 0.0144 | 1.08 | 0.16 | 0.21 | 0.01 |
| 22 | 0.0274 | 0.0023 | 1.04 | 0.10 | 0.52 | 0.02 |
| 23 | 0.0116 | 0.0009 | 0.86 | 0.08 | 0.74 | 0.05 |
| 24 | 0.0065 | 0.0004 | 1.18 | 0.09 | 1.46 | 0.09 |
| 25 | 0.0050 | 0.0003 | 1.19 | 0.09 | 1.50 | 0.17 |
| 26 | 0.0045 | 0.0002 | 1.51 | 0.13 | 1.52 | 0.12 |
| 27 | 0.0031 | 0.0003 | 1.07 | 0.14 | 2.63 | 0.11 |
| 28 | 0.0038 | 0.0002 | 1.10 | 0.16 | 1.63 | 0.25 |
| 29 | 0.0053 | 0.0003 | 1.33 | 0.12 | 1.54 | 0.27 |
| 30 | 0.0073 | 0.0007 | 1.15 | 0.12 | 1.09 | 0.12 |
| 31 | 0.0113 | 0.0002 | 1.20 | 0.02 | 0.94 | 0.10 |
| 32 | 0.0067 | 0.0002 | 1.05 | 0.03 | 1.01 | 0.06 |
| 33 | 0.0977 | 0.0125 | 0.96 | 0.15 | 0.22 | 0.04 |
| 34 | 0.0157 | 0.0022 | 1.32 | 0.28 | 0.74 | 0.01 |
| 35 | 0.0116 | 0.0005 | 1.20 | 0.09 | 0.71 | 0.03 |
| 36 | 0.0123 | 0.0031 | 0.86 | 0.20 | 0.32 | 0.08 |
| 37 | 0.0133 | 0.0007 | 1.45 | 0.10 | 0.60 | 0.03 |
| 38 | 0.0088 | 0.0007 | 1.43 | 0.19 | 1.69 | 0.09 |
| 39 | 0.0196 | 0.0005 | 1.14 | 0.04 | 0.60 | 0.04 |
| 40 | 0.0097 | 0.0005 | 1.74 | 0.11 | 1.16 | 0.11 |
| 41 | 0.0052 | 0.0004 | 1.15 | 0.12 | 1.04 | 0.01 |
| 42 | 0.0204 | 0.0017 | 0.93 | 0.12 | 0.42 | 0.03 |
| 43 | 0.0616^a^ | 0.0241 | -^a^ | - | 0.19 | 0.03 |

Table S1. Overview of average of the obtained *EC_50_*, *n* and *k_ini_* values. ^a^ Large error in Hill plot due to solubility issues. EC_50_ calculated from correlation with k_ini_ (see Figure S165).

3. **QSAR ANALYSIS**
   3.1. **Fit all Models – 3 and 4 parameters**

| No.  Des. | Descriptors | | | | | R^2^ | | |
| --- | --- | --- | --- | --- | --- | --- | --- | --- |
| 3 | | ALOGPs | ALOGPs-sq | Mv | – | | 0.7901 |  |
| 3 | | ALOGPs | ALOGPs-sq | J3D | – | | 0.7892 |  |
| 3 | | ALOGPs | ALOGPs-sq | Mp | – | | 0.7836 |  |
| 3 | | ALOGPs | ALOGPs-sq | nH | – | | 0.7822 |  |
| 3 | | ALOGPs | ALOGPs-sq | AMW | – | | 0.7768 |  |
| 3 | | ALOGPs | ALOGPs-sq | J | – | | 0.7680 |  |
| 3 | | ALOGPs | ALOGPs-sq | E3u | – | | 0.7672 |  |
| 3 | | ALOGPs | ALOGPs-sq | ARR | – | | 0.7654 |  |
| 3 | | ALOGPs | ALOGPs-sq | Density (g/cm^3^) | – | | 0.7615 |  |
| 3 | | ALOGPs | ALOGPs-sq | Surface tension (dyne/cm) | – | | 0.7571 |  |
| 3 | | ALOGPs | ALOGPs-sq | Lop | – | | 0.7560 |  |
| 3 | | ALOGPs | ALOGPs-sq | Du | – | | 0.7558 |  |
| 3 | | ALOGPs | ALOGPs-sq | E3e | – | | 0.7554 |  |
| 3 | | ALOGPs | ALOGPs-sq | Ui | – | | 0.7533 |  |
| 3 | | ALOGPs | ALOGPs-sq | LogD duodenum (pH 4.6) | – | | 0.7516 |  |
| 3 | | ALOGPs | ALOGPs-sq | LogD Jejunum & Ileum (pH 6.5) | – | | 0.7513 |  |
| 3 | | ALOGPs | ALOGPs-sq | LogD (pH 7.2) blood | – | | 0.7503 |  |
| 3 | | ALOGPs | ALOGPs-sq | LogD (pH 7.4) blood | – | | 0.7497 |  |
| 3 | | ALOGPs | ALOGPs-sq | LogD (pH 8) colon | – | | 0.7458 |  |
| 3 | | ALOGPs | ALOGPs-sq | Me | – | | 0.7394 |  |
| 4 | | ALOGPs | ALOGPs-sq | nCIC | J3D | | 0.8160 |  |
| 4 | | ALOGPs | ALOGPs-sq | nH | J | | 0.8152 |  |
| 4 | | ALOGPs | ALOGPs-sq | AMW | J | | 0.8151 |  |
| 4 | | ALOGPs | ALOGPs-sq | AMW | J3D | | 0.8141 |  |
| 4 | | ALOGPs | ALOGPs-sq | J3D | Ui | | 0.8140 |  |
| 4 | | ALOGPs | ALOGPs-sq | Density (g/cm3) | J3D | | 0.8138 |  |
| 4 | | ALOGPs | ALOGPs-sq | Density (g/cm3) | J | | 0.8121 |  |
| 4 | | ALOGPs | ALOGPs-sq | Parachor (cm3) | nH | | 0.8099 |  |
| 4 | | ALOGPs | ALOGPs-sq | Molar refractivity (cm3) | nH | | 0.8085 |  |
| 4 | | ALOGPs | ALOGPs-sq | Polarizability (cm3) | nH | | 0.8084 |  |
| 4 | | ALOGPs | ALOGPs-sq | Molecular Volume (cm3) | nH | | 0.8084 |  |
| 4 | | ALOGPs | ALOGPs-sq | Me | Mp | | 0.8080 |  |
| 4 | | ALOGPs | ALOGPs-sq | LogD (pH 6.5) | AMW | | 0.8069 |  |
| 4 | | ALOGPs | ALOGPs-sq | LogD (pH 7.2) | AMW | | 0.8068 |  |
| 4 | | ALOGPs | ALOGPs-sq | LogD (pH 7.4) | AMW | | 0.8068 |  |
| 4 | | ALOGPs | ALOGPs-sq | LogD (pH 8) | nH | | 0.8063 |  |
| 4 | | ALOGPs | ALOGPs-sq | LogD (pH 8) | AMW | | 0.8062 |  |
| 4 | | ALOGPs | ALOGPs-sq | LogD (pH 4.6) | AMW | | 0.8062 |  |
| 4 | | ALOGPs | ALOGPs-sq | LogD (pH 7.4) | nH | | 0.8056 |  |
| 4 | | ALOGPs | ALOGPs-sq | LogD (pH 7.2) | nH | | 0.8054 |  |

Table S2 - 20 Best fitted 3 and 4 parameter models, ranked by R^2^ values.

4 parameter models are fitted with a subset of descriptors.

3.2. **Descriptor Definitions**

ALOGPs – octanol/water partition coefficient

ALOGPs-sq – octanol/water partition coefficient squared

AMW - average molecular weight

ARR – aromatic ratio

De - D total accessibility index / weighted by Sanderson electronegativity

Density (g/cm3)

Du - D total accessibility index / unweighted

E3e - 3rd component accessibility directional WHIM index / weighted by Sanderson electronegativity

E3u - 3rd component accessibility directional WHIM index / unweighted

J - Balaban distance connectivity index

J3D - 3D-Balaban index

LogD (pH 7.2) – distribution constant, calculated at pH 7.2

LogD (pH 7.4) blood – distribution constant, calculated at pH 7.4

LogD (pH 8) colon – distribution constant, calculated at pH 8

LogD duodenum (pH 4.6) – distribution constant, calculated at pH 4.6

LogD Jejunum & Ileum (pH 6.5) – distribution constant, calculated at pH 6.5

Lop - Lopping centric index

Molecular Volume (cm3)

Mp - mean atomic polarizability (scaled on Carbon atom)

Mv - mean atomic van der Waals volume (scaled on Carbon atom)

nCIC - number of rings (cyclomatic number)

nH - number of Hydrogen atoms

Parachor (cm3)

Polarizability (cm3)

Surface Tension (dyne/cm) - surface free energy

Ui - unsaturation index

3.3. **Model Fits**

|  |  | **Coefficients** | | | | | | | | | | | | | | | | | | | | | | | | |
| --- | --- | --- | --- | --- | --- | --- | --- | --- | --- | --- | --- | --- | --- | --- | --- | --- | --- | --- | --- | --- | --- | --- | --- | --- | --- | --- |
|  |  |  | **Linear Fit** | | **Bootstrap** | |  | **Linear Fit** | | **Bootstrap** | |  | **Linear Fit** | | **Bootstrap** | |  | **Linear Fit** | | **Bootstrap** | |  | **Linear Fit** | | **Bootstrap** | |
| **Model parameters** | **R^2 value** | **Intercept** | **2.5%  CI** | **97.5%**  **CI** | **2.5%  CI** | **97.5%**  **CI** | **ALogPs** | **2.5%  CI** | **97.5%**  **CI** | **2.5%  CI** | **97.5%**  **CI** | **ALogPs**  **^2** | **2.5%  CI** | **97.5%**  **CI** | **2.5%  CI** | **97.5%**  **CI** | **3rd Param** | **2.5%  CI** | **97.5%**  **CI** | **2.5%  CI** | **97.5%**  **CI** | **4th param** | **2.5%**  **CI** | **97.5%**  **CI** | **2.5%**  **CI** | **97.5%**  **CI** |
| ALogPs, ALogPs^2  **(1)** | 0.6292 | -0.579 | -1.165 | 0.008 | -1.108 | -0.086 | 1.203 | 0.903 | 1.504 | 0.904 | 1.470 | -0.133 | -0.168 | -0.098 | -0.166 | -0.093 |  |  |  |  |  |  |  |  |  |  |
| ALogPs, ALogPs^2,  nH **(2)** | 0.782 | -1.659 | -2.276 | -1.041 | -2.454 | -1.033 | 1.310 | 1.073 | 1.547 | 1.038 | 1.601 | -0.176 | -0.207 | -0.144 | -0.213 | -0.130 | 0.065 | 0.040 | 0.090 | 0.043 | 0.087 |  |  |  |  |  |
| ALogPs, ALogPs^2,  Mv **(3)** | 0.7901 | 3.362 | 1.838 | 4.887 | 2.159 | 4.419 | 1.372 | 1.135 | 1.61 | 1.126 | 1.579 | -0.158 | -0.186 | -0.129 | -0.19 | -0.123 | -6.616 | -9.063 | -4.168 | -8.432 | -4.473 |  |  |  |  |  |
| ALogPs, ALogPs^2, J3D  **(4)** | 0.789 | -2.372 | -3.176 | -1.569 | -3.071 | -1.648 | 1.339 | 1.104 | 1.574 | 1.109 | 1.556 | -0.148 | -0.175 | -0.120 | -0.177 | -0.115 | 0.733 | 0.461 | 1.006 | 0.495 | 0.960 |  |  |  |  |  |
| ALogPs, ALogPs^2, ARR **(5)** | 0.765 | -0.222 | -0.719 | 0.274 | -0.647 | 0.070 | 1.366 | 1.114 | 1.618 | 1.117 | 1.605 | -0.153 | -0.182 | -0.123 | -0.186 | -0.116 | -1.675 | -2.387 | -0.963 | -2.290 | -1.065 |  |  |  |  |  |
| AlogPs, ALogPs^2, AMW **(6)** | 0.777 | 0.731 | 0.035 | 1.427 | -0.113 | 1.368 | 1.330 | 1.089 | 1.572 | 1.104 | 1.536 | -0.154 | -0.183 | -0.125 | -0.182 | -0.121 | -0.200 | -0.279 | -0.120 | -0.293 | -0.133 |  |  |  |  |  |
| ALogPs, ALogPs^2, nCIC, J3D (**7**) | 0.816 | -5.105 | -7.579 | -2.632 | -7.681 | -2.694 | 1.284 | 1.056 | 1.511 | 1.087 | 1.493 | -0.146 | -0.172 | -0.120 | -0.173 | -0.116 | 0.411 | 0.057 | 0.764 | 0.064 | 0.796 | 1.587 | 0.808 | 2.367 | 0.796 | 2.330 |
| ALogPs, ALogP^2, AMW, J3D (**8**) | 0.814 | -1.018 | -2.453 | 0.417 | -2.309 | 0.429 | 1.361 | 1.137 | 1.586 | 1.123 | 1.591 | -0.154 | -0.181 | -0.127 | -0.185 | -0.119 | -0.110 | -0.209 | -0.011 | -0.218 | -0.051 | 0.475 | 0.127 | 0.823 | 0.148 | 0.781 |
| ALogPs, ALogPs^2, nH, J (**9**) | 0.815 | -3.067 | -4.304 | -1.830 | -4.133 | -1.767 | 1.432 | 1.191 | 1.673 | 1.196 | 1.653 | -0.172 | -0.202 | -0.142 | -0.205 | -0.128 | 0.044 | 0.015 | 0.728 | 0.014 | 0.071 | 0.904 | 0.202 | 1.607 | 0.173 | 1.475 |
| ALogPs, ALogPs^2, Polarizability, nH (**10**) | 0.808 | -0.983 | -1.822 | -0.144 | -1.736 | 0.078 | 1.307 | 1.082 | 1.532 | 1.032 | 1.519 | -0.165 | -0.197 | -0.132 | -0.199 | -0.110 | -0.022 | -0.041 | -0.002 | -0.036 | 0.005 | 0.063 | 0.039 | 0.087 | 0.040 | 0.087 |

Table S3. Coefficients & Confidence intervals for Linear and Bootstrap fits for 2, 3 and 4 parameter models.

3.4. **Model fit plots**

Figure S166. Plots showing the predicted vs actual values for Log(1/EC_50_) for 10 of the models fitted

3.5. **R Code from linear fits**

**R Code and Results from linear fits of 2, 3 and 4 parameter models**

library(boot)

dataset<-read.csv("Tambjamines_New_numbers_classified_cleaned.csv")
ds.sb<-dataset[,c('ï..Compound.no','Ring.substituent', 'NH.substituent','Enamine.Substituent','R.type','Log.1.EC50.','ALOGPs','ALOGPs.sq','nH','Mv','J3D','AMW','J','ARR','nCIC','Polarizability..cm3.')]

plot(ds.sb$ALOGPs, ds.sb$Log.1.EC50., main="Simple plot of Log(1/EC50) against ALOGPs", xlab="ALOGPs", ylab="Log(1/EC50)")
#plots ALOGPs against log(1/ec50)

\# define stats function to give to boot strap, at this stage just the quadratic fit
bs <- function(formula, data, indices) {
d <- data[indices,] # allows boot to select sample
fit <- lm(formula, data=d)
return(coef(fit)) 
}

\# define simpler data frame and give x and y variables
\# gives dataframe with only 2 columns (the necessary columns)
df1 <-data.frame(x=ds.sb$ALOGPs,y=ds.sb$Log.1.EC50.)

\# define the strata
\# limits of ALOGPs are below 1.5 and above 6 in this set
strata <- ifelse(ds.sb$ALOGPs > 1.5, 1,0)+ ifelse(ds.sb$ALOGPs > 6, 1,0)

**#ALOGPs, ALOGPS-sq**

\# do the bootstrap
res_plainfit <-boot(data = df1, statistic = bs, strata = strata, R=999, formula = y ~ x + I(x^2))

\# now evaluate the confidence limit from the boot strap, index = 1 is the intercept, 2 the
\# coefficient of ALOGPs and 3 the coefficient of ALOGPs^2
\# bca - Adjusted Bootstrap percentile interval

> boot.ci(res_plainfit, type="bca",index=1)

BOOTSTRAP CONFIDENCE INTERVAL CALCULATIONS

Based on 999 bootstrap replicates

CALL :

boot.ci(boot.out = res_plainfit, type = "bca", index = 1)

Intervals :

Level	BCa

95%	(-1.1079, -0.0859 )

Calculations and Intervals on Original Scale

> boot.ci(res_plainfit, type="bca",index=2)

BOOTSTRAP CONFIDENCE INTERVAL CALCULATIONS

Based on 999 bootstrap replicates

CALL :

boot.ci(boot.out = res_plainfit, type = "bca", index = 2)

Intervals :

Level	BCa

95%	( 0.904, 1.470 )

Calculations and Intervals on Original Scale

> boot.ci(res_plainfit, type="bca",index=3)

BOOTSTRAP CONFIDENCE INTERVAL CALCULATIONS

Based on 999 bootstrap replicates

CALL :

boot.ci(boot.out = res_plainfit, type = "bca", index = 3)

Intervals :

Level	BCa

95%	(-0.1658, -0.0926 )

Calculations and Intervals on Original Scale

\# do the usual linear least squares for the quadratic
\# lm is linear model using df1 as the data source
fitplain <- lm(y ~ x + I(x^2), data = df1)

\# look at the coefficients and confidence limit from the least squares

> summary(fitplain)

Call:

lm(formula = y ~ x + I(x^2), data = df1)

Residuals:

Min	1Q	Median	3Q	Max

-0.71723 -0.17356 0.02163 0.25389 0.49909

Coefficients:

Estimate Std. Error t value Pr(>|t|)

(Intercept) -0.57873	0.29008 -1.995	0.0529 .

x	1.20333	0.14861	8.097 5.83e-10 ***

I(x^2)	-0.13293	0.01746 -7.613 2.67e-09 ***

---

Signif. codes: 0 ‘***’ 0.001 ‘**’ 0.01 ‘*’ 0.05 ‘.’ 0.1 ‘ ’ 1

Residual standard error: 0.3056 on 40 degrees of freedom

Multiple R-squared: 0.6292,	Adjusted R-squared: 0.6106

F-statistic: 33.93 on 2 and 40 DF, p-value: 2.42e-09confint(fitplain)
2.5 % 97.5 %
(Intercept) -1.1829892 -0.008978968
x 0.9142820 1.515710944
I(x^2) -0.1699811 -0.099315781
>

> confint(fitplain)

2.5 %	97.5 %

(Intercept) -1.1650147 0.00754727

x	0.9029908 1.50367778

I(x^2)	-0.1682162 -0.09763807

**#ALOGPs, ALOGPs-sq, nH**

\# now extend to include nH

dfnH <-data.frame(x=ds.sb$ALOGPs,y=ds.sb$Log.1.EC50., z=ds.sb$nH)

\# usual least square fit and estimates

fitnH <- lm(y ~ x + I(x^2) + z, data = dfnH)

> summary(fitnH)

Call:

lm(formula = y ~ x + I(x^2) + z, data = dfnH)

Residuals:

Min	1Q	Median	3Q	Max

-0.52009 -0.12461 -0.02989 0.14692 0.54953

Coefficients:

Estimate Std. Error t value Pr(>|t|)

(Intercept) -1.65858	0.30541 -5.431 3.19e-06 ***

x	1.30991	0.11713 11.183 9.83e-14 ***

I(x^2)	-0.17580	0.01584 -11.101 1.23e-13 ***

z	0.06525	0.01247	5.233 5.98e-06 ***

---

Signif. codes: 0 ‘***’ 0.001 ‘**’ 0.01 ‘*’ 0.05 ‘.’ 0.1 ‘ ’ 1

Residual standard error: 0.2372 on 39 degrees of freedom

Multiple R-squared: 0.7822,	Adjusted R-squared: 0.7654

F-statistic: 46.67 on 3 and 39 DF, p-value: 5.615e-13

> confint(fitnH)

2.5 %	97.5 %

(Intercept) -2.27633322 -1.0408314

x	1.07298335 1.5468355

I(x^2)	-0.20783293 -0.1437676

z	0.04002894 0.0904637

\# now bootstrap

res_fitnH <-boot(data = dfnH, statistic = bs, strata = strata, R=999, formula = y ~ x + I(x^2)+z)

#res_fitnH is including nH

\# look at confidence intervals (now seem a little different to above)

#uses the bootstrap function

\# index 1 - intercept?

\# index 2 - ALOGPs

\# index 3 - ALOGPs^2

\# index 4 - nH

> boot.ci(res_fitnH, type="bca",index=1)

BOOTSTRAP CONFIDENCE INTERVAL CALCULATIONS

Based on 999 bootstrap replicates

CALL :

boot.ci(boot.out = res_fitnH, type = "bca", index = 1)

Intervals :

Level	BCa

95%	(-2.454, -1.033 )

Calculations and Intervals on Original Scale

> boot.ci(res_fitnH, type="bca",index=2)

BOOTSTRAP CONFIDENCE INTERVAL CALCULATIONS

Based on 999 bootstrap replicates

CALL :

boot.ci(boot.out = res_fitnH, type = "bca", index = 2)

Intervals :

Level	BCa

95%	( 1.038, 1.601 )

Calculations and Intervals on Original Scale

> boot.ci(res_fitnH, type="bca",index=3)

BOOTSTRAP CONFIDENCE INTERVAL CALCULATIONS

Based on 999 bootstrap replicates

CALL :

boot.ci(boot.out = res_fitnH, type = "bca", index = 3)

Intervals :

Level	BCa

95%	(-0.2132, -0.1302 )

Calculations and Intervals on Original Scale

> boot.ci(res_fitnH, type="bca",index=4)

BOOTSTRAP CONFIDENCE INTERVAL CALCULATIONS

Based on 999 bootstrap replicates

CALL :

boot.ci(boot.out = res_fitnH, type = "bca", index = 4)

Intervals :

Level	BCa

95%	( 0.0433, 0.0874 )

Calculations and Intervals on Original Scale

**#ALOGPs, ALOGPs-sq, Mv**

\# now extend to include Mv

dfMv <-data.frame(x=ds.sb$ALOGPs,y=ds.sb$Log.1.EC50., z=ds.sb$Mv)

\# usual least square fit and estimates

fitMv <- lm(y ~ x + I(x^2) + z, data = dfMv)

#lines(ds.sb$ALOGPs[ord],fitted(fitMv)[ord],col="green")

summary(fitMv)

Call: lm(formula = y ~ x + I(x^2) + z, data = dfMv)

Residuals:

Min	1Q	Median	3Q	Max

-0.62519 -0.09623 -0.00267 0.10307 0.50699

Coefficients:

Estimate Std. Error t value Pr(>|t|)

(Intercept) 3.36237	0.75384	4.460 6.76e-05 ***

x	1.37214	0.11736 11.692 2.56e-14 ***

I(x^2)	-0.15793	0.01407 -11.227 8.75e-14 ***

z	-6.61570	1.20981 -5.468 2.83e-06 ***

---

Signif. codes: 0 ‘***’ 0.001 ‘**’ 0.01 ‘*’ 0.05 ‘.’ 0.1 ‘ ’ 1

Residual standard error: 0.2328 on 39 degrees of freedom

Multiple R-squared: 0.7901,	Adjusted R-squared: 0.774

F-statistic: 48.93 on 3 and 39 DF, p-value: 2.732e-13

confint(fitMv)

2.5 %	97.5 %

(Intercept) 1.8375883 4.8871494

x	1.1347599 1.6095207

I(x^2)	-0.1863835 -0.1294758

z	-9.0627723 -4.1686238

\# now bootstrap

res_fitMv <-boot(data = dfMv, statistic = bs, strata = strata, R=999, formula = y ~ x + I(x^2)+z)

#res_fitMv is including Mv

#uses the bootstrap function

\# index 1 - intercept?

\# index 2 - ALOGPs

\# index 3 - ALOGPs^2

\# index 4 - Mv

> boot.ci(res_fitMv, type="bca",index=1)

BOOTSTRAP CONFIDENCE INTERVAL CALCULATIONS

Based on 999 bootstrap replicates

CALL :

boot.ci(boot.out = res_fitMv, type = "bca", index = 1)

Intervals :

Level	BCa

95%	( 2.159, 4.419 )

Calculations and Intervals on Original Scale

> boot.ci(res_fitMv, type="bca",index=2)

BOOTSTRAP CONFIDENCE INTERVAL CALCULATIONS

Based on 999 bootstrap replicates

CALL :

boot.ci(boot.out = res_fitMv, type = "bca", index = 2)

Intervals :

Level	BCa

95%	( 1.126, 1.579 )

Calculations and Intervals on Original Scale

> boot.ci(res_fitMv, type="bca",index=3)

BOOTSTRAP CONFIDENCE INTERVAL CALCULATIONS

Based on 999 bootstrap replicates

CALL :

boot.ci(boot.out = res_fitMv, type = "bca", index = 3)

Intervals :

Level	BCa

95%	(-0.1895, -0.1229 )

Calculations and Intervals on Original Scale

> boot.ci(res_fitMv, type="bca",index=4)

BOOTSTRAP CONFIDENCE INTERVAL CALCULATIONS

Based on 999 bootstrap replicates

CALL :

boot.ci(boot.out = res_fitMv, type = "bca", index = 4)

Intervals :

Level	BCa

95%	(-8.432, -4.473 )

Calculations and Intervals on Original Scale

**#ALOGPs, ALOGPs-sq, J3D**

\# now extend to include J3D

dfJ3D <-data.frame(x=ds.sb$ALOGPs,y=ds.sb$Log.1.EC50., z=ds.sb$J3D)

\# usual least square fit and estimates

fitJ3D <- lm(y ~ x + I(x^2) + z, data = dfJ3D)

#lines(ds.sb$ALOGPs[ord],fitted(fitJ3D)[ord],col="green")

> summary(fitJ3D)

Call: lm(formula = y ~ x + I(x^2) + z, data = dfJ3D)

Residuals:

Min	1Q	Median	3Q	Max

-0.60320 -0.11738 0.00310 0.09931 0.49719

Coefficients:

Estimate Std. Error t value Pr(>|t|)

(Intercept) -2.3723	0.3971 -5.973 5.64e-07 ***

x	1.3394	0.1162 11.527 3.94e-14 ***

I(x^2)	-0.1476	0.0136 -10.850 2.42e-13 ***

z	0.7332	0.1348	5.441 3.09e-06 ***

---

Signif. codes: 0 ‘***’ 0.001 ‘**’ 0.01 ‘*’ 0.05 ‘.’ 0.1 ‘ ’ 1

Residual standard error: 0.2333 on 39 degrees of freedom

Multiple R-squared: 0.7892,	Adjusted R-squared: 0.773

F-statistic: 48.67 on 3 and 39 DF, p-value: 2.971e-13

> confint(fitJ3D)

2.5 %	97.5 %

(Intercept) -3.1755219 -1.5689714

x	1.1043710 1.5744203

I(x^2)	-0.1750866 -0.1200634

z	0.4606703 1.0058329

\# now bootstrap

res_fitJ3D <-boot(data = dfJ3D, statistic = bs, strata = strata, R=999, formula = y ~ x + I(x^2)+z)

#res_fitJ3D is including J3D

\# look at confidence intervals (now seem a little different to above)

#uses the bootstrap function

\# index 1 - intercept?

\# index 2 - ALOGPs

\# index 3 - ALOGPs^2

\# index 4 - J3D

> boot.ci(res_fitJ3D, type="bca",index=1)

BOOTSTRAP CONFIDENCE INTERVAL CALCULATIONS

Based on 999 bootstrap replicates

CALL :

boot.ci(boot.out = res_fitJ3D, type = "bca", index = 1)

Intervals :

Level	BCa

95%	(-3.071, -1.648 )

Calculations and Intervals on Original Scale

> boot.ci(res_fitJ3D, type="bca",index=2)

BOOTSTRAP CONFIDENCE INTERVAL CALCULATIONS

Based on 999 bootstrap replicates

CALL :

boot.ci(boot.out = res_fitJ3D, type = "bca", index = 2)

Intervals :

Level	BCa

95%	( 1.109, 1.556 )

Calculations and Intervals on Original Scale

> boot.ci(res_fitJ3D, type="bca",index=3)

BOOTSTRAP CONFIDENCE INTERVAL CALCULATIONS

Based on 999 bootstrap replicates

CALL :

boot.ci(boot.out = res_fitJ3D, type = "bca", index = 3)

Intervals :

Level	BCa

95%	(-0.1770, -0.1145 )

Calculations and Intervals on Original Scale

> boot.ci(res_fitJ3D, type="bca",index=4)

BOOTSTRAP CONFIDENCE INTERVAL CALCULATIONS

Based on 999 bootstrap replicates

CALL :

boot.ci(boot.out = res_fitJ3D, type = "bca", index = 4)

Intervals :

Level	BCa

95%	( 0.4952, 0.9602 )

Calculations and Intervals on Original Scale

>

**#ALOGPs, ALOGPs-sq, ARR**

\# now extend to include ARR

dfARR <-data.frame(x=ds.sb$ALOGPs,y=ds.sb$Log.1.EC50., z=ds.sb$ARR)

\# usual least square fit and estimates

fitARR <- lm(y ~ x + I(x^2) + z, data = dfARR)

> summary(fitARR)

Call: lm(formula = y ~ x + I(x^2) + z, data = dfARR)

Residuals:

Min	1Q	Median	3Q	Max

-0.65423 -0.09241 0.00022 0.10878 0.51673

Coefficients:

Estimate Std. Error t value Pr(>|t|)

(Intercept) -0.22238	0.24537 -0.906	0.37

x	1.36602	0.12449 10.973 1.73e-13 ***

I(x^2)	-0.15271	0.01467 -10.412 8.05e-13 ***

z	-1.67499	0.35198 -4.759 2.67e-05 ***

---

Signif. codes: 0 ‘***’ 0.001 ‘**’ 0.01 ‘*’ 0.05 ‘.’ 0.1 ‘ ’ 1

Residual standard error: 0.2461 on 39 degrees of freedom

Multiple R-squared: 0.7654,	Adjusted R-squared: 0.7473

F-statistic: 42.41 on 3 and 39 DF, p-value: 2.358e-12

> confint(fitARR)

2.5 %	97.5 %

(Intercept) -0.7186996 0.2739332

x	1.1142092 1.6178228

I(x^2)	-0.1823700 -0.1230402

z	-2.3869397 -0.9630480

\# now bootstrap

res_fitARR <-boot(data = dfARR, statistic = bs, strata = strata, R=999, formula = y ~ x + I(x^2)+z)

#res_fitARR is including ARR

\# look at confidence intervals (now seem a little different to above)

#uses the bootstrap function

\# index 1 - intercept?

\# index 2 - ALOGPs

\# index 3 - ALOGPs^2

\# index 4 - ARR

> boot.ci(res_fitARR, type="bca",index=1)

BOOTSTRAP CONFIDENCE INTERVAL CALCULATIONS

Based on 999 bootstrap replicates

CALL :

boot.ci(boot.out = res_fitARR, type = "bca", index = 1)

Intervals :

Level	BCa

95%	(-0.6466, 0.0697 )

Calculations and Intervals on Original Scale

Some BCa intervals may be unstable

> boot.ci(res_fitARR, type="bca",index=2)

BOOTSTRAP CONFIDENCE INTERVAL CALCULATIONS

Based on 999 bootstrap replicates

CALL :

boot.ci(boot.out = res_fitARR, type = "bca", index = 2)

Intervals :

Level	BCa

95%	( 1.117, 1.605 )

Calculations and Intervals on Original Scale

> boot.ci(res_fitARR, type="bca",index=3)

BOOTSTRAP CONFIDENCE INTERVAL CALCULATIONS

Based on 999 bootstrap replicates

CALL :

boot.ci(boot.out = res_fitARR, type = "bca", index = 3)

Intervals :

Level	BCa

95%	(-0.1863, -0.1160 )

Calculations and Intervals on Original Scale

> boot.ci(res_fitARR, type="bca",index=4)

BOOTSTRAP CONFIDENCE INTERVAL CALCULATIONS

Based on 999 bootstrap replicates

CALL :

boot.ci(boot.out = res_fitARR, type = "bca", index = 4)

Intervals :

Level	BCa

95%	(-2.290, -1.065 )

Calculations and Intervals on Original Scale

**#ALOGPs, ALOGPs-sq, AMW**

\# now extend to include AMW

dfAMW <-data.frame(x=ds.sb$ALOGPs,y=ds.sb$Log.1.EC50., z=ds.sb$AMW)

\# usual least square fit and estimates

fitAMW <- lm(y ~ x + I(x^2) + z, data = dfAMW)

> summary(fitAMW)

Call: lm(formula = y ~ x + I(x^2) + z, data = dfAMW)

Residuals:

Min	1Q	Median	3Q	Max

-0.61861 -0.12959 0.02152 0.15637 0.41613

Coefficients:

Estimate Std. Error t value Pr(>|t|)

(Intercept) 0.73091	0.34409	2.124	0.04 *

x	1.33046	0.11940 11.143 1.09e-13 ***

I(x^2)	-0.15404	0.01433 -10.747 3.20e-13 ***

z	-0.19952	0.03928 -5.080 9.72e-06 ***

---

Signif. codes: 0 ‘***’ 0.001 ‘**’ 0.01 ‘*’ 0.05 ‘.’ 0.1 ‘ ’ 1

Residual standard error: 0.2401 on 39 degrees of freedom

Multiple R-squared: 0.7768,	Adjusted R-squared: 0.7597

F-statistic: 45.25 on 3 and 39 DF, p-value: 8.955e-13

> confint(fitAMW)

2.5 %	97.5 %

(Intercept) 0.03492571 1.4268963

x	1.08895355 1.5719752

I(x^2)	-0.18302806 -0.1250454

z	-0.27896332 -0.1200808

\# now bootstrap

res_fitAMW <-boot(data = dfAMW, statistic = bs, strata = strata, R=999, formula = y ~ x + I(x^2)+z)

#res_fitAMW is including AMW

\# look at confidence intervals (now seem a little different to above)

#uses the bootstrap function

\# index 1 - intercept?

\# index 2 - ALOGPs

\# index 3 - ALOGPs^2

\# index 4 - AMW

> boot.ci(res_fitAMW, type="bca",index=1)

BOOTSTRAP CONFIDENCE INTERVAL CALCULATIONS

Based on 999 bootstrap replicates

CALL :

boot.ci(boot.out = res_fitAMW, type = "bca", index = 1)

Intervals :

Level	BCa

95%	(-0.1126, 1.3684 )

Calculations and Intervals on Original Scale

Some BCa intervals may be unstable

> boot.ci(res_fitAMW, type="bca",index=2)

BOOTSTRAP CONFIDENCE INTERVAL CALCULATIONS

Based on 999 bootstrap replicates

CALL :

boot.ci(boot.out = res_fitAMW, type = "bca", index = 2)

Intervals :

Level	BCa

95%	( 1.104, 1.536 )

Calculations and Intervals on Original Scale

> boot.ci(res_fitAMW, type="bca",index=3)

BOOTSTRAP CONFIDENCE INTERVAL CALCULATIONS

Based on 999 bootstrap replicates

CALL :

boot.ci(boot.out = res_fitAMW, type = "bca", index = 3)

Intervals :

Level	BCa

95%	(-0.1820, -0.1212 )

Calculations and Intervals on Original Scale

> boot.ci(res_fitAMW, type="bca",index=4)

BOOTSTRAP CONFIDENCE INTERVAL CALCULATIONS

Based on 999 bootstrap replicates

CALL :

boot.ci(boot.out = res_fitAMW, type = "bca", index = 4)

Intervals :

Level	BCa

95%	(-0.2931, -0.1331 )

Calculations and Intervals on Original Scale

Some BCa intervals may be unstable

**#ALOGPS, ALOGPs-sq, nCIC, J3D**

#ALogPs, ALogPs^2, nCIC, J3D

dfnCIC_J3D <-data.frame(x=ds.sb$ALOGPs,y=ds.sb$Log.1.EC50., z=ds.sb$nCIC, w=ds.sb$J3D)

\# usual least square fit and estimates

fitnCIC_J3D <- lm(y ~ x + I(x^2) + z + w, data = dfnCIC_J3D)

> summary(fitnCIC_J3D)

Call: lm(formula = y ~ x + I(x^2) + z + w, data = dfnCIC_J3D)

Residuals:

Min	1Q	Median	3Q	Max

-0.49204 -0.14596 0.03286 0.09284 0.47415

Coefficients:

Estimate Std. Error t value Pr(>|t|)

(Intercept) -5.1054	1.2220 -4.178 0.000166 ***

x	1.2837	0.1125 11.409 7.73e-14 ***

I(x^2)	-0.1457	0.0129 -11.295 1.04e-13 ***

z	0.4105	0.1746	2.351 0.024036 *

w	1.5873	0.3851	4.122 0.000196 ***

---

Signif. codes: 0 ‘***’ 0.001 ‘**’ 0.01 ‘*’ 0.05 ‘.’ 0.1 ‘ ’ 1

Residual standard error: 0.2209 on 38 degrees of freedom

Multiple R-squared: 0.816,	Adjusted R-squared: 0.7966

F-statistic: 42.12 on 4 and 38 DF, p-value: 1.784e-13

> confint(fitnCIC_J3D)

2.5 %	97.5 %

(Intercept) -7.57929116 -2.6315691

x	1.05592808 1.5114640

I(x^2)	-0.17181680 -0.1195890

z	0.05696876 0.7640868

w	0.80774675 2.3668022

\# now bootstrap

res_fitnCIC_J3D <-boot(data = dfnCIC_J3D, statistic = bs, strata = strata, R=999, formula = y ~ x + I(x^2)+z +w)

#res_fitnCIC_J3D is including nCIC_J3D

\# look at confidence intervals - uses the bootstrap function

\# index 1 - intercept?

\# index 2 - ALOGPs

\# index 3 - ALOGPs^2

\# index 4 - nCIC

\# index 5 - J3D

> boot.ci(res_fitnCIC_J3D, type="bca",index=1)

BOOTSTRAP CONFIDENCE INTERVAL CALCULATIONS

Based on 999 bootstrap replicates

CALL :

boot.ci(boot.out = res_fitnCIC_J3D, type = "bca", index = 1)

Intervals :

Level	BCa

95%	(-7.681, -2.694 )

Calculations and Intervals on Original Scale

> boot.ci(res_fitnCIC_J3D, type="bca",index=2)

BOOTSTRAP CONFIDENCE INTERVAL CALCULATIONS

Based on 999 bootstrap replicates

CALL :

boot.ci(boot.out = res_fitnCIC_J3D, type = "bca", index = 2)

Intervals :

Level	BCa

95%	( 1.087, 1.493 )

Calculations and Intervals on Original Scale

> boot.ci(res_fitnCIC_J3D, type="bca",index=3)

BOOTSTRAP CONFIDENCE INTERVAL CALCULATIONS

Based on 999 bootstrap replicates

CALL :

boot.ci(boot.out = res_fitnCIC_J3D, type = "bca", index = 3)

Intervals :

Level	BCa

95%	(-0.1730, -0.1161 )

Calculations and Intervals on Original Scale

> boot.ci(res_fitnCIC_J3D, type="bca",index=4)

BOOTSTRAP CONFIDENCE INTERVAL CALCULATIONS

Based on 999 bootstrap replicates

CALL :

boot.ci(boot.out = res_fitnCIC_J3D, type = "bca", index = 4)

Intervals :

Level	BCa

95%	( 0.0638, 0.7957 )

Calculations and Intervals on Original Scale

> boot.ci(res_fitnCIC_J3D, type="bca",index=5)

BOOTSTRAP CONFIDENCE INTERVAL CALCULATIONS

Based on 999 bootstrap replicates

CALL :

boot.ci(boot.out = res_fitnCIC_J3D, type = "bca", index = 5)

Intervals :

Level	BCa

95%	( 0.796, 2.330 )

Calculations and Intervals on Original Scale

**#ALOGPs, ALOGPs-sq, AMW, J3D**

#ALogPs, ALogPs^2, AMW, J3D

dfAMW_J3D <-data.frame(x=ds.sb$ALOGPs,y=ds.sb$Log.1.EC50., z=ds.sb$AMW, w=ds.sb$J3D)

\# usual least square fit and estimates

fitAMW_J3D <- lm(y ~ x + I(x^2) + z + w, data = dfAMW_J3D)

> summary(fitAMW_J3D)

Call: lm(formula = y ~ x + I(x^2) + z + w, data = dfAMW_J3D)

Residuals:

Min	1Q	Median	3Q	Max

-0.58907 -0.10352 0.01421 0.08473 0.47939

Coefficients:

Estimate Std. Error t value Pr(>|t|)

(Intercept) -1.01799	0.70868 -1.436 0.15905

x	1.36148	0.11096 12.270 8.67e-15 ***

I(x^2)	-0.15404	0.01325 -11.625 4.43e-14 ***

z	-0.10996	0.04868 -2.259 0.02973 *

w	0.47467	0.17187	2.762 0.00881 **

---

Signif. codes: 0 ‘***’ 0.001 ‘**’ 0.01 ‘*’ 0.05 ‘.’ 0.1 ‘ ’ 1

Residual standard error: 0.2219 on 38 degrees of freedom

Multiple R-squared: 0.8141,	Adjusted R-squared: 0.7946

F-statistic: 41.61 on 4 and 38 DF, p-value: 2.144e-13

> confint(fitAMW_J3D)

2.5 %	97.5 %

(Intercept) -2.4526500 0.41666156

x	1.1368500 1.58610083

I(x^2)	-0.1808690 -0.12721712

z	-0.2085162 -0.01140107

w	0.1267242 0.82260733

\# now bootstrap

res_fitAMW_J3D <-boot(data = dfAMW_J3D, statistic = bs, strata = strata, R=999, formula = y ~ x + I(x^2)+z +w)

#res_fitAMW_J3D is including AMW_J3D

\# look at confidence intervals - uses the bootstrap function

\# index 1 - intercept?

\# index 2 - ALOGPs

\# index 3 - ALOGPs^2

\# index 4 - AMW

\# index 5 - J3D

> boot.ci(res_fitAMW_J3D, type="bca",index=1)

BOOTSTRAP CONFIDENCE INTERVAL CALCULATIONS

Based on 999 bootstrap replicates

CALL :

boot.ci(boot.out = res_fitAMW_J3D, type = "bca", index = 1)

Intervals :

Level	BCa

95%	(-2.309, 0.429 )

Calculations and Intervals on Original Scale

> boot.ci(res_fitAMW_J3D, type="bca",index=2)

BOOTSTRAP CONFIDENCE INTERVAL CALCULATIONS

Based on 999 bootstrap replicates

CALL :

boot.ci(boot.out = res_fitAMW_J3D, type = "bca", index = 2)

Intervals :

Level	BCa

95%	( 1.123, 1.591 )

Calculations and Intervals on Original Scale

> boot.ci(res_fitAMW_J3D, type="bca",index=3)

BOOTSTRAP CONFIDENCE INTERVAL CALCULATIONS

Based on 999 bootstrap replicates

CALL :

boot.ci(boot.out = res_fitAMW_J3D, type = "bca", index = 3)

Intervals :

Level	BCa

95%	(-0.1852, -0.1194 )

Calculations and Intervals on Original Scale

> boot.ci(res_fitAMW_J3D, type="bca",index=4)

BOOTSTRAP CONFIDENCE INTERVAL CALCULATIONS

Based on 999 bootstrap replicates

CALL :

boot.ci(boot.out = res_fitAMW_J3D, type = "bca", index = 4)

Intervals :

Level	BCa

95%	(-0.2182, -0.0512 )

Calculations and Intervals on Original Scale

> boot.ci(res_fitAMW_J3D, type="bca",index=5)

BOOTSTRAP CONFIDENCE INTERVAL CALCULATIONS

Based on 999 bootstrap replicates

CALL :

boot.ci(boot.out = res_fitAMW_J3D, type = "bca", index = 5)

Intervals :

Level	BCa

95%	( 0.1479, 0.7805 )

Calculations and Intervals on Original Scale

**#ALOGPs, ALOGPs-sq, nH, J**

#ALogPs, ALogPs^2, nH, J

dfnH_J <-data.frame(x=ds.sb$ALOGPs,y=ds.sb$Log.1.EC50., z=ds.sb$nH, w=ds.sb$J)

\# usual least square fit and estimates

fitnH_J <- lm(y ~ x + I(x^2) + z + w, data = dfnH_J)

> summary(fitnH_J)

Call: lm(formula = y ~ x + I(x^2) + z + w, data = dfnH_J)

Residuals:

Min	1Q	Median	3Q	Max

-0.51391 -0.10098 -0.02125 0.07999 0.54360

Coefficients:

Estimate Std. Error t value Pr(>|t|)

(Intercept) -3.06707	0.61117 -5.018 1.25e-05 ***

x	1.43233	0.11898 12.039 1.55e-14 ***

I(x^2)	-0.17239	0.01484 -11.620 4.49e-14 ***

z	0.04414	0.01418	3.113 0.00351 **

w	0.90448	0.34719	2.605 0.01304 *

---

Signif. codes: 0 ‘***’ 0.001 ‘**’ 0.01 ‘*’ 0.05 ‘.’ 0.1 ‘ ’ 1

Residual standard error: 0.2213 on 38 degrees of freedom

Multiple R-squared: 0.8152,	Adjusted R-squared: 0.7957

F-statistic: 41.9 on 4 and 38 DF, p-value: 1.933e-13

> confint(fitnH_J)

2.5 %	97.5 %

(Intercept) -4.30432090 -1.82982567

x	1.19146973 1.67318501

I(x^2)	-0.20242439 -0.14235652

z	0.01543404 0.07283738

w	0.20163869 1.60732816

>

\# now bootstrap

res_fitnH_J <-boot(data = dfnH_J, statistic = bs, strata = strata, R=999, formula = y ~ x + I(x^2)+z +w)

#res_fitnH_J is including nH_J

\# look at confidence intervals - uses the bootstrap function

\# index 1 - intercept?

\# index 2 - ALOGPs

\# index 3 - ALOGPs^2

\# index 4 - nH

\# index 5 - J

> boot.ci(res_fitnH_J, type="bca",index=1)

BOOTSTRAP CONFIDENCE INTERVAL CALCULATIONS

Based on 999 bootstrap replicates

CALL :

boot.ci(boot.out = res_fitnH_J, type = "bca", index = 1)

Intervals :

Level	BCa

95%	(-4.133, -1.767 )

Calculations and Intervals on Original Scale

> boot.ci(res_fitnH_J, type="bca",index=2)

BOOTSTRAP CONFIDENCE INTERVAL CALCULATIONS

Based on 999 bootstrap replicates

CALL :

boot.ci(boot.out = res_fitnH_J, type = "bca", index = 2)

Intervals :

Level	BCa

95%	( 1.196, 1.653 )

Calculations and Intervals on Original Scale

> boot.ci(res_fitnH_J, type="bca",index=3)

BOOTSTRAP CONFIDENCE INTERVAL CALCULATIONS

Based on 999 bootstrap replicates

CALL :

boot.ci(boot.out = res_fitnH_J, type = "bca", index = 3)

Intervals :

Level	BCa

95%	(-0.2047, -0.1278 )

Calculations and Intervals on Original Scale

> boot.ci(res_fitnH_J, type="bca",index=4)

BOOTSTRAP CONFIDENCE INTERVAL CALCULATIONS

Based on 999 bootstrap replicates

CALL :

boot.ci(boot.out = res_fitnH_J, type = "bca", index = 4)

Intervals :

Level	BCa

95%	( 0.0139, 0.0705 )

Calculations and Intervals on Original Scale

> boot.ci(res_fitnH_J, type="bca",index=5)

BOOTSTRAP CONFIDENCE INTERVAL CALCULATIONS

Based on 999 bootstrap replicates

CALL :

boot.ci(boot.out = res_fitnH_J, type = "bca", index = 5)

Intervals :

Level	BCa

95%	( 0.1729, 1.4745 )

Calculations and Intervals on Original Scale

**#ALOGPs, ALOGPs-sq, Polarizability, nH**

#ALogPs, ALogPs^2, Polarizability..cm3., nH

dfPol_nH <-data.frame(x=ds.sb$ALOGPs,y=ds.sb$Log.1.EC50., z=ds.sb$Polarizability..cm3., w=ds.sb$nH)

\# usual least square fit and estimates

fitPol_nH <- lm(y ~ x + I(x^2) + z + w, data = dfPol_nH)

> summary(fitPol_nH)

Call: lm(formula = y ~ x + I(x^2) + z + w, data = dfPol_nH)

Residuals:

Min	1Q	Median	3Q	Max

-0.57511 -0.10224 -0.02149 0.08645 0.58694

Coefficients:

Estimate Std. Error t value Pr(>|t|)

(Intercept) -0.983048	0.414418 -2.372	0.0229 *

x	1.306968	0.111287 11.744 3.26e-14 ***

I(x^2)	-0.164507	0.015837 -10.387 1.18e-12 ***

z	-0.021601	0.009462 -2.283	0.0281 *

w	0.062608	0.011900	5.261 5.86e-06 ***

---

Signif. codes: 0 ‘***’ 0.001 ‘**’ 0.01 ‘*’ 0.05 ‘.’ 0.1 ‘ ’ 1

Residual standard error: 0.2253 on 38 degrees of freedom

Multiple R-squared: 0.8084,	Adjusted R-squared: 0.7883

F-statistic: 40.09 on 4 and 38 DF, p-value: 3.786e-13

> confint(fitPol_nH)

2.5 %	97.5 %

(Intercept) -1.82199334 -0.144102492

x	1.08167950 1.532255543

I(x^2)	-0.19656852 -0.132446274

z	-0.04075559 -0.002446523

w	0.03851716 0.086699013

fitPol_nH_pred<-fitted(fitPol_nH) #predicted values of fit3

\# plot(fitPol_nH, dfPol_nH$y, main="fitPol_nH predicted vs actual") # plots predicted vs actual values

\# now bootstrap

res_fitPol_nH <-boot(data = dfPol_nH, statistic = bs, strata = strata, R=999, formula = y ~ x + I(x^2)+z +w)

#res_fitPol_nH is including Pol_nH

\# look at confidence intervals - uses the bootstrap function

\# index 1 - intercept?

\# index 2 - ALOGPs

\# index 3 - ALOGPs^2

\# index 4 - Molec.Volume..cm3.

\# index 5 - nH

> boot.ci(res_fitPol_nH, type="bca",index=1)

BOOTSTRAP CONFIDENCE INTERVAL CALCULATIONS

Based on 999 bootstrap replicates

CALL :

boot.ci(boot.out = res_fitPol_nH, type = "bca", index = 1)

Intervals :

Level	BCa

95%	(-1.7356, 0.0784 )

Calculations and Intervals on Original Scale

> boot.ci(res_fitPol_nH, type="bca",index=2)

BOOTSTRAP CONFIDENCE INTERVAL CALCULATIONS

Based on 999 bootstrap replicates

CALL :

boot.ci(boot.out = res_fitPol_nH, type = "bca", index = 2)

Intervals :

Level	BCa

95%	( 1.032, 1.519 )

Calculations and Intervals on Original Scale

> boot.ci(res_fitPol_nH, type="bca",index=3)

BOOTSTRAP CONFIDENCE INTERVAL CALCULATIONS

Based on 999 bootstrap replicates

CALL :

boot.ci(boot.out = res_fitPol_nH, type = "bca", index = 3)

Intervals :

Level	BCa

95%	(-0.1990, -0.1102 )

Calculations and Intervals on Original Scale

> boot.ci(res_fitPol_nH, type="bca",index=4)

BOOTSTRAP CONFIDENCE INTERVAL CALCULATIONS

Based on 999 bootstrap replicates

CALL :

boot.ci(boot.out = res_fitPol_nH, type = "bca", index = 4)

Intervals :

Level	BCa

95%	(-0.0363, 0.0050 )

Calculations and Intervals on Original Scale

> boot.ci(res_fitPol_nH, type="bca",index=5)

BOOTSTRAP CONFIDENCE INTERVAL CALCULATIONS

Based on 999 bootstrap replicates

CALL :

boot.ci(boot.out = res_fitPol_nH, type = "bca", index = 5)

Intervals :

Level	BCa

95%	( 0.0399, 0.0865 )

Calculations and Intervals on Original Scale

>

3.6. **R Code from mixed effect fits**

**R Code & results from mixed effect fits using lme4 library.**

dataset<-read.csv("Tambjamines_New_numbers_classified_cleaned.csv")
ds_alkyl <- subset(dataset, R.type =="alkyl")

ds_OMe_alkyl <- subset(dataset, R.type =="alkyl" & Ring.substituent == "OMe")
#subset

#plot the points for subset 1 and then label them by their Rgroup (NH.substituent) + colour by top substituent

#plot with labels and title, labels each point with Nh.substituent and colour by top substituent
plot(ds_OMe_alkyl$ALOGPs,ds_OMe_alkyl$Log.1.EC50., main="Plot of Log(1/EC50) against ALOGPs, 
alkyl chain only, OMe ring substituent", ylim=c(0.4,3.0), xlab="ALOGPs", ylab="Log(1/EC50)", text(ds_OMe_alkyl$ALOGPs,ds_OMe_alkyl$Log.1.EC50., ds_OMe_alkyl$NH.substituent, cex=0.5, pos=3), col=(ifelse(ds_OMe_alkyl$Enamine.Substituent=='NH', "red", "blue")))

plot(ds_OBn_alkyl$ALOGPs,ds_OBn_alkyl$Log.1.EC50., main="Plot of Log(1/EC50) against ALOGPs, 
alkyl chain only, OBn ring substituent", ylim=c(0.4,3.0), xlab="ALOGPs", ylab="Log(1/EC50)", text(ds_OBn_alkyl$ALOGPs,ds_OBn_alkyl$Log.1.EC50., ds_OBn_alkyl$NH.substituent, cex=0.5, pos=3), col=(ifelse(ds_OBn_alkyl$Enamine.Substituent=='NH', "red", "blue")))

#plot with the 2 different Enamine.Substituents in different colours

plot(ds_alkyl$ALOGPs,ds_alkyl$Log.1.EC50.,col=ds_alkyl$Enamine.Substituent, pch=c(16, 17)[as.numeric(ds_alkyl$Ring.substituent)], main="Plot of Log(1/EC50) vs ALOGPs - alkyl R chain", sub="Points coloured by Enamine Substituent type", ylim=c(0.4,3.0),xlab="ALOGPs",ylab="Log(1/EC50)")
\# triangle – OMe
\# circle – OBn
\# black – NH
\# green - NH-Ph

> # Try a parabolic fit, with the maximum and curator the same for both sets but different intercept
> # This uses fixed and random effects by group so use lme4 library 
> 
> library(lme4)
> 
> # formula has the random or grouping effects of Enamine.Substituent
> fit_l_alkyl <- lmer(data = ds_alkyl, Log.1.EC50. ~ (1|Enamine.Substituent) + ALOGPs + I(ALOGPs^2))
> 
> summary(fit_l_alkyl)
Linear mixed model fit by REML ['lmerMod']
Formula: Log.1.EC50. ~ (1 | Enamine.Substituent) + ALOGPs + I(ALOGPs^2)
Data: ds_alkyl

REML criterion at convergence: 16.6

Scaled residuals:

Min	1Q Median	3Q	Max

-2.3650 -0.3438 0.1081 0.5481 1.6244

Random effects:

Groups	Name	Variance Std.Dev.

Enamine.Substituent (Intercept) 0.03059 0.1749

Residual	0.06238 0.2498

Number of obs: 28, groups: Enamine.Substituent, 2

Fixed effects:

Estimate Std. Error t value

(Intercept) -0.73933	0.32341 -2.286

ALOGPs	1.37279	0.14548	9.436

I(ALOGPs^2) -0.15605	0.01661 -9.393

Correlation of Fixed Effects:

(Intr) ALOGPs

ALOGPs	-0.881

I(ALOGPs^2) 0.814 -0.979

> 
> coef(fit_l_alkyl)
$Enamine.Substituent

(Intercept)	ALOGPs I(ALOGPs^2)

NH	-0.6254821 1.372787 -0.1560464

NH-Ph -0.8531819 1.372787 -0.1560464

attr(,"class")

[1] "coef.mer"
> 
> # calculate their 3 fitted curves and plot
> # define a fine x grid to calculate the curves
> x <- (0:80)/10
> 
> # coef(fit_l_alkyl) gives the coefficients for each group (fixed and random combined). 
> # (coef(fit_l_alkyl)$Enamine.Substituent)gives the table of pluses in effect a matrix from which we can 
> # extract the numbers, first index is the group and can be a numeric index or use the name
> # (i.e. "NH") and second index is the coefficient (intercept, ALOGP etc)
> 
> # coef(fit_l_alkyl) is a matrix of coefficients?
> 
> # y1 takes the first line - NH
> y1 <- (coef(fit_l_alkyl)$Enamine.Substituent)[1,1]+(coef(fit_l_alkyl)$Enamine.Substituent)[1,2]*x+(coef(fit_l_alkyl)$Enamine.Substituent)[1,3]*x*x
> 
> 
> #add line to the plot
> lines(x,y1,col="black")
> 
> # y2 takes the second line - NH-Ph
> y2 <- (coef(fit_l_alkyl)$Enamine.Substituent)[2,1]+(coef(fit_l_alkyl)$Enamine.Substituent)[2,2]*x+(coef(fit_l_alkyl)$Enamine.Substituent)[2,3]*x*x
> 
> lines(x,y2,col="green")
>  
> 
> #
> #Just the OMe subset
> #
> #plot with the 2 different Enamine.Substituents in different colours
> plot(ds_OMe_alkyl$ALOGPs,ds_OMe_alkyl$Log.1.EC50.,col=ds_OMe_alkyl$Enamine.Substituent, pch=c(16, 17)[as.numeric(ds_OMe_alkyl$Ring.substituent)], main="Plot of Log(1/EC50) vs ALOGPs - 
+ alkyl R chain, OMe ring substituent", sub="Points coloured by Enamine Substituent type", ylim=c(0.4,3.0),xlab="ALOGPs",ylab="Log(1/EC50)")
> # triangle - OMe
> # black - NH
> # green - NH-Ph
> 
> 
> # Try a parabolic fit, with the maximum and curator the same for both sets but different intercept
> # This uses fixed and random effects by group so use lme4 library 
> 
> library(lme4)
> 
> # formula has the random(?) or grouping effects of Top.Substituent
> fit_l_OMe_alkyl <- lmer(data = ds_OMe_alkyl, Log.1.EC50. ~ (1|Top.Substituent) + ALOGPs + I(ALOGPs^2))
> 
> summary(fit_l_OMe_alkyl)
Linear mixed model fit by REML ['lmerMod']
Formula: Log.1.EC50. ~ (1 | Enamine.Substituent) + ALOGPs + I(ALOGPs^2)
Data: ds_OMe_alkyl

REML criterion at convergence: -5.4

Scaled residuals:

Min	1Q Median	3Q	Max

-1.3440 -0.5360 -0.1963 0.2020 2.1088

Random effects:

Groups	Name	Variance Std.Dev.

Enamine.Substituent (Intercept) 0.06455 0.2541

Residual	0.01738 0.1318

Number of obs: 20, groups: Enamine.Substituent, 2

Fixed effects:

Estimate Std. Error t value

(Intercept) -1.13221	0.24732 -4.578

ALOGPs	1.63726	0.08750 18.712

I(ALOGPs^2) -0.19202	0.01069 -17.960

Correlation of Fixed Effects:

(Intr) ALOGPs

ALOGPs	-0.649

I(ALOGPs^2) 0.586 -0.971

> coef(fit_l_OMe_alkyl)

$Enamine.Substituent

(Intercept)	ALOGPs I(ALOGPs^2)

NH	-0.9556585 1.637261 -0.1920198

NH-Ph -1.3087691 1.637261 -0.1920198

attr(,"class")

[1] "coef.mer"

> 
> # calculate their 3 fitted curves and plot
> # define a fine x grid to calculate the curves
> x <- (0:80)/10
> 
> # coef(fit_l_OMe_alkyl) gives the coefficients for each group (fixed and random combined). 
> # (coef(fit_l_OMe_alkyl)$Enamine.Substituent)gives the table of pluses in effect a matrix from which we can 
> # extract the numbers, first index is the group and can be a numeric index or use the name
> # (i.e. "NH") and second index is the coefficient (intercept, ALOGP etc)
> 
> # coef(fit_l_OMe_alkyl) is a matrix of coefficients?
> 
> # y1 takes the first line - NH
> y1 <- (coef(fit_l_OMe_alkyl)$Enamine.Substituent)[1,1]+(coef(fit_l_OMe_alkyl)$Enamine.Substituent)[1,2]*x+(coef(fit_l_OMe_alkyl)$Enamine.Substituent)[1,3]*x*x
> 
> 
> #add line to the plot
> lines(x,y1,col="black")
> 
> # y2 takes the second line - NH-Ph
> y2 <- (coef(fit_l_OMe_alkyl)$Enamine.Substituent)[2,1]+(coef(fit_l_OMe_alkyl)$Enamine.Substituent)[2,2]*x+(coef(fit_l_OMe_alkyl)$Enamine.Substituent)[2,3]*x*x
> 
> lines(x,y2,col="green")

1. a) K. Dairi, S. Tripathy, G. Attardo and J.–F. Lavallee, *Tetrahedron Lett*., 2006, **47**, 2605–2606; b) P. Iglesias Hernández, D. Moreno, A. Araujo Javier, T. Torroba, R. Pérez-Tomás, R. Quesada, *Chem. Commun.*, 2012, **48**, 1556-1558
2. E. Hernando, V. Soto-Cerrato, S. Cortés-Arroyo, R. Pérez-Tomás and R. Quesada, ***Org. Biomol. Chem.***, 2014, **12**, 1771–1778
3. [V. Saggiomo](http://pubs.rsc.org/en/results?searchtext=Author%3AVittorio Saggiomo), [S. Otto](http://pubs.rsc.org/en/results?searchtext=Author%3ASijbren Otto), [I. Marques](http://pubs.rsc.org/en/results?searchtext=Author%3AIgor Marques), [V. Félix](http://pubs.rsc.org/en/results?searchtext=Author%3AVítor Félix), [T. Torroba](http://pubs.rsc.org/en/results?searchtext=Author%3ATomás Torroba) and [R. Quesada](http://pubs.rsc.org/en/results?searchtext=Author%3ARoberto Quesada), ***Chem. Commun.***, 2012, **48**, 5274–5276
4. D. M. Pinkerton, M. G. Banwell and A. C. Willis, *Org. Lett*., 2007, **9**, 5127–5130
